# Mechanistic Insights into the Ene-Reductase-Catalyzed Promiscuous Reduction of Oximes to Amines

**DOI:** 10.1021/acscatal.2c06137

**Published:** 2023-02-06

**Authors:** Willem B. Breukelaar, Nakia Polidori, Amit Singh, Bastian Daniel, Silvia M. Glueck, Karl Gruber, Wolfgang Kroutil

**Affiliations:** † Department of Chemistry, NAWI Graz, University of Graz, Heinrichstraße 28, 8010 Graz, Austria; ‡ Institute of Molecular Biosciences, University of Graz, Humboldtstraße 50, 8010 Graz, Austria; § Field of Excellence BioHealth, University of Graz, 8010 Graz, Austria; ∥ BioTechMed Graz, 8010 Graz, Austria

**Keywords:** oxime, reduction, mechanism, ene-reductase, biocatalysis

## Abstract

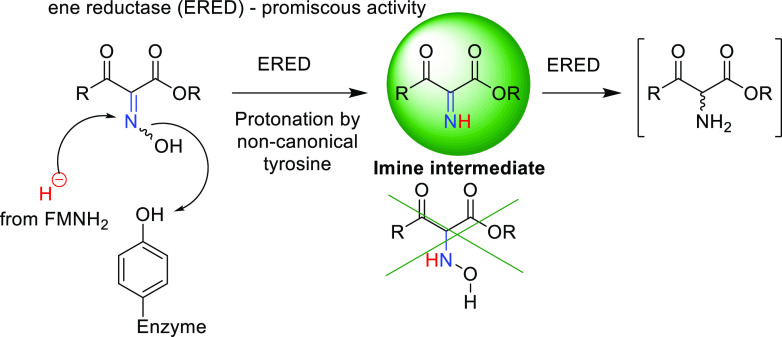

The biocatalytic reduction of the oxime moiety to the corresponding amine group has only recently been found to be a promiscuous activity of ene-reductases transforming α-oximo β-keto esters. However, the reaction pathway of this two-step reduction remained elusive. By studying the crystal structures of enzyme oxime complexes, analyzing molecular dynamics simulations, and investigating biocatalytic cascades and possible intermediates, we obtained evidence that the reaction proceeds *via* an imine intermediate and not *via* the hydroxylamine intermediate. The imine is reduced further by the ene-reductase to the amine product. Remarkably, a non-canonical tyrosine residue was found to contribute to the catalytic activity of the ene-reductase OPR3, protonating the hydroxyl group of the oxime in the first reduction step.

## Introduction

**Scheme 1:**
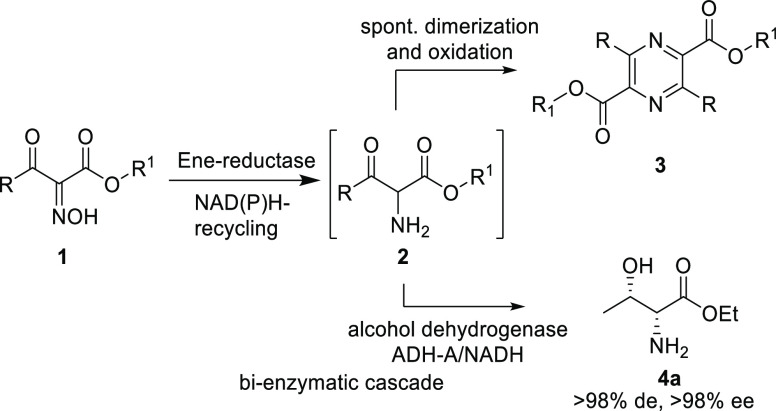
Ene-Reductase-Catalyzed Transformations of α-Oximo β-Keto Esters

Promiscuous and new-to-nature enzymatic activities recently obtained increased attention since they allow expansion of the repertoire of biocatalytic transformations.^1−10^ Although wild-type microorganisms and cell preparations have been reported to transform oximes to the corresponding carbonyl compounds,^11−15^ pyrazines,^16^ or hydroxylamines/amines,^17−19^ the reduction of an oxime moiety using a defined enzyme has only been reported very recently as a promiscuous activity.^20^ Thereby, α-oximo β-keto esters **1** were reduced by ene-reductases from the old yellow enzyme family (EREDs^21−31^) to the presumed corresponding α-amino intermediate **2** (Scheme 1),^20^ which spontaneously dimerized and oxidized, leading finally to the pyrazine product **3**. Intermediate **2** could never be detected, neither by chromatographic and mass spectroscopic methods nor by NMR. Alternatively, the keto moiety of intermediate **2** of a selected substrate was reduced *via* a bi-enzymatic cascade by the alcohol dehydrogenase ADH-A to afford the d-threonine ester **4a**.

**Scheme 2:**
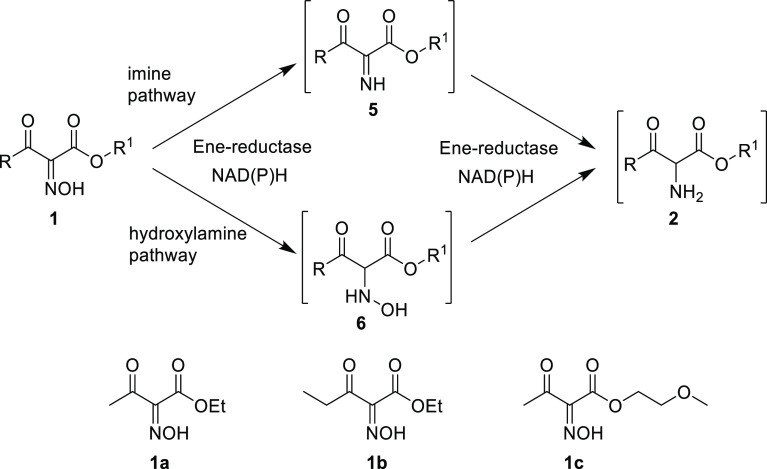
Possible Intermediates in the Reduction of Oxime **1** to Amine **2**

Elucidation of the mechanism of an enzyme-catalyzed reaction, as well as the amino acid residues involved, does not only lead to a better understanding of nature’s catalysts but can also be exploited to improve the efficiency and scope of a biocatalytic reaction.^32−35^ As the reduction of the oxime group of substrate **1** involves two reduction steps to get to the amine moiety in intermediate **2**, the questions remained whether a hydroxylamine or an imine is the intermediate (Scheme 2) and how the enzyme catalyzes these steps.

## Results and Discussion

To identify the reaction pathway of the ene-reductase-catalyzed reduction of the oxime moiety of **1** to the intermediate **2** going either *via* the imine or *via* the hydroxylamine moiety catalyzed by EREDs (Scheme 2), it was envisioned that indications might be obtained by co-crystallization or crystallization, followed by soaking with selected substrates **1a–c** using the EREDs OPR3 from *Lycopersicon esculentum*(36) and XenA from *Pseudomonas putida*.^37^

**Scheme 3:**
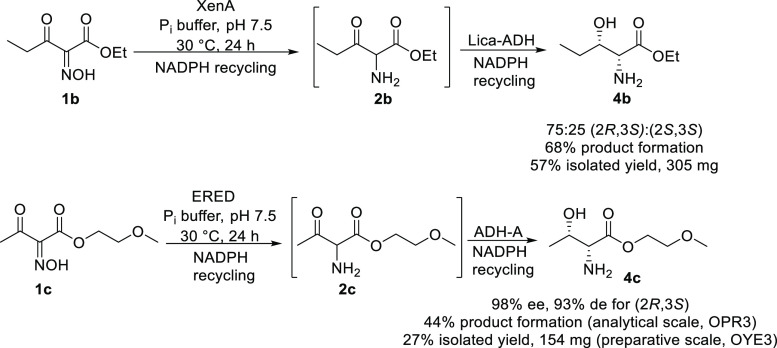
Two-Step Reduction of Oximes **1b** and **1c** to Get Indications for the Formation of the Amino Moiety The enzymes originate from *L. esculentum* (OPR3), *P. putida* (XenA), *Rhodococcus ruber* (ADH-A), and a variant of the ADH from *L. kefir* (*Lk*ADH-Lica). Reaction conditions: ene-reductase (4.89 μM for XenA and 4.46 μM for OPR3), 10 mM substrate, 0.5 mM NADPH, 0.5 mM reduced nicotinamide adenine dinucleotide (NADH) (only for reactions with ADH-A), 50 mM glucose, 4 mg/mL GDH, 5% dimethyl sulfoxide (DMSO) (v/v), 50 mM phosphate buffer, pH 7.5, 30 °C, 24 h, and 120 rpm. Total volume: 0.5 mL. Product formation is defined by the amount of substrate transformed to the amino alcohol (as the *N*-benzoyl derivative) as deduced from high-performance liquid chromatography (HPLC) analysis on a chiral stationary phase using calibration curves.

As the bi-enzymatic cascade developed previously suggested the formation of the amino functionality in **4a** for substrate **1a** only,^20^ amine formation for **1b,c** needs to be proven prior to using these substrates for crystallization experiments. Therefore, the amine forming cascade was tested for the other two substrates **1b** and **1c** (Scheme 3). For substrate **1b**, a *Lactobacillus kefir* variant^38^ named *Lk*ADH-Lica here proved to be most suitable, allowing the formation of two diastereomers of the amino alcohol **4b** with a ratio of 75:25 for (2*R*,3*S*)/(2*S*,3*S*) at 68% product formation (Scheme 3). For oxime substrate **1c**, ADH-A was found to be the best out of a small panel of ADHs tested (see the Supporting Information for details), although this enzyme reduced the ketone moiety in the oxime starting material to a small extent, affording also the corresponding β-hydroxy-α-oximo ester. However, this compound was shown not to be a substrate for the EREDs, which meant that the cascade proceeds *via* initial oxime reduction by the ERED, followed by ketone reduction (see the Supporting Information). The bi-enzymatic reduction cascade of oxime **1c** again resulted in the amino alcohol functionality with high ee (98%) and 93% de.

Although amine formation was clearly proven, the stereochemical outcome of the various transformations performed with different ADHs indicated that actually the ADH and not the ERED might be the enzyme controlling the stereochemical outcome at Cα (see the Supporting Information for details).^39,40^ Thus, when using the same ERED but varying the ADH, significantly different results were obtained for the optical purity at Cα, which should be controlled by the ERED only. If the ADH controls the final absolute configuration at both chiral centers, this indicates that compound **2** is chirally labile and prone to fast racemization. Using the chemically synthesized racemic hydrochloride salt of the amino compound **2a** for a biocatalytic reduction in the presence of ADH-A in a D_2_O-based buffer revealed complete incorporation of deuterium at Cα, indeed indicating rapid racemization of this intermediate (see the Supporting Information).

To gain more insight into the reaction pathway, we determined the crystal structures of the two enzymes OPR3 and XenA in complex with the oxime substrates **1a–c**, either by co-crystallization or by soaking. All the crystal structures obtained had a high resolution (1.9–1.35 Å) and showed clear electron density features for the oximes (see the Supporting Information for details). In general, XenA structures showed a better-defined electron density for the substrate when prepared by soaking, while for OPR3, clearer results were obtained by co-crystallization.

**Figure 1:**
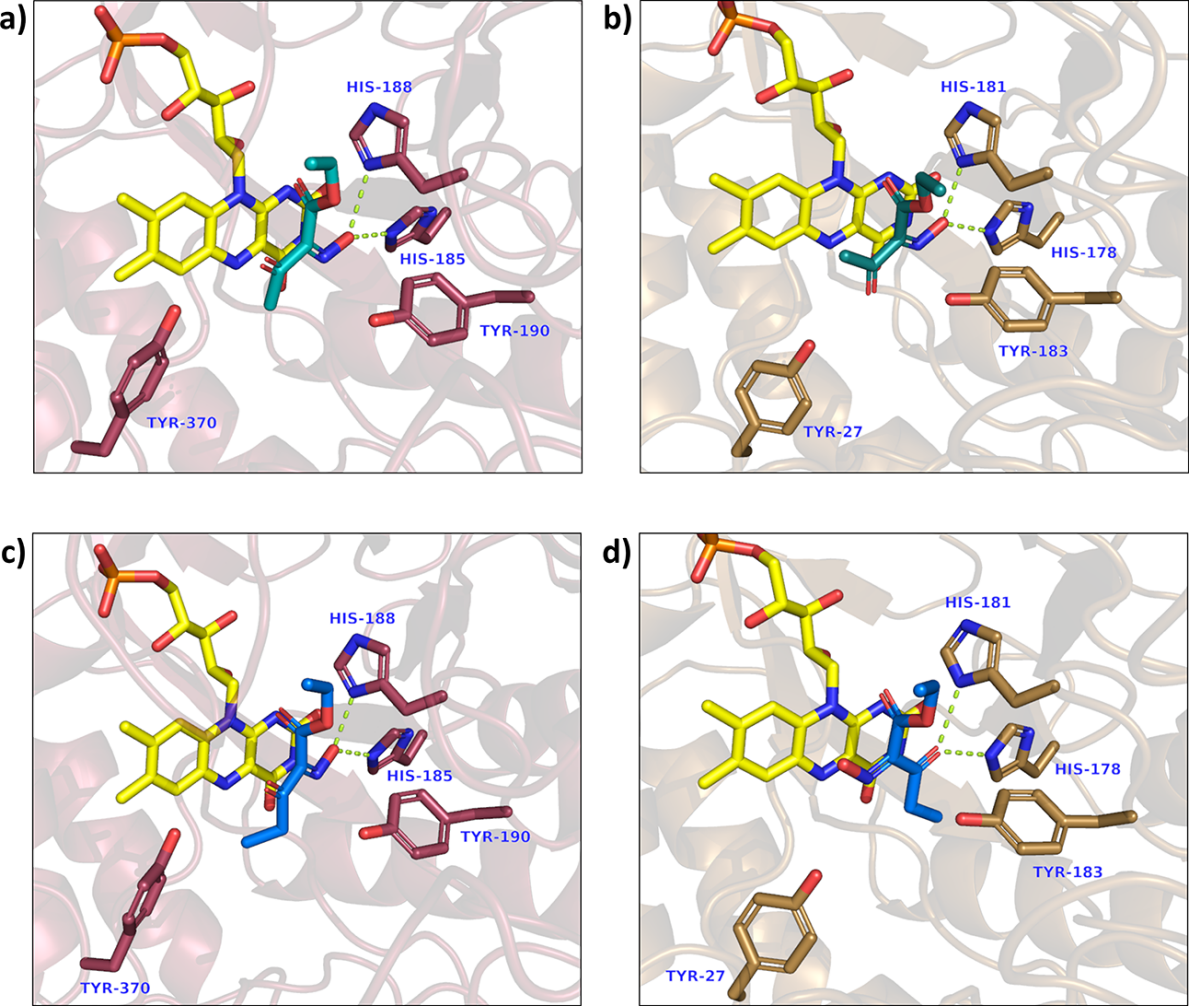
Crystal structures of OPR3/XenA in complex with substrates **1a** and **1b**. (a) OPR3/**1a**. (b) XenA/**1a**. (c) OPR3/**1b**. (d) XenA/**1b**.

As a representative selection of the structures obtained, Figure 1a–c shows OPR3/XenA with substrates **1a** and **1b** with similar binding modes. In these structures, the (possibly deprotonated) hydroxyl group of the oxime is hydrogen-bonded to the histidine residues that are usually involved in the interaction with the electron-withdrawing group attached to the commonly reduced C=C. Similar binding modes exhibiting a hydroxyl group interacting with the active site histidines have previously been observed in other OYE ligand complexes, e.g., for OPR3 in complex with *para*-hydroxybenzaldehyde and for XenA in complex with 8-hydroxycoumarin.^41−44^ According to the literature, these particular binding modes are probably favored by the oxidized, electron-deficient flavin, which alters the p*K*_a_ of the substrates by stabilizing the oximate anion.^45^ The oximes investigated here can indeed easily be deprotonated as indicated by the p*K*_a_ values of their oxime–OH groups. QM calculations estimated p*K*_a_ values of 7.4, 7.1, and 7.2 for oxime **1a**, **1b**, and **1c**, respectively. Comparable experimental p*K*_a_ values are reported for chemically similar oximes^46^ and for the 8-hydroxycoumarin and *para*-hydroxybenzaldehyde.^45,47−51^ Substrates of ene-reductases usually bind with the hydride-receiving atom within van der Waals distance from the N5 of the flavin and with an enzyme-dependent angle between the flavin N10–N5 axis and the atom receiving the hydride.^52^ In the cases mentioned above, none of these criteria are satisfied, and the binding modes very likely correspond to catalytically non-active complexes. Thus, we will refer to it as the “non-productive binding mode”.

However, the structure of XenA in complex with oxime **1b** resembles a possibly productive binding mode (Figure 1d), as the most electron-withdrawing group, the ketone, is hydrogen-bonded to the histidine residues, and the oxime moiety is placed at a suitable distance to the flavin N5. It is important to note that the binding mode of oxime **1b** in XenA places the C=N bond in a comparable position to the C=C double bond of activated alkenes, the typical substrates for these enzymes. As the hydride is known to be transferred to the β-carbon when using activated alkenes as substrates, we hypothesized that the hydride in the first step of oxime reduction is transferred to the nitrogen of the oxime moiety for substrate **1b**. Both the hydroxyl group of the oxime and the β-keto ester motif induce a positive polarity at the nitrogen suitable for hydride transfer. Nevertheless, a closer comparison with a previously deposited structure of a XenA–coumarin complex (Figure S7) shows that the C=N bond of the oxime is still not in an ideal position for hydride transfer. Especially, the angle defined by the N10–N5 axis and the oxime N-atom is too narrow. This indicates that the binding mode of oxime **1b** observed in the crystal structure is closer to a productive binding mode but still not perfectly suited for oxime reduction.^52^ Nevertheless, molecular dynamics (MD) simulations starting from this structure (see the Supporting Information) revealed suitable productive near-attack conformations (NACs) which are in close agreement with the described features for ERED substrates.^45,53,54^ NACs represent the subpopulations of the substrate approaching the configuration of the relevant transition state.

**Figure 2:**
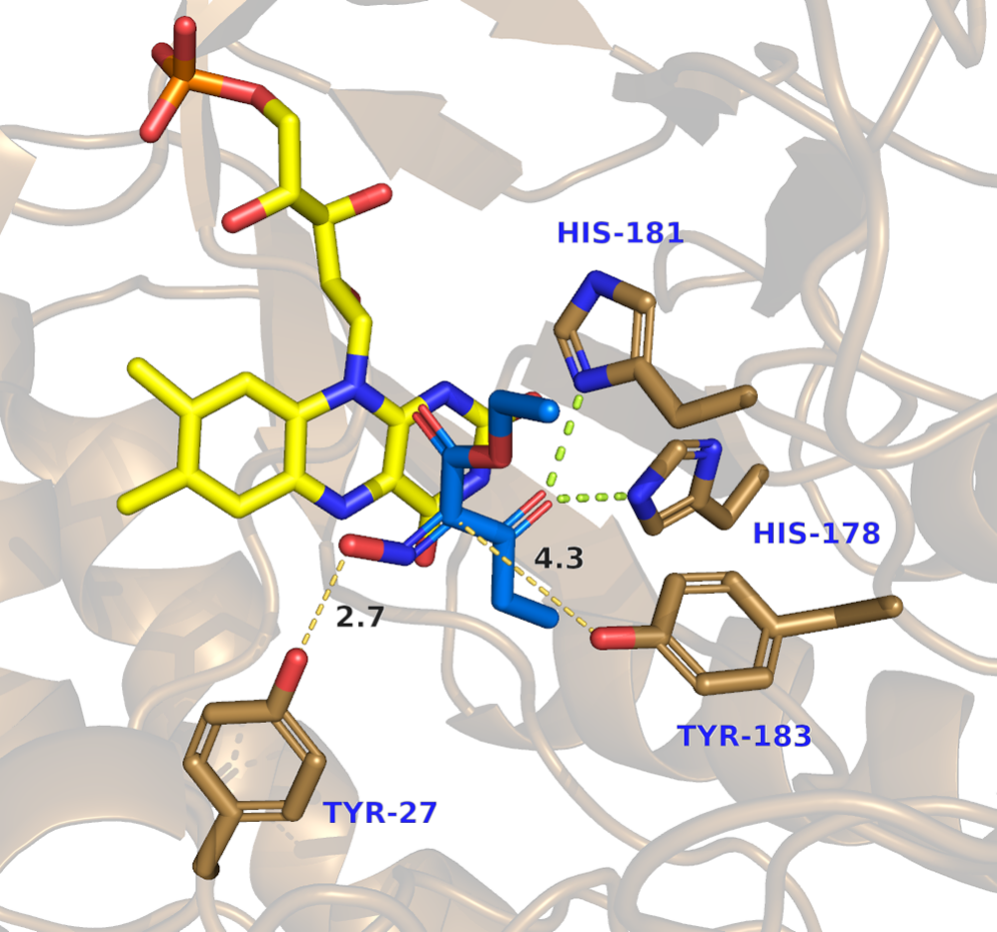
Binding of oxime **1b** in the active site of XenA obtained from MD simulation.

**Figure 3:**
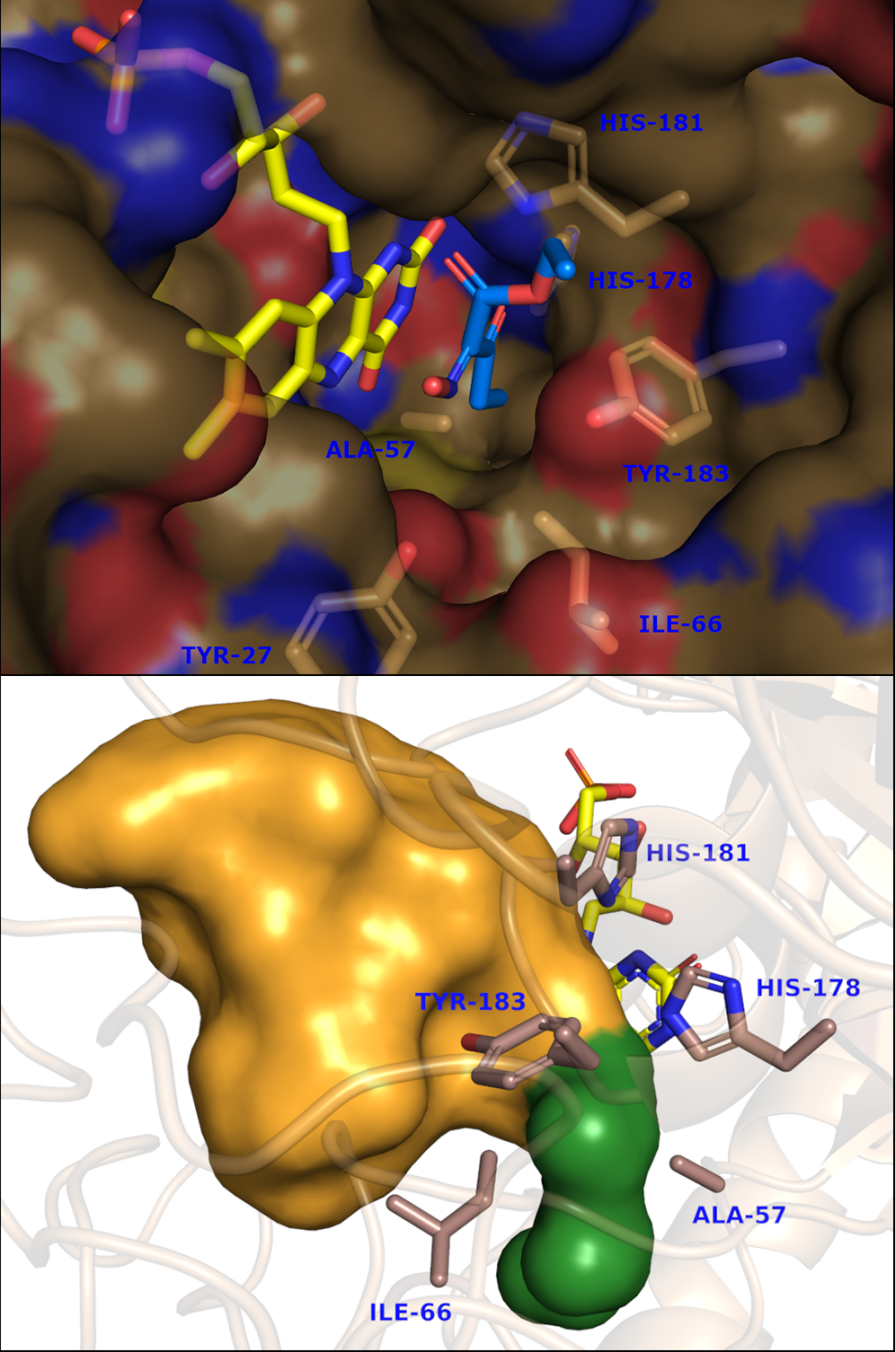
Pocket in the active site of XenA in the surface mode, harboring the propanoyl moiety of oxime **1b** (top) and the active site cavity calculated using the HOLLOW program (bottom). The pocket bearing the propanoyl moiety is highlighted in green for clarity.

**Scheme 4:**
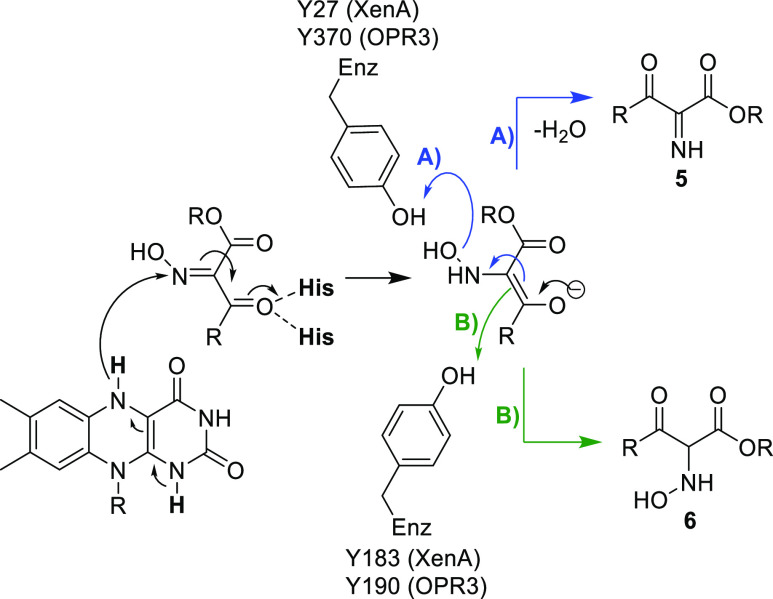
Possible Pathways Involving the Canonical and Non-canonical Tyrosine in Oxime Reduction

Figure 2 shows a snapshot of one of the NACs of XenA and oxime **1b**. In this binding mode, the hydroxyl group of the oxime is at a hydrogen bond distance (2.7 Å) from tyrosine Y27. This would not only stabilize the binding of the oxime, but Y27 could also act as a proton donor after hydride transfer, enabling a subsequent dehydration reaction and imine formation (Scheme 4, pathway A). Comparison with the other previously investigated enzymes^20^ revealed that this tyrosine is structurally conserved, though in class I/II OYEs, it is located close to the C-terminus of the enzyme (Y370 in OPR3). It must be emphasized that such a residue (like Y27 in XenA) has never been proposed to be involved in a reaction mechanism of these enzymes. Previous experiments with XenA Y27F showed no involvement in the oxidative half-reaction.^55^ Tyrosine 183, the canonical proton donor of XenA,^55^ is also found at a hydrogen bond distance from the Cα of the oxime. Hydride transfer to the oxime nitrogen followed by proton transfer from the canonical tyrosine would lead to the formation of the hydroxylamine (Scheme 4, pathway B). Consequently, both mechanisms are feasible. To understand the putative catalytic activity of these tyrosine residues for oxime reduction and to obtain hints about the identity of the intermediate, the XenA variants Y27F and Y183F, as well as the corresponding OPR3 variants Y370F and Y190F, were produced, tested in biotransformations, and structurally and kinetically characterized (see the next page after Figure 3).

As an additional aspect of the crystallographic study, the structure of XenA with **1b** (Figure 1d) might explain why only XenA efficiently transformed substrate **1b**, and OPR3 did not. Binding pose metadynamics performed on both productive and non-productive binding modes (non-productive modeled from the crystal structure of XenA_Y183F, *vide infra*) showed that the productive binding mode appeared to have higher stability than the non-productive binding mode (Figure S11). In fact, it presents a lower root-mean-square deviation (RMSD) than the non-productive binding pose, which shows a steep RMSD increase over time, indicating poor binding of the substrate in the active site.

A closer look to the active site of XenA revealed a pocket delimited by the residues A57, I66, H178, H181, and Y183 (Figure 2), which is not present in other previously investigated enzymes.^20^ The presence of this pocket was supported by analysis with the computer program HOLLOW.^56^ The propanoyl moiety of oxime **1b** points into this cavity and is likely stabilized by hydrophobic interactions (Figure 3). Substrates with an acetyl moiety (such as oximes **1a** and **1c**) give the non-productive binding mode described above. Therefore, the presence of this pocket in XenA might explain why it is the only enzyme able to reduce oxime **1b** efficiently and why oximes with substituents larger than an ethyl group are not accepted as substrates.^20^

### Tyrosine Variants in Biotransformations

Testing the tyrosine variants for oxime reduction showed that the exchange of the non-canonical tyrosine Y370 to phenylalanine in OPR3 led to significantly decreased product formation compared to the wild type (Table 1, OPR3_Y370F). This is true for the transformation leading to pyrazine **3a** and the cascade with the alcohol dehydrogenase ADH-A leading to the amino alcohol **4a**. This indicates that the non-canonical tyrosine Y370 is indeed important for fast oxime reduction. Interestingly, the exchange of the canonical tyrosine (Y190F) did not have an observable effect in this experiment, indicating that fast protonation in the active site is feasible, likely *via* a water molecule. Looking at the results obtained for XenA, product formation was comparable for both tyrosine variants, the canonical Y183F and the non-canonical one (Y27F). Thus, the involvement of the non-canonical tyrosine is not observed here. For a more detailed analysis, stopped-flow pre-steady state kinetics were performed.

**Table 1:** Biotransformations of Oximes **1a** and **1b** with Tyrosine Variants in Comparison to the Wild Types Leading Either to the Pyrazine **3** (Scheme 1) or to the Amino Alcohol **4** (Schemes 1 and 2)

enzyme	substrate	pyrazine **3** (%)^a^^,^^b^	amino alcohol **4** (%)^d^^,^^e^
OPR3_WT	**1a**	43	38
OPR3_Y190F	**1a**	57	44
OPR3_Y370F	**1a**	14^c^	18
XenA_WT	**1b**	38	68
XenA_Y183F	**1b**	37	61
XenA_Y27F	**1b**	33	72

a Reaction conditions: ene-reductase (4.89 μM for XenA and 4.60 μM for OPR3), 10 mM substrate, 0.5 mM NADPH, 50 mM glucose, 4 mg/mL GDH, 5% DMSO (v/v), 50 mM phosphate buffer, pH 7.5, 30 °C, 24 h, and 120 rpm.

b Product formation determined by GC-FID using calibration curves.

c Using 9.2 μM enzyme.

d Reaction conditions: ene-reductase (4.89 μM for XenA and 4.60 μM for OPR3), 10 mM substrate, 0.5 mM NADPH, 0.5 mM NADH (for substrate **1a**), 2 mg/mL ADH-A (partially purified CFE, for substrate **1a**), 5 mg/mL *Lk*ADH-Lica (crude CFE, for substrate **1b**), 50 mM glucose, 4 mg/mL GDH (crude CFE), 5% DMSO (v/v), 50 mM phosphate buffer, pH 7.5, 30 °C, 24 h, and 120 rpm. Total volume: 0.5 mL.

e Product formation determined by HPLC analysis on a chiral stationary phase using calibration curves.

### Stopped Flow Experiments

**Figure 4:**
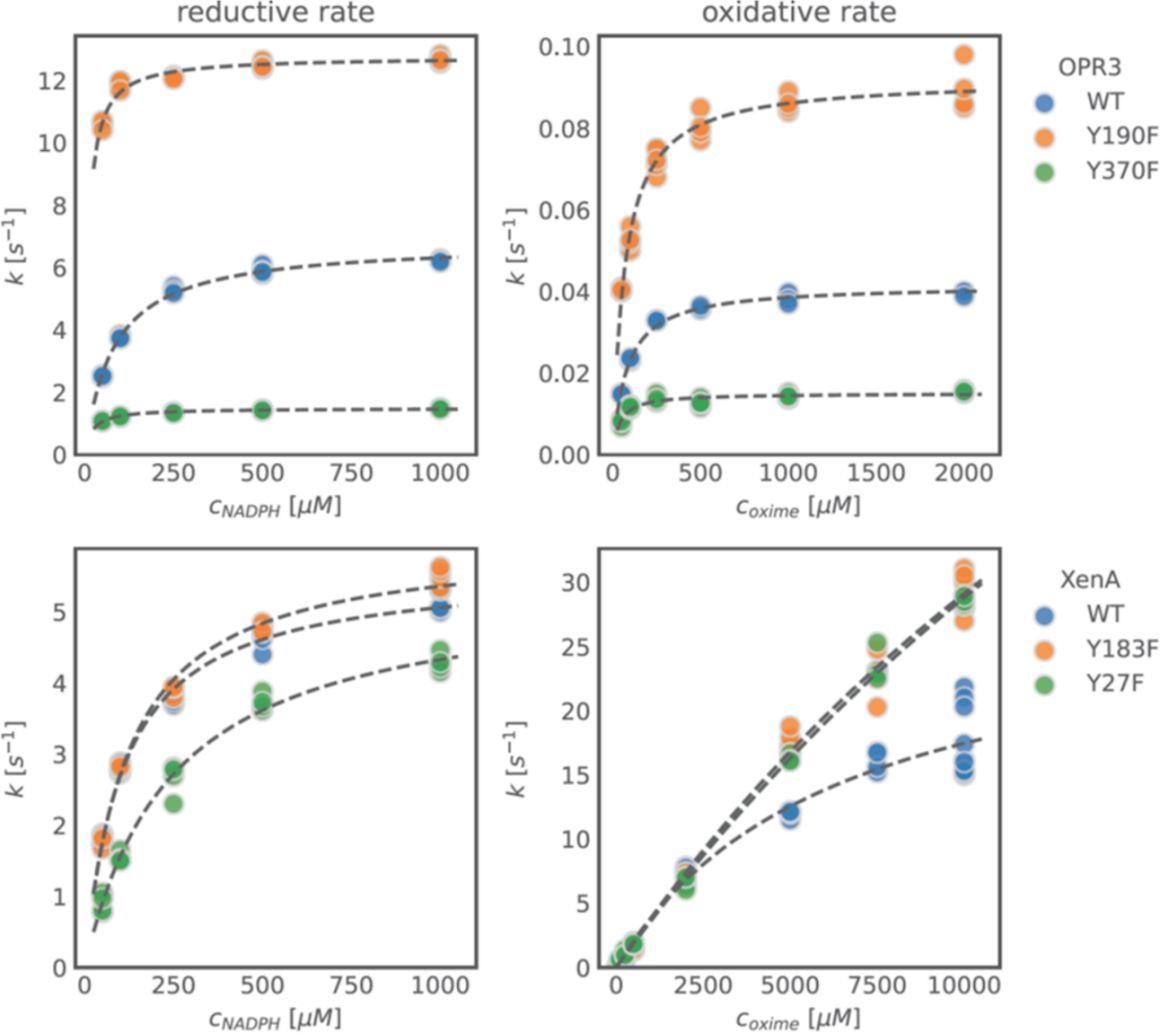
Stopped-flow pre-steady-state kinetics. Reductive (left) and oxidative (right) half-reaction of OPR3 (top)/XenA (bottom) and their variants.

OPR3 and its variants showed a typical Michaelis–Menten behavior for both the reductive and oxidative half-reactions (Figure 4, top row). In comparison to the wild type, the non-canonical tyrosine variant Y370F of OPR3 showed a 4-fold decrease in both *k*_red_ and *K*_d_ in the reductive half-reaction and a 3- and 2-fold decrease in *k*_ox_ and *K*_d_ in the oxidative half-reaction (Table 2). It is clear that Y370 plays some role in the binding and in the reactivity with reduced nicotinamide adenine dinucleotide phosphate (NADPH) as well as with the oxime. The effect of the Y370F mutation on the oxidative half-reaction is a clear indication that this residue has a role in the catalytic mechanism. This was also supported by the lower product formation in the biotransformation (Table 1). On the other hand, the canonical tyrosine variant OPR3_Y190F showed a 2-fold increase in *k*_red_ and an 8-fold decrease for the NADPH *K*_d_. Though a parallel 2-fold increase in *k*_ox_ is also observed, the *K*_d_ for the oxime seems not to be affected by the mutation (Table 2), indicating that the canonical tyrosine residue does not play a role in substrate binding in this enzyme.

**Table 2:** Kinetic Parameters of the Reductive and Oxidative Half-Reactions of XenA, OPR3, and Tyrosine Variants

enzyme	*K*_d_ NADPH (μM)	*k*_red_ (s^–1^)	*K*_d_ oxime (μM)	*k*_ox_ (s^–1^)
OPR3_WT	80.6 ± 2.4	6.82 ± 0.05	79 ± 4^b^	0.042 ± 0.001^b^
OPR3_Y190F	9.8 ± 0.6	12.76 ± 0.06	69 ± 4^b^	0.092 ± 0.001^b^
OPR3_Y370F	20.0 ± 0.9	1.49 ± 0.01	37.4 ± 4.4^b^	0.015 ± 0.000^b^
XenA_WT^c^	108.5 ± 5.1	5.61 ± 0.07	6434 ± 1571^a^	28.7 ± 3.2^a^
XenA_Y183F	121.2 ± 5.2	6.01 ± 0.08	n.a.	n.a.
XenA_Y27F	245 ± 15	5.39 ± 0.12	n.a.	n.a.

a For oxime **1b**.

b For oxime **1a**.

c The kinetic data for the reductive half-reaction differ from the previously reported ones^55,57^ most likely due to different assay conditions as well as a different positions of the His-tag. n.a. not applicable due to the non-Michaelis Menten behavior.

Wild-type XenA showed a typical Michaelis–Menten behavior for the oxidative half-reaction with the oxime **1b** (Figure 4, top right). The calculated high *K*_d_ for oxime **1b** (Table 2) clearly indicates this enzyme’s low affinity for the substrate and might explain why this enzyme performed poorly in the biotransformation of the other oximes in our previous work.^20^ Both the Y183F and the Y27F variants of XenA showed a non-Michaelis–Menten behavior, with a linear increase of the rate over the concentration (Figure 4, bottom right). This may indicate that both tyrosines might have a role in the binding of the oxime.

### Crystal Structures of the Tyrosine Variants

To assess if the introduced amino acid exchanges affected the overall structure and the binding of the oxime, we also crystallized the tyrosine variants in complex with selected oxime substrates (Table S12). The protomers in the crystal structures of the tyrosine variants are perfectly superimposable with the ones of the wild-type enzymes, confirming that the exchanges did not lead to major structural alterations. Interestingly, the binding mode of the oxime substrates also did not change in most of the structures. The only exception was found for XenA Y183F in complex with oxime **1b** (Figure S6b): while the electron density of the substrate was clear in the wild-type enzyme—and only the productive binding mode could be modeled in it—the density for the oxime appears less defined in the Y183F variant, and both the productive and the non-productive binding mode could be fit in the density.

**Scheme 5:**
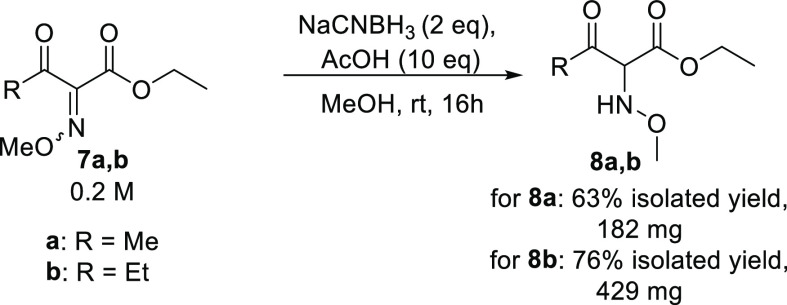
Synthesis of Hydroxylamine **8**

The insights gained from the binding modes obtained by crystallization and MD simulation, as well as the indicated role of Y370 in OPR3 for protonation of the OH group of the oxime, indicated that hydride transfer to the oxime nitrogen followed by expulsion of the protonated oxime OH might be the most likely mechanism for the initial reduction (Scheme 4A). This pathway would lead to the imine intermediate **5** and not the hydroxylamine moiety in **6**. As previous attempts of trapping or detecting this imine intermediate failed,^20^ and the synthesis is not feasible,^58^ the synthesis of the hydroxylamine **6** was envisioned in order to test whether or not **6** is a substrate. Although all synthetic attempts to prepare hydroxylamine **6** were unsuccessful, the reduction of the corresponding oxime methyl ether **7** using sodium cyanoborohydride gave *O*-methyl hydroxylamine **8** in 63–76% isolated yield, and good purity after column chromatography (Scheme 5). As oxime ether **7** has not yet been described to be a substrate for EREDs, the reduction of **7a** and **7b** by OPR3 and XenA was evaluated.

**Scheme 6:**
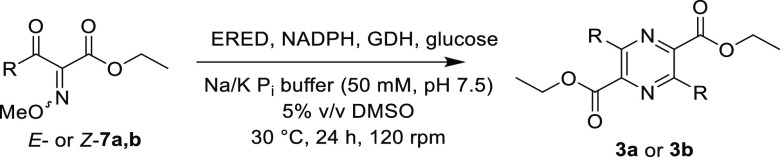
Pyrazine Formation of Oxime Methyl Ethers Catalyzed by EREDs

The oxime ether **7** is *E*/*Z*-configurationally stable, unlike the free OH oximes, which are expected to readily undergo *E*/*Z* interconversion in polar and/or protic solvents.^59−61^ The *E*/*Z* isomers of **7** were separated by silica gel chromatography and tested as individual isomers (Scheme 6 and Table 3). Interestingly, oxime ethers *E*- and *Z*-**7a** as well as *Z*-**7b** were transformed by the EREDs tested, leading to the same final products **3a** and **3b** as obtained in the reduction of **1a** and **1b**. Thus, the methyl ether moiety is expelled during reduction. Consequently, the compound formed before spontaneous dimerization must be again intermediate **2** (Scheme 1). An additional interesting observation was that the enzymes tested displayed an *E*/*Z*-preference. This preference is most pronounced for the methyl ether **7b**, of which only the *Z*-isomer *Z*-**7b** was converted to the pyrazine by XenA (Table 3, entries 5–6).

**Table 3:** Biotransformations with Oxime Methyl Ethers **7a,b**^a^

entry	substrate	ERED^b^	pyrazine (%)^c^^,^^d^
1	*Z*-**7a**	OPR3	50
2	*E*-**7a**	OPR3	56
3	*Z*-**7a**	XenA	12
4	*E*-**7a**	XenA	26
5	*Z*-**7b**	XenA	14
6	*E*-**7b**	XenA	n.d.

a Determined based on the chemical shift of the ketone carbon as described in the literature.^61^

b The enzymes originate from: *Saccharomyces cerevisiae* (OYE3),^62,63^*L. esculentum* (OPR3),^36^ and *P. putida* (XenA).^37^

c Reaction conditions: ene-reductase (200 μg/mL and 4.45 μM for OYE3, 4.89 μM for XenA, and 4.60 μM for OPR3), 10 mM substrate, 0.5 mM NADPH, 50 mM glucose, 4 mg/mL GDH, 5% DMSO (v/v), 50 mM phosphate buffer, pH 7.5, 30 °C, 24 h, and 120 rpm. Total volume: 0.5 mL.

d Determined by GC using calibration curves. n.d.: not detected.

**Scheme 7:**
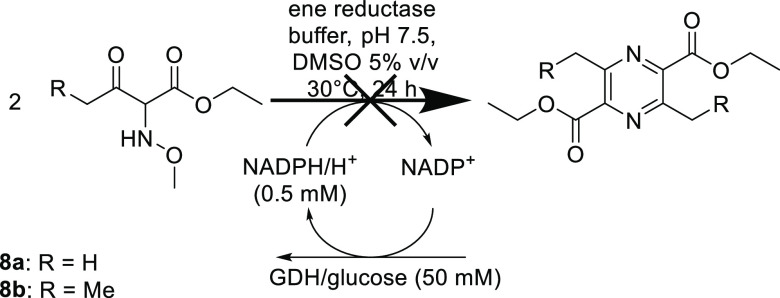
Unsuccessful Biotransformations of *O*-Methyl Hydroxylamine **8** to Pyrazine **2** Reaction conditions: ene-reductase (200 μg/mL, 4 μM), 10 mM substrate, 0.5 mM NADPH, 50 mM glucose, 4 mg/mL GDH, 5% DMSO (v/v), 50 mM phosphate buffer, pH 7.5, 30 °C, 24 h, and 120 rpm. Total volume: 0.5 mL.

If the reduction of **7** followed by dimerization to **3** would proceed *via* a hydroxylamine intermediate **8**, then **8** would also be a substrate for the ene-reductases. However, testing **8** as a substrate under otherwise identical reaction conditions (Scheme 7) led to no detectable pyrazine **3** formation. Additionally, it has to be mentioned that no hydroxylamine **8** was detected during the biotransformation of **7**. Consequently, all experiments indicate that imine **5** is the intermediate, rather than the hydroxylamine **6**.

Finally, the second reduction step (imine **5** to amine **2**) very likely also proceeds *via* hydride transfer to the nitrogen, following the common reactivity of ene-reductases. MD simulations using imine **5a** (modeled from the complex of oxime **1b** in XenA) show a considerable fraction of NACs, thus suggesting that the proposed mechanism is plausible (Figure S12).

## Conclusions

**Scheme 8:**
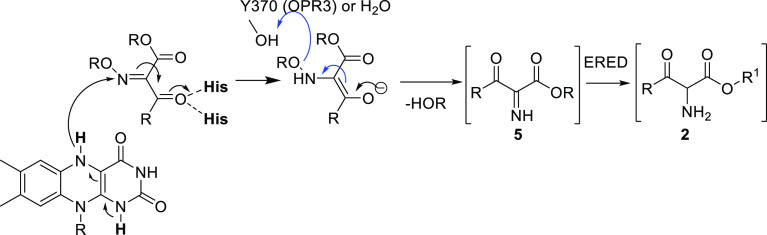
Summary of the Mechanism of ERED-Catalyzed Reduction of α-Oximo-β-keto Esters

Analyzing the crystal structures of selected ene-reductases with oxime substrates bound to the active site, a mechanism for oxime reduction for substrates **1** and **7** was proposed (Scheme 8). Thereby, the hydride from the reduced flavin is transferred to the oxime nitrogen. For OPR3, a non-canonical tyrosine residue was suggested as potentially catalytically relevant for the transformation of oxime substrates. The non-canonical tyrosine residue Y370 was shown to contribute to the reduction of the oxime moiety most likely *via* protonation of the hydroxy/methoxy moiety, leading to the imine intermediate. As this newly found non-canonical tyrosine accelerated the reaction of the transformation, this insight will give guidance for future designs of enzymes for oxime reduction. Showing that hydroxylamine ethers are non-substrates supported the hypothesis that the oxime reduction of the investigated α-oximo-β-keto esters proceeds *via* the imine intermediate.

The study opens an avenue to develop catalysts (organocatalysts/biocatalysts) for the asymmetric reduction of oximes and may open a new pathway to access optically pure amines.

## Supporting Information Available

Supplementary experiments, details on crystallization, MD, synthesis of substrates and references, analytics, and NMRs (PDF)
