# Supplementary material for: Mechanistic Insights into the Ene-Reductase-Catalyzed Promiscuous Reduction of Oximes to Amines
Source: ACS Catal. 2023 Feb 6;13(4):2610–8. doi: 10.1021/acscatal.2c06137 (PMC9942197; doi:10.1021/acscatal.2c06137)
Supplement: Supplementary file 1 — cs2c06137_si_001.pdf [file cs2c06137_si_001.pdf]

Supporting Information

Mechanistic insights into the ene-reductase catalyzed promiscuous reduction of oximes to amines

Willem B. Breukelaar‡,[a], Nakia Polidori‡,[b], Amit Singh[b], Bastian Daniel[b], Silvia M. Glueck[a], Karl Gruber[b,c,d]*, Wolfgang Kroutil[a,c,d]*

[a] Department of Chemistry, University of Graz, NAWI Graz, Heinrichstraße 28, 8010 Graz, Austria.

[b] Institute of Molecular Biosciences, University of Graz, Humboldtstraße 50, 8010 Graz, Austria.

[c] Field of Excellence BioHealth, University of Graz, 8010 Graz, Austria.

[d] BioTechMed Graz, 8010 Graz, Austria.

*Corresponding authors. E-mails: Karl.Gruber@uni-graz.at, Wolfgang.Kroutil@uni-graz.at

Contents

General.......... 3
Supplementary experiments.......... 4
Optimization of the reaction conditions for the cascade biotransformations on oxime **1b**.......... 4
Optimization of the reaction conditions for the cascade biotransformations on oxime **1c**.......... 6
ADH-catalyzed reductions of aminoketones.......... 8
Biocatalysis with tyrosine variants.......... 10
Production of enzymes.......... 11
Wild-type ene-reductases.......... 11
Ene-reductase variants.......... 13
Alcohol dehydrogenases.......... 15
Protein crystallization.......... 18
Data collection and structure solution.......... 22
Structures of wild-type enzymes and variants.......... 30
Comparison with previously deposited structures.......... 33
Molecular dynamics simulations.......... 35
NAC-MD Simulations.......... 35
Binding Pose Metadynamics (BPMD).......... 37
Biocatalysis.......... 39
Analytical scale biotransformations.......... 39
Preparative scale biotransformations.......... 42
Stop-flow experiments.......... 45
Synthesis of substrates.......... 46
Synthesis of reference compounds.......... 51
Racemic reference products for cascade biotransformations on oxime **1b**.......... 51
Synthesis of enantioenriched ethyl 2-benzamido-3-hydroxypentanoate.......... 54
Reference products for cascade biotransformations on oxime **1c**.......... 60
Analytics.......... 62
GC-FID, achiral stationary phase.......... 62
HPLC-UV, chiral stationary phase.......... 64
Reversed-phase HPLC-UV (achiral stationary phase, qualitative).......... 77
HPLC-MS.......... 90
References.......... 99
Appendix A: NMR spectra.......... 101

General

NMR spectra were recorded on a 300 MHz instrument using the residual solvent signal as reference, with chemical shift reported in ppm, coupling constants (J) in Hz. TLC analysis was done on aluminium backed silica gel 60 F_{254} sheets and compounds were visualized by UV (254 nm) or by dipping the plate into a staining agent (basic permanganate, cerium ammonium molybdate or ninhydrin solution) and heating when required. Silica gel chromatography was either performed on an automated system using an amount of silica gel approximately 10-20 times the mass of the crude product an eluted with a gradient of ethyl acetate in cyclohexane or done manually (column diameter chosen based on mass and solubility of the crude product, height approximately 17 cm), eluting with gravity or pressurized air. Reagents were purchased from Sigma-Aldrich, Merck, Fluorochem, Fisher Scientific, Alfa Aesar, Acros organics, TCI and ABCR and used as received. Anhydrous solvents were purchased over molecular sieves from Acros Organics and Fisher Scientific and used as received. Unless stated otherwise, “water” refers to demineralised water, “buffer” refers to 50 mM Na/K P_i buffer, pH 7.5 (prepared by dissolving 7.58 g $Na_2HPO_4 \cdot 2H_2O$ and 1.01 g KH_2PO_4 in 1 L of water, no pH adjustment required). Analytical scale biotransformations were performed in 1.5 mL microcentrifuge tubes in temperature-controlled incubation chambers. Glucose dehydrogenase (GDH) was obtained as a crude cell-free extract from DSM as GDH.001 ($NADP^+$: 12 U/mg, NAD^+; 7 U/mg) and used as received.

Supplementary experiments

Optimization of the reaction conditions for the cascade biotransformations on oxime 1b

We started our investigations by screening five alcohol dehydrogenases in combination with XenA (Scheme S1, Table S1).

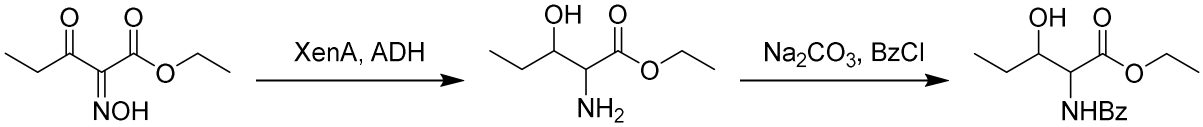

Scheme S1: Cascade reaction with oxime ***1b***

Table S1: Results from ADH cascade screening.

ADH	ADH concentration (mg/mL)	Aminoalcohol formation (%)	Diastereomeric excess (%)
ADH-A	40	8.9	0
*Lk*ADH	40	n.d.	n/a
*Lb*ADH	40	n.d.	n/a
*Lk*ADH-Lica	8	41.5	52
*Sy*ADH	8	n.d.	n/a

Conditions: oxime **1b** (10 mM), XenA (4.89 µM), NADPH (0.5 mM), NADH (0.5 mM), glucose (50 mM), GDH (4 mg/mL) ADH (crude CFE, 40 mg/mL for ADH-A, *Lk*ADH and *Lb*ADH, 8 mg/mL for Lk*ADH-Lica* and *Sy*ADH). Buffer: 50 mM Pi, pH 7.5, 5% DMSO v/v. 120 rpm, 24 h, 30 °C. Total volume: 0.5 mL. n.d.: not detected. Product formation is defined by the amount of substrate transformed to the aminoalcohol (as the *N*-benzoyl derivative) as deduced from HPLC analysis on a chiral stationary phase using calibration curves.

Next, we investigated whether the ADHs reduce the ketone moiety of the oxime to form an α-oximo-ß-hydroxy ester. Fortunately, none of the investigated enzymes (ADH-A, *Lb*ADH, *Lk*ADH, *Lk*ADH-Lica and *Sy*ADH) reduced oxime **1b**.

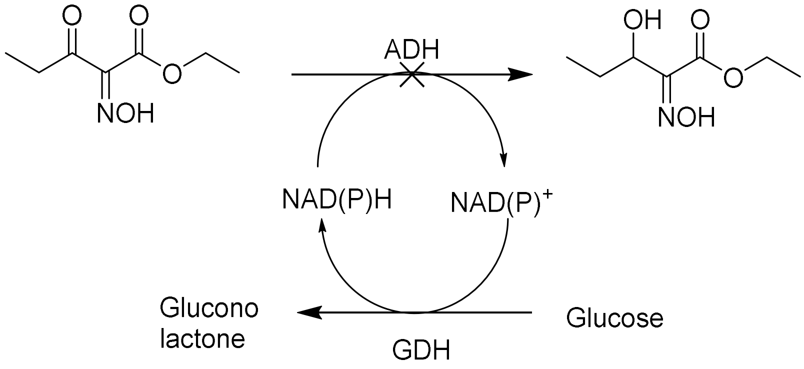

Scheme S2: Control experiment with oxime ***1b*** *and ADHs. Reaction conditions: oxime (10 mM), ADH (crude CFE, 1 mg/mL), NADH (0.5 mM), NADPH (0.5 mM) glucose (50 mM), GDH (crude CFE, 4 mg/mL). Buffer: 50 mM Na/K Pi, pH 7.5, 5% DMSO v/v. 120 rpm, 24h, 30 °C. Total volume: 0.5 mL.*

Having identified ADH-A and *Lk*ADH-Lica as suitable candidates, we investigated the optimal ADH concentrations (Scheme S1, Table S2) for both product formation and stereochemical outcome.

Table S2: ADH concentration screening for cascade reactions.

ADH	ADH concentration (mg/mL)	Aminoalcohol formation (%)	A/B/C/D*
ADH-A	2	n.d.	
	5	6.5	42:58:0:0
	10	10.6	50:50:0:0
	20	19.4	63:33:0:4
	40	13.2	54:22:0:24
Lica-ADH	1	6.5	27:0:0:73
	2	51.6	25:0:0:75
	**5**	**67.9**	**25:0:0:75**
	10	38.1	28:0:0:72
	20	41.1	27:0:0:73

Conditions: oxime **1b** (10 mM), XenA (0.2 mg/mL), NADPH (0.5 mM), NADH (0.5 mM), glucose (50 mM), GDH (4 mg/mL), ADH (CFE, see table). Buffer: 50 mM Pi, pH 7.5, 5% DMSO v/v. 120 rpm, 24 h, 30 °C. Total volume: 0.5 mL. A: 2*S*,3*S*, B: 2*R*,3*R*, C: 2*S*,3*R*, D: 2*R*,3*S*. n.d.: not detected. Product formation is defined by the amount of substrate transformed to the aminoalcohol (as the *N*-benzoyl derivative) as deduced from HPLC analysis on a chiral stationary phase using calibration curves.

While, unexpectedly, product formations of up to 19.4% could be obtained with ADH-A, *Lk*ADH-Lica was identified as the best ADH in terms of both product formation and stereochemical outcome.

In an attempt to simplify the reaction procedure, we also conducted the cascade biotransformation on oxime **1b** using 5% v/v 2-PrOH, serving both as cosolvent and as hydride source (Scheme S3, Table S3). Although product formations of up to 53% were obtained under the conditions tested, significantly increased amounts of the ADH would be required to get product formations similar to those obtained with the glucose/GDH recycling system. Therefore, glucose/GDH recycling was used for further cascade experiments.

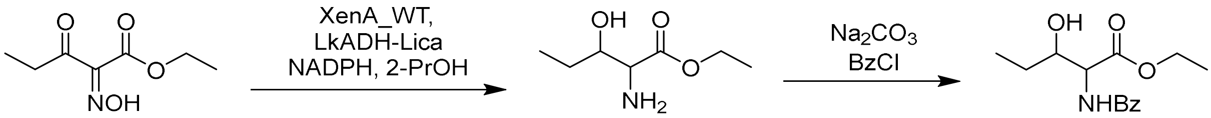

*Scheme S3: Cascade with oxime **1b** using* Lk*ADH-Lica for cofactor recycling*

*Table S3: Results from cascade with oxime **1b** using* Lk*ADH-Lica for cofactor recycling.*

Entry	*Lk*ADH-Lica *concentration (mg/mL)*	*Product formation (%)*
1	0.5	0
2	1	0
3	2	<5
4	5	36
5	10	53

Reaction conditions: oxime (10 mM), XenA-WT (purified, 4.89 µM), Lica-ADH (crude CFE), NADPH (0.5 mM), Buffer: 50 mM Na/K Pi, pH 7.5, 5% 2-PrOH v/v. 120 rpm, 24h, 30 °C. Total volume: 0.5 mL. Product formation is defined by the amount of substrate transformed to the aminoalcohol (as the *N*-benzoyl derivative) as deduced from HPLC analysis on a chiral stationary phase using calibration curves.

Optimization of the reaction conditions for the cascade biotransformations on oxime 1c

We started our investigations by testing a small panel of alcohol dehydrogenases, using OYE3 as ERED, as this enzyme gave good results for pyrazine formation from substrate **1c**.[1] The reaction equation (derivatization not shown) and results can be found in Scheme S4 and Table S4, respectively.

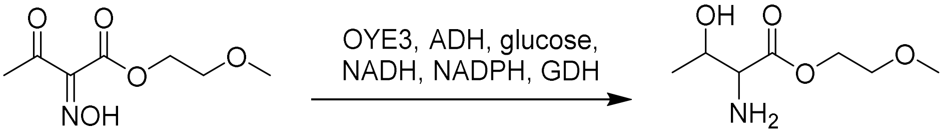

*Scheme S4: Cascade reaction with OYE3, oxime **1c** and various ADHs*

*Table S4: Results from ADH screening for cascade **1c**.*

Entry	*ADH*	*Product formation (%)*	*Stereochemistry*
1	ADH-A	24	98:2 D/D-allo
2	*Lb*ADH	1	>99% L-enantiomer
3	*Lk*ADH	4	60:40 D-allo/L
4	*Lk*ADH-Lica	17	50:50 D-allo/L
5	*Sy*ADH	0	n/a
6	*Ras*ADH	4	75:25 D/D-allo

Reaction conditions: oxime (10 mM), OYE3 (purified, 4.45 µM), (ADH, crude CFE, 2mg/mL for ADH-A, 5 mg/mL for the others), NADH (0.5 mM), NADPH (0.5 mM), glucose (50 mM), GDH (crude CFE, 4 mg/mL). Buffer: 50 mM Na/K Pi, pH 7.5, 5% DMSO v/v. 120 rpm, 24h, 30 °C. Total volume: 0.5 mL. Product formation is defined by the amount of substrate transformed to the aminoalcohol (as the *N*-benzoyl derivative) as deduced from HPLC analysis on a chiral stationary phase using calibration curves.

Next, we investigated whether the ADHs also acted on the ketone group of the oxime, giving α-oximo-ß-hydroxy ester **S1** (as shown in Scheme S5 for ADH-A). Out of the six ADHs tested, only *Lb*ADH and *Lk*ADH did not give this product.

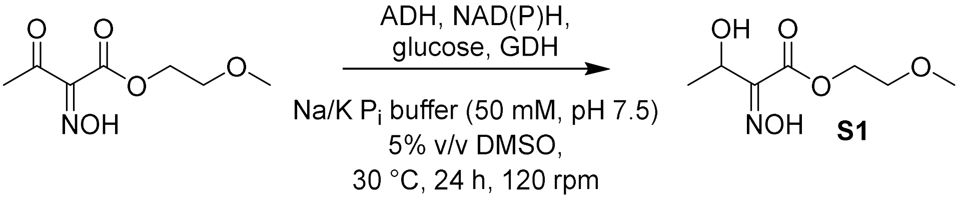

*Scheme S5: Control experiments for the cascade biotransformations with oxime **1c**. Reaction conditions: oxime (10 mM), ADH (crude CFE, 2mg/mL for ADH-A, 5 mg/mL for the others), NADH (0.5 mM), NADPH (0.5 mM) glucose (50 mM), GDH (crude CFE, 4 mg/mL). Buffer: 50 mM Na/K Pi, pH 7.5, 5% DMSO v/v. 120 rpm, 24h, 30 °C. Total volume: 0.5 mL.*

If α-oximo-ß-hydroxy ester **S1** is a substrate for the EREDs, this might mean that the cascade reaction can start with either ADH-catalyzed or ERED-catalyzed reduction. However, a biotransformation with this α-oximo-ß-hydroxy ester as substrate under standard reaction conditions with OYE3 as ERED showed no aminoalcohol formation.

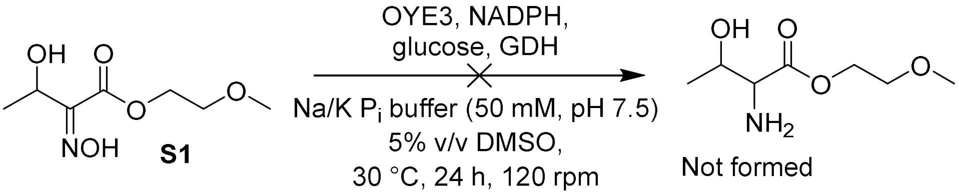

Scheme S6: Attempted ERED-catalyzed reduction of the α-oximo-ß-hydroxy ester. Reaction conditions: oxime (10 mM), OYE3 (purified, 4.45 µM), NADPH (0.5 mM) glucose (50 mM), GDH (crude CFE, 4 mg/mL). Buffer: 50 mM Na/K Pi, pH 7.5, 5% DMSO v/v. 120 rpm, 24h, 30 °C. Total volume: 0.5 mL.

Having thus established that the cascade proceeds *via* initial oxime reduction to an aminoketone species followed by ketone reduction, and that ADH-A is the best ADH out of the enzymes tested, we screened four EREDs previously shown to efficiently convert oxime **1c** to the pyrazine (Scheme S7, Table S5).[1]

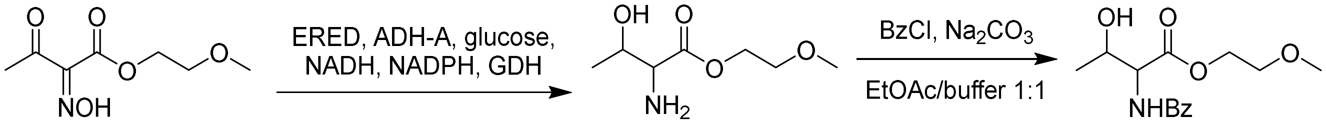

*Scheme S7: Screening of cascade biotransformations with oxime **1c***

*Table S5: Results from cascade biocatalysis with oxime **1c***

#	Substrate	ER[a]	Product formation (%)[b,c]	Ee1 (%)	Ee2 (%)	De (%)	Absolute configuration (major)
1	**1c**	OYE3	50 (27)	>98 (*R*)	93 (*S*)	93	2*R*,3*S*
2		OPR3	44	>98 (*R*)	98 (*S*)	98	2*R*,3*S*
3		FOYE	40	>98 (*R*)	95 (*S*)	95	2*R*,3*S*
4		XenA	11	>98 (*R*)	95 (*S*)	95	2*R*,3*S*

[a] The enzymes originate from: *Arabidopsis thaliana* (OPR3), *Saccharomyces cerevisiae* (OYE3), *Pseudomonas putida* (XenA) and *Rhodococcus ruber* (ADH-A). [b] oxime (10 mM), ERED (purified, 4.45 µM for OYE3, 4.60 µM for OPR3, 5.17 µM for FOYE, 4.89 µM for XenA), (ADH, crude CFE, 2mg/mL for ADH-A), NADH (0.5 mM), NADPH (0.5 mM), glucose (50 mM), GDH (crude CFE, 4 mg/mL). Buffer: 50 mM Na/K Pi, pH 7.5, 5% DMSO v/v. 120 rpm, 24h, 30 °C. Total volume: 0.5 mL. [c] Product formation is defined by the amount of substrate transformed to the aminoalcohol (as the *N*-benzoyl derivative) as deduced from HPLC analysis on a chiral stationary phase using calibration curves. Numbers between parentheses represent isolated yields.

ADH-catalyzed reductions of aminoketones

First, we investigated the optimum amount of ADH-A for the reduction of aminoketone **2a** (Scheme S8, Table S6).

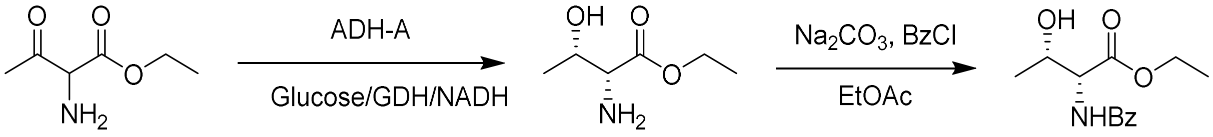

Scheme S8: ADH-A catalysed reduction of aminoketone.

Table S6: Product formations from ADH-A catalyzed reduction of aminoketone

Entry	*ADH-A concentration (mg/mL)*	*Product formation (%)*
1	2	35
2	4	47
**3**	**6**	**52**
4	8	52
5	10	51

Reaction conditions: aminoketone (10 mM), NADH (0.5 mM), glucose (50 mM), ADH-A (heat-treated CFE) GDH (crude CFE, 4 mg/mL). Buffer: 50 mM Pi, pH 7.5, 5% DMSO v/v. 120 rpm, 24h, 30 °C. Total volume: 0.5 mL. Product formation is defined by the amount of substrate transformed to the aminoalcohol (as the *N*-benzoyl derivative) as deduced from HPLC analysis on a chiral stationary phase using calibration curves.

In all reactions, only the D-threonine derivative was found, showing that solely the ADH controls the stereochemical outcome of the reaction.

To prove that the ADHs used for the cascades with oximes **1b** and **1c** control the stereochemical outcomes, the corresponding aminoketones were used as substrate in ADH-catalyzed reductions. When using aminoketone **2c**, an ADH-A concentration of 6 mg/mL was used, the optimum concentration for aminoketone **2a**. This gave a combined product formation of 36%, in a ratio of D/D-allo/L-allo isomers of 96:2:2 (Scheme S9), proving that the ADH also controls the stereochemical outcome in this cascade.

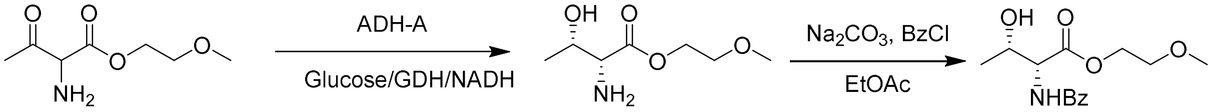

*Scheme S9: Biocatalytic reduction of the aminoketone derivative of oxime **1c**. Reaction conditions: aminoketone (10 mM), NADH (0.5 mM), glucose (50 mM), ADH-A (heat-treated CFE, 6 mg/mL) GDH (crude CFE, 4 mg/mL). Buffer: 50 mM Na/K Pi, pH 7.5, 5% DMSO v/v. 120 rpm, 24h, 30 °C. Total volume: 0.5 mL. Product formation is defined by the amount of substrate transformed to the aminoalcohol (as the N-benzoyl derivative) as deduced from HPLC analysis on a chiral stationary phase using calibration curves.*

Finally, we tested the reduction of aminoketone **2b**, catalyzed by *Lk*ADH-Lica. Using 2-PrOH as cosolvent and as hydride source, product formations of up to 82% were obtained, with stereochemical outcomes highly similar to those observed in cascade biotransformations (Scheme S10, Table S7). This proves that the stereochemical outcome of the cascade with oxime **1b** is fully controlled by the ADH.

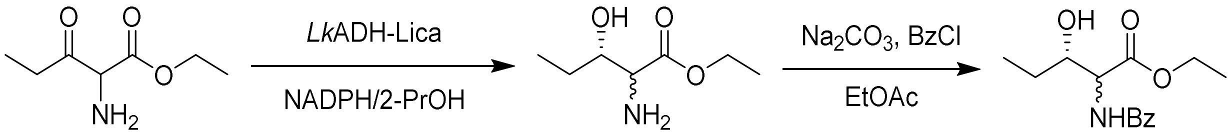

Scheme S10: LkADH-Lica catalyzed reduction of aminoketone

Table S7: Product formations from Lk*ADH-Lica catalyzed reduction of aminoketone.*

Entry	*Lk*ADH-Lica *concentration (mg/mL)*	*Product formation (%)*	*Ratio 2*R,*3*S*/2*S,*3*S
1	2	11	68:32
2	4	56	70:30
3	6	74	72:28
4	8	76	73:27
5	10	82	73:27

Reaction conditions: aminoketone (10 mM), *Lk*ADH-Lica (crude CFE), NADPH (0.5 mM). Buffer: 50 mM Na/K Pi, pH 7.5, 5% 2-PrOH v/v. 120 rpm, 24h, 30 °C. Total volume: 0.5 mL. Product formation is defined by the amount of substrate transformed to the aminoalcohol (as the *N*-benzoyl derivative) as deduced from HPLC analysis on a chiral stationary phase using calibration curves.

Biocatalysis with tyrosine variants

To determine whether the four tyrosine variants generated are active EREDs, we performed a biotransformation using cyclohexenone as substrate (Scheme S11, Table S8).

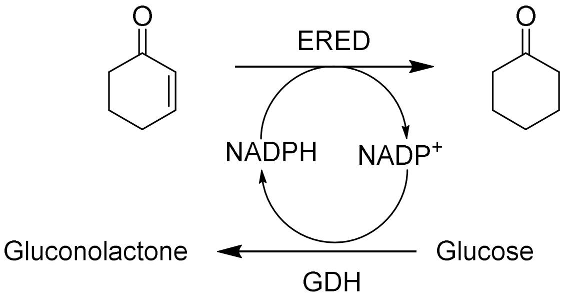

Scheme S11: Biotransformations with cyclohexenone as substrate

Table S8: Results from biotransformations with cyclohexenone.

Enzyme	*Product formation (%)*
XenA_WT	65
XenA_Y27F	59
XenA_Y183F	30
OPR3_WT	61
OPR3_Y190F	55
OPR3_Y370F	85

Conditions: cyclohexenone (50 mM), ERED (purified, 0.2 mg/mL), NADPH (0.5 mM), glucose (50 mM), GDH (4 mg/mL). Buffer: 50 mM Na/K Pi, pH 7.5, 5% DMSO v/v. 120 rpm, 24h, 30 °C. Total volume: 0.5 mL. Product formation is defined by the amount of substrate transformed to cyclohexanone as deduced from GC analysis using calibration curves.

Interestingly, both "canonical" tyrosine variants XenA_Y183F and OPR3_Y190F retain their ability to reduce the C=C double bond in cyclohexenone, albeit, in the case of XenA_Y183F, with reduced product formation. We suspect that in these cases, the proton is supplied by a molecule of water in the active site.

Production of enzymes

General
Enzymes were heterologously expressed in *E. coli* (various strains) under sterile conditions (laminar flow cabinet or Bunsen burner). All glassware was flamed out at 200 °C before use. All media were sterilized by autoclaving (121 °C, 20 min), all antibiotics solutions were sterilized by filtration (0.45 µM sterile filters). Details on all enzymes, literature references, their plasmids and *E. coli* strains used for expression as well as cultivation conditions can be found in Table S11.

Recipes for media, buffers, antibiotics, inducers and additives
LB medium (Lysogeny Broth): yeast extract (5 g/L), sodium chloride (5 g/L), tryptone (10 g/L).

TB medium (Terrific Broth): 9:1 TB base/10xKP_i buffer, mixed just before use.
-TB base: yeast extract (24 g/L), tryptone (12 g/L), glycerol (4 mL/L)
-10x KP_i buffer (1 M potassium phosphate, pH 7.5): KH_2PO_4 (23.1 g/L), K_2HPO_4 (125.4 g/L)

Stock solutions of antibiotics and inducers were as follows:
-Kan: 100 mg/mL kanamycin sulphate in water
-Amp: 50 mg/mL ampicillin sodium in water
-Chlor: 34 mg/mL chloramphenicol in ethanol
-IPTG: 1 M isopropylthio-β-galactoside in water
-AHTC: 4.3 mM anhydrotetracycline in ethanol

50 mM Na/K P_i buffer pH 7.5: $Na_2HPO_4 \cdot 2H_2O$ (7.58 g) and KH_2PO_4 (1.01 g) in water (1 L).

Lysis buffer (buffer A): NaH_2PO_4 (50 mM), NaCl (300 mM), imidazole (20 mM) in water, pH adjusted to 8. Degassed by ultrasonication (20 minutes) and filtered.

Elution buffer (buffer B): NaH_2PO_4 (50 mM), NaCl (300 mM), imidazole (500 mM) in water, pH adjusted to 8. Degassed by ultrasonication (20 minutes) and filtered.

Wild-type ene-reductases

Transformation
A suspension of *E. coli* (100 µL, see Table S11 for strain) was mixed with plasmid solution (3 µM) and placed on ice for 30 min. The cells were then heat-shocked at 42 °C for 10 seconds, followed by addition of LB medium (250 µL) and incubation for one hour (37°C, 450 rpm). 100 µL of this suspension was spread on an LB/agar plate (containing the corresponding antibiotic), which was incubated at 37 °C overnight.

Cultivation
Precultures containing LB medium, the corresponding antibiotic and either a single colony from the transformation agar plate or an aliquot of glycerol stock (1% of total volume of the preculture) were incubated overnight (Table S11). The obtained cultures were used to inoculate the main medium (1% v/v preculture), which were grown until an OD_{600} of 0.6-0.8. Then, enzyme expression was induced with IPTG and incubated overnight (Table S11).

Harvesting
The cells were harvested by centrifugation (4000 rpm, 4 °C, 20 min). The obtained pellets were washed by resuspending them in 0.9% aq. NaCl or 50 mM Na/K P_i buffer (pH 7.5) and centrifuging again (as

above). The washed pellets were resuspended in buffer A (1x 25 mL for 500 mL LB medium, 2x25 mL for 500 mL TB medium) in a 50 mL centrifuge tube, a spatula tip of FMN (approx. 10 mg) was added and the cells were disrupted by sonication on ice (see Table S9 for details).

Table S9: Details on sonication of cells for EREDs

Enzyme	*Amplitude (%)*	*Time on (s)*	*Time off (s)*	*Total pulse time (min)*
OYE3	40	1	4	5
OPR3_WT	40	1	4	5
XenA_WT	40	1	2	5
FOYE	30	2	4	3

The suspensions were centrifuged (18000 rpm, 4 °C, 30 min) and the supernatant filtered through a 0.45 µm filter. This solution was loaded onto a 5 mL His-trap column (pre-washed with water (5 CV) and buffer A (10 CV). Sample loading was done at a flow rate of 1 mL/min. The column was washed with 20 CV buffer A at 5 mL/min, the ERED was then eluted with 4 CV buffer B at 5 mL/min. The yellow fractions obtained from elution with buffer B were collected and concentrated using a 30 kDa Vivaspin column (centrifugation at 4800 rpm, 10 °C) to a volume of 2.5 mL. The buffer was exchanged to 50 mM Na/K P_i (pH 7.5) using a PD_{10} column. The protein solution was diluted to a concentration of 4.0 mg/mL, aliquoted and stored at -20 °C.

For protein crystallization, the protocol was adapted as follow. The cells were harvested by centrifugation with a J-LITE JLA-8.1000 Fixed-Angle Aluminum Rotor (6000 rpm, 30 minutes) and resuspended in lysis buffer (100 mM Tris-HCl, 300 mM NaCl, 25 mM imidazole, pH 8.0) supplemented with a spatula tip of FMN (approx. 10 mg). The cells were destroyed by sonication and the supernatant was recovered by ultracentrifugation with a JA-25.50 Fixed-Angle Aluminum Rotor (20000 rpm, 45 minutes).
The proteins were purified by affinity chromatography (IMAC) using a Histrap FF 5 mL column followed by size-exclusion chromatography (SEC) using a HiLoad® 16/600 Superdex® 200 column. The proteins were eluted using 20 mM Tris-HCl, 100 mM NaCl, pH 8.0 and their purity was confirmed by SDS-PAGE.

For stopped-flow measurements, the proteins were purified exclusively by IMAC using KPi buffer (50 mM KPi, 300 mM NaCl, 25 mM imidazole, pH 7.5). The purified proteins were then dialyzed overnight against 50 mM KPi buffer, pH 7.5. The dialyzed fractions were aliquoted, flash-frozen in liquid nitrogen and stored at -80°C.

Ene-reductase variants

Gene synthesis and transformation
Mutant genes encoding enzyme variants were synthetized and cloned in the respective vectors by Biomatik (Canada). Plasmids encoding XenA and its variants were transformed by heat shock in *E. coli* BL21 Star (pLys). In case of OPR3 and its variants, plasmids were transformed in *E. coli* BL21 CodonPlus using the same protocol. The transformed cells were selected by antibiotic resistance on agar plates supplemented with ampicillin and chloramphenicol. Single colonies were used to prepare the relative glycerol stocks.

Cultivation and Harvesting
The variants were expressed and purified using the same protocol as the wild-type enzymes.

All the variants were cloned in a pET-21a(+) vector (like the wild-type enzymes). Below are the DNA sequences of the variants.

OPR3_Y190F:

CATATGGCCAGCAGTGCCCAGGATGGCAATAATCCGCTGTTTAGTCCGTATAAAATGGGTAAATTCAATCTGA
GCCATCGTGTTGTTCTGGCACCGATGACCCGCTGTCGTGCCCTGAATAATATTCCGCAGGCCGCACTGGGTGA
ATATTATGAACAGCGTGCAACCGCCGGCGGCTTTCTGATTACCGAAGGCACCATGATTAGCCCGACCAGCGCA
GGCTTTCCGCATGTGCCGGGTATTTTTACCAAAGAACAGGTGCGCGAATGGAAAAAGATTGTTGATGTGGTTC
ATGCCAAAGGCGCCGTTATTTTCTGTCAGCTGTGGCATGTGGGCCGTGCCAGCCATGAAGTTTATCAGCCGGC
AGGCGCAGCCCCGATTAGCAGCACCGAAAAACCGATTAGTAATCGCTGGCGTATTCTGATGCCGGATGGTAC
CCATGGCATCTATCCGAAACCGCGCGCAATTGGTACCTATGAAATTAGTCAGGTGGTGGAAGATTATCGTCGT
AGTGCACTGAATGCAATTGAAGCCGGTTTTGATGGTATTGAAATTCATGGTGCCCATGGTTTTCTGATTGATCA
GTTTCTGAAAGATGGCATTAATGATCGCACCGATGAATATGGTGGTAGTCTGGCAAATCGCTGCAAATTCATT
ACCCAGGTTGTTCAGGCAGTTGTTAGCGCAATTGGCGCAGATCGCGTTGGCGTGCGTGTTAGTCCGGCCATTG
ATCATCTGGATGCCATGGATAGTAATCCGCTGAGCCTGGGTCTGGCAGTTGTGGAACGTCTGAATAAGATTCA
GCTGCATAGTGGCAGCAAACTGGCCTATCTGCATGTGACCCAGCCGCGTTATGTGGCATACGGTCAGACCGA
AGCCGGTCGTCTGGGTAGTGAAGAAGAAGCCCGCCTGATGCGCACCCTGCGCAATGCCTATCAGGGTAC
CTTTATTTGCAGCGGTGGCTATACCCGCGAACTGGGCATTGAAGCCGTGGCACAGGGTGACGCCGATCTGGT
GAGCTATGGTCGTCTGTTTATTAGTAATCCGGATCTGGTTATGCGCATTAAGCTGAATGCCCCGCTGAATAAGT
ATAATCGTAAAACCTTTTATACCCAGGATCCGGTTGTGGGTTATACCGATTATCCGTTTCTGCAGGGCAATGGT
AGTAATGGTCCGCTGAGTCGCCTGCTCGAG

OPR3_Y370F:

CATATGGCCAGCAGTGCCCAGGATGGCAATAATCCGCTGTTTAGTCCGTATAAAATGGGTAAATTCAATCTGA
GCCATCGTGTTGTTCTGGCACCGATGACCCGCTGTCGTGCCCTGAATAATATTCCGCAGGCCGCACTGGGTGA
ATATTATGAACAGCGTGCAACCGCCGGCGGCTTTCTGATTACCGAAGGCACCATGATTAGCCCGACCAGCGCA
GGCTTTCCGCATGTGCCGGGTATTTTTACCAAAGAACAGGTGCGCGAATGGAAAAAGATTGTTGATGTGGTTC
ATGCCAAAGGCGCCGTTATTTTCTGTCAGCTGTGGCATGTGGGCCGTGCCAGCCATGAAGTTTATCAGCCGGC
AGGCGCAGCCCCGATTAGCAGCACCGAAAAACCGATTAGTAATCGCTGGCGTATTCTGATGCCGGATGGTAC
CCATGGCATCTATCCGAAACCGCGCGCAATTGGTACCTATGAAATTAGTCAGGTGGTGGAAGATTATCGTCGT
AGTGCACTGAATGCAATTGAAGCCGGTTTTGATGGTATTGAAATTCATGGTGCCCATGGTTATCTGATTGATC
AGTTTCTGAAAGATGGCATTAATGATCGCACCGATGAATATGGTGGTAGTCTGGCAAATCGCTGCAAATTCAT
TACCCAGGTTGTTCAGGCAGTTGTTAGCGCAATTGGCGCAGATCGCGTTGGCGTGCGTGTTAGTCCGGCCATT
GATCATCTGGATGCCATGGATAGTAATCCGCTGAGCCTGGGTCTGGCAGTTGTGGAACGTCTGAATAAGATTC
AGCTGCATAGTGGCAGCAAACTGGCCTATCTGCATGTGACCCAGCCGCGTTATGTGGCATACGGTCAGACCG

AAGCCGGTCGTCTGGGTAGTGAAGAAGAAGAAGCCCGCCTGATGCGCACCCTGCGCAATGCCTATCAGGGTA
CCTTTATTTGCAGCGGTGGCTATACCCGCGAACTGGGCATTGAAGCCGTGGCACAGGGTGACGCCGATCTGG
TGAGCTATGGTCGTCTGTTTATTAGTAATCCGGATCTGGTTATGCGCATTAAGCTGAATGCCCCGCTGAATAAG
TATAATCGTAAAACCTTTTTCACCCAGGATCCGGTTGTGGGTTATACCGATTATCCGTTTCTGCAGGGCAATGG
TAGTAATGGTCCGCTGAGTCGCCTGCTCGAG

XenA_Y27F:

CATATGAGTGCCCTGTTTGAACCGTATACCCTGAAAGATGTTACCCTGCGTAATCGCATTGCAATTCCGCCGAT
GTGCCAGTTTATGGCAGAAGATGGCCTGATTAATGATTGGCATCAGGTTCATTATGCCAGTATGGCCCGTGGT
GGTGCAGGCCTGCTGGTGGTTGAAGCAACCGCAGTTGCACCGGAAGGCCGCATTACCCCGGGCTGTGCAGGC
ATTTGGAGTGATGCCCATGCACAGGCATTTGTTCCGGTGGTTCAGGCAATTAAGGCAGCCGGTAGCGTGCCG
GGCATTCAGATTGCACATGCAGGCCGCAAAGCCAGTGCAAATCGCCCGTGGGAAGGCGATGATCATATTGGC
GCAGATGATGCACGCGGTTGGGAAACCATTGCCCCGAGTGCCATTGCATTTGGCGCACATCTGCCGAATGTGC
CGCGTGCCATGACCCTGGATGATATTGCACGTGTGAAACAGGATTTTGTGGATGCAGCACGTCGTGCCCGTGA
TGCAGGCTTTGAATGGATTGAACTGCATTTTGCACATGGTTATCTGGGCCAGAGTTTCTTTAGCGAACATAGTA
ATAAGCGTACCGATGCCTATGGCGGTAGCTTTGATAATCGTAGTCGCTTTCTGCTGGAAACCCTGGCCGCAGT
GCGCGAAGTGTGGCCGGAAAATCTGCCGCTGACCGCACGTTTTGGCGTGCTGGAATATGATGGCCGCGATGA
ACAGACCCTGGAAGAAAGCATTGAACTGGCACGCCGCTTTAAAGCCGGTGGTCTGGATCTGCTGAGCGTTAG
TGTGGGTTTTACCATTCCGGAAACCAATATTCCGTGGGGTCCGGCATTCATGGGCCCGATTGCCGAACGTGTT
CGTCGCGAAGCCAAACTGCCGGTTACCAGTGCATGGGGTTTTGGTACCCCGCAGCTGGCAGAAGCCGCACTG
CAGGCAAATCAGCTGGATCTGGTGAGCGTTGGCCGCGCACATCTGGCAGATCCGCATTGGGCATATTTTGCCG
CCAAAGAACTGGGCGTGGAAAAAGCCAGCTGGACCCTGCCGGCCCCGTATGCACATTGGCTGGAACGCTATC
GTCTCGAG

XenA_Y183F:

CATATGAGTGCCCTGTTTGAACCGTATACCCTGAAAGATGTTACCCTGCGTAATCGCATTGCAATTCCGCCGAT
GTGCCAGTATATGGCAGAAGATGGCCTGATTAATGATTGGCATCAGGTTCATTATGCCAGTATGGCCCGTGGT
GGTGCAGGCCTGCTGGTGGTTGAAGCAACCGCAGTTGCACCGGAAGGCCGCATTACCCCGGGCTGTGCAGGC
ATTTGGAGTGATGCCCATGCACAGGCATTTGTTCCGGTGGTTCAGGCAATTAAGGCAGCCGGTAGCGTGCCG
GGCATTCAGATTGCACATGCAGGCCGCAAAGCCAGTGCAAATCGCCCGTGGGAAGGCGATGATCATATTGGC
GCAGATGATGCACGCGGTTGGGAAACCATTGCCCCGAGTGCCATTGCATTTGGCGCACATCTGCCGAATGTGC
CGCGTGCCATGACCCTGGATGATATTGCACGTGTGAAACAGGATTTTGTGGATGCAGCACGTCGTGCCCGTGA
TGCAGGCTTTGAATGGATTGAACTGCATTTTGCACATGGTTTTCTGGGCCAGAGTTTCTTTAGCGAACATAGTA
ATAAGCGTACCGATGCCTATGGCGGTAGCTTTGATAATCGTAGTCGCTTTCTGCTGGAAACCCTGGCCGCAGT
GCGCGAAGTGTGGCCGGAAAATCTGCCGCTGACCGCACGTTTTGGCGTGCTGGAATATGATGGCCGCGATGA
ACAGACCCTGGAAGAAAGCATTGAACTGGCACGCCGCTTTAAAGCCGGTGGTCTGGATCTGCTGAGCGTTAG
TGTGGGTTTTACCATTCCGGAAACCAATATTCCGTGGGGTCCGGCATTCATGGGCCCGATTGCCGAACGTGTT
CGTCGCGAAGCCAAACTGCCGGTTACCAGTGCATGGGGTTTTGGTACCCCGCAGCTGGCAGAAGCCGCACTG
CAGGCAAATCAGCTGGATCTGGTGAGCGTTGGCCGCGCACATCTGGCAGATCCGCATTGGGCATATTTTGCCG
CCAAAGAACTGGGCGTGGAAAAAGCCAGCTGGACCCTGCCGGCCCCGTATGCACATTGGCTGGAACGCTATC
GTCTCGAG

Alcohol dehydrogenases

The ADHs were prepared as cell-free extracts (CFE), using a modified version of the general procedure below. Details are shown for each specific enzyme below.

General procedure:

Transformation

A suspension of *E. coli* (100 µL, see Table S11 for strain) was mixed with plasmid solution (3 µM) and placed on ice for 30 min. The cells were then heat-shocked at 42 °C for 10 seconds, followed by addition of LB medium (250 µL) and incubation for one hour (37°C, 450 rpm). 100 µL of this suspension was spread on an LB/agar plate (containing the corresponding antibiotic), which was incubated at 37 °C overnight.

Cultivation

Precultures containing LB medium, the corresponding antibiotic and either a single colony from the transformation agar plate or an aliquot of glycerol stock (1% of total volume of the preculture) were incubated overnight (Table S11). The obtained cultures were used to inoculate the main medium (1% v/v preculture), which were grown until an OD_{600} of 0.6-0.8. Then, enzyme expression was induced with IPTG and incubated overnight (Table S11).

Harvesting

The cells were harvested by centrifugation (4000 rpm, 4 °C, 20 min). The obtained pellets were washed by resuspending them in 0.9% aq. NaCl or 50 mM Na/K P_i buffer (pH 7.5) and centrifuging again (as above). The washed pellets were resuspended in 50 mM Na/K P_i buffer, pH 7.5 (1x 25 mL for 500 mL LB medium, 2x25 mL for 500 mL TB medium) in a 50 mL centrifuge tube and the cells were disrupted by sonication on ice (see Table S9 for details).

Table S10: Details on sonication of cells for ADHs

Enzyme	*Amplitude (%)*	*Time on (s)*	*Time off (s)*	*Total pulse time (min)*
ADH-A	40	1	4	5
*Lb*ADH	20	2	4	2.50
*Lk*ADH	20	2	4	2.50
*Lk*ADH-Lica	20	2	4	4
*Sy*ADH	20	2	4	2.50
*Ras*ADH	30	1	4	5

ADH-A: The general procedure was followed, with the following modifications: 0.1 mg/mL $ZnCl_2$ was added to the main medium (added from sterile 25 mg/mL stock solution in water just before inoculation) and the culture was grown for 24 hours before induction. At induction, amp (100 mg/mL) was added again. For sonication, the pellets were resuspended in 50 mL centrifuge tubes (25 mL per 500 mL LB medium). After sonication (Table S10), the suspension was transferred to a round-bottom flask and heated in an oil bath at 60 °C for 20 minutes. The white, cloudy suspension was centrifuged (14000 rpm, 4 °C, 20 minutes) and the supernatant was shock-frozen in liquid nitrogen and lyophilized overnight.

***Lb*ADH and *Lk*ADH**: The general procedure was followed, with the following modifications: 1 mM $MgCl_2$ (added from a sterile 1 M stock of $MgCl_2 \cdot 6H_2O$ in water) was added to the main medium just before inoculation. The harvested pellet was washed by resuspending it in tris-HCl buffer (100 mM; pH 7.5, containing 1 mM $MgCl_2$, 30 mL/500 mL TB medium) followed by centrifugation (4000 rpm, 4 °C, 20 min). The pellet was resuspended (buffer as above, 40 mL) and split equally over two 50 mL

centrifuge tubes. After sonication (Table S10), the suspensions were centrifuged (14000 rpm, 4 °C, 20 minutes) and the supernatant was shock-frozen in liquid nitrogen and lyophilized overnight.

***Lk*ADH-Lica**: The general procedure was followed, with the following modifications: 1 mM $MgCl_2$ (added from a sterile 1 M stock of $MgCl_2 \cdot 6H_2O$ in water) was added to the main medium just before inoculation. The harvested pellet was washed by resuspending it in MOPS-NaOH buffer (50 mM; pH 7.0, containing 1 mM $MgCl_2$, 30 mL/500 mL TB medium) followed by centrifugation (4000 rpm, 4 °C, 20 min). The pellet was resuspended (buffer as above, 40 mL) and split equally over two 50 mL centrifuge tubes. After sonication (Table S10), the suspensions were centrifuged (14000 rpm, 4 °C, 20 minutes) and the supernatant was shock-frozen in liquid nitrogen and lyophilized overnight.

***Sy*ADH**: The general procedure was followed, with the following modifications: The main culture was grown to an OD_{600} of 1.7 before induction. The pellets were washed by resuspending them in tris-HCl buffer (100 mM, pH 7.5, 20 mL/330 mL TB medium) and centrifuging (4500 rpm, 4 °C, 20 min). The washed pellets were suspended (as above) and sonicated (Table S10). The suspensions were centrifuged (14000 rpm, 4 °C, 20 min) and the supernatants were shock-frozen in liquid nitrogen and lyophilized overnight.

***Ras*ADH**: The general procedure was followed, with the following modifications: The pellets were harvested by centrifugation (8000 rpm, 4 °C, 20 min). The pellets were washed by resupsending them in KP_i buffer (25 mM, pH 7.5) and centrifuging again (as above). For sonication, the washed pellets were suspended in tris-HCl buffer (20 mM, pH 7.4, 0.8 mM $CaCl_2$). After sonication (Table S10), the suspensions were centrifuged (17000 rpm, 4 °C, 25 min) and the supernatants were shock-frozen in liquid nitrogen and lyophilized overnight.

Table S11: Details on enzymes and their preparation

Name	pEG[a]	Source organism	*E. coli* host strain	Plasmid backbone	Tag (C or N terminus)	Medium	Antibiotic		Inducer		Preculture		Expression		
							Name	Final conc. (µg/mL)	Name	Final conc. (mM)	T (°C)	rpm	T(°C)	rpm	time
OYE3[2]	362	*Saccharomyces cerevisiae*	BL21 (DE3)	pET28a	His6 (C)	LB	Kan	50	IPTG	0.2	37	120	20	140	16h
OPR3_ WT[2]	367	*Lycopersicon esculentum*	BL21 (DE3) RIL	pET21d(+)	His6 (N)	LB	Amp & Chlor	50 &34	IPTG	0.5	37	120	25	120	16h
XenA_ WT[2]	145	*Pseudomonas putida*	BL21 (DE3)	pET21a(+)	His6 (C)	TB	Amp	100	IPTG	0.2	37	120	20	120	16h
FOYE[1,3]	369	*Ferrovum sp JA12*	BL21 (DE3)	pET28a(+)	His6 (N)	TB	Kan	50	IPTG	0.05	37	120	25	120	16h
ADH-A[4]	010	*Rhodococcus ruber DSM 44541*	BL21 (DE3)	pET22b	none	LB	Amp	100	IPTG	2	30	120	20	120	24h
*Lb*ADH[5]	180	*Lactobacillus brevis*	C43 (DE3)	pASK-IBA5 plus	Strep (N)	TB	Amp	100	AHTC	0.43 **µM**	30	120	20	120	16h
*Lk*ADH[6]	326	*Lactobacillus kefir*	BL21 (DE3)pLysS	pET21A	None	TB	Amp	100	AHTC	0.43 **µM**	30	120	20	120	16h
*Lk*ADH-Lica[7,8]	475	*Lactobacillus kefir*	BL21 (DE3)	pASK-IBA5 Plus	Strep (N)	TB	Amp	100	AHTC	0.43 **µM**	30	120	20	120	16h
*Sy*ADH[9]	053	*Sphingobium yanoikuyae DSM 6900*	BL21 (DE3)	pET26b	His6 (C)	TB	Kan	50	IPTG	1	30	120	20	120	16h
*Ras*ADH [10]	105	*Ralstonia sp. DSM 6428*	BL21 (DE3)	pET-YSBLIC3C	His6 (N)	LB	Kan	30	IPTG	1	37	180	16	180	16h

[a] Internal reference number. pEG stands for “plasmids of the Elk Group”. Please refer to this number in any inquiries.

DNA sequences

For completeness and easier finding, the previously reported DNA sequences of the used enzymes are listed below.

OYE3:
CCATGGCACC GTTTGTGAAA GGTTTTGAAC CGATTAGCCT GCGTGATACC AACCTGTTTG
AACCTATCAA AATTGGTAAT ACCCAGCTGG CACATCGTGC AGTTATGCCT CCGCTGACCC
GTATGCGTGC AACCCATCCG GGTAATATTC CGAATAAAGA ATGGGCAGCA GTGTATTATG
GTCAGCGTGC ACAGCGTCCG GGTACAATGA TTATTACCGA AGGCACCTTT ATTAGTCCGC
AGGCAGGCGG TTATGATAAT GCACCGGGTA TTTGGAGTGA TGAACAGGTT GCAGAATGGA
AAAACATTTT TCTGGCCATT CATGATTGTC AGAGCTTTGC ATGGGTTCAG CTGTGGTCAC
TGGGTTGGGC AAGCTTTCCG GATGTTCTGG CACGTGATGG TCTGCGTTAT GATTGTGCAA
GCGATCGTGT TTATATGAAT GCAACCCTGC AAGAGAAAGC CAAAGATGCA AATAATCTGG
AACATAGCCT GACCAAAGAT GATATCAAAC AGTATATCAA AGATTATATC CACGCAGCCA
AAAACAGCAT TGCAGCCGGT GCAGATGGTG TTGAAATTCA TAGCGCAAAT GGTTATCTGC
TGAACCAGTT TCTGGATCCG CATAGCAATA AACGTACCGA TGAATATGGT GGCACCATTG
AAAATCGTGC CCGTTTTACC CTGGAAGTTG TTGATGCACT GATTGAAACC ATTGGTCCGG
AACGTGTGGG TCTGCGTCTG AGCCCGTATG GTACATTTAA TAGCATGAGC GGTGGTGCAG
AACCGGGTAT CATTGCACAG TATAGCTATG TTCTGGGTGA ACTGGAAAAA CGTGCAAAAG
CAGGTAAACG TCTGGCATTT GTTCATCTGG TTGAACCGCG TGTTACCGAT CCGAGCCTGG
TTGAAGGTGA AGGCGAATAT AGCGAAGGTA CAAATGATTT TGCCTATAGC ATTTGGAAAG
GTCCGATTAT TCGTGCAGGT AATTATGCAC TGCATCCGGA AGTTGTGCGT GAACAGGTGA
AAGATCCGCG TACCCTGATT GGTTATGGTC GCTTTTTTAT CAGCAATCCG GATCTGGTTT
ATCGTCTGGA AGAAGGTCTG CCGCTGAACA AATATGATCG TAGCACCTTT TATACCATGA
GCGCAGAAGG TTATACCGAT TATCCGACCT ATGAAGAAGC AGTTGATCTG GGTTGGAATA
AAAAC

OPR3_WT:
tttaagaaggagatataccATGGCTAGCCACCACCACCACCACCACATGGCGTCTTCAGCTCAAGATGGAAACAATC
CCCTTTTCTCTCCTTACAAGATGGGCAAGTTCAATCTATCCCACAGGGTAGTATTGGCTCCGATGACAAGGTGC
AGAGCACTGAATAATATTCCACAGGCGGCGCTAGGGGAGTATTACGAGCAGAGAGCGACGGCCGGTGGATT
TCTGATCACTGAAGGCACTATGATTTCTCCGACTTCAGCTGGGTTTCCTCATGTGCCAGGGATTTTCACAAAGG
AACAAGTAAGGGAATGGAAGAAAATAGTTGATGTAGTGCATGCAAAGGGTGCTGTCATATTTTGTCAGCTGT
GGCATGTTGGTCGTGCATCTCATGAAGTGTATCAACCTGCTGGAGCTGCACCAATATCATCCACTGAGAAGCC
TATATCAAATAGGTGGAGAATTCTAATGCCTGATGGAACTCATGGGATTTATCCAAAACCAAGAGCAATTGGA
ACCTATGAGATCTCACAAGTTGTTGAAGATTATCGCAGGTCGGCCTTGAATGCTATTGAAGCAGGTTTCGATG
GTATTGAAATCCATGGAGCTCACGGTTACTTGATTGATCAATTCTTGAAAGATGGGATCAATGACCGGACAGA
TGAGTATGGTGGATCACTAGCCAACCGGTGCAAATTCATCACACAGGTGGTTCAAGCAGTAGTCTCAGCAATA
GGAGCTGATCGCGTAGGCGTTAGAGTTTCACCAGCAATAGATCATCTTGATGCCATGGACTCTAATCCACTCA
GCCTTGGCTTAGCAGTTGTTGAAAGACTAAACAAAATCCAACTCCATTCTGGTTCCAAGCTTGCCTATCTTCAT
GTAACACAGCCACGATACGTAGCATATGGGCAAACTGAAGCAGGCAGACTTGGCAGTGAAGAGGAAGAGGC
TCGTTTAATGAGGACTTTGAGGAACGCGTATCAGGGGACATTCATTTGCAGTGGTGGATACACTAGGGAACT
AGGAATTGAGGCTGTGGCACAAGGTGATGCTGATCTCGTGTCATATGGTCGTCTTTTCATCTCTAATCCTGATT
TGGTTATGAGAATCAAGCTAAATGCACCTCTAAATAAGTATAACAGGAAGACATTCTATACTCAAGATCCAGT
TGTGGGATACACAGATTACCCTTTCCTTCAAGGAAATGGAAGCAATGGACCGTTATCGCGTCTGTGAgaactcga
gcaccaccaccaccaccactga

XenA_WT:
tttaagaaggagatatacatATGAGCGCACTGTTTGAACCGTATACCCTGAAAGATGTTACCCTGCGTAATCGTATTG
CAATTCCGCCTATGTGTCAGTATATGGCAGAAGATGGCCTGATTAACGATTGGCATCAGGTTCATTATGCAAG
CATGGCACGTGGTGGTGCCGGTCTGCTGGTTGTTGAAGCAACCGCAGTTGCACCGGAAGGTCGTATTACACC

GGGTTGTGCCGGTATTTGGTCTGATGCACATGCACAGGCATTTGTTCCGGTTGTTCAGGCAATTAAAGCAGCC
GGTAGCGTTCCGGGTATTCAGATTGCACATGCCGGTCGTAAAGCAAGCGCAAATCGTCCGTGGGAAGGTGAT
GATCATATTGGTGCAGATGATGCACGTGGTTGGGAAACCATTGCACCGAGCGCAATTGCATTTGGTGCACATC
TGCCGAATGTTCCGCGTGCAATGACCCTGGATGATATTGCACGTGTGAAACAGGATTTTGTTGATGCAGCACG
TCGTGCACGTGATGCCGGTTTTGAATGGATCGAACTGCATTTTGCACATGGTTATCTGGGTCAGAGCTTTTTTA
GCGAACATAGCAACAAACGTACCGATGCCTATGGTGGTAGCTTTGATAATCGTAGCCGTTTTCTGCTGGAAAC
CCTGGCAGCAGTTCGTGAAGTTTGGCCTGAAAATCTGCCGCTGACCGCACGTTTTGGTGTTCTGGAATATGAT
GGTCGTGATGAACAGACCCTGGAAGAAAGCATTGAACTGGCACGTCGTTTTAAAGCCGGTGGTCTGGATCTG
CTGTCTGTTAGCGTTGGTTTTACCATTCCGGAAACCAATATTCCGTGGGGTCCGGCATTTATGGGTCCGATTGC
AGAACGTGTTCGTCGTGAAGCAAAACTGCCGGTTACCAGCGCATGGGGTTTTGGTACACCGCAGCTGGCAGA
AGCAGCACTGCAGGCAAATCAGCTGGATCTGGTTAGCGTGGGTCGTGCACATCTGGCAGATCCGCATTGGGC
ATATTTTGCAGCAAAAGAACTGGGTGTTGAAAAAGCAAGCTGGACCCTGCCTGCACCGTATGCACATTGGCTG
GAACGTTATCGTCGGCCGCATCATCACCATCATCACTAAcggccgtaactcgagcaccaccaccaccaccactga

FOYE:
ATGAGCCTGCTGTTTAGCCCGTATCAGCTGGGTAGCCTGAGCCTGGCAAATCGTCTGGTTATTGCACCGATGT
GTCAGTATAGCGCAGTTGATGGTATTGCACAGGATTGGCATCTGATGCATCTGGGTCGTCTGGCAATTAGCGG
TGCAGGTCTGGTGATTGTTGAAGCAACCGGTGTTAATCCGGAAGGTCGTATTACCCCGTTTTGTCTGGGTCTG
TATAACGATGAACAAGAAGCAGCACTGGGTCGTATTGTTGCATTTGCACGTGAATTTGGTCAGGCCAAAATGG
CAATTCAGCTGGCACATGCAGGTCGTAAAGCAAGCACCCGTCGTCCGTGGGATCCGGGTAGTCCGTATAGTCC
GGAAGAAGGTGGTTGGCAGACCTGGGCACCGAGCGCCATTAAATTCTATGAAGAAAGCCTGACACCGCATCC
GATGAGCATTGAAGATCTGGAAACCGTTAAACAGGATTTTGTGAATAGCGCAATTCGTGCAGAACGTGCAGG
TTTTAAAGCAATTGAACTGCATGGTGCACATGGCTATCTGATTCATCAGTTTCTGAGTCCGCTGAGCAATCAGC
GTCAGGATCAGTATGGTGGTAGTCTGGAAAATCGTATGCGTTATCCGCTGGAAATTCTGAGCGCAGTTAAACA
TGCACTGAGTGCAGAAATGGTTGTTGGTATGCGTATTAGCGCAGTGGATTGGGCACCTGGTGGTCTGACCATT
GAAGAAAGTATTACCTTTAGCCAAGAATGCGAAAAACGTGGTGCCGGTTTTATTCATGTTAGTACCGGTGGTC
TGGTTGCACATCAGCAGATTCCGGTTGGTCCGGGTTATCAGGTTGAACATGCACAGGCAATTAAACAGAATGT
GAATATTCCGACCATGGCCGTTGGTCTGATTACCCATAGCGCACAGGCAGAAACCATTCTGAAAAGCGAACAG
GCCGATATGATTGCAATTGCCCGTGCAGCACTGAAAAATCCGCATTGGCCGTGGACCGCAGCGCTGGAACTG
GGTGATAAACCGTTTGCACCGCCTCAGTATCAGCGTGCACGTTAA

ADH-A:
ATGAAAGCCGTCCAGTACACCGAGATCGGCTCCGAGCCGGTCGTTGTCGACATCCCCACCCCGACGCCCGGG
CCGGGTGAGATCCTGCTGAAGGTCACCGCGGCCGGGCTGTGCCACTCGGACATCTTCGTGATGGACATGCCG
GCGGCGCAGTACGCCTACGGCCTGCCGCTCACCCTCGGCCACGAGGGTGTCGGCACCGTCGCCGAACTCGGC
GAGGGCGTCACGGGATTCGGGGTGGGGGACGCCGTCGCCGTGTACGGGCCGTGGGGCTGCGGTGCGTGCC
ACGCCTGCGCGCGCGGCCGGGAGAACTACTGCACCCGCGCCGCCGACCTGGGCATCACGCCACCCGGTCTCG
GCTCGCCCGGATCGATGGCCGAGTACATGATCGTCGATTCGGCGCGCCACCTCGTCCCGATCGGAGACCTCGA
CCCGGTCGCCGCGGCGCCGCTCACCGACGCCGGTCTGACGCCGTACCACGCGATCTCCCGGGTCCTGCCGCTG
CTGGGGCCGGGCTCGACGGCCGTCGTCATCGGTGTCGGCGGGCTCGGCCACGTCGGCATCCAGATCCTGCGC
GCCGTCAGCGCGGCCCGTGTGATCGCCGTCGACCTCGACGACGACCGTCTCGCCCTCGCCCGCGAGGTCGGC
GCCGACGCGGCGGTGAAGTCGGGCGCCGGTGCGGCGGACGCGATCCGGGAACTGACCGGCGGCCAGGGCG
CGACGGCGGTGTTCGACTTCGTCGGCGCCCAGTCGACGATCGACACGGCGCAGCAGGTGGTCGCGGTCGAC
GGGCACATCTCGGTCGTGGGCATCCACGCCGGCGCACACGCCAAGGTCGGGTTCTTCATGATCCCGTTCGGC
GCCTCCGTCGTGACCCCGTACTGGGGCACCCGGTCGGAACTGATGGAGGTCGTCGCGCTGGCCCGCGCCGGC
CGGCTGGACATCCACACCGAGACGTTCACCCTCGACGAGGGGCCGGCGGCGTACCGGCGGCTGCGCGAGGG
CAGCATCCGCGGCCGCGGCGTGGTGGTTCCCTGA

*Lb*ADH:
ATGGCTAGCTGGAGCCACCCGCAGTTCGAAAAAGGCGCCATGAGCAATCGTCTGGATGGTAAAGTTGCAATT
ATTACCGGTGGCACCCTGGGTATTGGTCTGGCAATTGCAACCAAATTTGTTGAAGAAGGTGCCAAAGTGATGA

TTACCGGTCGTCATAGTGATGTTGGTGAAAAAGCAGCAAAAAGCGTTGGTACACCGGATCAGATTCAGTTTTT
TCAGCATGATAGCAGTGATGAAGATGGTTGGACCAAACTGTTTGATGCAACCGAAAAAGCATTTGGTCCGGTT
AGCACCCTGGTTAATAATGCAGGTATTGCCGTTAATAAAAGCGTTGAAGAAACCACCACCGCAGAATGGCGT
AAACTGCTGGCAGTTAATCTGGATGGTGTTTTTTTGGCACCCGTCTGGGTATTCAGCGCATGAAAAATAAAG
GTCTGGGTGCCAGCATTATTAATATGAGCAGCATTGAAGGCTTTGTTGGTGATCCGAGCCTGGGTGCATATAA
TGCAAGCAAAGGTGCAGTTCGCATTATGAGCAAAAGCGCAGCACTGGATTGTGCACTGAAAGATTATGATGT
TCGTGTGAATACCGTGCATCCGGGTTATATTAAAACACCGCTGGTTGATGATCTGCCTGGTGCCGAAGAAGCA
ATGAGCCAGCGTACCAAAACCCCGATGGGTCATATTGGTGAACCGAATGATATTGCCTATATTTGTGTTTATCT
GGCCAGCAATGAAAGCAAATTTGCAACAGGTAGCGAATTTGTTGTGGATGGTGGTTATACCGCACAGTAATG
A

*Lk*ADH:
CATATGACCGATCGTCTGAAAGGTAAAGTTGCAATTGTTACCGGTGGCACCCTGGGTATTGGTCTGGCAATTG
CAGATAAATTTGTTGAAGAAGGTGCCAAAGTTGTTATTACCGGTCGTCATGCAGATGTTGGTGAAAAAGCAGC
AAAAAGCATTGGTGGCACCGATGTTATTCGTTTTGTTCAGCATGATGCAAGTGATGAAGCAGGTTGGACCAAA
CTGTTTGATACCACCGAAGAAGCATTTGGTCCGGTTACCACCGTTGTTAATAATGCAGGTATTGCAGTTAGCAA
AAGCGTTGAAGATACCACCACAGAAGAATGGCGTAAACTGCTGAGCGTTAATCTGGATGGTGTTTTTTTGGC
ACCCGTCTGGGTATTCAGCGTATGAAAAACAAAGGTCTGGGTGCCAGCATTATCAATATGAGCAGCATTGAA
GGTTTTGTTGGTGATCCGACCCTGGGTGCATATAATGCAAGCAAAGGTGCAGTTCGTATTATGAGCAAAAGTG
CAGCACTGGATTGTGCCCTGAAAGATTATGATGTTCGTGTTAATACCGTTCACCCTGGCTATATCAAAACACCG
CTGGTTGATGATCTGGAAGGTGCCGAAGAAATGATGAGCCAGCGTACCAAAACCCCGATGGGTCATATTGGT
GAACCGAATGATATTGCCTGGATTTGTGTTTATCTGGCCAGTGATGAAAGTAAATTTGCGACCGGTGCCGAAT
TTGTTGTTGATGGTGGTTATACCGCACAGTAACTCGAG

*Lk*ADH-Lica:
atggctagctggagccacccgcagttcgaaaaaggcgccgagaccATGACCGATCGTCTGAAGGGCAAAGTAGCCATCGTAA
CCGGCGGGACACACGGTATCGGTTTGGCAATCGCCACTAAATTTGTAACAGAGGGTGCGAAAGTAGTTATTA
CTGGTCGTCGCGCGGATGTTGGTGAAAAGGCCGCCAAATCAATCGGCGGCACTGATGTTATTCGCTTTGTCCA
GCACGATGTTTCCGATGAAGCAGGCTGGGGCAAACTGTTCGACACCACCGAGGAGACATTCGGCCCGGTTAC
GACCCTGGTGAACAATGCAGGGATTGGCTATCGCAAAAGCGTTGAAGACACTACCACGGAGGAATGGCGTA
AACTGCTGTCCGTTAATCTGGATGGTGTTTTTTTCGGCACCCGTCTGGGCATTCAGCGCATGAAATGTAAAGG
CTTGGGCGCTAGCATCATCAATATGAGCAGTATTCTGGGGCAGGTAGGCGATCCGGCGACTGGGGCATACTC
CGCTTCCAAGGGGGCGGTACGTATCATGTCGAAAAGCGCAGCGCTGCTGTGCGCACTGAAGGACTACGATGT
GCGTGTCAACACAGTACATCCGGGCCCCATCAAGACCCCGTTAATGGATGATATGCCGGGTGCTGAGGAAAT
GATGTCACAGCGTACGAAAACCCCTATGGGTCACATTGGCGAACCGAATGACATCGCATGGGTCTGTGTGTAC
CTGGCATCTGACGAATCGAAATTTGCGACGGGTGCAGAATTTGTGGTCGACGGCGGGTATACCGCACAGTGA
ggtctctgatatctaactaagcttgacctg

*Sy*ADH:
aggccacgtgtcttgtccaggtacccatATGACCACCCTGCCGACCGTTCTGATTACCGGTGCAAGCAGCGGTATTGGTG
CAACCTATGCAGAACGTTTTGCACGTCGTGGTCATGATCTGGTTCTGGTTGCACGTGATAAAGTTCGTCTGGAT
GCACTGGCAGCACGTCTGCGTGATGAAAGCGGTGTTGCAGTTGAAGCACTGCAGGCAGATCTGACCCGTCCG
GCAGATCTGGCAGCAGTTGAAATTCGTCTGCGTGAAGATGCACGTATTGGCATTCTGATTAATAATGCAGGTA
TGGCACAGAGCGGTGGTTTTGTTCAGCAGACCGCAGAAGGTATTGAACGTCTGATTACCCTGAATACCACCGC
ACTGACCCGTCTGGCTGCAGCAGTTGCACCGCGTTTTGTTCAGAGCGGCACCGGTGCAATTGTTAATATTGGT
AGCGTTGTTGGTTTTGCACCGGAATTTGGTATGAGCATTTATGGTGCAACCAAAGCCTTTGTTCTGTTTCTGAG
CCAGGGTCTGAATCTGGAACTGAGCCCGAGCGGTATTTATGTTCAGGCAGTTCTGCCTGCAGCAACCCGTACC
GAAATTTGGGGTCGTGCAGGTATTGATGTTAATACCCTGCCGGAAGTTATGGAAGTTGATGAACTGGTTGAT
GCAGCACTGGTTGGTTTTGATCGTCGTGAACTGGTTACCATTCCTCCGCTGCATGTTGCAGCACGTTGGGATG
CACTGGATGGTGCACGTCAGGGTCTGATGAGCGATATTCGTCAGGCACAGGCAGCAGATCGTTATCGTCCGG
AAGCAAGCGCATGGTCACATCCGCAGTTTGAAAAATAATAActcgagctctggagcacaagactggcctcatgggcct

*Ras*ADH:
ATGGGCAGCA GCCATCATCA TCATCATCAC AGCAGCGGCC TGGAAGTTCT GTTCCAGGGA
CCAGCAATGT ATCGTCTGCT GAATAAAACC GCAGTTATTA CCGGTGGTAA TAGCGGTATT
GGTCTGGCAA CCGCAAAACG TTTTGTTGCC GAAGGTGCCT ATGTTTTTAT TGTTGGTCGT
CGTCGTAAAG AACTGGAACA GGCAGCAGCA GAAATTGGTC GTAATGTTAC CGCAGTTAAA
GCCGATGTTA CCAAACTGGA AGATCTGGAT CGTCTGTATG CAATTGTTCG TGAACAGCGT
GGTAGCATTG ATGTTCTGTT TGCAAATAGC GGTGCCATTG AACAGAAAAC CCTGGAAGAA
ATTACACCGG AACATTATGA TCGCACCTTT GATGTTAATG TGCGTGGTCT GATTTTTACC
GTTCAGAAAG CACTGCCGCT GCTGCGTGAT GGTGGTAGCG TTATTCTGAC CAGCAGCGTT
GCCGGTGTTC TGGGTCTGCA GGCACATGAT ACCTATAGCG CAGCAAAAGC AGCAGTTCGT
AGCCTGGCAC GTACCTGGAC CACCGAACTG AAAGGTCGTA GCATTCGTGT TAATGCAGTT
AGTCCGGGTG CAATTGATAC CCCGATTATT GAAAATCAGG TTAGCACCCA GGAAGAAGCA
GACGAACTGC GCGCAAAATT TGCAGCAGCA ACACCGCTGG GTCGTGTTGG TCGTCCGGAA
GAACTGGCAG CAGCCGTTCT GTTTCTGGCA AGTGATGATA GCAGCTATGT TGCAGGTATT
GAACTGTTTG TTGATGGTGG TCTGACCCAG GTTTAA

Protein crystallization

The purified proteins were concentrated to approximately 10 mg/mL (see Table S12 for exact values) and crystallization trials were set up using the sitting-drop vapor diffusion method. Solutions from Morpheus I, Index, and JCSG+ screens were mixed to the protein solutions on a 96-well 3-lens crystallization plate using an Oryx 8 crystallization robot. The volume of each drop was 1μL. In all cases, yellow protein crystals appeared after 4-5 days in different conditions. The quality of the crystals was improved employing micro-seeding from the first screens. Only in a few cases, optimization of the initial conditions was required.

For the soaking procedure, a fresh stock of oxime saturated solution was prepared every time in 20 mM Tris-HCl buffer (pH 8.0). The saturating concentration was estimated by weighing 10.0 mg of oxime and adding small aliquots of buffer until complete solubilization. An approximate saturating concentration of 800 mM was estimated for oximes **1a** and **1c**, while only 200 mM was estimated for oxime **1b**.

To prepare the saturated stock solution, an aliquot of oxime was added in 500 μL of 20 mM Tris-HCl buffer (pH 8.0). The excess of oxime was separated from the saturated solutions by centrifugation and the supernatant was used to prepare 400 mM and 200 mM stocks for soaking. The soaking solution was obtained by mixing 1 μL of reservoir solution with 1 μL of oxime stock solution, directly on a free well of the crystallization plate. The crystals were harvested from the crystallization drop and placed in the soaking solution for different times. After this, the crystals were flash-frozen in liquid nitrogen without additional cryoprotection.

For the co-crystallization, a stock solution of oxime was mixed to a concentrated protein solution in order to obtain a final protein concentration identical to the one used for crystallization. After spinning down the precipitated protein, this solution was used for co-crystallization.

Data collection and structure solution

Diffraction data from various crystals were measured remotely at different beamlines at the ESRF (Grenoble) and DESY (Hamburg). Diffraction images were processed using the AutoPROC[11] pipeline including XDS[12], Pointless[13], Aimless[14], CCP4[15] and STARANISO (Global Phasing Ltd., United Kingdom). The structures of XenA in complex with oxime **1b** and OPR3 in complex with oxime **1a** were solved by molecular replacement using available structures of the two enzymes (PDB-entries 3N19 and 3HGS, respectively) as search models. The remaining structures were solved using the first two as starting models. Structures of the ligands were generated using Schrödinger[16] and processed with eLBOW available in Phenix package[17] to generate the corresponding CIF-files with geometric restraints. All the data are reported in Table 1 format (Table S13-18)

Table S12. Conditions used for crystallization of XenA, OPR3 and the relative variants in complex with the oximes substrates.

Structure (PDB)	Enzyme concentration	Oxime concentration (soaked/cocrystallized)	Reservoir solution	Protein:reservoir mix
**XenA-oxime 1a complex (8A8I)**	9.0 mg/mL	400 mM (soaked)	90 mM halogen mix (30 mM NaF, 30 mM NaBr, 30 mM NaI), 5% MPD, 25% PEG 3350, 100 mM Tris pH 7.5	2:1
**XenA-oxime 1b complex (8AU8)**	9.3 mg/mL	100 mM (soaked)	90 mM halogen mix (30 mM NaF, 30 mM NaBr, 30 mM NaI), 25% MPD, 10% PEG 3350, 100 mM Tris pH 7.5	1:1
**XenA-oxime 1c complex (8AU9)**	9.6 mg/mL	400 mM (soaked)	Morpheus I, condition B8	1:1
**OPR3-oxime 1a complex (8AUA)**	11.5 mg/mL	100 mM (cocrystal.)	Morpheus I, condition G10	2:1
**OPR3-oxime 1b complex (8AUB)**	13.9 mg/mL	100 mM (soaked)	100 mM CaCl2,10 % w/v PEG 8000, 100 mM Tris pH 8.5	3 :1
**OPR3-oxime 1c complex (8AUE)**	10.0 mg/mL	200 mM (cocrystal.)	Morpheus I, condition H10	2:1
**XenA Y27F-oxime 1a complex (8AUH)**	10.4 mg/mL	100 mM (cocrystal.)	Morpheus I, condition A8	1:1
**XenA Y27F-oxime 1c complex (8AUI)**	10.4 mg/mL	400 mM (soaked)	Morpheus I, condition A8	1:1
**XenA Y183F-oxime 1a complex (8AUG)**	9.6 mg/mL	400 mM (soaked)	Morpheus I, condition A12	2:1
**XenA Y183F-oxime 1b complex (8AUF)**	9.6 mg/mL	100 mM (soaked)	Index, condition 84	1:1
**OPR3 Y190F-oxime 1a complex (8AUJ)**	11.2 mg/mL	200 mM (cocrystal.)	Morpheus I, condition G10	1:1
**OPR3 Y190F-oxime 1b complex (8AUM)**	11.2 mg/mL	100 mM (cocrystal.)	Morpheus I, condition C7	2:1
**OPR3 Y190F-oxime 1c complex (8AUL)**	11.2 mg/mL	400 mM (soaked)	Morpheus I, condition A3	1:1
**OPR3 Y370F-oxime 1a complex (8AUN)**	11.1 mg/mL	200 mM (cocrystal.)	Morpheus I, condition G2	2:1
**OPR3 Y370F-oxime 1b complex (8AUQ)**	11.1 mg/mL	100 mM (cocrystal.)	Morpheus I, condition G10	2.1
**OPR3 Y370F-oxime 1c complex (8AUO)**	11.1 mg/mL	200 mM (cocrystal.)	Morpheus I, condition D9	2:1

Table S13. Statistics of X-ray data collection and refinement of the crystal structures of XenA wt. Statistics for the highest-resolution shell are shown in parentheses.

	XenA wt		
	Complex with **oxime 1a**	Complex with **oxime 1b**	Complex with **oxime 1c**
**Wavelength**	0.9677	0.968	1.03322
**Resolution range**	44.67 - 1.88 (1.947 - 1.88)	38.87 - 1.58 (1.636 - 1.58)	45.68 - 1.5 (1.554 - 1.5)
**Space group**	P 21 21 2	P 21 21 2	P 2 21 21
**Unit cell**	83.149 158.863 49.672 90 90 90	84.165 157.966 57.614 90 90 90	57.9302 84.0004 158.843 90 90 90
**Total reflections**	255258 (21610)	483032 (48681)	708080 (64380)
**Unique reflections**	99224 (8305)	104533 (10472)	124140 (12205)
**Multiplicity**	2.6 (2.3)	4.6 (4.6)	5.7 (5.3)
**Completeness (%)**	94.33 (79.72)	98.63 (99.85)	99.48 (99.07)
**Mean I/sigma(I)**	3.64 (1.00)	5.51 (1.77)	7.85 (1.27)
**Wilson B-factor**	18.18	17.61	15.33
**R-merge**	0.2049 (1.084)	0.1624 (0.7661)	0.1099 (1.329)
**R-meas**	0.258 (1.406)	0.1841 (0.8668)	0.1207 (1.472)
**R-pim**	0.1547 (0.8841)	0.08414 (0.3939)	0.04913 (0.6222)
**CC1/2**	0.951 (0.704)	0.973 (0.763)	0.997 (0.696)
**CC***	0.987 (0.909)	0.993 (0.93)	0.999 (0.906)
**Reflections used in refinement**	98045 (8212)	104533 (10470)	124140 (12173)
**Reflections used for R-free**	1440 (125)	1113 (112)	6317 (611)
**R-work**	0.1838 (0.4582)	0.1639 (0.3296)	0.1783 (0.3950)
**R-free**	0.2367 (0.4489)	0.1892 (0.3346)	0.2031 (0.3985)
**CC(work)**	0.918 (0.374)	0.935 (0.803)	0.942 (0.792)
**CC(free)**	0.920 (0.541)	0.936 (0.788)	0.937 (0.787)
**Number of non-hydrogen atoms**	6383	6674	6483
**macromolecules**	5683	5771	5696
**ligands**	120	150	132
**solvent**	580	753	655
**Protein residues**	718	724	720
**RMS(bonds)**	0.007	0.006	0.005
**RMS(angles)**	0.88	0.84	0.81
**Ramachandran favored (%)**	96.08	96.49	96.23
**Ramachandran allowed (%)**	3.92	3.23	3.63
**Ramachandran outliers (%)**	0	0.28	0.14
**Rotamer outliers (%)**	1.07	1.05	0.71
**Clashscore**	2.11	2.14	1.83
**Average B-factor**	20.65	22.39	22.45
**macromolecules**	19.74	20.86	21.39
**ligands**	20.47	28.64	26.31
**solvent**	29.53	32.89	30.91
**PDB deposition**	8A8I	8AU8	8AU9

Table S14. Statistics of X-ray data collection and refinement of the crystal structures of OPR3 wt. Statistics for the highest-resolution shell are shown in parentheses.

	OPR3 wt		
	Complex with **oxime 1a**	Complex with **oxime 1b**	Complex with **oxime 1c**
**Wavelength**	0.978564	0.965459	0.965459
**Resolution range**	48.95 - 1.9 (1.968 - 1.9)	44.2 - 1.62 (1.678 - 1.62)	44.51 - 1.82 (1.885 - 1.82)
**Space group**	P 1 21 1	P 1 21 1	P 1 21 1
**Unit cell**	49.521 90.797 89.945 90 98.721 90	49.794 88.397 89.921 90 99.904 90	49.719 89.026 90.074 90 100.274 90
**Total reflections**	187858 (12623)	276550 (28456)	196774 (20482)
**Unique reflections**	57745 (4029)	176405 (18096)	123419 (12649)
**Multiplicity**	3.3 (3.1)	1.6 (1.6)	1.6 (1.6)
**Completeness (%)**	93.04 (65.02)	91.63 (94.27)	90.44 (92.81)
**Mean I/sigma(I)**	8.31 (2.23)	5.06 (1.19)	6.25 (1.24)
**Wilson B-factor**	20.15	17.69	21.05
**R-merge**	0.106 (0.5869)	0.08339 (0.5163)	0.07937 (0.5119)
**R-meas**	0.1273 (0.7057)	0.117 (0.7156)	0.1116 (0.715)
**R-pim**	0.06959 (0.3874)	0.08196 (0.4938)	0.07835 (0.4981)
**CC1/2**	0.991 (0.698)	0.987 (0.619)	0.993 (0.677)
**CC***	0.998 (0.907)	0.997 (0.875)	0.998 (0.898)
**Reflections used in refinement**	57726 (4029)	176134 (18096)	123305 (12641)
**Reflections used for R-free**	2753 (201)	1852 (189)	2097 (211)
**R-work**	0.2094 (0.2952)	0.1921 (0.2847)	0.1717 (0.2849)
**R-free**	0.2683 (0.3378)	0.2145 (0.3319)	0.2048 (0.3075)
**CC(work)**	0.938 (0.848)	0.957 (0.773)	0.965 (0.838)
**CC(free)**	0.902 (0.803)	0.964 (0.671)	0.972 (0.853)
**Number of non-hydrogen atoms**	6374	6453	6418
**macromolecules**	5807	5929	5842
**ligands**	84	121	111
**solvent**	483	403	465
**Protein residues**	734	742	734
**RMS(bonds)**	0.007	0.006	0.006
**RMS(angles)**	0.91	0.81	0.82
**Ramachandran favored (%)**	97.09	97.28	97.25
**Ramachandran allowed (%)**	2.77	2.72	2.75
**Ramachandran outliers (%)**	0.14	0	0
**Rotamer outliers (%)**	2.92	1.6	1.3
**Clashscore**	5.73	4	3.05
**Average B-factor**	24.34	21.33	24.21
**macromolecules**	24.01	20.88	23.67
**ligands**	21.05	23.13	25.25
**solvent**	28.94	27.39	30.65
**PDB deposition**	8AUA	8AUB	8AUE

Table S15. Statistics of X-ray data collection and refinement of the crystal structures of XenA Y183F variant. Statistics for the highest-resolution shell are shown in parentheses.

	XenA Y183F	
	Complex with **oxime 1a**	Complex with **oxime 1b**
**Wavelength**	1.03319	1.03319
**Resolution range**	46.37 - 1.351 (1.4 - 1.351)	46.5 - 1.351 (1.399 - 1.351)
**Space group**	P 21 21 2	P 21 21 2
**Unit cell**	84.07 157.63 57.34 90 90 90	84.35 158.69 57.38 90 90 90
**Total reflections**	1104040 (103478)	1108408 (105704)
**Unique reflections**	165361 (15877)	164258 (15636)
**Multiplicity**	6.7 (6.5)	6.7 (6.8)
**Completeness (%)**	98.95 (95.96)	97.21 (93.30)
**Mean I/sigma(I)**	15.54 (2.05)	20.01 (3.78)
**Wilson B-factor**	13.85	13.26
**R-merge**	0.06493 (0.8722)	0.05009 (0.409)
**R-meas**	0.0704 (0.9473)	0.05429 (0.4425)
**R-pim**	0.0269 (0.3643)	0.02068 (0.1668)
**CC1/2**	0.999 (0.8)	0.999 (0.954)
**CC***	1 (0.943)	1 (0.988)
**Reflections used in refinement**	165353 (15873)	164258 (15631)
**Reflections used for R-free**	2099 (201)	2100 (200)
**R-work**	0.1569 (0.3070)	0.1479 (0.1862)
**R-free**	0.1730 (0.3362)	0.1598 (0.2000)
**CC(work)**	0.942 (0.815)	0.974 (0.949)
**CC(free)**	0.936 (0.705)	0.963 (0.905)
**Number of non-hydrogen atoms**	6854	7060
**macromolecules**	5827	5939
**ligands**	161	129
**solvent**	866	992
**Protein residues**	724	721
**RMS(bonds)**	0.005	0.005
**RMS(angles)**	0.84	0.85
**Ramachandran favored (%)**	96.11	96.09
**Ramachandran allowed (%)**	3.89	3.91
**Ramachandran outliers (%)**	0	0
**Rotamer outliers (%)**	1.56	0.51
**Clashscore**	1.71	1.51
**Average B-factor**	18.97	18.25
**macromolecules**	17.36	16
**ligands**	20.71	18.58
**solvent**	29.48	31.71
**PDB deposition**	8AUG	8AUF

Table S16. Statistics of X-ray data collection and refinement of the crystal structures of XenA Y27F variant. Statistics for the highest-resolution shell are shown in parentheses.

	**XenA Y27F**	
	Complex with **oxime 1a**	Complex with **oxime 1c**
**Wavelength**	1.03319	1.03319
**Resolution range**	47.84 - 1.352 (1.401 - 1.352)	40.67 - 1.54 (1.595 - 1.54)
**Space group**	P 21 21 2	P 2 21 21
**Unit cell**	83.71 160.35 50.12 90 90 90	57.77 84.18 157.85 90 90 90
**Total reflections**	963904 (89901)	765444 (68850)
**Unique reflections**	147314 (14197)	113912 (11124)
**Multiplicity**	6.5 (6.3)	6.7 (6.2)
**Completeness (%)**	99.61 (97.31)	99.41 (98.09)
**Mean I/sigma(I)**	28.21 (6.70)	17.69 (2.47)
**Wilson B-factor**	11.17	17.04
**R-merge**	0.03677 (0.2198)	0.0623 (0.7223)
**R-meas**	0.03997 (0.2396)	0.06757 (0.7884)
**R-pim**	0.01549 (0.09431)	0.02584 (0.3109)
**CC1/2**	1 (0.976)	0.999 (0.877)
**CC***	1 (0.994)	1 (0.967)
**Reflections used in refinement**	147314 (14197)	113892 (11121)
**Reflections used for R-free**	2101 (202)	1439 (141)
**R-work**	0.1414 (0.1603)	0.1596 (0.2731)
**R-free**	0.1582 (0.1829)	0.1822 (0.2773)
**CC(work)**	0.974 (0.947)	0.972 (0.928)
**CC(free)**	0.958 (0.922)	0.966 (0.903)
**Number of non-hydrogen atoms**	6762	6706
**macromolecules**	5814	5743
**ligands**	145	154
**solvent**	803	809
**Protein residues**	725	720
**RMS(bonds)**	0.005	0.005
**RMS(angles)**	0.85	0.8
**Ramachandran favored (%)**	96.53	96.23
**Ramachandran allowed (%)**	3.33	3.63
**Ramachandran outliers (%)**	0.14	0.14
**Rotamer outliers (%)**	0.69	0.88
**Clashscore**	1.2	2.43
**Average B-factor**	14.68	22.42
**macromolecules**	13.33	20.87
**ligands**	13.03	25.58
**solvent**	24.72	32.8
**PDB deposition**	8AUH	8AUI

Table S17. Statistics of X-ray data collection and refinement of the crystal structures of OPR3 Y190F variant. Statistics for the highest-resolution shell are shown in parentheses.

	**OPR3 Y190F**		
	Complex with **oxime 1a**	Complex with **oxime 1b**	Complex with **oxime 1c**
**Wavelength**	0.9677	1.03319	1.03319
**Resolution range**	35.94 - 1.57 (1.626 - 1.57)	45.92 - 1.35 (1.398 - 1.35)	41.52 - 1.5 (1.554 - 1.5)
**Space group**	P 1 21 1	P 1 21 1	P 1 21 1
**Unit cell**	49.456 92.724 90.343 90 99.314 90	49.38 92.1 89.96 90 99.11 90	49.37 93.95 89.51 90 97.4 90
**Total reflections**	385321 (39893)	579024 (56678)	438551 (39879)
**Unique reflections**	210187 (21511)	165476 (15988)	126574 (12353)
**Multiplicity**	1.8 (1.9)	3.5 (3.5)	3.5 (3.2)
**Completeness (%)**	94.97 (97.33)	95.14 (92.14)	97.89 (95.81)
**Mean I/sigma(I)**	12.31 (2.92)	22.41 (4.86)	14.83 (2.37)
**Wilson B-factor**	17.47	14.42	16.95
**R-merge**	0.03617 (0.266)	0.02837 (0.1811)	0.0487 (0.5543)
**R-meas**	0.04953 (0.3592)	0.03354 (0.2129)	0.05766 (0.6645)
**R-pim**	0.03366 (0.2397)	0.0177 (0.1109)	0.03052 (0.3609)
**CC1/2**	0.998 (0.862)	0.999 (0.98)	0.999 (0.802)
**CC***	0.999 (0.962)	1 (0.995)	1 (0.943)
**Reflections used in refinement**	210187 (21511)	165475 (15967)	126571 (12350)
**Reflections used for R-free**	2204 (229)	2097 (203)	1550 (151)
**R-work**	0.1543 (0.2129)	0.1645 (0.2158)	0.1576 (0.2279)
**R-free**	0.1620 (0.2408)	0.1836 (0.2555)	0.1802 (0.2469)
**CC(work)**	0.971 (0.912)	0.966 (0.932)	0.971 (0.901)
**CC(free)**	0.975 (0.841)	0.964 (0.890)	0.967 (0.821)
**Number of non-hydrogen atoms**	6657	6954	6499
**macromolecules**	5978	6183	5835
**ligands**	92	126	105
**solvent**	587	645	559
**Protein residues**	732	732	729
**RMS(bonds)**	0.006	0.005	0.005
**RMS(angles)**	0.8	0.84	0.83
**Ramachandran favored (%)**	97.1	97.65	97.64
**Ramachandran allowed (%)**	2.9	2.35	2.36
**Ramachandran outliers (%)**	0	0	0
**Rotamer outliers (%)**	1.58	1.52	2.09
**Clashscore**	4.72	3.99	3.05
**Average B-factor**	22.06	20.38	21.27
**macromolecules**	21.17	19.42	20.4
**ligands**	18.31	19.1	20.36
**solvent**	31.79	29.87	30.5
**PDB deposition**	8AUJ	8AUM	8AUL

Table S18. Statistics of X-ray data collection and refinement of the crystal structures of OPR3 Y370F variant. Statistics for the highest-resolution shell are shown in parentheses.

	OPR3 Y370F		
	Complex with **oxime 1a**	Complex with **oxime 1b**	Complex with **oxime 1c**
**Wavelength**	0.9677	0.967697	0.9677
**Resolution range**	33.59 - 1.492 (1.545 - 1.492)	32.6 - 1.424 (1.475 - 1.424)	45.83 - 1.58 (1.636 - 1.58)
**Space group**	P 1 21 1	P 1 21 1	P 1 21 1
**Unit cell**	49.312 92.833 90.151 90 99.322 90	49.311 92.602 89.982 90 99.235 90	49.174 92.222 90.257 90 99.253 90
**Total reflections**	446447 (39367)	1039325 (105414)	376295 (38847)
**Unique reflections**	241139 (20980)	146068 (14447)	106577 (10783)
**Multiplicity**	1.9 (1.9)	7.1 (7.3)	3.5 (3.6)
**Completeness (%)**	93.85 (81.56)	98.12 (97.55)	98.10 (99.60)
**Mean I/sigma(I)**	9.49 (2.91)	14.62 (2.28)	12.05 (3.21)
**Wilson B-factor**	15.84	17.41	17.39
**R-merge**	0.051 (0.2437)	0.06761 (0.7516)	0.07211 (0.5707)
**R-meas**	0.06994 (0.3292)	0.07306 (0.8081)	0.08558 (0.6752)
**R-pim**	0.04762 (0.2198)	0.0274 (0.2949)	0.0455 (0.3561)
**CC1/2**	0.994 (0.874)	0.998 (0.884)	0.996 (0.762)
**CC***	0.999 (0.966)	1 (0.969)	0.999 (0.93)
**Reflections used in refinement**	241139 (20976)	146068 (14443)	106576 (10783)
**Reflections used for R-free**	2525 (220)	7314 (724)	1120 (114)
**R-work**	0.1576 (0.1987)	0.1598 (0.2432)	0.1669 (0.2632)
**R-free**	0.1756 (0.2419)	0.1856 (0.2673)	0.1835 (0.3150)
**CC(work)**	0.963 (0.911)	0.971 (0.911)	0.962 (0.878)
**CC(free)**	0.961 (0.890)	0.951 (0.888)	0.960 (0.844)
**Number of non-hydrogen atoms**	6730	6589	6679
**macromolecules**	5963	5883	5909
**ligands**	129	93	101
**solvent**	638	613	669
**Protein residues**	733	728	725
**RMS(bonds)**	0.006	0.005	0.006
**RMS(angles)**	0.85	0.82	0.87
**Ramachandran favored (%)**	97.24	97.5	97.35
**Ramachandran allowed (%)**	2.76	2.5	2.65
**Ramachandran outliers (%)**	0	0	0
**Rotamer outliers (%)**	1.42	1.76	2.07
**Clashscore**	3.22	5.64	2.18
**Average B-factor**	20.03	23.85	22.36
**macromolecules**	18.88	22.19	21.18
**ligands**	20.94	22.71	20.56
**solvent**	30.6	39.87	33.06
**PDB deposition**	8AUN	8AUQ	8AUO

Structures of wild-type enzymes and variants

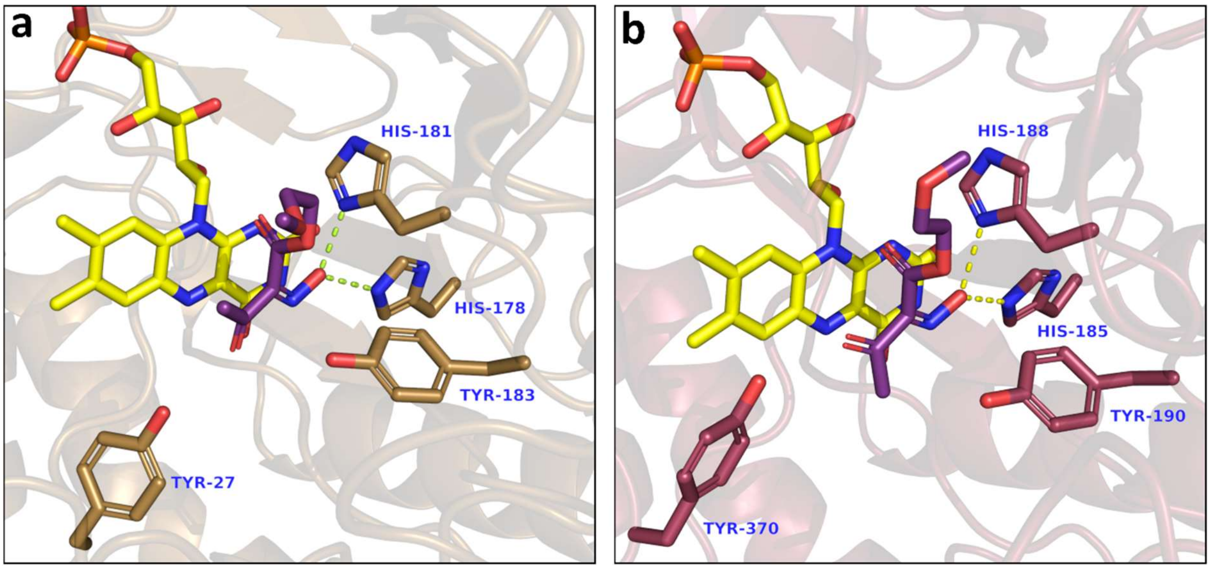

Figure S1: XenA_wt (a) and OPR3_wt (b) in complex with oxime 1c

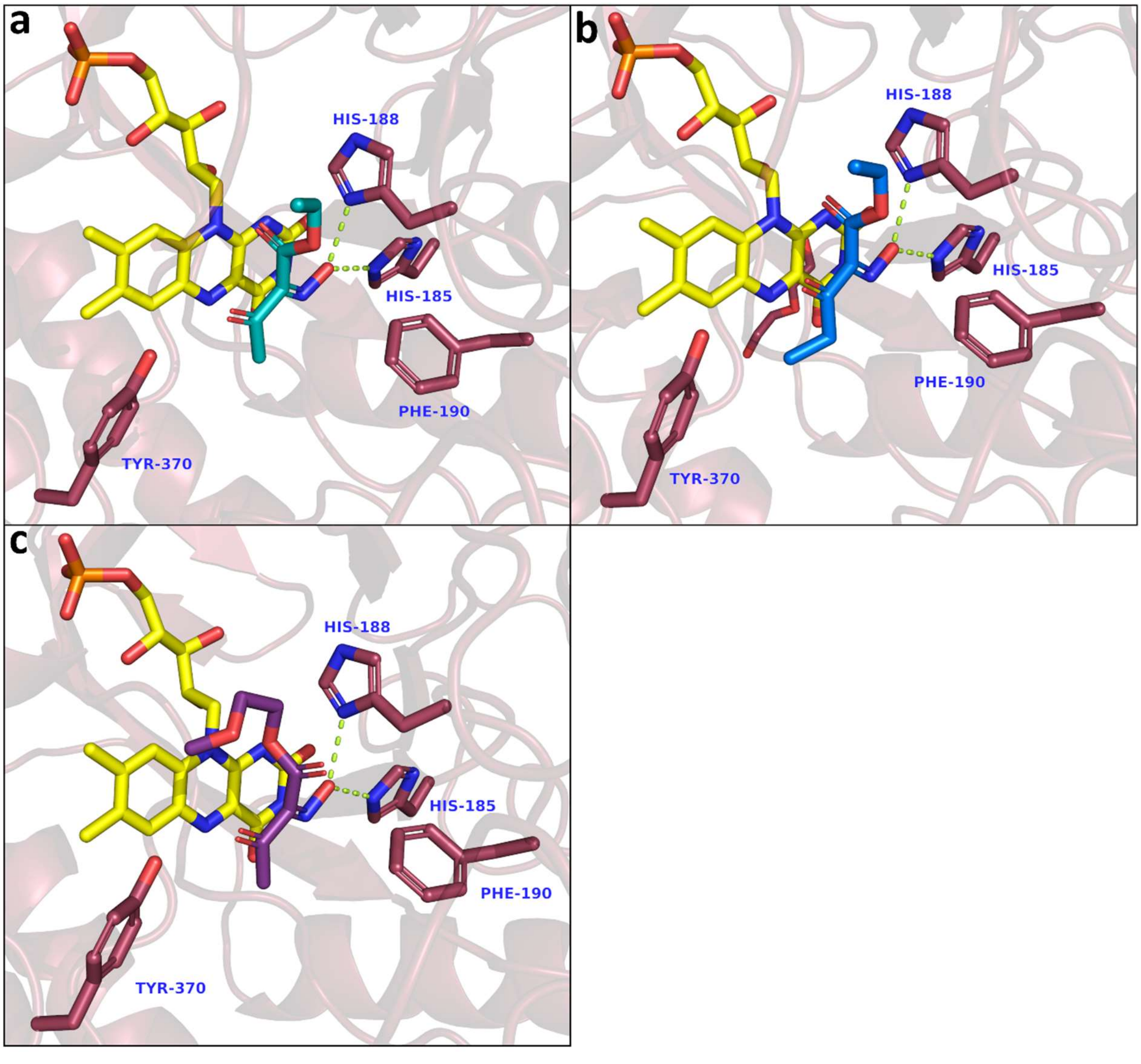

Figure S2: OPR3_Y190F in complex with a) oxime 1a, b) oxime 1b, and c) oxime 1c

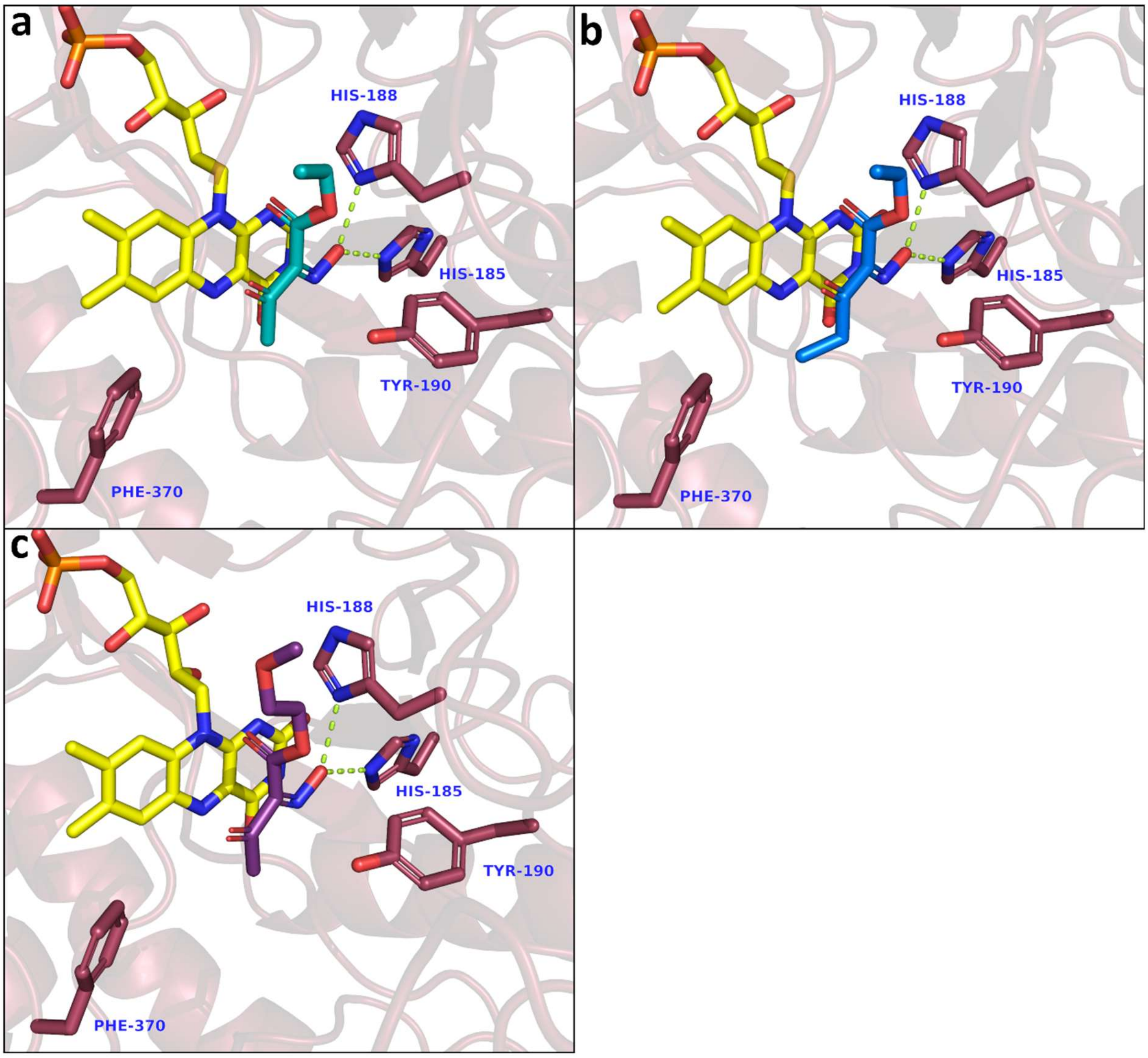

Figure S3: OPR3_Y370F in complex with a) oxime 1a, b) oxime 1b, and c) oxime 1c

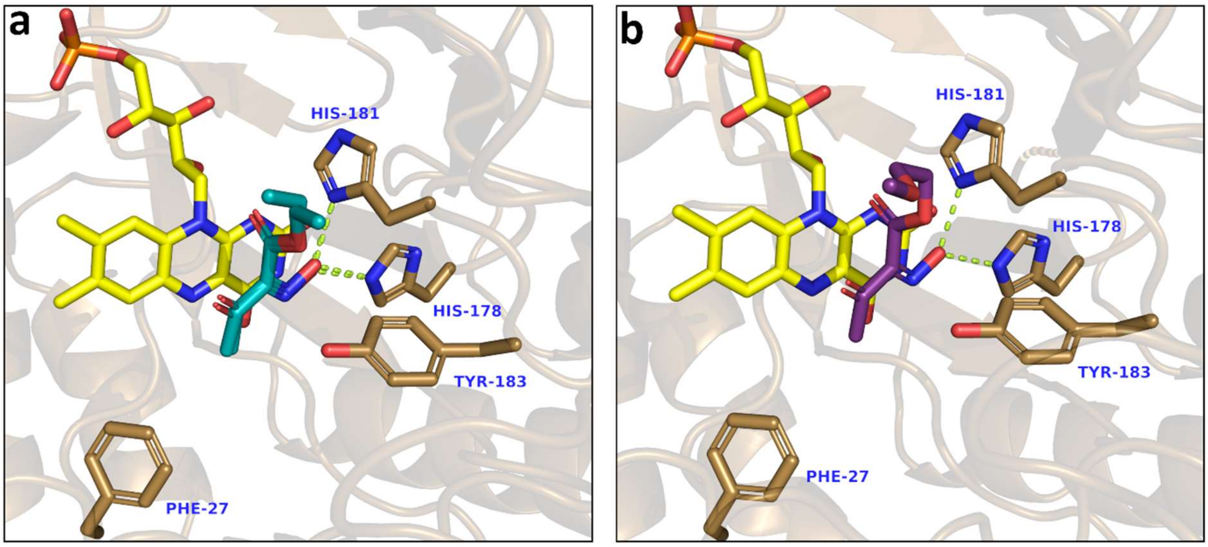

*Figure S4: XenA_Y27F in complex with a) oxime **1a** and b) oxime **1c***

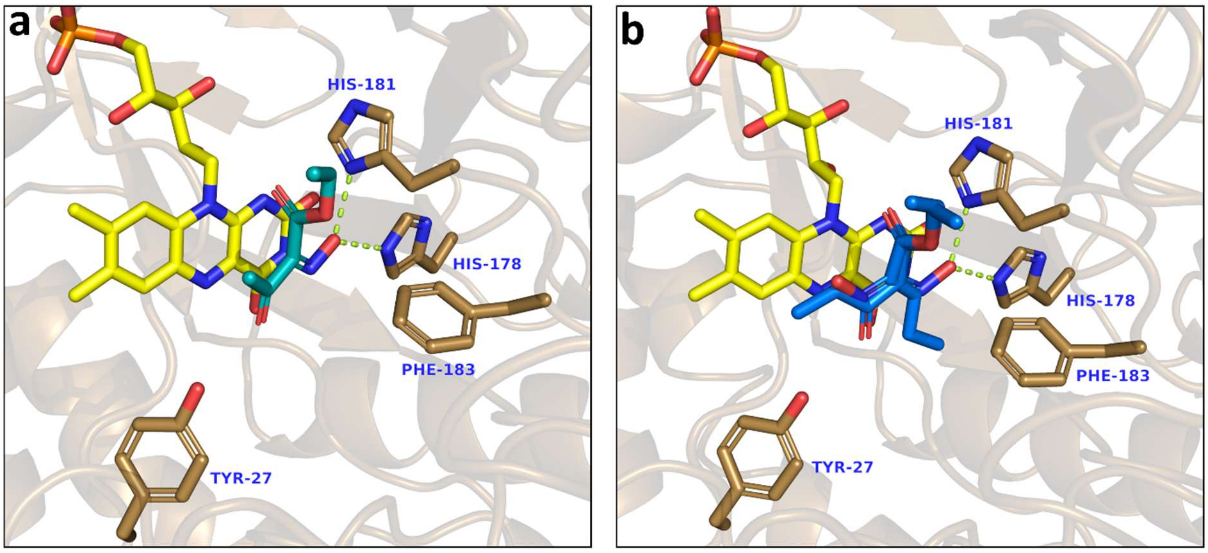

*Figure S5: XenA_Y183F in complex with a) oxime **1a** and b) oxime **1b***

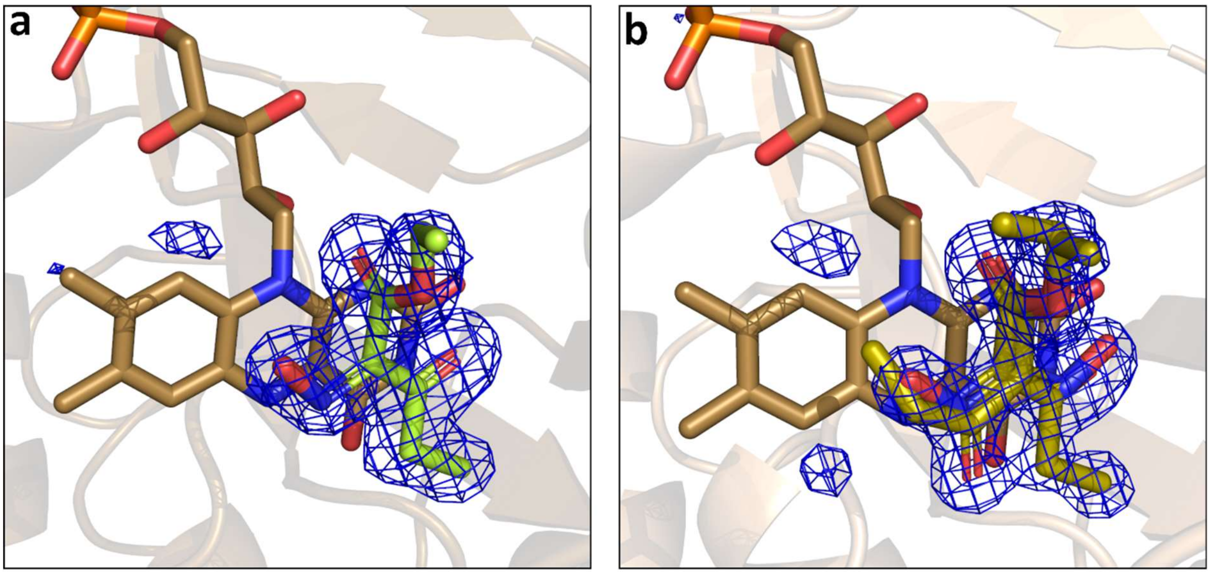

*Figure S6: Polder map of oxime **1b** in XenA_wt (a) and XenA_Y183F (b)*

Comparison with previously deposited structures

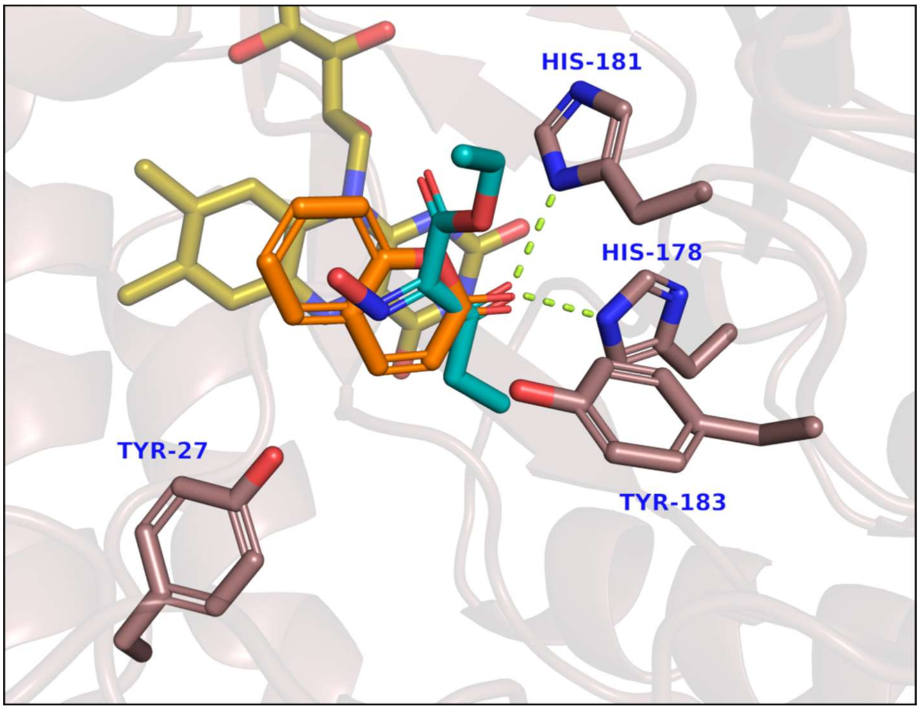

Figure S7: Binding mode of oxime 1b in XenA_wt compared to the one of coumarin. The structure of XenA in complex with coumarin can be found under the PDB code 2H90.

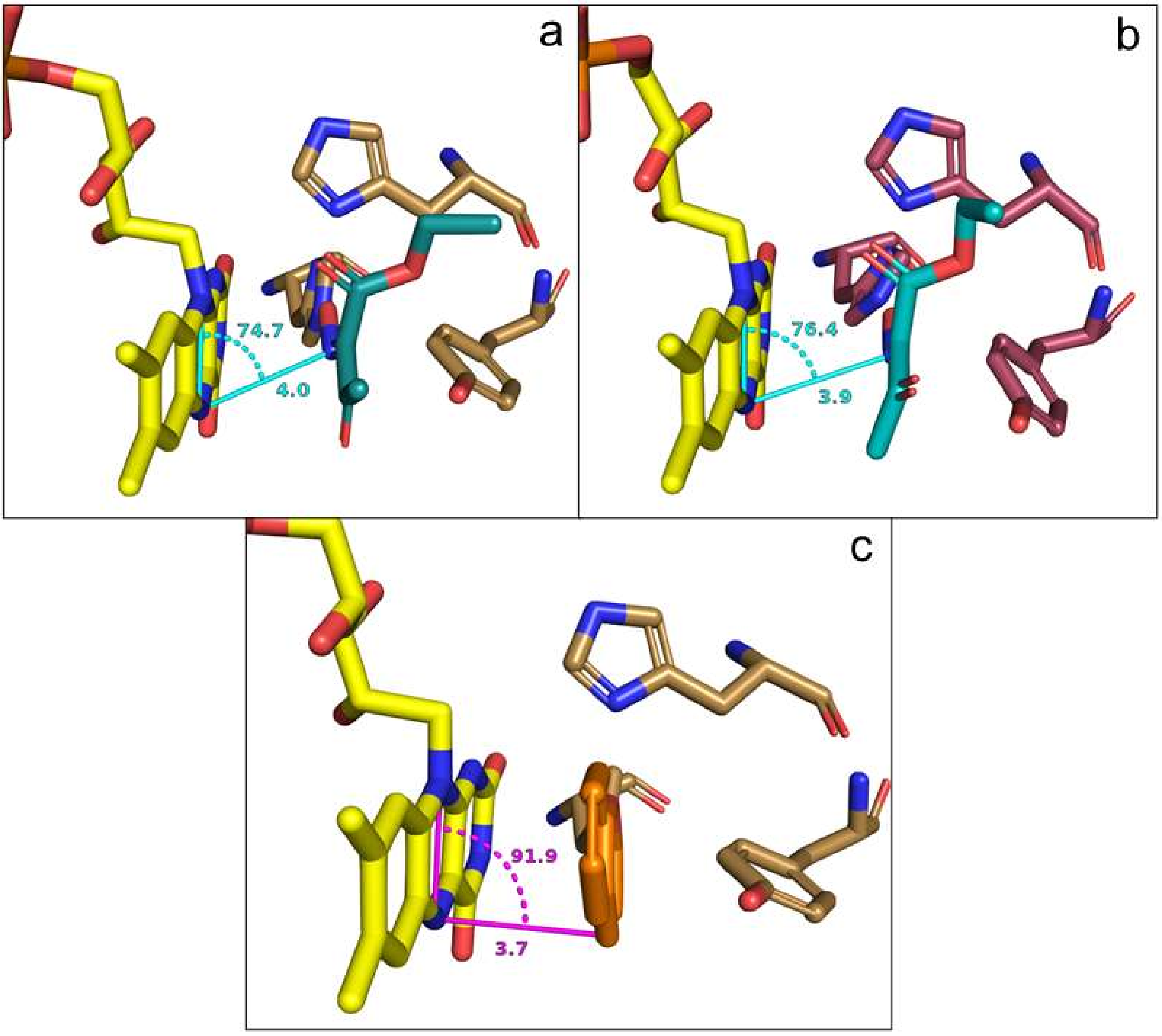

*Figure S8: Angle formed between the flavin N10, N5 and the hydride receiving atom of the oxime **1a** in XenA_wt (a) and OPR3_wt (b). c) XenA_wt in complex with coumarin (PDB: 2H90) as a representation of a productive angle and distance for hydride transfer*

Molecular dynamics simulations

NAC-MD Simulations

The crystal structures were used as input and further prepared using the protein preparation wizard in the Maestro program, with careful emphasis on the protonation state of active site histidines (His-181, His-183 in OPR3, and His-178 and His-181 in XenA). Each system then undergoes an energy minimization step only for the H atoms, followed by solvation with a buffer of at least 10 Å surrounding the protein and neutralization by adding a respective number of ions.

For equilibration, we ran 50 ps NVT at 10 K and 50 ps NVT at 300 K, followed by 100 ps NPT at 300 K with restraining of solute-heavy atoms. Finally, we ran a short 10 ps without any restrains at 300 K. The equilibrated system was then simulated with 10 independent production runs of 1 ns at 300 K. Desmond[18] MD program has been used with OPLS4[19] all-atom potential. A time step of 1 fs was used throughout the simulations, along with Nose–Hoover thermostat and Martyna–Tobias–Klein barostat, with relaxation times of 1.0 and 2.0 ps, respectively. The particle mesh Ewald method was used to treat long-range interactions, and a non-bonded cutoff of 9.0 Å was used for short-range interactions. A different random seed was used to generate the initial velocities for each of the 10 independent 1 ns production runs.

Near attack conformations (NACs) represents subpopulations of substrate that approach the configuration of the relevant transition state, often defined in terms of distances and angles. Figure S9 shows the characterization of NAC for our productive binding mode based on the typical OYE catalysis.[20,21]

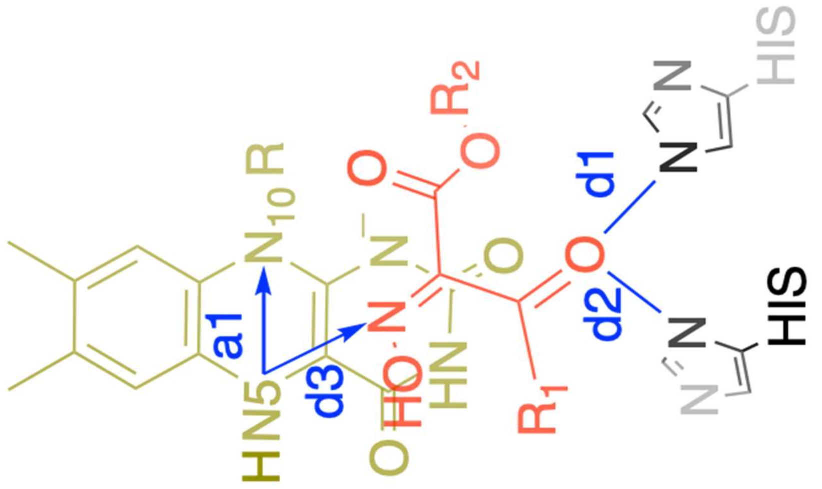

Figure S9: Near Attack Conformation (NAC) parameters where d1 and d2 are the distance vector between the carbonyl oxygen and N atom of respective histidine residues, and d3 is another distance vector between the hydride bearing N5 atom of flavin to hydride acceptor N of the oxime, whereas a1 represents the angle between the N1, N5 of flavins to N of the oxime. A NAC is defined as existing if d1 or d2 should be ≤ 3.5 Å and d3 should be ≤ 4.0 Å, and a1 should be between 85 to 110 degrees. Oxime is shown in red, flavin in green, and active site histidines in black, whereas the NAC parameters are coloured in blue. This figure should not be considered for interpreting the 3D representation of the oxime binding mode. This has been drawn for a clearer explanation of NAC parameters.

The described geometrical parameters for NAC were averaged over 10 independent production runs of 1 ns. Transfer of the H nucleus in enzymatic reactions is a sub-picosecond timescale process.[22] Considering this, we deemed 1 ns of simulation enough to represent the hydride transfer reaction time, and our 10 independent replicas, each with a different random velocity, ensure the unbiased sampling of each possible state on the hydride transfer landscape.

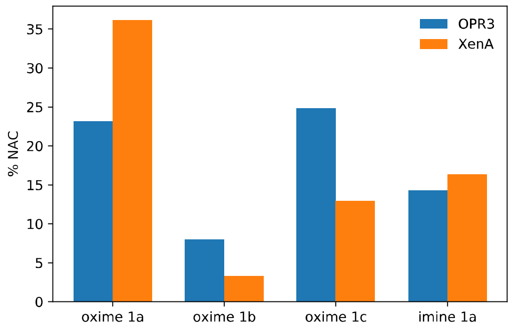

Figure S10: Near Attack Conformation (NAC) analysis of 10 independent MD simulations of 1 ns each

Binding Pose Metadynamics (BPMD)

Productive and non-productive binding poses of oxime **1h** in complex with reduced XenA were used as the starting structures for BPMD simulations.

The BPMD protocol was developed to assess the stability of ligands in a binding pocket. It is a variant of metadynamics simulations, where the root-mean-square deviation (RMSD) of bound ligand heavy atoms is a measure of its stability in the binding pocket. A loose binding pose will experience a higher RMSD compared to lower RMSD fluctuations for a tightly bound pose of the same ligand in a binding site. Using ligand heavy atoms' RMSD as a collective variable (CV), 10 independent metadynamics simulations were performed, each of 10 ns. The alignment before the RMSD calculation was done by selecting Cαs of the protein residues within 3 Å of the ligand.

To compare the binding poses, two scores were derived from the BPMD simulations: firstly, PoseScore is the average RMSD from the starting pose. A higher PoseScore with a steep increase from the starting pose describes a poorly bound ligand pose and vice versa. Secondly, PersScore is a fraction of how often the original number of hydrogen bonds (in the first frame) persists in the last 2 ns of BPMD simulations. PersScore ranges from 0 to 1, where 0 indicates that either the starting ligand pose did not have any hydrogen bond interactions with the target or that the hydrogen bond interactions have been lost during the simulations, while 1 indicates that the hydrogen bonds between the staring ligand pose and the last 2 ns of the simulations have been retained. The default protocol[23] available with the Schrödinger program was used to run BPMD simulations.

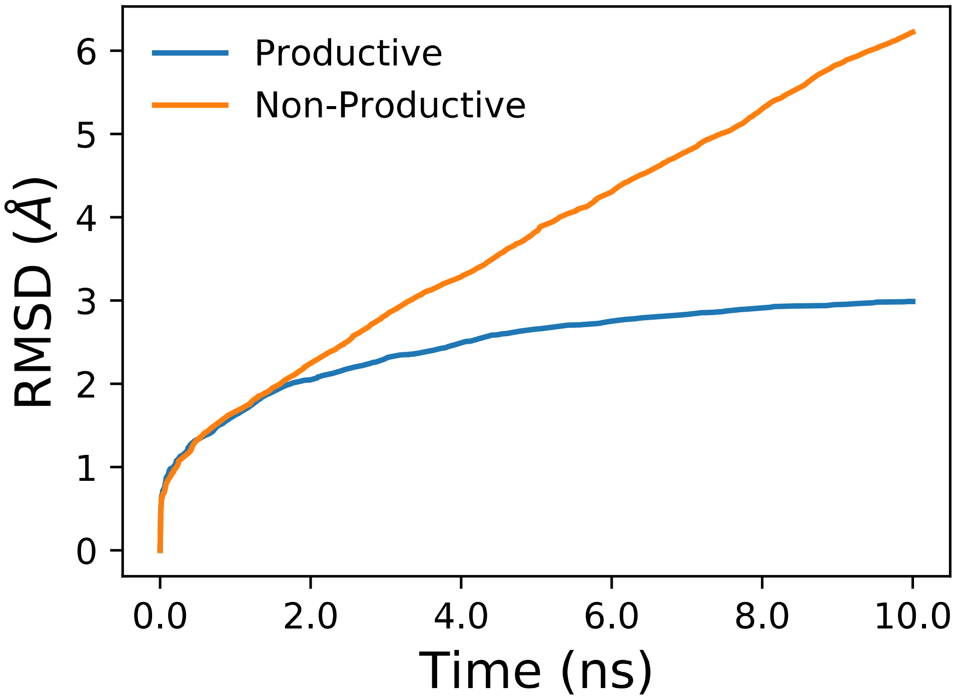

Figure S11: Binding pose metadynamics analysis. 2.95 and 5.8 are the PoseScore values for productive and non-productive binding modes, respectively. The PersScore for the productive binding mode is 0.10, whereas it is zero for the non-productive binding mode.

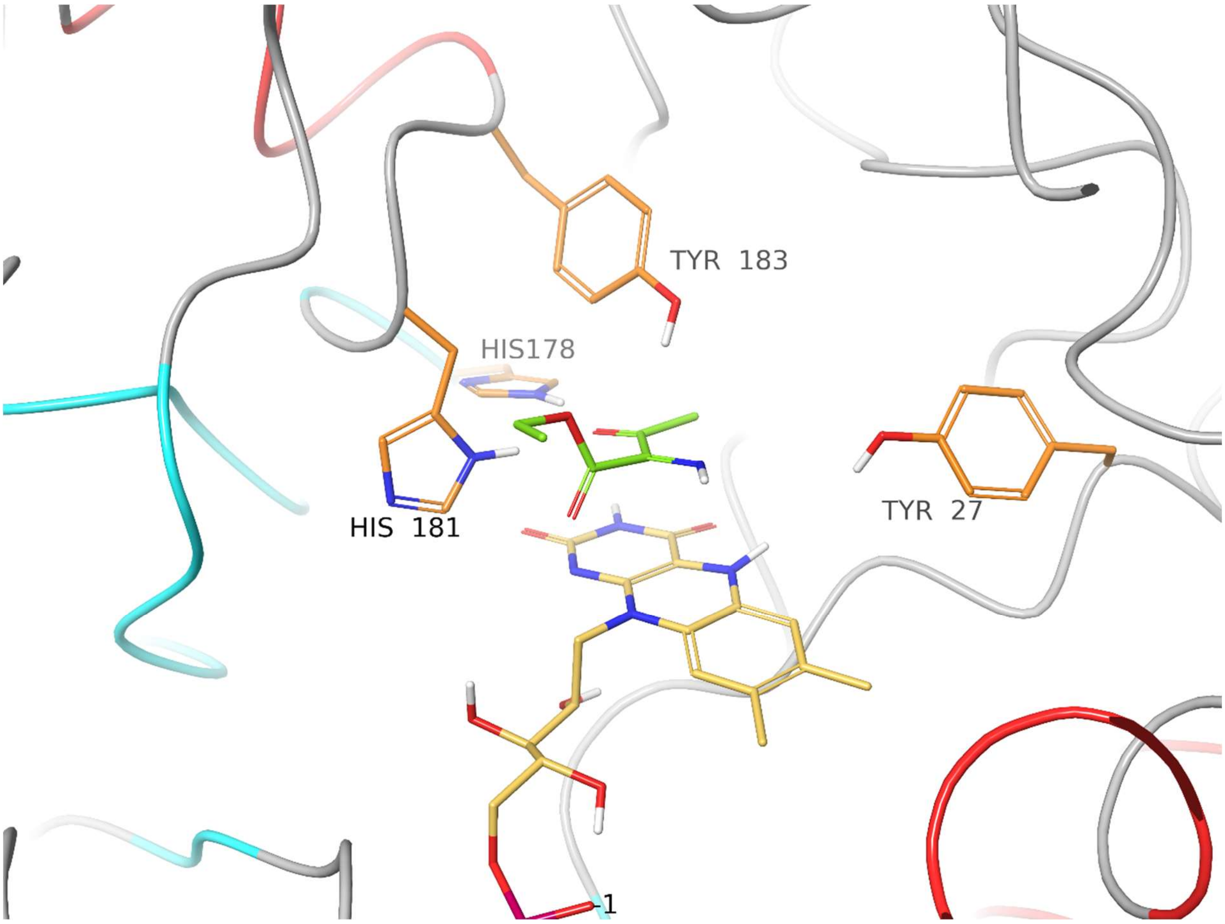

Figure S12: Modeled binding pose for imine 5a in the active site of XenA. The original crystal structure of XenA complexed with oxime 1h was used as a template. Imine 5a is shown in green, reduced flavin in yellow, and the oxime binding site residues in orange. The

Biocatalysis

Analytical scale biotransformations

All analytical scale biotransformations were carried out on 500 µL scale. Unless specifically stated otherwise, "buffer" refers to 50 mM Na/K phosphate buffer (pH 7.5).

<u>General procedure A: Oxime to pyrazine</u>

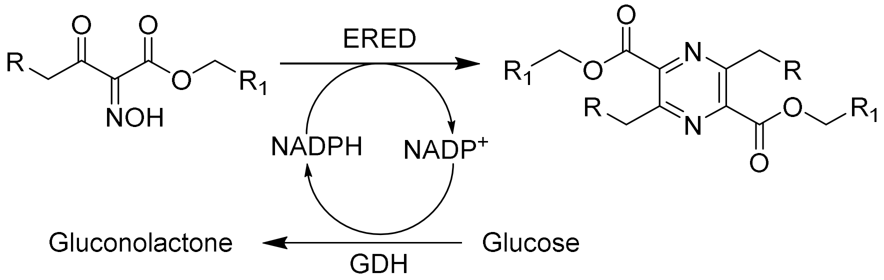

Scheme S12: Reaction equation for general procedure A

Table S19: Reaction composition for general procedure A

Component	*Final concentration*	*Stock concentration*	*Volume added (µL)*	*Stock dissolved in*
Oxime substrate	10 mM	200 mM	25	DMSO
Purified ene-reductase	0.2 mg/mL	4 mg/mL (for most enzymes)	25 (for most enzymes)	Buffer
NADPH	0.5 mM	10 mM	25	Buffer
D-glucose	50 mM	1 M	25	Buffer
GDH (crude CFE)	4 mg/mL	8 mg/mL	250	Buffer
DMSO	5 % v/v	n/a	25	
Phosphate buffer 50 mM pH 7.5	To adjust volume to 500 µL	n/a	175 (for most reactions)	

The glucose dehydrogenase (GDH, 250 µL of an 8 mg/mL solution in buffer; final concentration 4 mg/mL) was diluted with buffer (volume required to give a total volume of 0.5 mL after addition of all other reagents) in a 1.5 mL microcentrifuge tube. Then, the ene-reductase (volume required to give 0.2 mg/mL final concentration; in buffer; volume varies based on concentration of stock) was added, followed by NADPH (25 µL of a 10.0 mM solution in buffer; final concentration 0.5 mM) and D-glucose (25 µL of a 1.0 M solution in buffer; final concentration 50 mM) in indiscriminate order, followed by the oxime substrate (always added last, 25 µL of a 200 mM solution in DMSO; final concentration 10 mM). The reaction mixtures were shaken in an orbital shaker in vertical position for 24 hours at 120 rpm and 30 °C. Then, the reaction mixtures were extracted with EtOAc containing 10 mM tetramethylpyrazine as internal standard (2x 500 µL), dried over Na_2SO_4, transferred to 1.5 mL glass vials and analyzed on GC-FID.
For qualitative analysis, the reactions mixtures were extracted with EtOAc without internal standard (2x500 µL), dried over Na_2SO_4, transferred to 1.5 mL glass vials and analyzed on GC-MS.
Details on analytics for each individual compound can be found in the analytics section.

General procedure B: Cyclohexenone to cyclohexanone

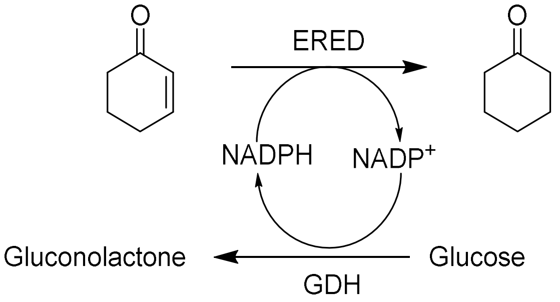

Scheme S13: Reaction equation for general procedure B

Table S20: Reaction composition for general procedure B

Component	*Final concentration*	*Stock concentration*	*Volume added (µL)*	*Stock dissolved in*
Cyclohexenone	50 mM	1.0 M	25	DMSO
Purified ene-reductase	0.2 mg/mL	4 mg/mL (for most enzymes)	25 (for most enzymes)	Buffer
NADPH	0.5 mM	10 mM	25	Buffer
D-glucose	50 mM	1 M	25	Buffer
GDH (crude CFE)	4 mg/mL	8 mg/mL	250	Buffer
DMSO	5 % v/v	n/a	25	
Phosphate buffer 50 mM pH 7.5	To adjust volume to 500 µL	n/a	175 (for most reactions)	

The glucose dehydrogenase (GDH, 250 µL of an 8 mg/mL solution buffer; final concentration 4 mg/mL) was diluted with buffer (volume required to give a total volume of 0.5 mL after addition of all other reagents) in a 1.5 mL microcentrifuge tube. Then, the ene-reductase (volume required to give 0.2 mg/mL final concentration; in buffer; volume varies based on concentration of stock) was added, followed by NADPH (25 µL of a 10.0 mM solution in buffer; final concentration 0.5 mM) and D-glucose (25 µL of a 1.0 M solution in buffer; final concentration 50 mM) in indiscriminate order, followed by the cyclohexenone (always added last, 25 µL of a 1.0 M solution in DMSO; final concentration 50 mM). The reaction mixtures were shaken in an orbital shaker in vertical position for 24 hours at 120 rpm and 30 °C. Then, the reaction mixtures were extracted with EtOAc containing 10 mM tetramethylpyrazine as internal standard (2x 500 µL), dried over Na_2SO_4, transferred to 1.5 mL glass vials and analyzed on GC-FID. Details on analytics for each individual compound can be found in the analytics section.

General procedure C: Oxime to amidoalcohol

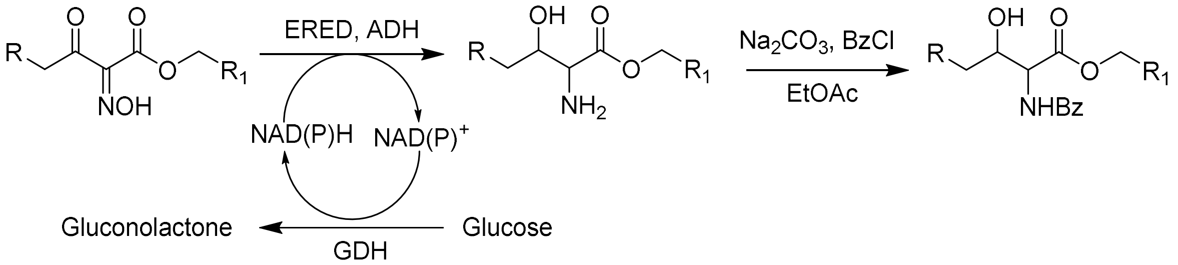

Scheme S14: Reaction equation for general procedure C

Table S21: Reaction composition for general procedure C

Component	*Final concentration*	*Stock concentration*	*Volume added (µL)*	*Stock dissolved in*
Oxime	10 mM	200 mM	25	DMSO
Purified ene-reductase	0.2 mg/mL	4 mg/mL (for most enzymes)	25 (for most enzymes)	Buffer
ADH (CFE)	varies	varies	100 µL	Buffer
NADPH	0.5 mM	10 mM	25	Buffer
NADH	0.5 mM	10 mM	25	Buffer
D-glucose	50 mM	1 M	25	Buffer
GDH (crude CFE)	4 mg/mL	8 mg/mL	250	Buffer
DMSO	5 % v/v	n/a	25	
Phosphate buffer 50 mM pH 7.5	To adjust volume to 500 µL	n/a	150 (for most reactions)	

The glucose dehydrogenase (GDH, 250 µL of an 8 mg/mL solution in buffer; final concentration 4 mg/mL) was diluted with buffer (volume required to give a total volume of 0.5 mL after addition of all other reagents) in a 1.5 mL microcentrifuge tube. Then, the ene-reductase (volume required to give 0.2 mg/mL final concentration; in buffer, pH 7.5; volume varies based on concentration of stock) was added, followed by the ADH (usually added at this stage as 100 µL of a five-fold stock solution in buffer, sometimes as a solid, weighed directly into the microcentrifuge tube before addition of the GDH and buffer solutions). Then, NADPH (25 µL of a 10.0 mM solution in buffer; final concentration 0.5 mM), NADH (only added for reactions with ADH-A, RasADH and SYADH; 25 µL of a 10.0 mM solution in buffer; final concentration 0.5 mM) and D-glucose (25 µL of a 1.0 M solution in buffer; final concentration 50 mM) were added in indiscriminate order, followed by the oxime substrate (always added last, 25 µL of a 200 mM solution in DMSO; final concentration 10 mM). The reaction mixtures were shaken in an orbital shaker in vertical position for 24 hours at 120 rpm and 30 °C. The compounds were derivatised by addition of Na_2CO_3 (sat aq, 20 µL), followed by benzoyl chloride (500 µL of a 30 mM solution in EtOAc containing 10 mM internal standard (acetanilide for oximes **1a** and **1c**, benzanilide for oxime **1b**), final concentration of BzCl: 15 mM, 3 eq) shaken in an orbital shaker in vertical position for 1 hour at 120 rpm and 30 °C, centrifuged, the layers separated and the aqueous layer extracted once more with EtOAc (containing 10 mM internal standard, 500 µL) and the combined organic layers were dried over Na_2SO_4, transferred to 1.5 mL glass vial and analyzed on normal-phase HPLC with chiral stationary phase. Details on analytics for each individual compound can be found in the analytics section.

Preparative scale biotransformations

*Deuterium incorporation test of aminoketone **2a***

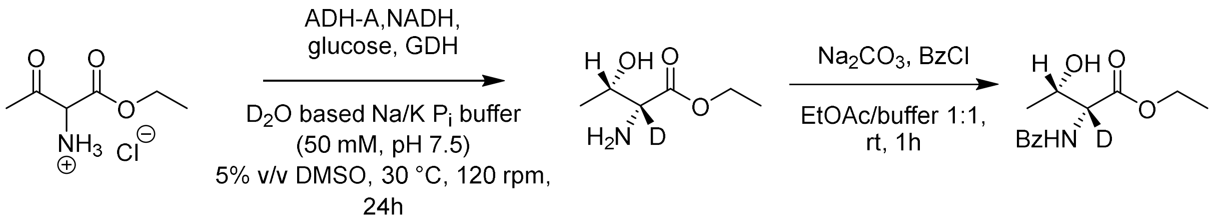

$Na_2HPO_4 \cdot 2H_2O$ (720 mg) and KH_2PO_4 (96 mg) were dissolved in D_2O (99.8 atom% D; 95 mL) to give 50 mM Na/K P_i buffer (pH 7.5). ADH-A (heat-treated CFE, 200 mg; 2 mg/mL final concentration), NADH (35.5 mg; 0.05 mmol; final concentration 0.5 mM), NADPH (41.7 mg; 0.05 mmol; 0.5 mM final concentration), D-glucose (901 mg; 5.0 mmol; final concentration 50 mM) and GDH (200 mg crude CFE; final concentration 2 mg/mL) were added and dissolved by gently swirling the flask. The amine hydrochloride (**2a**·HCl; 182 mg; 1.0 mmol; final concentration 10 mM, 5 mL of a 200 mM solution in DMSO) was added and the reaction was shaken in an orbital shaker for 24 hours (120 rpm, 30 °C). Then, the reaction mixture was transferred to a 500 mL round-bottom flask, the pH was adjusted to 9 using Na_2CO_3 (sat aq) and benzoyl chloride (348 µL; 3.0 mmol; 3.0 eq, dissolved in 100 mL EtOAc) was added, and the reaction was stirred vigorously for one hour. The mixture was centrifuged (8000 rpm, 4 °C, 10 min), after which the aqueous layer was saturated with sodium chloride and diethyl ether (30 mL) was added. The layers were separated and the aqueous phase extracted with EtOAc/Et_2O 3:1 (3x 130 mL). The combined organic layers were dried over Na_2SO_4, filtered and solvent was removed under reduced pressure (40 °C). The crude material (approx. 730 mg of a yellow oil) was purified by column chromatography (SiO_2, EtOAc/cyclohexane, 6 to 65% EtOAc) to give ethyl *N*-benzoyl D-threoninate-2-d in 32% isolated yield (80 mg; 0.32 mmol).

TLC R_f (SiO_2, EtOAc 1:1) 0.31.

The absolute configuration was determined to be >99% 2*R*, 3*S* using chiral stationary phase HPLC-UV as described in the analytics section for the biotransformations yielding ethyl *N*-benzoyl threoninates.

^{1}H NMR (300 MHz, Chloroform-d) δ 7.91 – 7.81 (m, 2H), 7.56 – 7.48 (m, 1H), 7.48 – 7.37 (m, 2H), 7.09 (s, 1H), 4.44 (q, J = 6.4 Hz, 1H), 4.24 (q, J = 7.1 Hz, 2H), 2.94 (s, 1H), 1.30 (t, J = 7.0 Hz, 6H).

Comparison of the ^{1}H NMR spectrum with that of synthetic (non-labeled) ethyl *N*-benzoyl-D-threoninate[1] clearly shows deuteration in only the α position.

*Cascade biotransformation from oxime **1a***

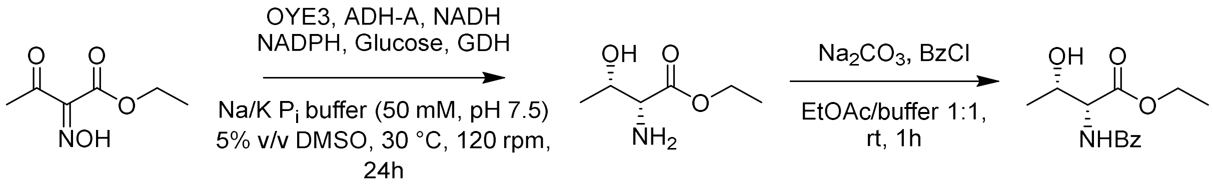

OYE3 (10 mL of a 4.0 mg/mL solution in Na/K P_i buffer, pH 7.5; final concentration 0.2 mg/mL) was added to buffer (Na/K P_i, pH 7.5, 180 mL) in a 500 mL Erlenmeyer flask. ADH-A (heat-treated CFE; 400 mg; final concentration 2 mg/mL) and GDH (crude CFE; 400 mg; final concentration 2 mg/mL) were added. NADPH (41.7 mg; 0.05 mmol; 0.5 mM final concentration), NADH (71 mg; 0.1 mmol; final concentration 0.5 mM) and D-glucose (1.8 g; 10.0 mmol; final concentration 50 mM) were added and dissolved by gently swirling the flask. The oxime (318.3 mg; 2.0 mmol; final concentration 10 mM, 10 mL of a 200 mM solution in DMSO) was added and the reaction was shaken in an orbital shaker for 24

hours (120 rpm, 30 °C). Then, the reaction mixture was transferred to a 1 L round-bottom flask, the pH was adjusted to 9 using Na_2CO_3 (sat aq) and benzoyl chloride (697 µL; 6.0 mmol; 3.0 eq, dissolved in 100 mL EtOAc) was added, and the reaction was stirred vigorously for one hour. The layers were separated by filtration through a pad of celite (approx. 2 cm). The aqueous phase was extracted with EtOAc (3x 100 ml). The combined organic layers were dried over Na_2SO_4, filtered and solvent was removed under reduced pressure (40 °C). The crude material (approx. 1.3 g of a yellow oil) was purified by column chromatography (SiO_2, EtOAc/cyclohexane, 5 to 65% EtOAc) to give ethyl benzoyl-*D*-threoninate in 41% isolated yield (205 mg; 0.82 mmol).

TLC R_f (SiO_2, EtOAc 1:2) 0.1.

The absolute configuration was determined to be >99% 2*R*,3*S* using chiral stationary phase HPLC-UV as described in the analytics section for the cascade biotransformations from oxime **1a**.

^{1}H NMR (300 MHz, Chloroform-d) δ 7.86 (dt, J = 7.0, 1.5 Hz, 2H), 7.59 – 7.49 (m, 1H), 7.44 (dd, J = 8.2, 6.5 Hz, 2H), 7.07 (d, J = 8.8 Hz, 1H), 4.82 (dd, J = 8.8, 2.5 Hz, 1H), 4.46 (qd, J = 6.4, 2.5 Hz, 1H), 4.26 (q, J = 7.2 Hz, 2H), 1.38 – 1.22 (m, 6H).

The data matches that of the chemically synthesised product.[1]

*Cascade biotransformation from oxime **1b***

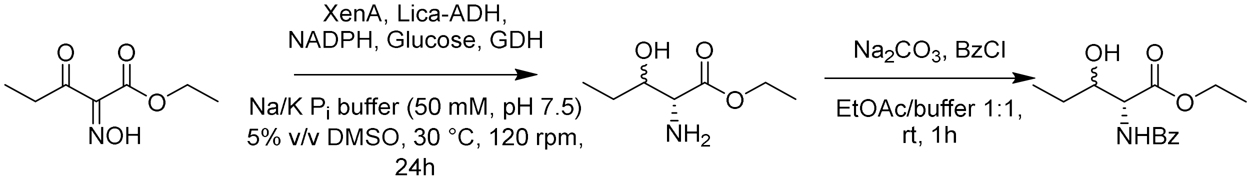

XenA (10 mL of a 4.0 mg/mL solution in Na/K P_i buffer, pH 7.5; final concentration 0.2 mg/mL) was added to buffer (Na/K P_i, pH 7.5, 180 mL) in a 500 mL Erlenmeyer flask. *Lk*ADH-Lica (crude CFE; 1.0 g; final concentration 5 mg/mL) and GDH (crude CFE; 400 mg; final concentration 2 mg/mL) were added. NADPH (41.7 mg; 0.05 mmol; 0.5 mM final concentration) and D-glucose (1.8 g; 10.0 mmol; final concentration 50 mM) were added and dissolved by gently swirling the flask. The oxime (346.3 mg; 2.0 mmol; final concentration 10 mM, 10 mL of a 200 mM solution in DMSO) was added and the reaction was shaken in an orbital shaker for 24 hours (120 rpm, 30 °C). Then, the reaction mixture was transferred to a 1 L round-bottom flask, the pH was adjusted to 9 using Na_2CO_3 (sat aq) and benzoyl chloride (697 µL; 6.0 mmol; 3.0 eq, dissolved in 100 mL EtOAc) was added, and the reaction was stirred vigorously for one hour. The layers were separated by filtration through a pad of celite (approx. 2 cm). The aqueous phase was extracted with EtOAc (3x 100 ml). The combined organic layers were dried over Na_2SO_4, filtered and solvent was removed under reduced pressure (40 °C). The crude material (approx. 1 g of a yellow oil) was purified by column chromatography (SiO_2, EtOAc/cyclohexane, 7 to 70% EtOAc) to give ethyl (2*R*)-2-benzamido-3-hydroxypentanoate in 57% isolated yield (305 mg; 1.15 mmol).

TLC R_f (SiO_2, EtOAc/cyclohexane 1:3) 0.25.

The absolute configuration was determined to be 68:32 2*R*, 3*S*/2*S*,3*S* using chiral stationary phase HPLC-UV as described in the analytics section for the cascade biotransformations from oxime **1b**.

^{1}H NMR (300 MHz, Chloroform-d) δ 7.92 – 7.79 (m, 2H), 7.62 – 7.39 (m, 3H), 7.22 (d, J = 7.1 Hz, 1H), 6.99 (d, J = 8.9 Hz, 0H), 4.91 (td, J = 8.0, 7.2, 2.6 Hz, 1H), 4.36 – 4.21 (m, 2H), 4.21 – 4.11 (m, 0H), 4.00 (ddd, J = 7.7, 5.8, 3.1 Hz, 1H), 1.70 – 1.49 (m, 2H), 1.33 (q, J = 7.2 Hz, 3H), 1.04 (td, J = 7.4, 2.3 Hz, 3H).

The data matches with that of the chemically synthesised product (see section on synthesis of references).

*Cascade biotransformation from oxime **1c***

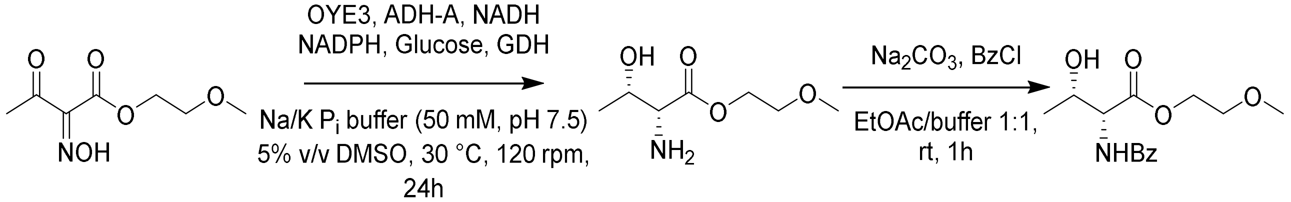

OYE3 (10 mL of a 4.0 mg/mL solution in Na/K P_i buffer, pH 7.5; final concentration 0.2 mg/mL) was added to buffer (Na/K P_i, pH 7.5, 180 mL) in a 500 mL Erlenmeyer flask. ADH-A (heat-treated CFE; 400 mg; final concentration 2 mg/mL) and GDH (crude CFE; 400 mg; final concentration 2 mg/mL) were added. NADPH (41.7 mg; 0.05 mmol; 0.5 mM final concentration), NADH (71 mg; 0.1 mmol; final concentration 0.5 mM) and D-glucose (1.8 g; 10.0 mmol; final concentration 50 mM) were added and dissolved by gently swirling the flask. The oxime (378.3 mg; 2.0 mmol; final concentration 10 mM, 10 mL of a 200 mM solution in DMSO) was added and the reaction was shaken in an orbital shaker for 24 hours (120 rpm, 30 °C). Then, the reaction mixture was transferred to a 1 L round-bottom flask, the pH was adjusted to 9 using Na_2CO_3 (sat aq) and benzoyl chloride (697 µL; 6.0 mmol; 3.0 eq, dissolved in 100 mL EtOAc) was added, and the reaction was stirred vigorously for one hour. The layers were separated by filtration through a pad of celite (approx. 2 cm). The aqueous phase was extracted with EtOAc (3x 100 ml). The combined organic layers were dried over Na_2SO_4, filtered and solvent was removed under reduced pressure (40 °C). The crude material (approx. 840 g of a yellow oil) was purified by column chromatography (SiO_2, EtOAc/cyclohexane, 8 to 80% EtOAc) to give 2-methoxyethyl benzoyl-*D*-threoninate in 27% isolated yield (154 mg; 0.55 mmol).

TLC R_f (SiO_2, EtOAc/cyclohexane 1:1) 0.1.

The absolute configuration was determined to be 97:2:1 D/D-allo/L using chiral stationary phase HPLC-UV as described in the analytics section for the cascade biotransformations from oxime 1C.

^{1}H NMR (300 MHz, Chloroform-d) δ 7.92 – 7.83 (m, 2H), 7.56 – 7.46 (m, 1H), 7.41 (td, J = 8.0, 7.0, 2.2 Hz, 2H), 7.20 (d, J = 9.0 Hz, 1H), 4.88 (dd, J = 9.0, 2.4 Hz, 1H), 4.55 – 4.37 (m, 2H), 4.28 (ddd, J = 12.1, 5.3, 3.7 Hz, 1H), 3.62 (ddd, J = 5.5, 3.6, 2.0 Hz, 3H), 3.37 (s, 3H), 1.27 (d, J = 6.5 Hz, 3H).

The data matches with that of chemically synthesised 2-methoxyethyl benzoyl *D*-treonine, but this product contains some benzoic acid (could not be quantified due to overlapping peaks in ^{1}H NMR) that co-eluted during column chromatography.

Stop-flow experiments

The pre-steady state kinetics were collected using a stopped-flow device (SF-61DX2, TgK Scientific, UK) at 25 °C under anoxic conditions in a glove box (Belle Technology, Weymouth, UK). Oxygen was removed from the buffers by flushing with nitrogen and subsequent incubation in the glove box overnight. Oxygen was removed from the samples by incubation in the glove box for 30 minutes and periodical mixing. Spectral changes of the flavin cofactor were followed at a wavelength of 450 nm using a KinetaScanT diode array detector (MG-6560, Hi-Tech) employing the Kinetic Studio software (TgK Scientific, UK).

For the reductive rates, 35 µM of enzyme was shot against different concentrations of NADPH. For the oxidative rates, the same amount of enzyme was pre-reduced using a sub-stoichiometrical amount of NADPH and shot against different concentrations of oxime **1a** (OPR3) or oxime **1b** (XenA). In both the cases, the absorbance signal at 450 nm was used to determine the rates. K_D values were calculated by a non-linear least-squares fit that was performed using the function 'curve_fit' from the optimize-module of the scipy-package in python. Standard Michealis-Menten type kinetics was used as model.

Synthesis of substrates

Oximes **1a**, **1b** and **1c** were synthesised as reported previously.[1]

*Synthesis of ethyl 2-amino-3-oxobutanoate hydrochloride (**2a**·HCl)*

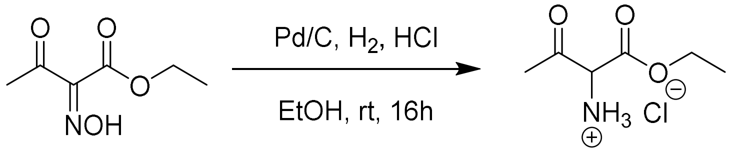

10 % Pd/C (178 mg; 0.167 mmol Pd; 3.34 mol%) was added to a solution of the oxime (796 mg; 5.0 mmol; 1.0 eq) in 15 mL of anhydrous ethanol. After degassing the suspension by bubbling with H_2 and placing the reaction under hydrogen atmosphere (balloon), HCl (1.25 M in EtOH; 20 mL; 25 mmol; 5 eq) was added dropwise. The suspension was stirred at room temperature overnight. Then the balloon was removed, the suspension degassed with Ar to remove HCl and the solvent evaporated under reduced pressure (40 °C) to give an orange oil. The amine hydrochloride was precipitated with cold Et_2O (dry, degassed, 20 mL) by first ultrasonicating and then stirring the suspension. The crude amine hydrochloride was obtained in 72% yield (656 mg; 3.59 mmol) as brown solids.

^{1}H NMR (300 MHz, Methanol-d4) δ 4.41 (q, J = 7.1 Hz, 2H), 1.48 – 1.28 (m, 6H).

^{13}C NMR (75 MHz, Methanol-d4) δ 195.69, 163.25, 63.45, 26.73, 12.89 (d, J = 6.7 Hz).

ESI-MS (positive mode): calculated 146.08 for [M+H], found 146.1.

*Synthesis of ethyl 2-amino-3-oxopentanoate hydrochloride (**2b**·HCl)*

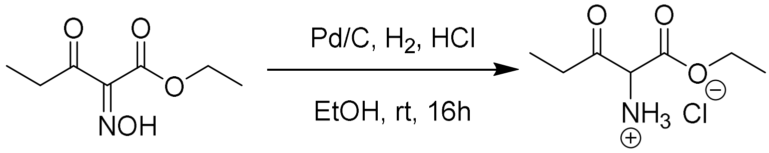

10 % Pd/C (106 mg; 0.1 mmol Pd; 3.34 mol%) was added to a solution of the oxime (533 mg; 3.0 mmol; 1.0 eq) in 9 mL of ethanol. After degassing the suspension with H_2 and placing the reaction under hydrogen atmpshere (balloon), HCl (1.25 M in EtOH; 12 mL; 15 mmol; 5 eq) was added dropwise. The suspension was stirred at room temperature overnight. Then the balloon was removed, the suspension degassed with Ar to remove HCl and the solvent evaporated under reduced pressure (40 °C) to give an orange oil. The amine hydrochloride was precipitated with cold anhydrous Et_2O (20 mL) by first ultrasonicating and then stirring the suspension. The crude amine hydrochloride was obtained in quantitative yield (610 mg; 3.0 mmol) as brown solids.

^{1}H NMR (300 MHz, Deuterium Oxide) δ 4.28 (q, J = 7.2 Hz, 2H), 2.92 (dq, J = 19.2, 7.2 Hz, 1H), 2.74 (dq, J = 19.1, 7.1 Hz, 1H), 1.24 (t, J = 7.2 Hz, 3H), 0.98 (t, J = 7.2 Hz, 3H).

^{13}C NMR (75 MHz, Deuterium Oxide) δ 201.11, 164.01, 64.63, 33.93, 13.04, 6.58.

ESI-MS (positive mode): calculated 160.10 for [M+H], found 160.2.

*Synthesis of 1-(2-methoxyethoxy)-1,3-dioxobutan-2-aminium chloride (**2c**·HCl)*

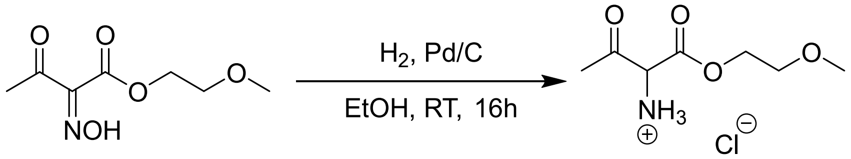

Palladium on charcoal (10% Pd basis; 178 mg; 0.167 mmol Pd; 3.33 mol% Pd) was added to a solution of the oxime (946 mg; 5.0 mmol; 1.0 eq) in absolute EtOH (12 mL). HCl (1.25 M in EtOH; 12 mL: 15.0 mmol; 3.0 eq) was added dropwise and the reaction was placed under hydrogen atmosphere (balloon). After stirring the reaction under hydrogen atmosphere overnight, the suspension was filtered and the solvent was removed *in vacuo* to yield the product in quantitative yield (1.07 g) as a brown oil in decent purity (containing some residual ethanol).

^{1}H NMR (300 MHz, Methanol-d_4) δ 4.63 – 4.27 (m, 4H), 3.75 – 3.67 (m, 3H), 3.39 (d, *J* = 1.7 Hz, 4H), 2.51 (s, 3H).

^{13}C NMR (75 MHz, Methanol-d_4) δ 195.6, 69.5, 65.7, 57.64, 57.0, 26.6, 17.0.

*Synthesis of ethyl 2-(methoxyimino)-3-oxobutanoate (**7a**)*

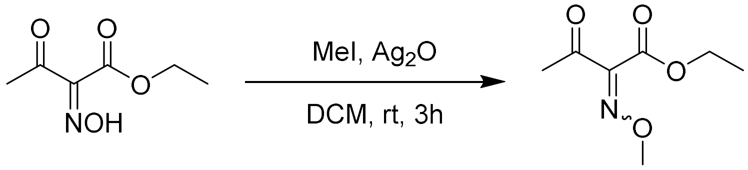

The procedure was based on one reported in literature.[24] Silver (I) oxide (2.55 g; 11 mmol; 1.1 eq) was added in portions over 30 minutes to a solution of oxime **1a** (1.59 g; 10 mmol; 1.0 eq) and methyl iodide (3.12 mL; 50 mmol; 5.0 eq) in DCM (20 mL) at room temperature. Three hours after completion of addition of the silver (I) oxide, TLC analysis indicated full consumption of the starting material. The suspension was filtered and the filtrate was concentrated *in vacuo*. The crude product was purified by column chromatography (SiO_2; EtOAc/cyclohexane, gradient of 0 to 13% EtOAc) to yield the desired products as colorless oils in 55% combined yield.

Z-***7a***:
Yield: 35% (612 mg; 3.53 mmol).

TLC R_f (SiO_2, EtOAc/cyclohexane 1:9) 0.38 (UV-254 nm).

^{1}H NMR (300 MHz, Chloroform-d) δ 4.37 (q, J = 7.2 Hz, 2H), 4.12 (s, 3H), 2.42 (s, 3H), 1.36 (t, J = 7.1 Hz, 3H).

^{13}C NMR (75 MHz, Chloroform-*d*) δ 192.87, 161.18, 150.08, 64.38, 62.13, 25.16, 14.02.

E-**7a**:
Yield: 20% (337 mg; 1.95 mmol).

TLC R_f (SiO_2, EtOAc/cyclohexane 1:9) 0.20 (UV-254 nm).

^{1}H NMR (300 MHz, Chloroform-d) δ 4.37 (q, J = 7.1 Hz, 2H), 4.10 (s, 3H), 2.41 (s, 3H), 1.36 (t, J = 7.1 Hz, 3H).

^{13}C NMR (75 MHz, Chloroform-*d*) δ 196.54, 160.09, 149.96, 64.26, 62.47, 30.14, 14.05.

The values for both isomers match those reported.[24]

E and *Z* isomers were assigned based on the chemical shift of the Cγ as described in literature.[24]

*Synthesis of ethyl 2-(methoxyamino)-3-oxobutanoate (**8a**)*

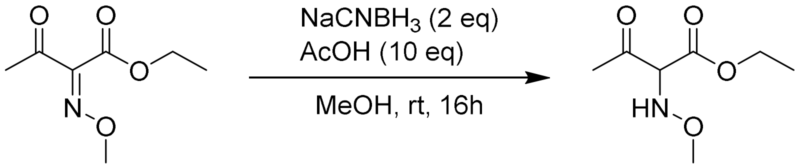

Z-**7a**

Acetic acid (0.94 mL; 16.43 mmol; 20 eq) was added to a solution of the *Z*-**7a** (286 mg; 1.65 mmol; 1.0 eq) in methanol (8.7 mL). Sodium cyanoborohydride (207 mg; 3.3 mmol; 2.0 eq) was added in one portion (gas evolution) and the mixture was stirred at room temperature overnight. Then, the solvent was removed *in vacuo* and water (15 mL) was added to the residue. The pH of the solution was adjusted to 9.0 using Na_2CO_3 (sat aq). The solution was extracted with EtOAc (3x 20 mL), the combined organic layers dried over Na_2SO_4, filtered and solvent removed under reduced pressure (40 °C). The crude product was purified by column chromatography (SiO_2, EtOAc/cyclohexane, gradient of 3 to 35% EtOAc) to yield the desired compound in 63% yield (182 mg; 1.04 mmol) as a colorless oil.

TLC R_f (SiO_2, EtOAc/cyclohexane 1:3) 0.33 (UV-254 nm).

^{1}H NMR (300 MHz, Chloroform-d) δ 4.60 (q, J = 6.4 Hz, 1H), 4.35 (qd, J = 7.2, 2.9 Hz, 2H), 3.92 (d, J = 3.5 Hz, 3H), 2.73 – 2.41 (m, 1H), 1.43 (dd, J = 6.6, 2.5 Hz, 3H), 1.36 (td, J = 7.1, 2.6 Hz, 3H).

^{13}C NMR (75 MHz, Chloroform-d) δ 162.37, 153.06, 66.73, 62.77, 61.78, 20.78, 14.13.

This compound has not previously been reported in literature.

ESI-MS (positive mode): calculated 176.09 for [M+H], found 176.1.

*Synthesis of ethyl 2-(methoxyimino)-3-oxopentanoate (**7b**)*

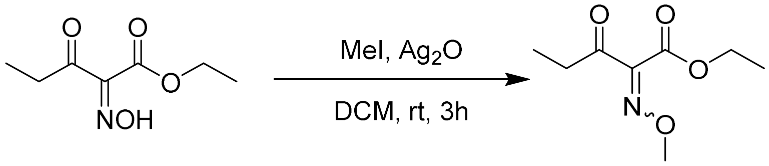

The procedure was based on one reported in literature.[24] Silver (I) oxide (2.55 g; 11 mmol; 1.1 eq) was added in portions over 30 minutes to a solution of oxime **1b** (1.73 g; 10 mmol; 1.0 eq) and methyl iodide (3.12 mL; 50 mmol; 5.0 eq) in DCM (20 mL) at room temperature. Thirty minutes after completion of addition of the silver (I) oxide, TLC analysis indicated near full consumption of the starting material. The suspension was filtered and the filtrate was concentrated *in vacuo*. The crude product was purified by column chromatography (SiO_2; EtOAc/cyclohexane, gradient of 1 to 18% EtOAc) to yield the desired products as colorless oils in 77% combined yield.

<u>*Z*-**7b**:</u>
Yield: 46% (854 mg; 4.56 mmol).

TLC R_f (SiO_2, EtOAc/cyclohexane 1:6) 0.45 (UV-254 nm).

^{1}H NMR (300 MHz, Chloroform-d) δ 4.36 (q, J = 7.1 Hz, 2H), 4.10 (s, 3H), 2.83 (q, J = 7.4 Hz, 2H), 1.35 (t, J = 7.2 Hz, 3H), 1.14 (t, J = 7.3 Hz, 3H).

^{13}C NMR (75 MHz, Chloroform-d) δ 195.76, 161.30, 149.51, 64.27, 62.07, 30.92, 14.05, 7.49.

The values match those reported in literature.[25]

<u>*E*-**7b**:</u>
Yield: 31% (582 mg; 3.11 mmol).

TLC R_f (SiO_2, EtOAc/cyclohexane 1:6) 0.34 (UV-254 nm).

^{1}H NMR (300 MHz, Chloroform-d) δ 4.42 – 4.26 (q, 2H), 4.07 (s, Hz, 3H), 2.74 – 2.57 (q, 2H), 1.35 (t, J = 7.2 Hz, 3H), 1.20 – 1.10 (t, 3H).

^{13}C NMR (75 MHz, Chloroform-d) δ 199.91, 160.25, 150.20, 64.17, 62.41, 36.05, 14.05, 6.62.

The values match those reported in literature.[26]

E and *Z* isomers were assigned based on the chemical shift of the Cγ as described in literature.[24]

*Synthesis of ethyl 2-(methoxyamino)-3-oxopentanoate (**8b**)*

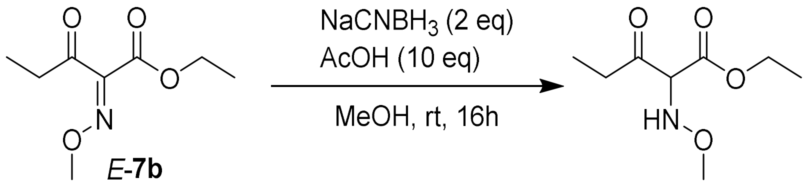

Acetic acid (1.72 mL; 30.0 mmol; 20 eq) was added to a solution of *E*-**7b** (562 mg; 3.0 mmol; 1.0 eq) in methanol (15 mL). Sodium cyanoborohydride (377 mg; 6.0 mmol; 2.0 eq) was added in one portion (gas evolution) and the mixture was stirred at room temperature overnight. Then, the solvent was removed *in vacuo* and water (30 mL) was added to the residue. The pH of the solution was adjusted to 9.0 using Na_2CO_3 (sat aq). The solution was extracted with EtOAc (3x 30 mL), the combined organic layers dried over Na_2SO_4, filtered and solvent removed under reduced pressure (40 °C). The crude product was purified by column chromatography (SiO_2, EtOAc/cyclohexane, gradient of 3 to 40% EtOAc) to yield the desired compound in 76% yield (429 mg; 2.27 mmol) as a colorless oil.

TLC (SiO_2, EtOAc/cyclohexane 1:3) R_f 0.36.

^{1}H NMR (300 MHz, Chloroform-d) δ 4.50 – 4.24 (m, 3H), 3.91 (s, 3H), 2.55 – 2.35 (m, 1H), 1.89 – 1.57 (m, 2H), 1.34 (t, J = 7.1 Hz, 3H), 1.00 (t, J = 7.4 Hz, 3H).

^{13}C NMR (75 MHz, Chloroform-d) δ 162.37, 152.38, 71.78, 62.81, 61.77, 27.69, 14.14, 9.17.

^{1}H-^{1}H COSY and HSQC spectra were also recorded (see Appendix A). The compound has not previously been reported in literature.

ESI-MS (positive mode): calculated 190.11 for [M+H], found 190.2.

*Synthesis of 2-methoxyethyl 3-hydroxy-2-(hydroxyimino)butanoate (**S1**)*

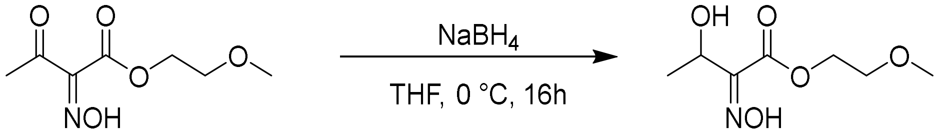

Sodium borohydride (227 mg; 6.0 mmol; 2.0 eq) was added in one portion to a solution of the oxime (576.5 mg; 3.0 mmol; 1.0 eq) in THF (15 mL) at 0 °C (gas evolution). TLC analysis (SiO_2, EtOAc 1:1, R_f product 0.21) indicated full consumption of the starting material within 30 minutes. While still at 0 °C, the reaction was quenched by addition of water (5 mL), followed by NH_4Cl (sat aq, 15 mL). EtOAc (10 mL) was added and the layers were separated. The aqueous phase was extracted with EtOAc (3x 25 mL). The combined organic layers dried over Na_2SO_4, filtered and solvent removed under reduced pressure (40 °C). The crude product (approx. 400 mg) was purified by column chromatography (SiO_2, EtOAc/cyclohexane, gradient of 5 to 75% EtOAc) to yield the desired compound in 19% yield (107 mg; 0.56 mmol) as a colorless oil.

TLC R_f (SiO_2, EtOAc/cyclohexane 1:1) 0.21 (UV-254 nm).

^1H NMR (300 MHz, Acetonitrile-d3) δ 9.82 (s, 0H), 9.19 (s, 1H), 4.47 (q, J = 6.6 Hz, 1H), 4.38 – 4.22 (m, 2H), 3.65 – 3.53 (m, 2H), 3.41 (s, 1H), 3.32 (d, J = 2.7 Hz, 3H), 2.33 – 2.08 (m, 1H), 1.39 (d, J = 6.8 Hz, 1H), 1.32 (d, J = 6.6 Hz, 2H).

^{13}C NMR (75 MHz, Acetonitrile-d3) δ 163.06, 154.79, 69.78, 66.05, 64.15 (d, J = 5.0 Hz), 61.89, 57.98, 20.11, 19.94.

ESI-MS (positive mode): calculated 192.09 for [M+H], found 192.1.

*Synthesis of ethyl 3-hydroxy-2-(hydroxyimino)pentanoate (**S2**)*

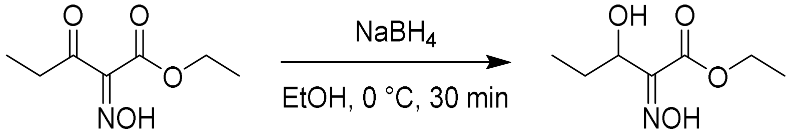

A solution of the ketone (346 mg; 2.0 mmol; 1.0 eq) in absolute ethanol (10 mL) was cooled to 0 °C, after which sodium borohydride (151 mg; 4.0 mmol; 2.0 eq) was added in one portion. TLC analysis after 30 minutes at 0 °C (SiO_2, EtOAc/cy 1:2) showed full conversion of the starting material, and the reaction was quenched by addition of ammonium chloride (sat aq, 10 mL). The mixture was then extracted with EtOAc (3x 20 mL), the combined organic layers dried over Na_2SO_4, filtered and solvent was removed under reduced pressure (40 °C). The crude was purified by column chromatography (SiO_2, EtOAc/cy, 6 to 50% EtOAc) to yield the alcohol in 33% yield (114 mg; 0.65 mmol).

TLC R_f (SiO_2, EtOAc/cy 1:2) 0.28.

^1H NMR (300 MHz, Chloroform-d) δ 4.94 (dd, J = 8.2, 5.9 Hz, 1H), 4.40 – 4.27 (m, 2H), 1.98 – 1.68 (m, 2H), 1.36 (t, J = 7.1 Hz, 3H), 1.01 (t, J = 7.5 Hz, 3H).

^{13}C NMR (75 MHz, Chloroform-d) δ 163.17, 152.57, 68.34, 62.13, 28.10, 13.96, 9.82.

Synthesis of reference compounds

Pyrazine reference products were synthesised enzymatically as reported previously.[1]

Racemic reference products for cascade biotransformations on oxime 1b

Synthesis of N-*benzoyl glycine ethyl ester (**S3**)*

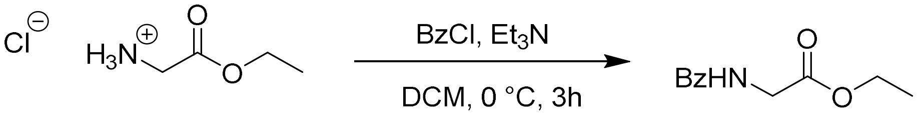

This reaction was performed following an adapted reported procedure.[27] A solution of glycine ethyl ester hydrochloride (2.79 g; 20 mmol; 1.0 eq) in DCM (HPLC grade, 50 mL) was cooled to 0 °C before careful addition of triethylamine (6.3 mL; 45 mmol; 2.25 eq). A thick suspension formed, and DCM (50 mL) was added. Benzoyl chloride (2.8 mL: 24 mmol; 1.2 eq) was added dropwise and the reaction was stirred at 0 °C for three hours, when TLC analysis (SiO_2, EtOAc/cy 1:1) showed significant product formation. The mixture was washed with water (2x 50 mL), sodium bicarbonate (sat. aq., 50 mL) and brine (50 mL) and the organic layer was concentrated under reduced pressure (40 °C). The crude product was purified by column chromatography (SiO_2, EtOAc/cy, 5 to 50% EtOAc) to yield the desired product as a white solid (3.79 g; 18.31 mmol; 92%).

TLC R_f (SiO_2, EtOAc/cyclohexane 1:2) 0.27 (UV-254 nm).

^{1}H NMR (300 MHz, Chloroform-d) δ 7.88 – 7.79 (m, 2H), 7.61 – 7.41 (m, 3H), 6.73 (s, 1H), 4.51 – 4.13 (m, 4H), 1.33 (t, J = 7.1 Hz, 3H).

The values match with those reported.[28]

*Synthesis of ethyl 2-benzamido-3-oxopentanoate (**S4**)*

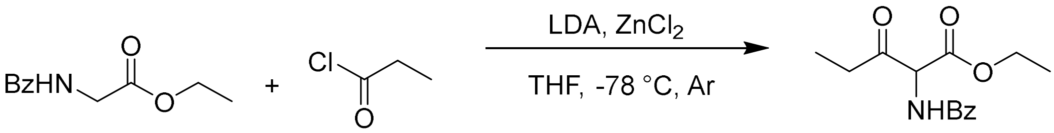

This reaction was performed following a reported procedure.[29] A flamed out round bottom flask with stir bar under argon atmosphere was charged with *N*-benzoyl glycine ethyl ester (622 mg; 3.0 mmol; 1.0 eq), and zinc chloride (491 mg; 3.6 mmol; 1.2 eq) and anhydrous THF (20 mL). After cooling the resulting solution to -78 °C, lithium diisopropylamide (1 M in THF/hexanes, 9.0 mL; 9.0 mmol; 3.0 eq) was carefully added, resulting in a deep orange solution. After stirring the reaction for 90 minutes at -78 °C, propanoyl chloride (288 µL; 3.3 mmol; 1.1 eq) was added and the mixture was stirred for another 90 minutes, during which the temperature rose to -60 °C. The reaction was quenched by addition of ammonium chloride (sat aq, 30 mL), which gave significant gas formation, and precipitated solids. After addition of water (20 mL), the two distinct layers were clear. The mixture was extracted with diethyl ether (3x 30 mL), the combined organic layers dried over Na_2SO_4, filtered and solvent was removed under reduced pressure (40 °C). The crude was purified by column chromatography (SiO_2, EtOAc/cy, 3 to 30% EtOAc) to give the mostly pure product (457 mg; 1.74 mmol; 58%). GC-MS analysis indicated presence of minor amounts of diisopropylamine and *N,N*-diisopropylpropionamide.

TLC R_f (SiO_2, EtOAc/cyclohexane 1:1) 0.75 (UV-254 nm, $KMnO_4$).

^{1}H NMR (300 MHz, Chloroform-d) δ 7.90 – 7.82 (m, 2H), 7.60 – 7.51 (m, 1H), 7.51 – 7.42 (m, 2H), 7.41 – 7.30 (m, 1H), 5.45 (d, J = 6.5 Hz, 1H), 4.31 (q, J = 7.1 Hz, 2H), 3.02 – 2.69 (m, 2H), 2.33 (q, J = 7.4 Hz, 0H), 1.33 (t, J = 7.1 Hz, 3H), 1.16 (t, J = 7.3 Hz, 4H).

^{13}C NMR (75 MHz, Chloroform-d) δ 201.82, 166.81, 166.34, 133.05, 132.09, 128.66, 127.25, 62.69 (d, J = 1.3 Hz), 34.45, 26.91, 14.05, 7.53.

The values match with those reported.[30]

*Synthesis of racemic ethyl 2-benzamido-3-hydroxypentanoate (**N-Bz-4b**)*

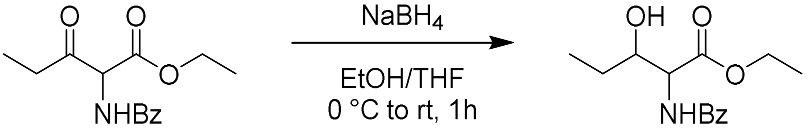

This reaction was performed following an adapted reported procedure.[31] The ß-keto ester (432 mg; 1.64 mmol; 1.0 eq) was dissolved in THF/EtOH (1:1, 5.5 mL) and cooled to 0 °C. Then, $NaBH_4$ (62 mg; 1.64 mmol; 1.0 eq) was added in one portion and the reaction was stirred at 0 °C for thirty minutes, after which TLC analysis (SiO_2, EtOAc/cy 1:2) showed complete conversion of the ketone. Water (5 mL) was added and the solvent removed under reduced pressure (40 °C). Approximately 20 mL of EtOAc was added to the residue, which was then washed with brine (2x 15 mL). The organic layer was dried over Na_2SO_4, filtered and solvent was removed under reduced pressure (40 °C) to yield 388 mg of crude product. The crude was purified by column chromatography (SiO_2, EtOAc/cyclohexane, 5 to 100% EtOAc) to yield the desired product in 28% yield (123 mg; 0.46 mmol).

TLC R_f (SiO_2, EtOAc/cyclohexane 1:2) 0.25 (UV-254 nm).

^{1}H NMR (700 MHz, Chloroform-d) δ 7.84 – 7.78 (m, 2H), 7.52 – 7.47 (m, 1H), 7.43 – 7.38 (m, 2H), 7.24 (d, J = 7.2 Hz, 1H), 7.04 (d, J = 8.9 Hz, 0H), 4.86 (ddd, J = 20.6, 8.1, 2.7 Hz, 1H), 4.29 – 4.19 (m, 2H), 3.95 (ddd, J = 8.3, 5.6, 3.2 Hz, 1H), 3.66 – 3.47 (m, 1H), 1.61 – 1.51 (m, 2H), 1.29 (t, J = 7.2 Hz, 3H), 1.00 (t, J = 7.5 Hz, 3H).

^{13}C NMR (176 MHz, Chloroform-d) δ 171.49, 170.52, 167.91, 133.89, 133.41, 132.00, 131.78, 128.62, 128.54, 127.24, 74.78, 73.76, 61.95, 57.91, 56.21, 27.10, 26.51, 14.17 (d, J = 5.7 Hz), 10.33, 10.13. (Signals for both diastereomers are noted).

Analysis of the two sets of doublets of doublets at 4.90-4.80 ppm (the proton at Cα) shows a 82:18 ratio of diastereomers.

ESI-MS (positive mode): calculated 266.14 for [M+H], found 266.1.

HPLC-UV analytics (Daicel Chiralpak IC column, n-heptane:2-PrOH 90:10, flow rate 1 mL/min, column oven 30 °C, 30 min) shows four distinct peaks with retention times of 11.50 min, 14.04 min, 16.8 min and 20.4 min. As shown in Figure S13, the first two peaks are enantiomers of each other, as are the second two peaks.

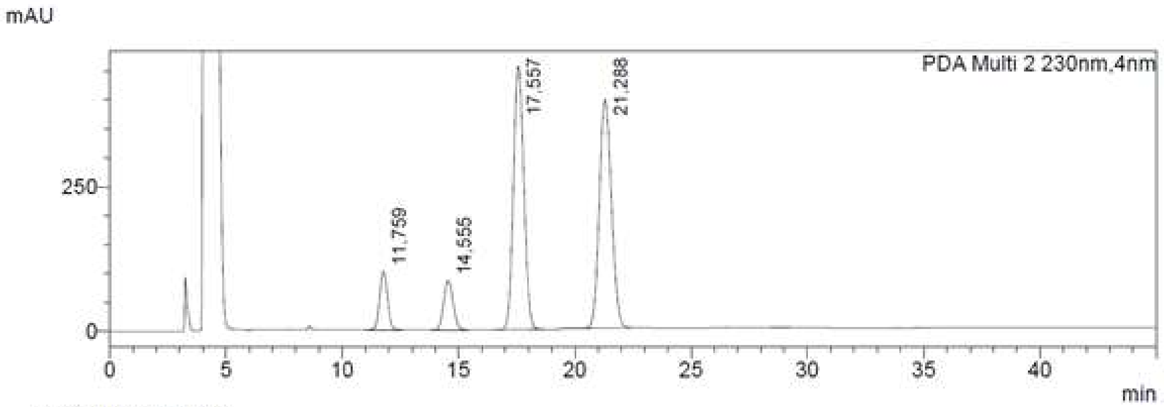

PDA Ch2 230nm

Peak#	Ret. Time	Name	Area	Height	Area%
1	11,759		2373985	100476	7,171
2	14,555		2353998	84825	7,111
3	17,557		14177970	454605	42,827
4	21,288		14199519	394956	42,892
Total			33105472	1034862	100,000

Figure S13: HPLC-UV chromatogram of the racemic reference product ethyl 2-benzamido-3-hydroxypentanoate

Synthesis of enantioenriched ethyl 2-benzamido-3-hydroxypentanoate

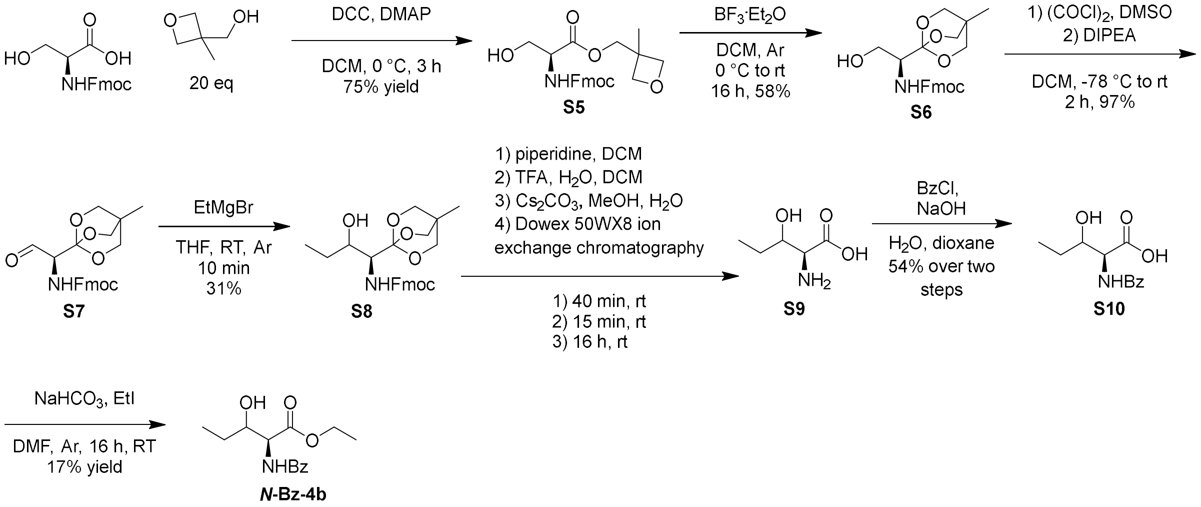

Figure S14: Synthesis of enantioenriched ethyl 2-benzamido-3-hydroxypentanoate

The first five steps of this synthesis (synthesis of ß-hydroxynorvaline) were done using a reported procedure.[32]
NMR spectra of compounds containing the acid-sensitive OBO ester were measured in acid-free $CDCl_3$, which was prepared by passing $CDCl_3$ through a pad of basic alumina.

Synthesis of N-*Fmoc-L-serine oxetane ester (**S5**)*[32]

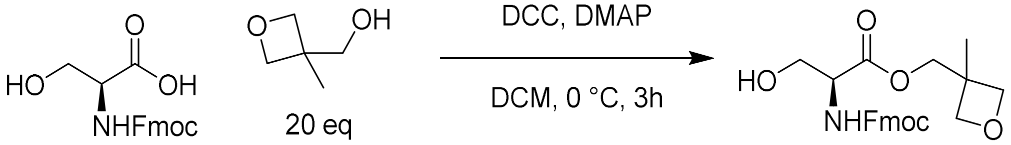

A solution of 3-methyl-3-oxetanemethanol (50 g; 490 mmol; 20 eq), DCC (7.58 g; 36.75 mmol; 1.5 eq) and DMAP (150 mg; 1.23 mmol; 5 mol%) in DCM (10 mL) was cooled to 0 °C. A solution of Fmoc-L-serine (8.012 g; 24.5 mmol; 1.0 eq) in DCM (600 mL) was added dropwise over 90 minutes. After stirring the mixture at 0 °C for 2.5 additional hours, the suspension was filtered to remove the urea by-product. The filtrate was washed with NH_4Cl (1% aq, 1x 500 mL) and $NaHCO_3$ (5% aq, 2x 500 mL), dried over Na_2SO_4, filtered and the solvent was removed under reduced pressure (40 °C). The crude product, 19 g of oil, was purified by column chromatography (SiO_2, EtOAc/cyclohexane 1:1, then 2:1, crude loaded in $CHCl_3$ +1% MeOH) to yield the desired ester in 75% yield (7.58 g; 18.33 mmol).

^{1}H NMR (300 MHz, Chloroform-d) δ 7.78 (d, J = 7.4 Hz, 2H), 7.63 (d, J = 6.8 Hz, 2H), 7.42 (t, J = 7.4 Hz, 2H), 7.33 (tt, J = 7.4, 1.2 Hz, 2H), 5.92 (d, J = 8.1 Hz, 1H), 4.50 (dp, J = 22.7, 5.9 Hz, 8H), 4.25 (t, J = 6.9 Hz, 1H), 4.19 – 4.07 (m, 2H), 3.92 (dd, J = 11.2, 3.2 Hz, 1H), 2.06 (s, 1H), 1.30 (d, J = 3.0 Hz, 3H).

^{13}C NMR (75 MHz, Chloroform-d) δ 170.7, 156.3, 143.8, 143.7, 141.3, 141.0, 127.8, 127.1, 125.1, 120.0, 79.5, 68.9, 67.2, 63.3, 56.4, 47.1, 39.7, 20.8.

The values match those reported in literature.[32]

Synthesis of N-*Fmoc-L-serine OBO ester(**S6**)*[32]

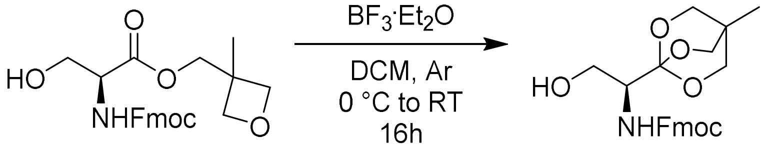

Boron trifluoride diethyl etherate (74 µL: 85.2 mg; 0.6 mmol; 5 mol%) was added to a solution of N-Fmoc-L-serine oxetane ester (4.85 g; 11.8 mmol; 1.0 eq) in anhydrous DCM (73 mL) cooled to 0 °C in a flamed out round-bottom flask under argon atmosphere. After stirring in the thawing ice bath overnight, the reaction was quenched by addition of triethylamine (2 mL). The solvent was removed under reduced pressure (40 ° C) and the crude residue was purified by column chromatography (SiO_2, EtOAc/cyclohexane, 8 to 60% EtOAc) to yield the desired product as a white powder in 58% yield (2.80 g, 6.8 mmol).

TLC R_f (SiO_2, EtOAc/cyclohexane 1:1) 0.24 (UV-254 nm).

^{1}H NMR (300 MHz, Chloroform-*d*) δ 7.78 (d, *J* = 7.5 Hz, 2H), 7.64 (t, *J* = 7.1 Hz, 2H), 7.45 – 7.39 (m, 2H), 7.33 (td, *J* = 7.4, 1.2 Hz, 2H), 5.39 (d, *J* = 9.5 Hz, 1H), 4.48 – 4.35 (m, 2H), 4.27 (t, *J* = 7.1 Hz, 1H), 3.96 (s, 8H), 3.71 (dq, *J* = 12.1, 4.9 Hz, 1H), 2.69 – 2.50 (m, 1H), 0.84 (s, 3H).

^{13}C NMR (75 MHz, Chloroform-*d*) δ 156.5, 144.1, 143.9, 141.3, 127.7, 127.0, 125.2, 120.0, 108.6, 72.8, 67.0, 62.0, 55.2, 47.2, 30.6, 14.3.

The values match those reported in literature.[32]

Synthesis of N-*Fmoc-L-ser(ald) OBO ester(**S7**)*[32]

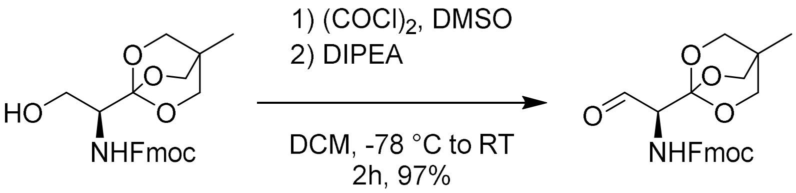

Oxalyl chloride (2 M in anhydrous DCM, 5.4 mL; 10.8 mmol; 1.6 eq) was diluted with anhydrous DCM (110 mL) in a flamed out 500 mL two neck round-bottom flask under argon atmosphere. The solution was cooled to -78 °C (liquid N_2, EtOH) and anhydrous DMSO (1.6 mL; 22.44 mmol; 3.3 eq) was added. After stirring for ten minutes, a pre-cooled (-78 °C) solution of the alcohol (2.80 g; 6.8 mmol; 1.0 eq) in anhydrous DCM (20 mL) was added to the oxalyl chloride-containing flask. The alcohol flask was rinsed with anhydrous DCM (1x 15 mL), which was also added to the mixture, which had turned into a white suspension. After stirring for 80 minutes at -78 °C, the cooling bath was removed and anhydrous DIPEA (5.92 mL; 34.0 mmol; 5.0 eq) was added and the mixture stirred at room temperature for 30 minutes. The mixture was then diluted with DCM (500 mL) and washed with NH_4Cl (3% aq, 2x 500 mL) and brine (1x 500 mL), dried over Na_2SO_4, filtered and the solvent was removed under reduced pressure (40 °C). This gave the product as a white solid in 97% yield (2.69 g; 6.6 mmol) in decent purity (TLC analysis (SiO_2, EtOAc:cyclohexane 1:1, R_f aldehyde 0.5) shows a small amount of the alcohol) that was not subjected to further purification as it is known to racemize during recrystallization or on silica gel.

^{1}H NMR (300 MHz, Chloroform-*d*) δ 9.74 (s, 1H), 7.78 (d, *J* = 7.5 Hz, 2H), 7.65 (d, *J* = 7.5 Hz, 2H), 7.42 (t, *J* = 7.4 Hz, 2H), 7.34 (td, *J* = 7.4, 1.3 Hz, 2H), 5.44 (d, *J* = 9.1 Hz, 1H), 4.67 (d, *J* = 9.1 Hz, 1H), 4.54 – 4.32 (m, 2H), 4.27 (t, *J* = 7.3 Hz, 1H), 3.99 (s, 6H), 0.86 (s, 3H).

^{13}C NMR (75 MHz, Chloroform-d) δ 195.7, 156.3, 143.9, 141.3, 127.7, 127.1, 125.3, 120.0, 107.2, 73.0, 67.5, 63.3, 47.1, 30.9, 14.3.

The values match those reported in literature.[32]

Grignard addition on N-*Fmoc-L-ser(ald) OBO ester (**S7**), synthesis of (9H-fluoren-9-yl)methyl ((1S)-2-hydroxy-1-(4-methyl-2,6,7-trioxabicyclo[2.2.2]octan-1-yl)butyl)carbamate (**S8**)*

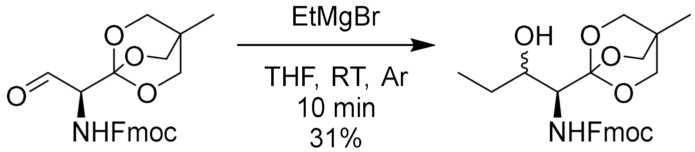

The procedure was based on one reported in literature.[32] The aldehyde (2.69 g; 6.6 mmol; 1.0 eq) was dissolved in anhydrous THF (66 mL) in a flamed out 250 mL two necked round-bottom flask under argon atmosphere. EtMgBr (1.0 M in THF, 40 mL, 40 mmol; 6.0 eq) was added and the mixture stirred at room temperature for 10 minutes. The solution was then poured into a mixture of NH_4Cl (5% aq, 500 mL) and DCM (400 mL). The organic layer was washed once more with NH_4Cl (5% aq, 500 mL), brine (1x 500 mL), dried over Na_2SO_4, filtered and the solvent was removed under reduced pressure (40 °C). The obtained crude (2.01 g) was purified by column chromatography (SiO_2, EtOAc:cyclohexane, 8 to 60% EtOAc) to give the desired product in 31% yield (911 mg; 2.07 mmol) as a white solid.

TLC (SiO_2, EtOAc:cyclohexane 1:2) R_f 0.5 (UV-254).

^{1}H NMR (300 MHz, Chloroform-*d*) δ 7.78 (d, *J* = 7.5 Hz, 2H), 7.64 (t, *J* = 7.4 Hz, 2H), 7.41 (t, *J* = 7.4 Hz, 2H), 7.33 (t, *J* = 7.3 Hz, 1H), 5.39 (d, *J* = 10.4 Hz, 1H), 4.42 (d, *J* = 6.2 Hz, 1H), 4.29 (d, *J* = 7.2 Hz, 1H), 4.20 – 4.04 (m, 1H), 3.96 (s, 3H), 3.88 (d, *J* = 10.3 Hz, 1H), 2.99 (s, 1H), 1.72 (s, 2H), 1.48 (ddt, *J* = 37.9, 13.7, 7.0 Hz, 1H), 0.95 (d, *J* = 7.5 Hz, 2H), 0.84 (s, 3H).

^{13}C NMR (75 MHz, Chloroform-*d*) δ 156.8, 144.2, 143.9, 141.3, 127.6, 127.6, 127.0, 125.24, 125.2, 120.0, 109.0, 72.8, 70.8, 66.9, 56.0, 47.0, 30.7, 26.0, 14.3, 10.1.

The values match those reported in literature.[33]

*Synthesis of ß-hydroxynorvaline (**S9**)*[32]

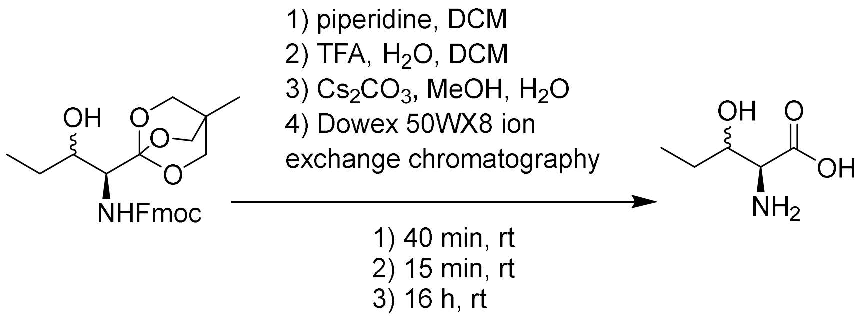

The synthesis was done using a modified literature reference.[32] Piperidine (5 mL, 50.62 mmol; 105 eq) was added to a solution of the protected amino acid (911 mg; 2.07 mmol; 1.0 eq) in DCM (20 mL). After stirring this mixture at room temperature for 20 minutes, the solvent was removed under reduced pressure (40 °C). The pale yellow residue thus obtained was redissolved in DCM (25 mL) and TFA (575 µL; 0.856 g; 7.51 mml; 3.63 eq) and water (410 µL, 410 mg; 22.76 mmol; 11 eq) were added. After stirring for 15 minutes, the solvent was again removed under reduced pressure (40 °C). Methanol (25 mL) was added to the pale yellow residue, which did not dissolve. Water (6.6 mL) and a solution of caesium carbonate (3.45 g; 10.6 mmol; 5.1 eq) in water (34.5 mL) was added, which resulted in a bubbling pale yellow suspension. After stirring at room temperature overnight, TLC (SiO_2, EtOAc/nBuOH/MeOH/H_2O) 1:1:1:1, ninhydrin stain) shows a large purple spot that smears out over the lower half of the TLC plate. The suspension was filtered through celite and the filtrate was acidified to pH 2.4 using 35% aq HCl. This solution was loaded onto a cation exchange column (Dowex 50WX8, hydrogen form, 12 cm x 1 cm) that was pre-washed with water and equilibrated with 0.01 N aq HCl. After loading the sample, the column was washed with 0.01 N aq HCl (200 mL), water (200 mL) and elution was attempted with 5% aq Et_3N (300 mL). As no product was obtained, the column was eluted

again with 1M aq NH_3 (1 L). The desired product was obtained in reasonable purity in quantitative yield.

TLC (SiO_2 EtOAc/nBuOH/MeOH/H_2O) 1:1:1:1, ninhydrin stain) R_f 0.55.

^{1}H NMR (300 MHz, Deuterium Oxide) δ 3.99 – 3.82 (m, 1H), 3.75 (d, *J* = 3.6 Hz, 0.2H), 3.58 (d, *J* = 4.3 Hz, 0.8H), 1.64 – 1.38 (m, 2H), 0.89 (t, *J* = 7.4 Hz, 3H).

^{13}C NMR (75 MHz, Deuterium Oxide) δ 173.1, 71.3, 64.4, 58.7, 46.7, 26.4, 9.4, 8.3.

The values match those reported in literature.[33]

*Benzoylation of ß-hydroxynorvaline, synthesis of (**S10**)*[1]

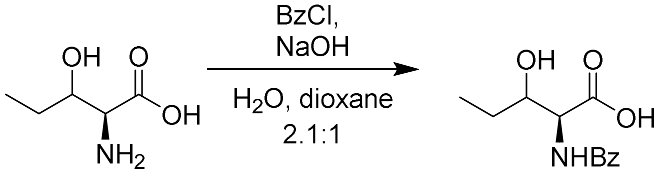

A solution of benzoyl chloride (279 µL; 2.4 mmol; 1.2 eq) in 1,4-dioxane (2 mL) was added slowly to a solution of the amino acid (266.3 mg; 2 mmol; 1.0eq) in 1 M aqueous NaOH (4.2 mmol; 2.1 eq) at room temperature. After stirring the mixture overnight, TLC analysis of the suspension (SiO_2; EtOAc/cyclohexane 1:1, UV-254 nm) shows some impurity, but mostly a spot with R_f 0, which the product is expected to have. After diluting with water (15 mL) and adjusting the pH from 3.7 to 9.0 with sat aq Na_2CO_3, the solution was extracted with EtOAc (2x 20 mL) to remove any amino ester impurities (formed from benzoylation of insufficiently deprotected material). The pH of the aqueous phase was then adjusted to 0.9 with 1 M aqueous HCl and extracted with EtOAc (3x 20 mL). The combined organic layers were dried over Na_2SO_4, filtered and the solvent was removed under reduced pressure (40 °C), which yielded the desired product in decent purity (NMR analysis shows minor amounts of EtOAc, 1,4-dioxane and benzoic acid) in 54% (266 mg; 1.12 mmol; yield over two steps, deprotection and benzoylation) as an amber-colored oil.

^{1}H NMR (300 MHz, Methanol-d_4) δ 7.89 (tt, *J* = 7.4, 1.7 Hz, 3H), 7.19 – 7.04 (m, 3H), 6.87 – 6.69 (m, 4H), 1.59 (p, *J* = 7.3 Hz, 2H), 1.09 – 0.95 (m, 3H).

^{13}C NMR (75 MHz, Methanol-d_4) δ 172.9, 132.6, 128.7, 127.0, 118.7, 115.8, 73.0, 56.6, 26.9, 13.1, 9.2.

NMR spectra of this compound have not previously been reported in literature

*Esterification of N-benzoyl-ß-hydroxynorvaline, synthesis of enantiomerically enriched **N-Bz-4b***[1]

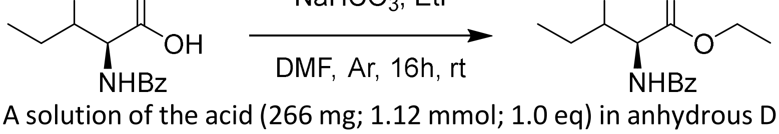

A solution of the acid (266 mg; 1.12 mmol; 1.0 eq) in anhydrous DMF (5 mL) at room temperature was purged with argon for five minutes. Then, ethyl iodide (450 µL, 5.6 mmol; 5.0 eq) and $NaHCO_3$ (264 mg; 3.14 mg; 2.8 eq) were added and the mixture was stirred overnight at room temperature. Then, the white suspension was poured into water (20 mL) and extracted with EtOAc (4x 20 mL). The combined organic layers were dried over Na_2SO_4, filtered and the solvent was removed under reduced pressure (40 °C). The crude product (approximately 1.1 g; still contained DMF) was purified by column chromatography (SiO_2, EtOAc/cyclohexane, 5 to 70% EtOAc) to yield the desired product in 17% isolated yield (50 mg; 0.19 mmol).

TLC R_f (SiO_2, EtOAc/cyclohexane 1:2) 0.22.

HPLC-UV analytics (Daicel Chiralpak IC column, n-heptane:2-PrOH 90:10, flow rate 1 mL/min, column oven 30 °C, 30 min) shows four distinct peaks with retention times of 11.50 min (73% of total area), 14.04 min (8%), 16.8 min (17%) and 20.4 min (2%) (see Figure S16), which were assigned the absolute configurations shown in Figure S15, based on the fact that an 80:20 diastereomeric ratio was determined *via* NMR spectroscopy of the amino acid, and the knowledge that the first two and second two peaks are enantiomers of each other (determined from a racemic sample that was synthesised in diastereomeric excess).

^{1}H NMR (700 MHz, Chloroform-d) δ 7.84 (d, J = 8.0 Hz, 2H), 7.50 (t, J = 7.5 Hz, 1H), 7.41 (q, J = 7.3 Hz, 2H), 7.12 (d, J = 9.3 Hz, 1H), 4.87 (dd, J = 19.4, 8.0 Hz, 1H), 4.30 – 4.17 (m, 2H), 4.14 (t, J = 7.0 Hz, 1H), 3.09 (s, 1H), 1.58 (qp, J = 14.9, 7.3 Hz, 2H), 1.28 (t, J = 7.4 Hz, 3H), 0.99 (t, J = 7.7 Hz, 3H).

^{13}C NMR (176 MHz, Chloroform-*d*) δ 171.49, 170.50, 168.00, 167.98, 133.81, 133.37, 132.04, 131.82, 128.64, 128.56, 127.25, 74.72, 73.69, 61.96, 61.76, 57.87, 56.29, 27.06, 26.50, 14.14, 10.32, 10.13. (Signals for both diastereomers are noted)

This compound has not previously been reported in literature.

A	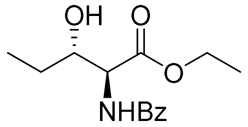	2S, 3S	11.50 min
B	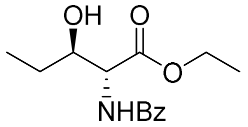	2R, 3R	14.04 min
C	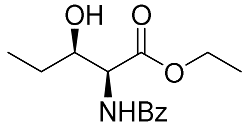	2S, 3R	16.8 min
D	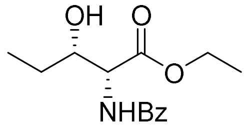	2R, 3S	20.4 min

Figure S15: Labeling, structure, absolute configuration and retention time on HPLC-UV of the reference product

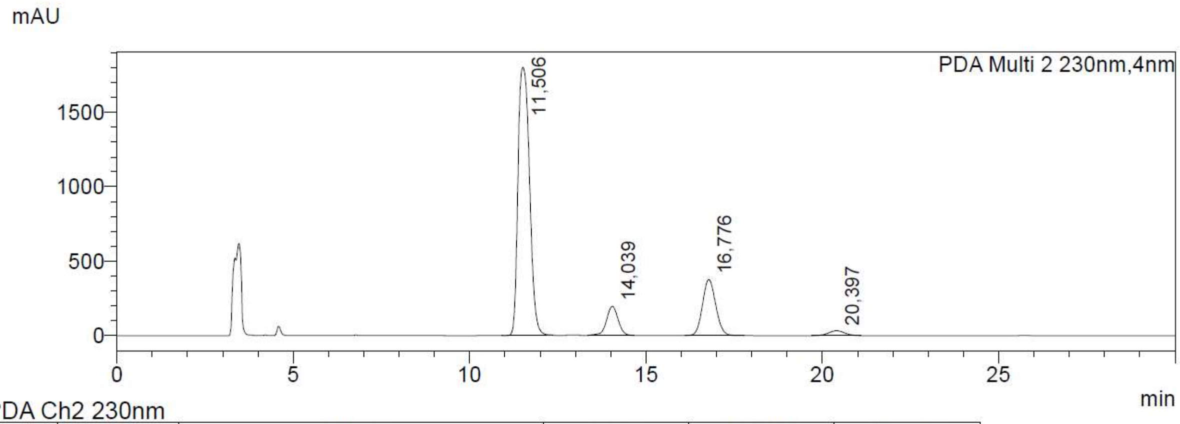

PDA Ch2 230nm

Peak#	Ret. Time	Name	Area	Height	Area%
1	11,506		40918419	1798157	72,805
2	14,039		4452046	195156	7,921
3	16,776		9801614	375985	17,440
4	20,397		1030479	32940	1,834
Total			56202560	2402239	100,000

Figure S16: HPLC-UV chromatogram of the enantioenriched reference product (this synthesis)

Reference products for cascade biotransformations on oxime 1c

Synthesis of 2-methoxyethyl benzoyl threoninates

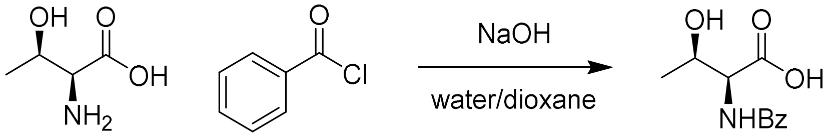

The four isomers of threonine were individually benzoylated using the procedure reported previously.[1]

Esterification of N-*benzoyl threonines, synthesis of **N-Bz-4c***

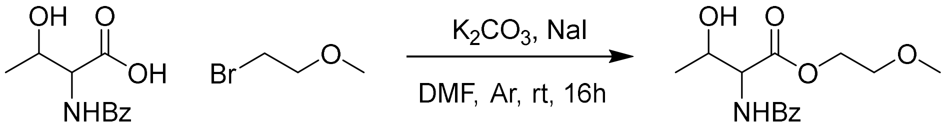

The esterifications were done by modifying a procedure from our previous publication.[1]

<u>For the D-threonine</u>: the carboxylic acid (863 mg; 3.7 mmol; 1.0 eq), 2-bromoethyl methyl ether (974 µL; 10.4 mmol; 2.8 eq) and sodium iodide (111 mg: 0.74 mmol; 0.2 eq) were dissolved in anhydrous DMF (20 mL) and potassium carbonate (1.44g; 10.4 mmol; 2.8 eq) was added. After stirring the suspension at room temperature overnight, the reaction mixture was filtered and the solvent of the filtrate was removed under reduced pressure (40 °C), the residue redissolved in brine/water (1:1, 20 mL). This was extracted with EtOAc (3x 20 mL). The combined organic layers were dried over Na_2SO_4, filtered and solvent was removed under reduced pressure (40 °C). The crude was purified by column chromatography (SiO_2, EtOAc/cy, 8 to 70% EtOAc) to yield the desired product in 40% isolated yield (223 mg; 1.5 mmol).

TLC R_f (SiO_2, EtOAc/cyclohexane) 0.05 with EtOAc/cy 1:1, R_f 0.43 with pure EtOAc.

^1H NMR (300 MHz, Chloroform-d) δ 7.92 – 7.83 (m, 2H), 7.57 – 7.49 (m, 1H), 7.48 – 7.40 (m, 2H), 7.09 (d, J = 8.9 Hz, 1H), 4.90 (dd, J = 8.9, 2.4 Hz, 1H), 4.58 – 4.42 (m, 2H), 4.29 (ddd, J = 12.0, 5.3, 3.5 Hz, 1H), 3.69 – 3.59 (m, 2H), 3.39 (s, 3H), 1.28 (d, J = 6.4 Hz, 3H).

^{13}C NMR (75 MHz, Chloroform-d) δ 171.08, 167.94, 133.80, 131.85, 128.58, 127.26, 70.22, 68.24, 63.85, 58.85, 57.92, 19.63.

ESI-MS (positive mode): calculated 282.13 for [M+H], found 282.2.

<u>For the L-threonine</u>: The same procedure was used, starting from 1.65 g (7.4 mmol) of *N*-benzoyl-L-threonine and using 41 mL of DMF. The stoichiometries for 2-bromoethyl methyl ether and sodium iodide were changed to 3.4 and 1.0 equivalents, respectively.

Yield: 186 mg; 1.24 mmol; 17%.

^1H NMR (300 MHz, Chloroform-d) δ 7.91 – 7.84 (m, 2H), 7.59 – 7.50 (m, 1H), 7.50 – 7.42 (m, 2H), 7.01 (d, J = 8.9 Hz, 1H), 4.91 (dd, J = 8.9, 2.4 Hz, 1H), 4.52 (ddd, J = 12.1, 6.2, 3.6 Hz, 2H), 4.30 (ddd, J = 12.0, 5.3, 3.4 Hz, 1H), 3.76 – 3.57 (m, 2H), 3.39 (s, 3H), 1.29 (dd, J = 6.8, 3.0 Hz, 3H).

^{13}C NMR (75 MHz, Chloroform-d) δ 171.07, 167.88, 133.83, 131.86, 128.60, 127.24, 70.23, 68.32, 63.79, 58.85, 57.88, 19.58.

ESI-MS (positive mode): calculated 282.13 for [M+H], found 282.2.

For the L-allothreonine: The same procedure was followed, starting from 480 mg (2.1 mmol) of *N*-benzoyl-L-allothreonine and using 11 mL of DMF. The stoichiometries for 2-bromoethyl methyl ether and sodium iodide were 5.0 and 1.0 equivalents, respectively. R_f: 0.15 (SiO_2, EtOAc/cy 1:1).

Yield: 119 mg; 0.42 mmol; 20% from L-allothreonine.

^{1}H NMR (300 MHz, Chloroform-d) δ 7.88 – 7.82 (m, 2H), 7.60 – 7.52 (m, 1H), 7.52 – 7.42 (m, 2H), 7.17 (d, J = 7.1 Hz, 1H), 4.90 (dd, J = 7.1, 3.4 Hz, 1H), 4.47 (dt, J = 11.9, 4.6 Hz, 1H), 4.41 – 4.24 (m, 2H), 3.65 (dd, J = 5.3, 4.1 Hz, 2H), 3.40 (s, 3H), 1.29 (d, J = 6.5 Hz, 3H).

^{13}C NMR (75 MHz, Chloroform-d) δ 170.19, 168.08, 133.34, 132.09, 128.68, 127.23, 70.01, 69.53, 64.37, 58.98, 58.89, 19.06.

ESI-MS (positive mode): calculated 282.13 for [M+H], found 282.2.

For the D-allothreonine: the reaction was carried out like the one for the L-allothreonine, starting from 385 mg of *N*-benzoyl-D-allothreonine and using 9.5 mL of DMF.

Yield: 216 mg; 0.77 mmol; 36% from D-allothreonine.

^{1}H NMR (300 MHz, Chloroform-d) δ 7.85 (dd, J = 7.2, 1.8 Hz, 2H), 7.60 – 7.50 (m, 1H), 7.50 – 7.41 (m, 2H), 7.20 (s, 1H), 4.96 – 4.83 (m, 1H), 4.45 (dtt, J = 10.8, 4.9, 1.1 Hz, 1H), 4.40 – 4.22 (m, 2H), 3.64 (t, J = 4.8 Hz, 2H), 3.39 (t, J = 0.8 Hz, 3H), 1.29 (d, J = 6.5 Hz, 3H).

^{13}C NMR (75 MHz, Chloroform-d) δ 170.18, 168.00, 133.40, 132.03, 128.64, 127.24, 70.02, 69.46, 64.33, 58.98, 58.88, 36.54.

ESI-MS (positive mode): calculated 282.13 for [M+H], found 282.2.

Analytics

GC-FID, achiral stationary phase

Reactions following general procedure B were analyzed using the GC method "achiral_lowboilers" (Table S22) on an Agilent 7890A GC with FID detector. The retention times are: cyclohexanone: 6.80 min, cyclohexenone: 7.45 min, tetramethylpyrazine (internal standard): 9.99 min (Figure S17).

Table S22: Details on analytical method "achiral_lowboilers"

Name	Achiral_lowboilers
Column	J&W HP-5 (30m x 250 µm x 0.25 µm)
Carrier gas	Helium
Column gas flow rate	1.2991 mL/min
Inlet temperature	250 °C
Detector temperature	300 °C
Injection volume	1 µL
Split ratio	90:1
Oven temperature program	40 °C, hold 2 min, ramp 10 °C/min to 180 °C, hold 1 min
Total run time	17 min

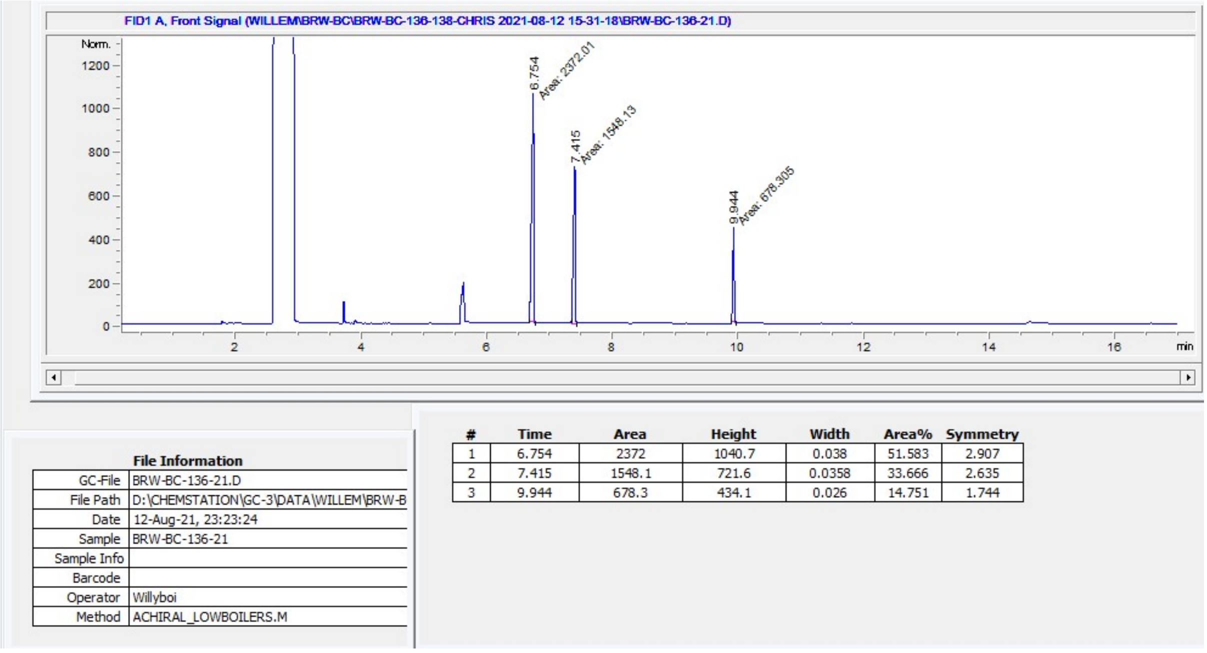

Figure S17: GC-FID spectrum of biotransformation of cyclohexenone with OPR3_Y190F as catalyst showing cyclohexanone (6.75 min), cyclohexenone (7.45 min) and tetramethylpyrazine (internal standard, 9.94 min).

Reactions following general procedure A were analyzed using the GC method "achiral_msd" (Table S23). The retention times are: tetramethylpyrazine (IS): 3.83 min, pyrazine 2A: 11.03, pyrazine 2C: 15.15 min, pyrazine 2H: 11.86 min.

Table S23: Details on analytical method "achiral_msd"

Name	Achiral_msd
Column	J&W HP-5 (30m x 250 µm x 0.25 µm)
Carrier gas	Helium
Column gas flow rate	1.1913 mL/min
Inlet temperature	250 °C

Detector temperature	300 °C
Injection volume	1 µL
Split ratio	90:1
Oven temperature programme	100 °C, 0.5 min, 10 °C/s to 300 °C, no hold
Total run time	20.5 min

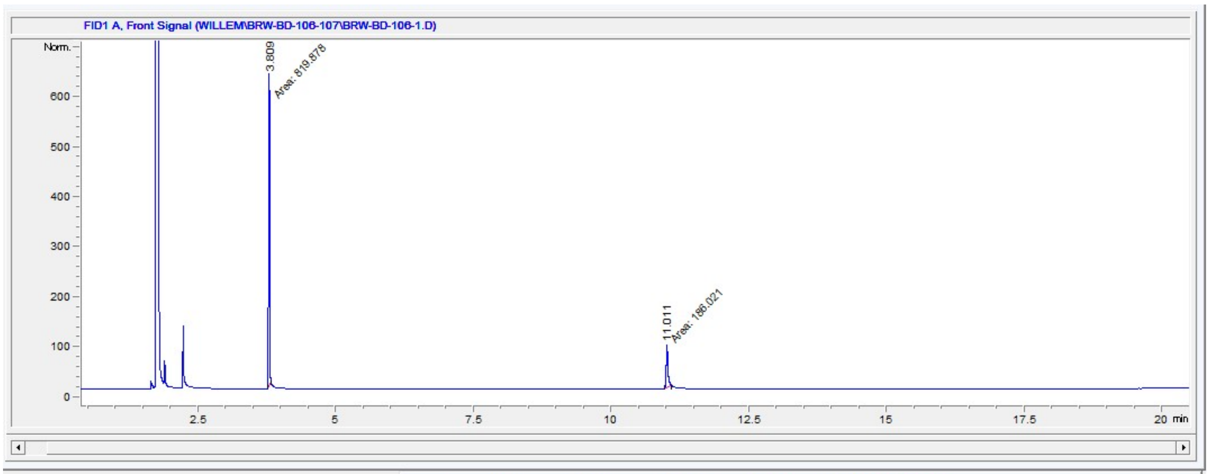

#	Time	Area	Height	Width	Area%	Symmetry
1	3.809	819.9	626.6	0.0218	81.507	1.745
2	11.011	186	86.1	0.036	18.493	0.731

Figure S18: GC-FID chromatogram of biotransformation of Z-ethyl 2-(methoxyimino)-3-oxobutanoate to pyrazine 3a with OYE3 as catalyst showing tetramethylpyrazine (internal standard, 3.81 min) and pyrazine 3a (11.01 min).

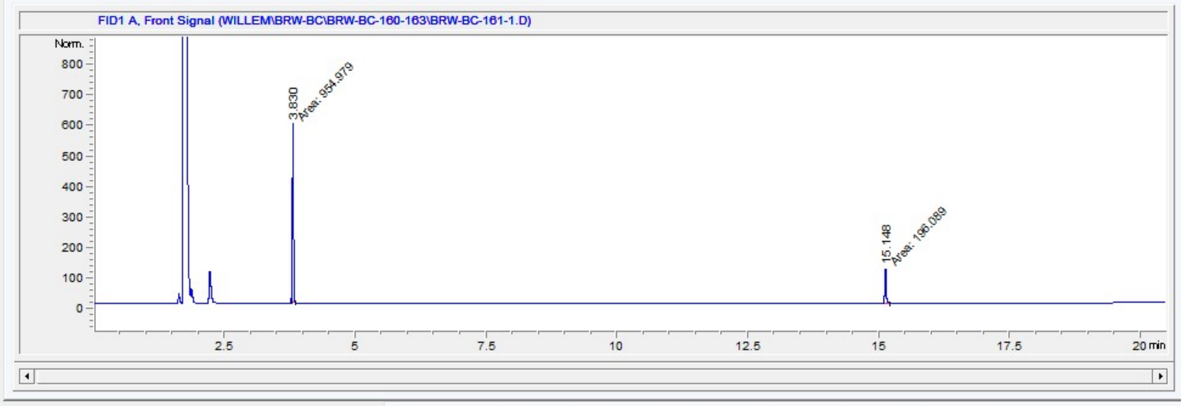

#	Time	Area	Height	Width	Area%	Symmetry
1	3.83	955	583.8	0.0273	82.965	1.569
2	15.148	196.1	113.6	0.0288	17.035	1.135

File Information

GC-File	BRW-BC-161-1.D
File Path	D:\CHEMSTATION\GC-3\DATA\WILLEM\BRW-B
Date	23-Sep-21, 19:36:34
Sample	BRW-BC-161-1
Sample Info	
Barcode	
Operator	Will
Method	ACHIRAL.M

Figure S19: GC-FID chromatogram of biotransformation of oxime 1c to pyrazine 3c with OPR3_WT as catalyst showing tetramethylpyrazine (internal standard, 3.83 min) and pyrazine 3c (15.15 min).

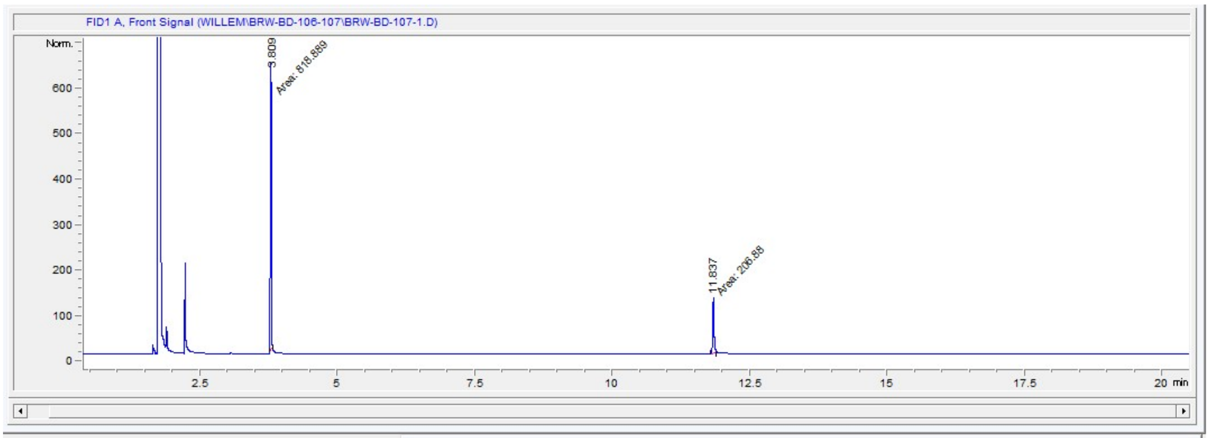

#	Time	Area	Height	Width	Area%	Symmetry
1	3.809	818.9	632.5	0.0216	79.832	1.507
2	11.837	206.9	121.7	0.0283	20.168	0.944

*Figure SS20: GC-FID chromatogram of biotransformation of Z-ethyl 2-(methoxyimino)-3-oxopentanoate (**Z-7b**) to pyrazine **3b** with OYE3 as catalyst showing tetramethylpyrazine (internal standard, 3.81 min) and pyrazine **3b** (11.84 min).*

HPLC-UV, chiral stationary phase

Cascade reactions with oxime **1a** and **1b**, following general procedure C, were analyzed on a Shimadzu HPLC system (Communication Bus Module CBM-20 A, Column Oven CTO-20 AC, Degasser DGU-20 A5, Liquid Chromatograph LC-20 AD, Auto sampler SIL-20 AC, Diode Array Detector SPD-M20 A) using a Daicel Chiralpak IC column with the parameters shown in Table S24.

Table S24: Parameters for HPLC-UV analysis with Daicel Chiralpak IC chiral column

Column	Daicel Chiralpak IC
n-heptane/2-PrOH	90:10 (isocratic)
Flow rate	1 mL/min
Oven temperature	30 °C
Total time	30 min
Absorption wavelength used for analysis	230 nm

Retention times of the compounds analyzed after cascade reactions with can be found in Table S25**.**

Table S25: Retention times of compounds analyzed in the cascade reactions.

Compound	Structure	Retention time (min)	Chromatogram
Oxime **1a** (substrate)	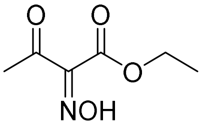	8.03	Figure S21
Pyrazine **3a** (product from non-cascade pathway)	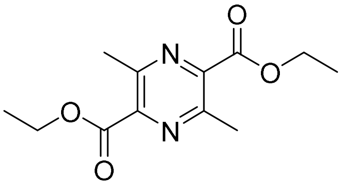	8.89	Figure S22
Acetanilide (internal standard)	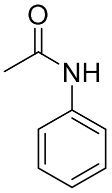	13.9	Figure S23

Benzanilide (internal standard)	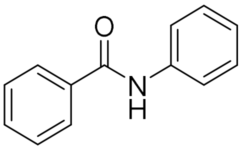	12.7	Figure S24
N-benzoyl-L-threonine ethyl ester	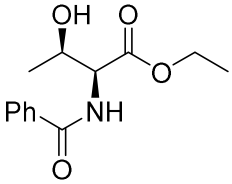	15.1	Figure S25
N-benzoyl-D-threonine ethyl ester	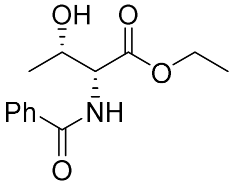	19.0	Figure S26
N-benzoyl-L-allothreonine ethyl ester	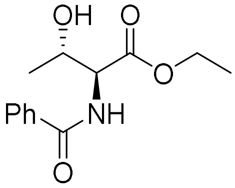	22.2	Figure S27
N-benzoyl-D-allothreonine ethyl ester	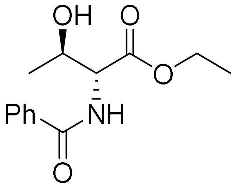	27.4	Figure S28
ethyl (2*S*,3*S*)-2-benzamido-3-hydroxypentanoate	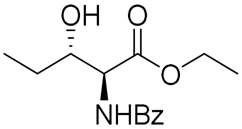	11.5	Figure S31, Figure S32
ethyl (2*R*,3*R*)-2-benzamido-3-hydroxypentanoate	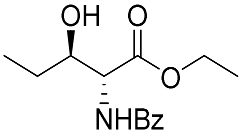	14.04	Figure S31, Figure S32
ethyl (2*S*,3*R*)-2-benzamido-3-hydroxypentanoate	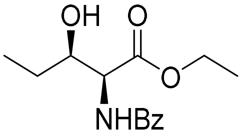	16.8	Figure S31, Figure S32
ethyl (2*R*,3*S*)-2-benzamido-3-hydroxypentanoate	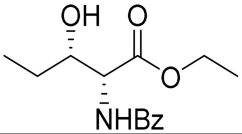	20.4	Figure S31, Figure S32

For cascade reactions with oxime **1a** as substrate, a chromatogram showing pyrazine **3a**, internal standard and all four enantiomers of the threonine ethyl ester product is shown in Figure S29. NB: an injection peak due to ethyl acetate is shown at 4.25 min. A chromatogram from a typical cascade biotransformation (with OYE3 and ADH-A) is shown in Figure S30.

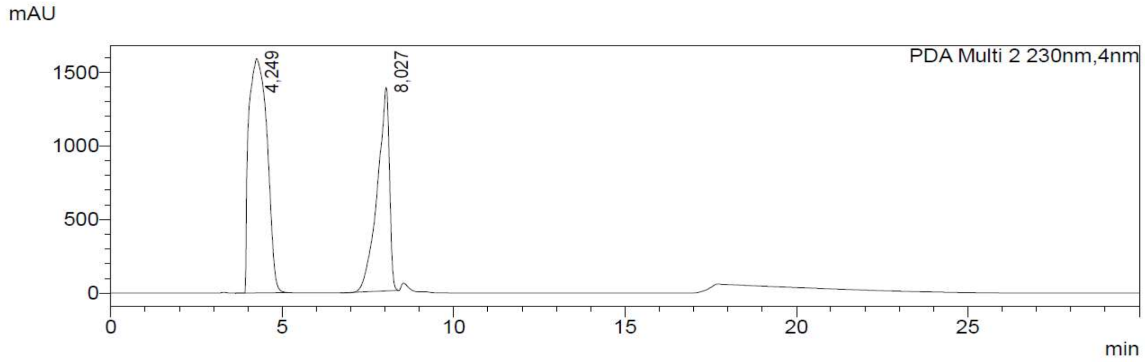

PDA Ch2 230nm

Peak#	Ret. Time	Name	Area	Height	Area%
1	4,249		58674011	1590880	63,317
2	8,027		33992519	1382594	36,683
Total			92666530	2973474	100,000

*Figure S21: HPLC-UV chromatogram of oxime **1a***

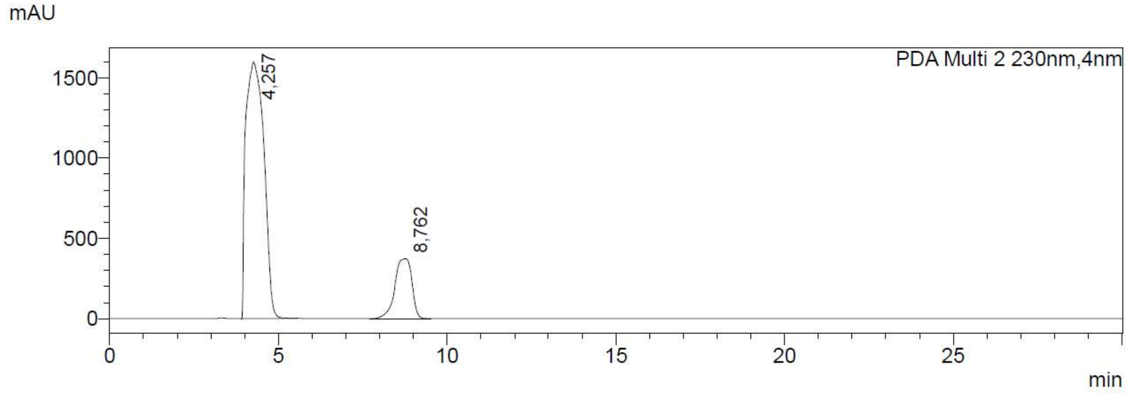

PDA Ch2 230nm

Peak#	Ret. Time	Name	Area	Height	Area%
1	4,257		59649741	1597881	82,029
2	8,762		13067794	373664	17,971
Total			72717535	1971545	100,000

*Figure S22: HPLC-UV chromatogram of pyrazine **3a***

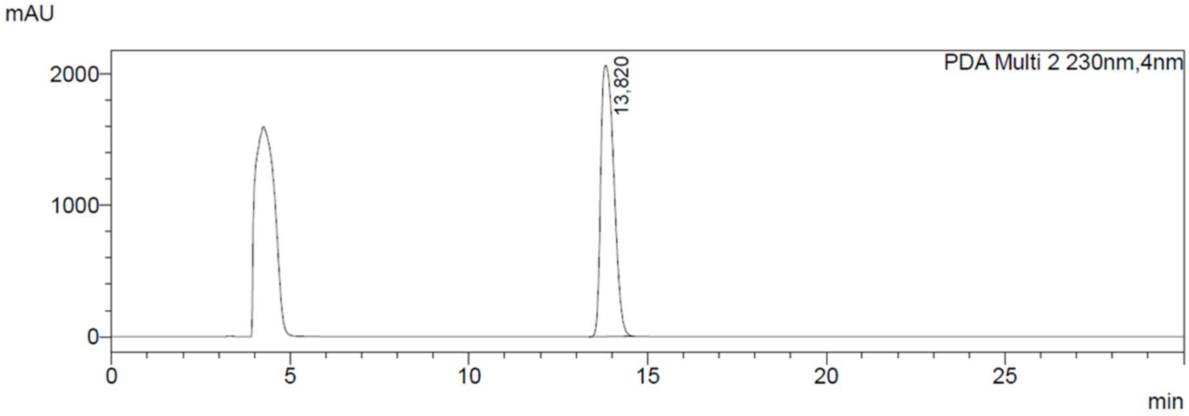

PDA Ch2 230nm

Peak#	Ret. Time	Name	Area	Height	Area%
1	13,820		52744215	2062433	100,000
Total			52744215	2062433	100,000

Figure S23: HPLC-UV chromatogram of acetanilide (analyzed on Daicel Chiralpak IC column)

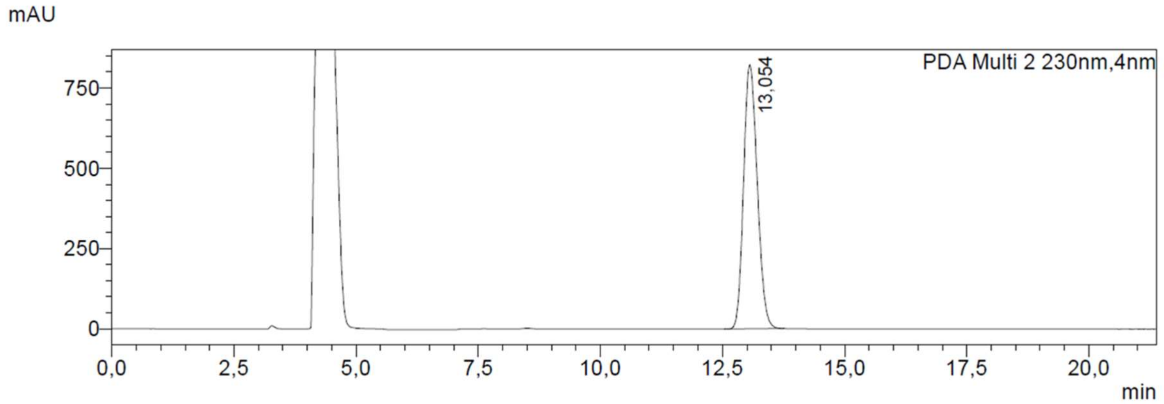

PDA Ch2 230nm

Peak#	Ret. Time	Name	Area	Height	Area%
1	13,054		16053224	822288	100,000
Total			16053224	822288	100,000

Figure S24: HPLC-UV chromatogram of benzanilide

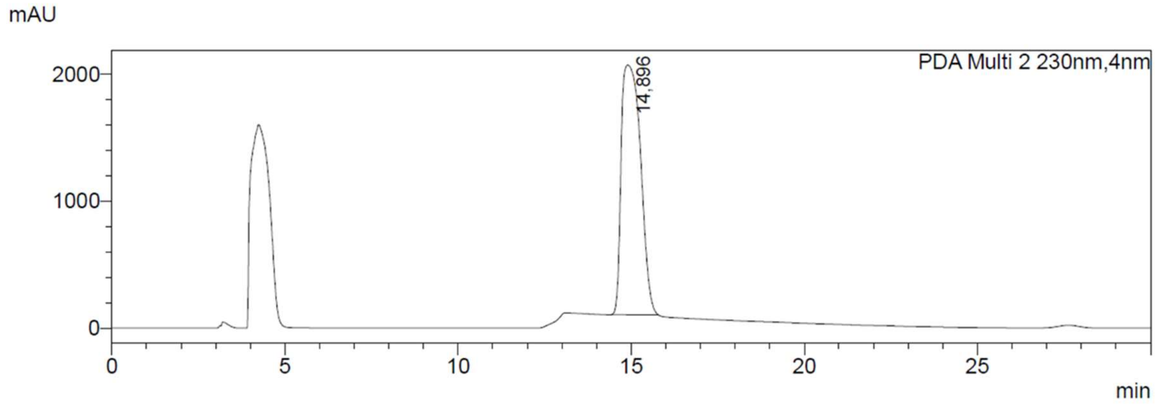

PDA Ch2 230nm

Peak#	Ret. Time	Name	Area	Height	Area%
1	14,896		76303714	1964925	100,000
Total			76303714	1964925	100,000

*Figure S25: HPLC-UV chromatogram of **L**-N-**Bz-4a***

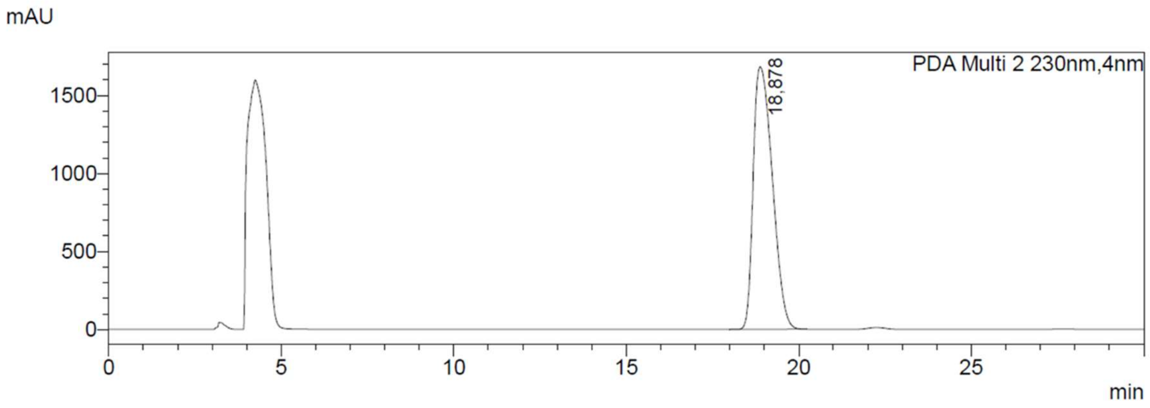

PDA Ch2 230nm

Peak#	Ret. Time	Name	Area	Height	Area%
1	18,878		65055968	1683567	100,000
Total			65055968	1683567	100,000

Figure S26: HPLC-UV chromatogram of ***D*****-N-*****Bz-4a***

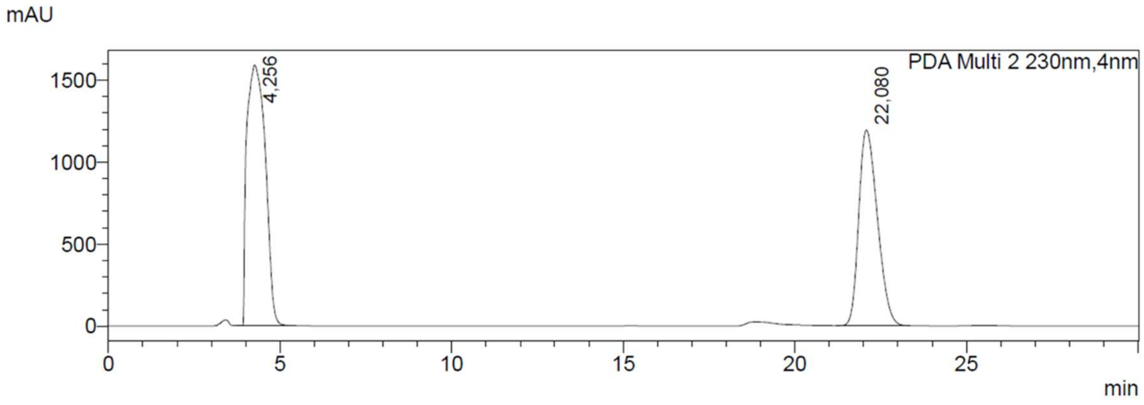

PDA Ch2 230nm

Peak#	Ret. Time	Name	Area	Height	Area%
1	4,256		59160465	1589233	56,713
2	22,080		45155376	1193816	43,287
Total			104315841	2783049	100,000

Figure S27: HPLC-UV chromatogram of ***L-allo*****-N-*****Bz-4a***

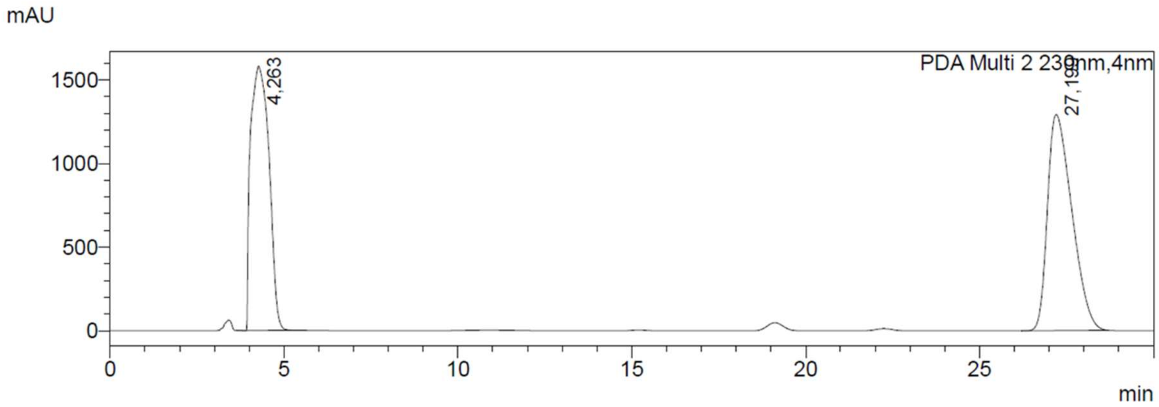

PDA Ch2 230nm					
Peak#	Ret. Time	Name	Area	Height	Area%
1	4,263		58120694	1580236	47,916
2	27,199		63176732	1291981	52,084
Total			121297426	2872218	100,000

*Figure S28: HPLC-UV chromatogram of **D-allo-*N*-Bz-4a***

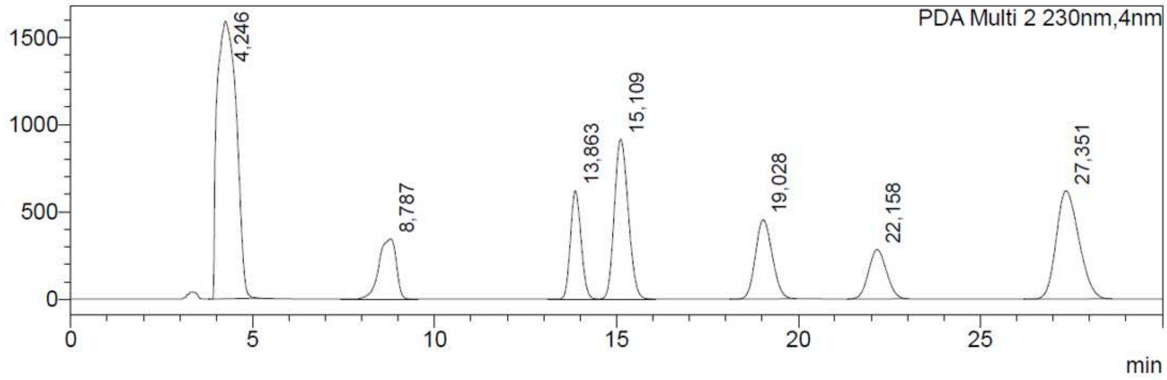

PDA Ch2 230nm					
Peak#	Ret. Time	Name	Area	Height	Area%
1	4,246		58035467	1589633	36,848
2	8,787		11405194	344893	7,241
3	13,863		12582689	620737	7,989
4	15,109		24249389	914769	15,396
5	19,028		14562373	453148	9,246
6	22,158		9733840	282977	6,180
7	27,351		26932059	620239	17,100
Total			157501010	4826396	100,000

*Figure S29: HPLC-UV chromatogram of pyrazine **3a**, acetanilide (IS) and the four enantiomers of N-benzoyl threonine ethyl ester (**N-Bz-4a**)*

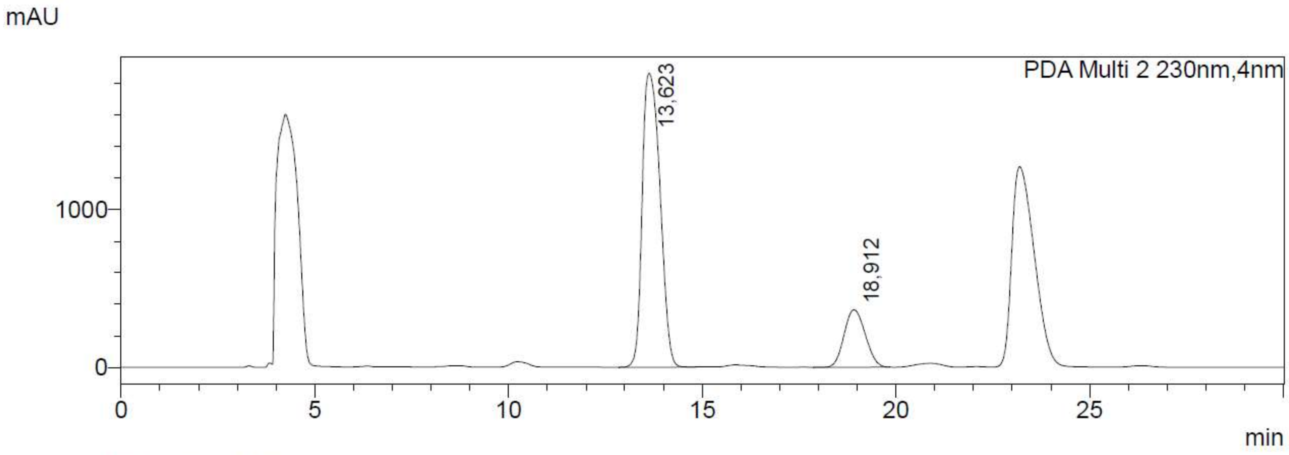

PDA Ch2 230nm

Peak#	Ret. Time	Name	Area	Height	Area%
1	13,623		59532742	1863943	81,110
2	18,912		13864517	364489	18,890
Total			73397259	2228432	100,000

Figure S30: HPLC-UV chromatogram of cascade biotransformation from oxime 1a (using OYE3 and ADH-A as catalysts), showing the internal standard (13.6 min), N-benzoyl ethyl D-threoninate (18.9 min) and an unidentified side product (23.4 min).

For cascade reactions with oxime **1b** as substrate, a chromatogram of a racemic sample (with dr of 86:14) is shown in Figure S31, a chromatogram of the enantioenriched product in Figure S32. A chromatogram of a biotransformation of oxime **1b** (with XenA and *Lk*ADH-Lica) is shown in Figure S33. NB: retention times vary slightly, likely because the column oven temperatures were not exactly identical.

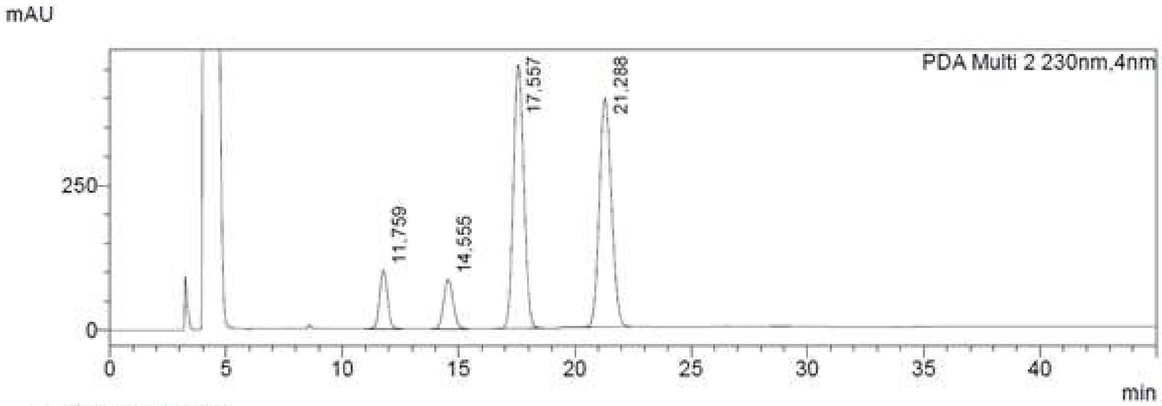

PDA Ch2 230nm

Peak#	Ret. Time	Name	Area	Height	Area%
1	11,759		2373985	100476	7,171
2	14,555		2353998	84825	7,111
3	17,557		14177970	454605	42,827
4	21,288		14199519	394956	42,892
Total			33105472	1034862	100,000

Figure S31: HPLC-UV chromatogram of racemic ethyl 2-benzamido-3-hydroxypentanoate (N-Bz-4b)

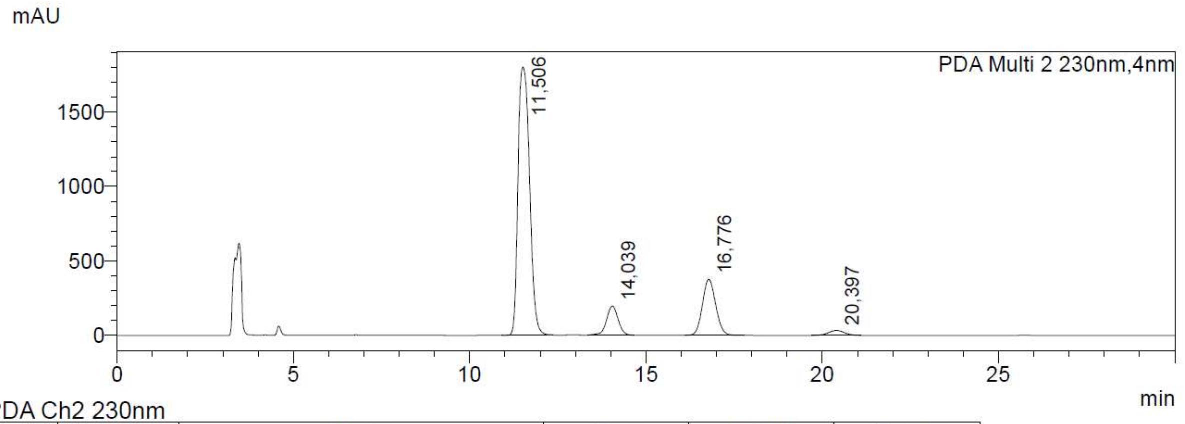

PDA Ch2 230nm

Peak#	Ret. Time	Name	Area	Height	Area%
1	11,506		40918419	1798157	72,805
2	14,039		4452046	195156	7,921
3	16,776		9801614	375985	17,440
4	20,397		1030479	32940	1,834
Total			56202560	2402239	100,000

Figure S32: HPLC-UV chromatogram of the enantioenriched ethyl 2-benzamido-3-hydroxypentanoate (N-Bz-4b), showing the 2S,3S isomer at 11.51 min, the 2R,3R isomer at 14.04 min, the 2S,3R isomer at 16.78 min and the 2R,3S isomer at 20.40 min.

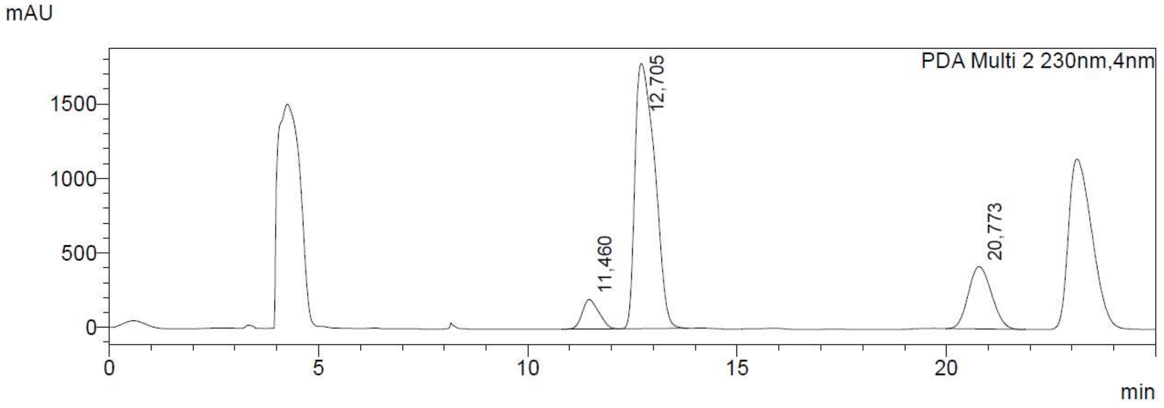

Figure S33: HPLC-UV chromatogram of a cascade biotransformation of oxime 1b with XenA and LkADH-Lica as catalysts, showing 2S,3S ethyl 2-benzamido-3-hydroxypentanoate (11.46 min), benzanilide (internal standard, 12.771 min) and 2R,3S ethyl 2-benzamido-3-hydroxypenta

PDA Ch2 230nm

Peak#	Ret. Time	Name	Area	Height	Area%
1	11,460		5446508	198200	6,788
2	12,705		58621105	1780106	73,058
3	20,773		16171051	419108	20,154
Total			80238664	2397413	100,000

Cascade reactions with oxime **1c**, following general procedure C, were analyzed by HPLC-UV using a Daicel Chiralpak IE column with the parameters shown in Table S26.

Table S26: Parameters for HPLC-UV analysis with Daicel Chiralpak IE chiral column

Column	Daicel Chiralpak IE
n-heptane/2-PrOH	80:20
Flow rate	1 mL/min
Oven temperature	30 °C
Total time	30 min
Absorption wavelength used for analysis	230 nm

Retention times of the compounds analyzed after cascade reactions with can be found in Table S27**.**

Table S27: Retention times of compounds analyzed in the cascade reactions.

Compound	Structure	Retention time (min)	Chromatogram
Acetanilide (internal standard)	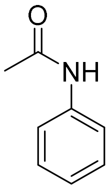	5.44	Figure S34
N-benzoyl-D-threonine 2-methoxyethyl ester	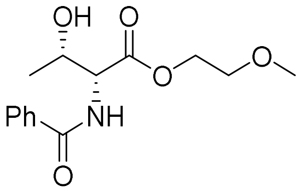	15.98	Figure S35
N-benzoyl-D-allo-threonine 2-methoxyethyl ester	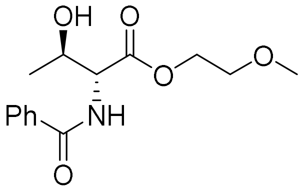	17.64	Figure S36
N-benzoyl-L-allo-allothreonine 2-methoxyethyl ester	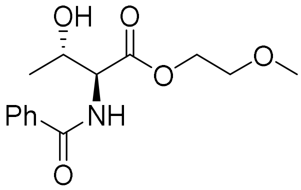	22.75	Figure S37
N-benzoyl-L-threonine 2-methoxyethyl ester	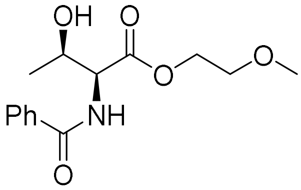	24.5	Figure S38
Pyrazine **3c**	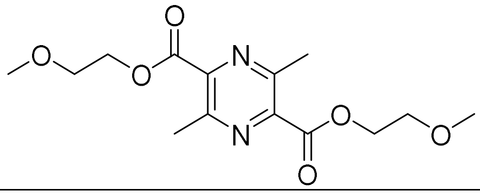	25.64	Figure S39

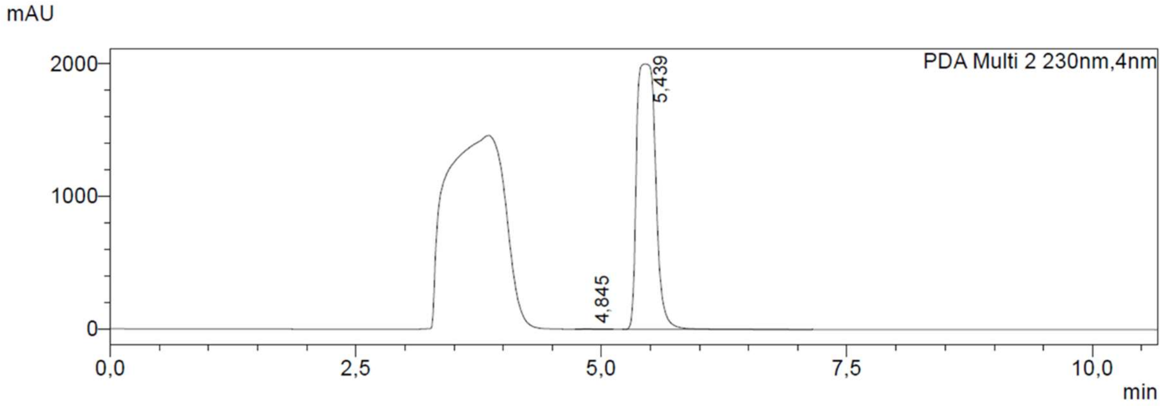

PDA Ch2 230nm

Peak#	Ret. Time	Name	Area	Height	Area%
1	4,845		12314	1680	0,046
2	5,439		26814301	1997548	99,954
Total			26826615	1999227	100,000

Figure S34: HPLC-UV chromatogram of acetanilide (analyzed on Daicel Chiralpak IE column)

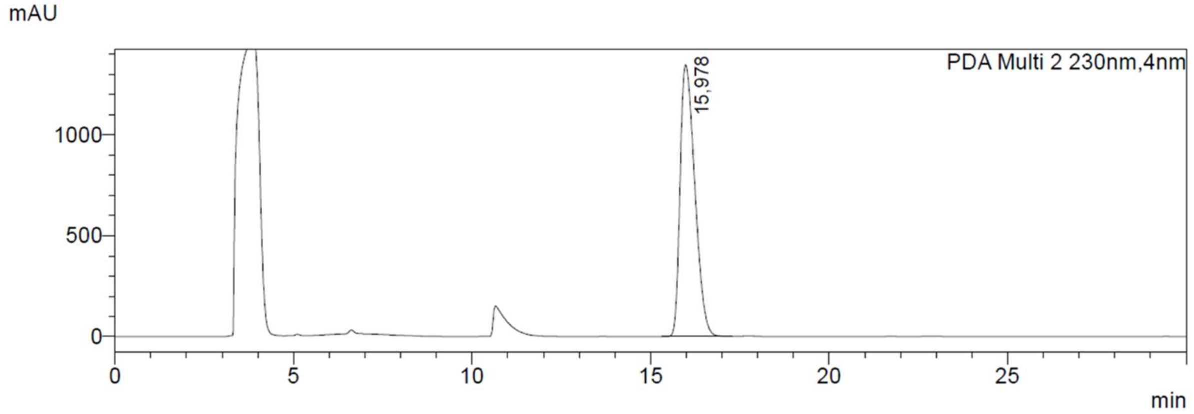

PDA Ch2 230nm

Peak#	Ret. Time	Name	Area	Height	Area%
1	15,978		38632566	1347885	100,000
Total			38632566	1347885	100,000

*Figure S35: HPLC-UV chromatogram of **D**-N-**Bz-4c***

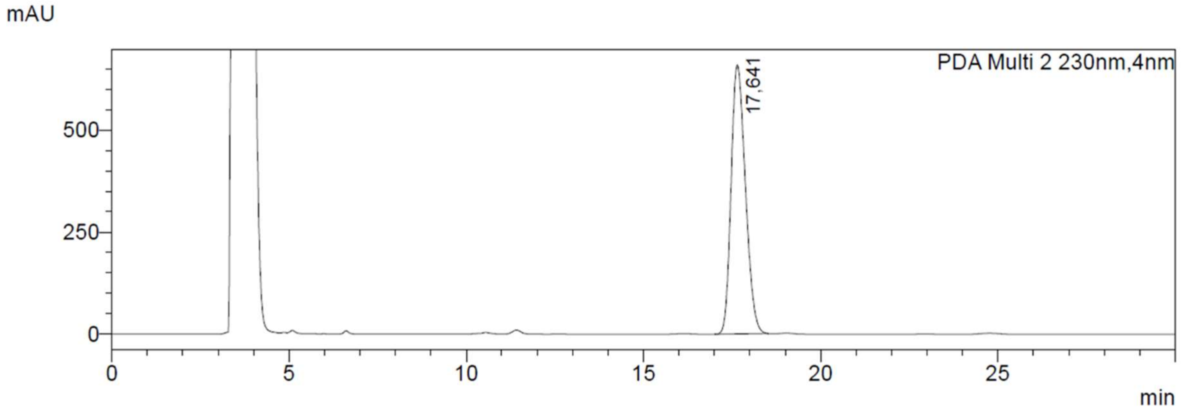

PDA Ch2 230nm

Peak#	Ret. Time	Name	Area	Height	Area%
1	17,641		18504482	659186	100,000
Total			18504482	659186	100,000

Figure S36: HPLC-UV chromatogram of ***D-allo*-N-*Bz-4c***

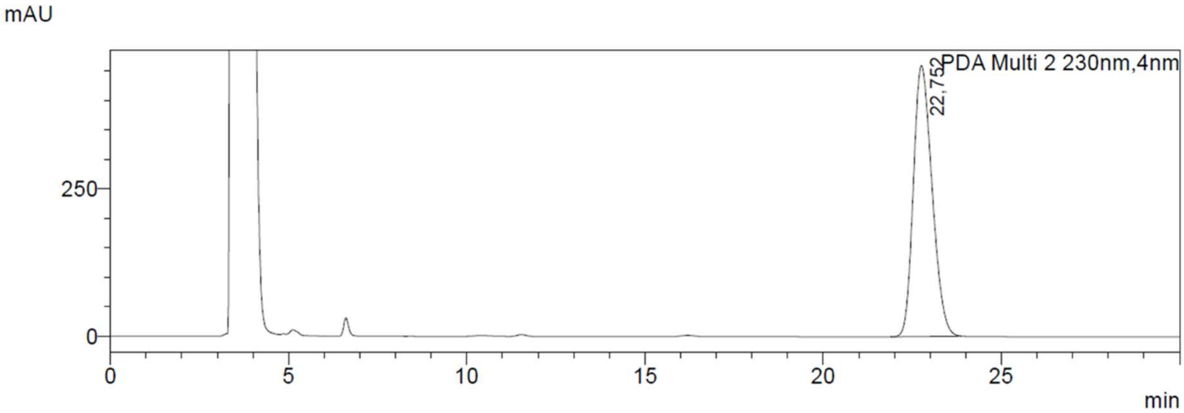

PDA Ch2 230nm

Peak#	Ret. Time	Name	Area	Height	Area%
1	22,752		16818201	459208	100,000
Total			16818201	459208	100,000

Figure S37: HPLC-UV chromatogram of ***L-allo*-N-*Bz-4c***

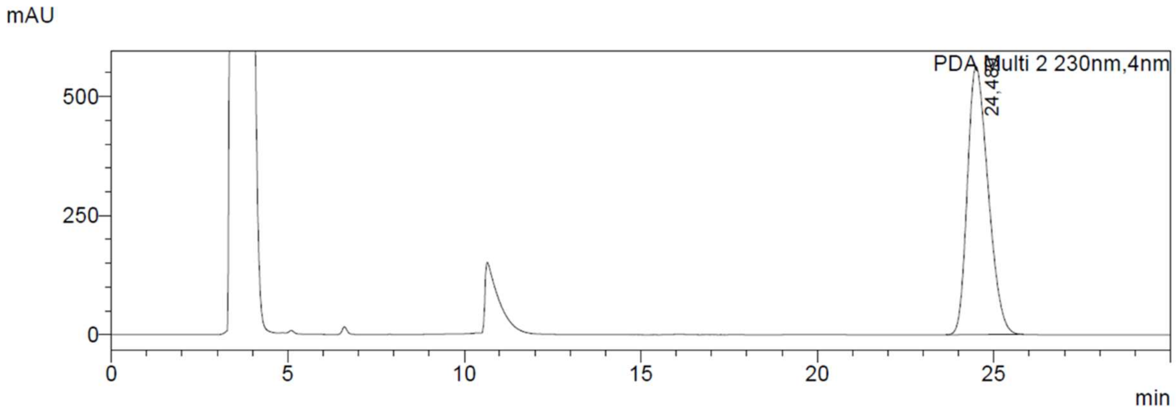

PDA Ch2 230nm

Peak#	Ret. Time	Name	Area	Height	Area%
1	24,489		23009202	563246	100,000
Total			23009202	563246	100,000

*Figure S38: HPLC-UV chromatogram of **L**-N-**Bz-4c***

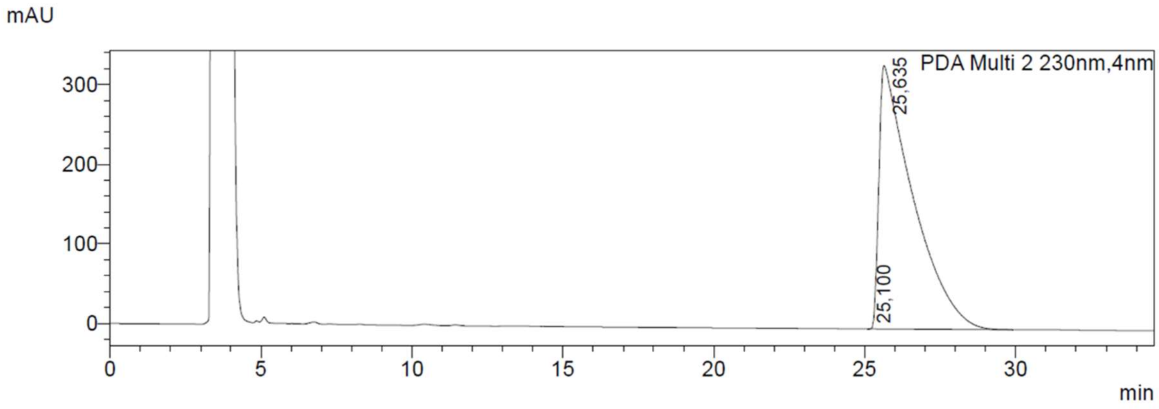

PDA Ch2 230nm

Peak#	Ret. Time	Name	Area	Height	Area%
1	25,100		-4	2	-0,000
2	25,635		25941204	330636	100,000
Total			25941200	330639	100,000

*Figure S39: HPLC-UV chromatogram of pyrazine **3c***

For cascade reactions with oxime **1c** as substrate, a chromatogram showing the internal standard and all four enantiomers of the *N*-benzoyl threonine 2-methoxyethyl ester product (***N*-Bz-4c**) is shown in Figure S40. A chromatogram from a typical cascade biotransformation (with OPR3 and ADH-A) is shown in Figure S41.

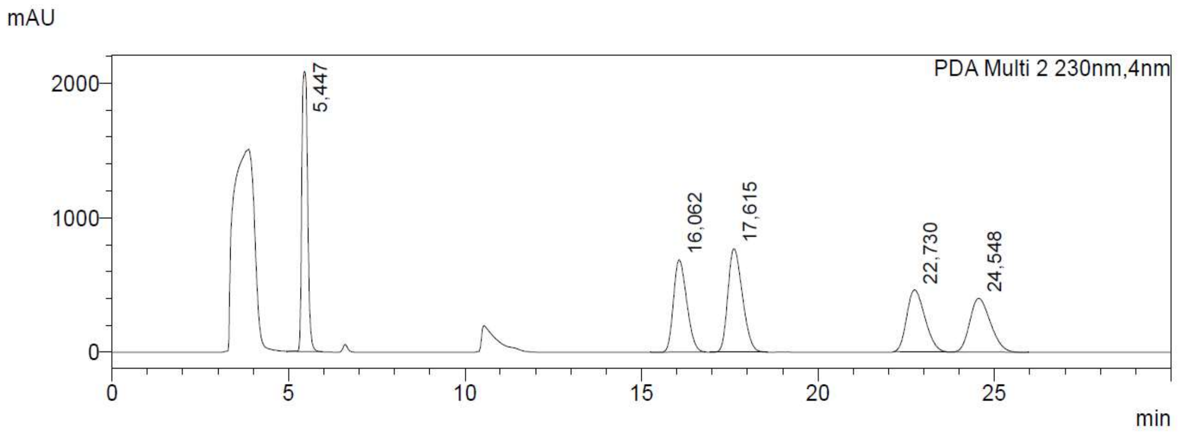

PDA Ch2 230nm

Peak#	Ret. Time	Name	Area	Height	Area%
1	5,447		23896374	2080322	24,656
2	16,062		17935319	685486	18,505
3	17,615		22076439	769648	22,778
4	22,730		16770532	463281	17,303
5	24,548		16242329	398853	16,758
Total			96920993	4397589	100,000

PDA Ch2 2

Peak#	Ret
1	
2	
3	
4	
5	
Total	

*Figure S40: HPLC-UV chromatogram of acetanilide and all four isomers of the N-benzoyl threonine 2-methoxyethyl ester (N-Bz-**4c**)*

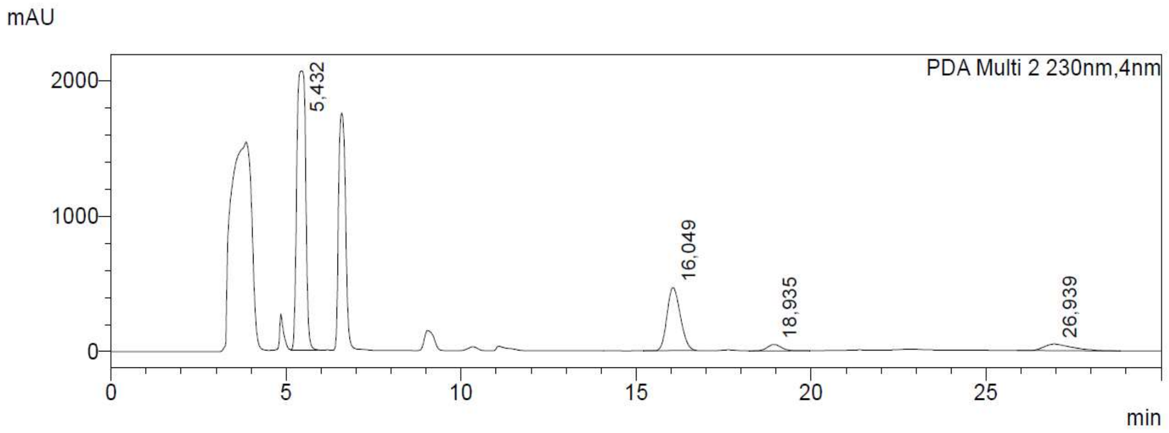

PDA Ch2 230nm

Peak#	Ret. Time	Name	Area	Height	Area%
1	5,432		35849392	2056470	68,952
2	16,049		12200742	464149	23,467
3	18,935		1272454	46413	2,447
4	26,939		2668882	48043	5,133
Total			51991471	2615075	100,000

PDA Ch2 230

Peak#	Ret. Ti
1	5,
2	16,
3	18,
4	26,
Total	

*Figure S41: HPLC-UV chromatogram of a biotransformation of oxime **1c** with OPR3 and ADH-A as catalysts*

Reversed-phase HPLC-UV (achiral stationary phase, qualitative)

Oxime ethers and hydroxylamines

For reactions using general procedure A with oxime ethers or hydroxylamines as substrate, analysis was done by RP-HPLC-UV using a Shimadzu HPLC system (Communication Bus Module CBM-20 A, Column Oven CTO-20 AC, Degasser DGU-20 A5, Liquid Chromatograph LC-20 AD, Auto sampler SIL-20 AC HT, Diode Array Detector SPD-M20 A) using a Phenomenex Luna® 5 µm C18(2) 100 Å column (250 x 4.6 mm) eluting with the following gradient of acetonitrile in water (both containing 0.1% TFA): 10% MeCN for two minutes, linear gradient to 100% MeCN over 26 minutes, hold at 100% MeCN for two minutes, then straight drop to 10% MeCN, hold at 10% MeCN for three minutes. Flow rate: 1 mL/min, column oven temperature 25 °C, wavelength used for analysis: 230 nm. The retention times can be found in Table S28. Representative chromatograms for these reactions can be found in Figure S50 through Figure S54.

Table S28: Retention times on RP-HPLC

Name	Number	Structure	Retention time (minutes)	Chromatogram
ethyl (*Z*)-2-(methoxyimino)-3-oxobutanoate	*Z*-**7a**	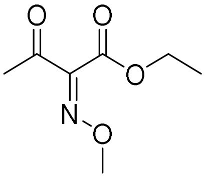	18.29	Figure S42
ethyl (*E*)-2-(methoxyimino)-3-oxobutanoate	*E*-**7a**	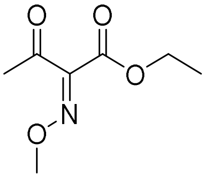	16.70	Figure S43
ethyl 2-(methoxyamino)-3-oxobutanoate	**8a**	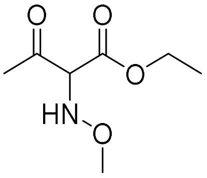	13.09	Figure S44
diethyl 3,6-dimethylpyrazine-2,5-dicarboxylate	**3a**	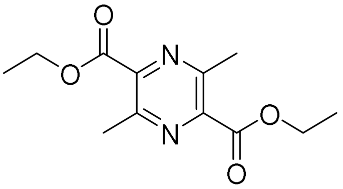	19.0	Figure S45
ethyl (*Z*)-2-(methoxyimino)-3-oxopentanoate	*Z*-**7b**	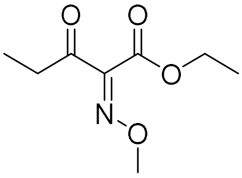	20.597	Figure S46
ethyl (*E*)-2-(methoxyimino)-3-oxopentanoate	*E*-**7b**	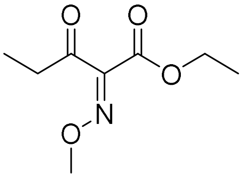	19.014	Figure S47
ethyl 2-(methoxyamino)-3-oxopentanoate	**8b**	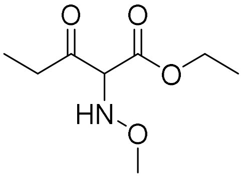	15.497	Figure S48
diethyl 3,6-diethylpyrazine-2,5-dicarboxylate	**3b**	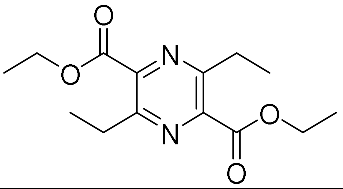	23.714	Figure S49

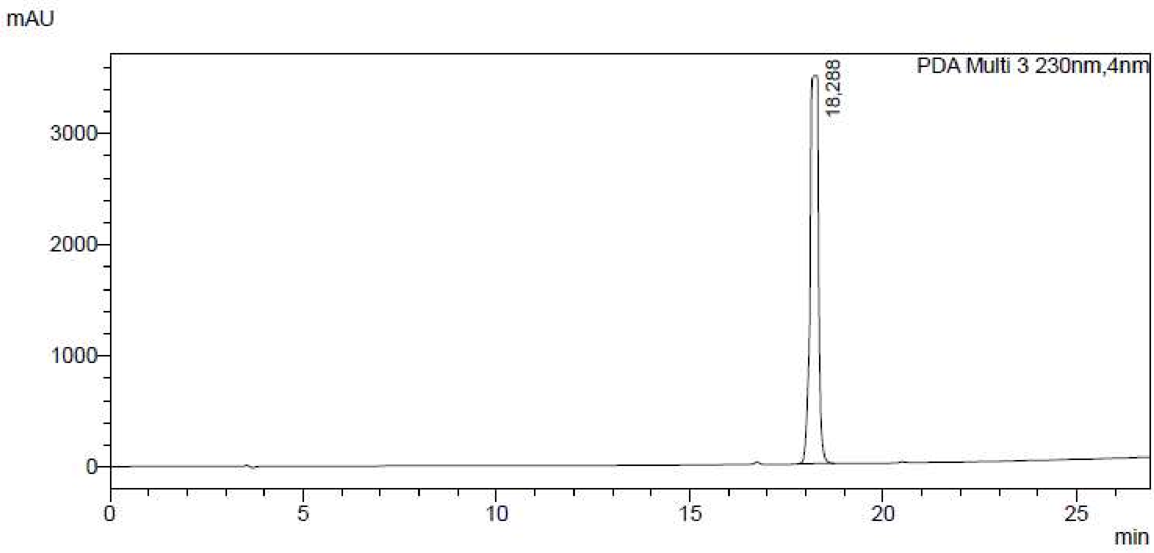

PDA Ch3 230nm

Peak#	Ret. Time	Area	Height	Conc.	Unit	Mark	Name
1	18,288	52955161	3488372	0,000		M	
Total		52955161	3488372				

Figure S42: RP-HPLC-UV chromatogram of ethyl (Z)-2-(methoxyimino)-3-oxobutanoate ***Z-7a***

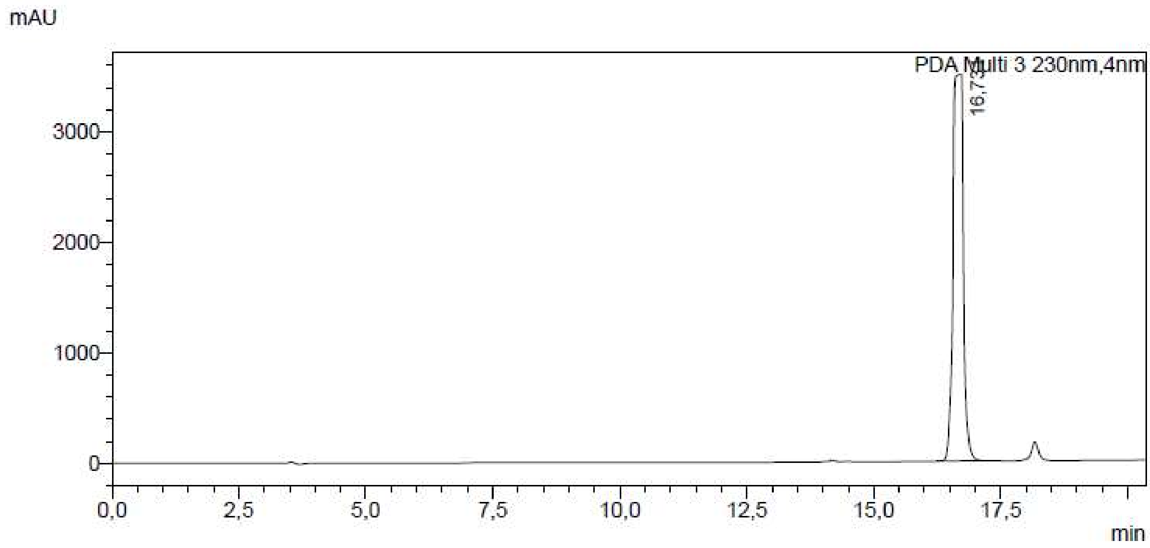

PDA Ch3 230nm

Peak#	Ret. Time	Area	Height	Conc.	Unit	Mark	Name
1	16,731	49188921	3492649	0,000		M	
Total		49188921	3492649				

Figure S43: RP-HPLC-UV chromatogram of ethyl (E)-2-(methoxyimino)-3-oxobutanoate ***E-7a***

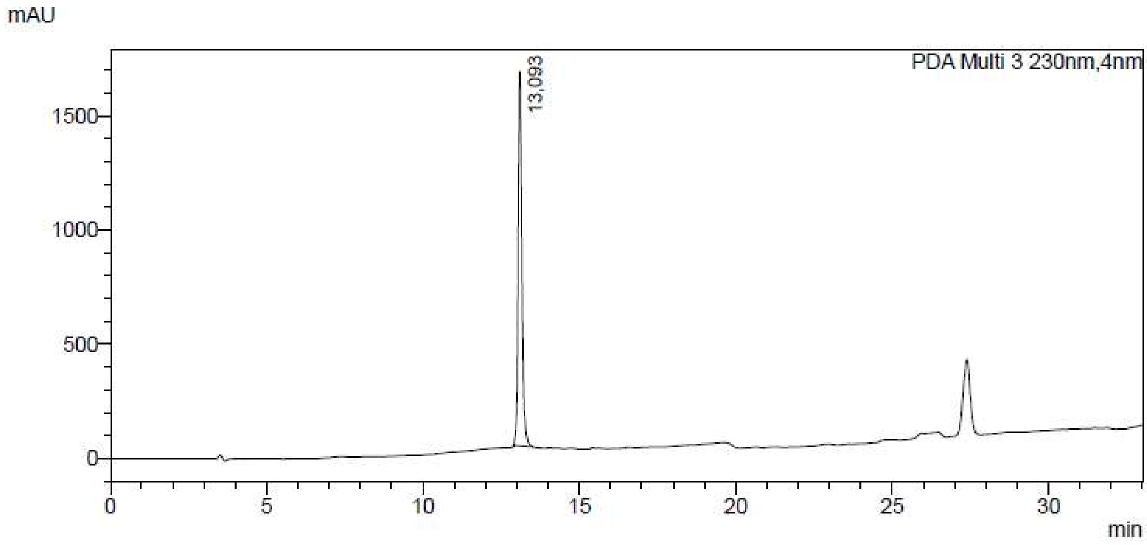

PDA Ch3 230nm

Peak#	Ret. Time	Area	Height	Conc.	Unit	Mark	Name
1	13,093	13773950	1635042	100,000		M	
Total		13773950	1635042				

Figure S44: RP-HPLC-UV chromatogram of ethyl 2-(methoxyamino)-3-oxobutanoate ***8a***

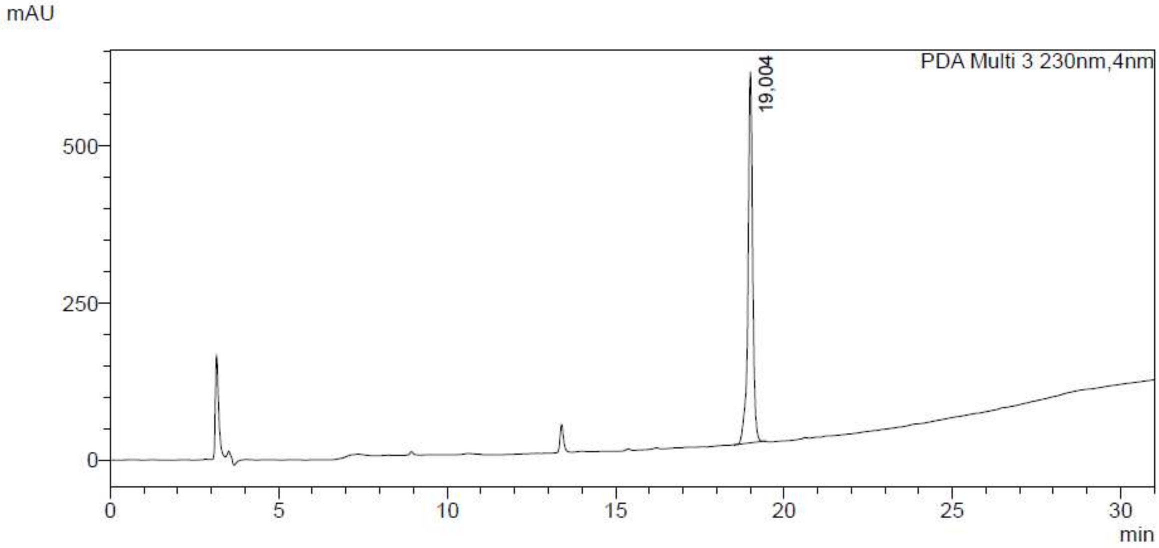

PDA Ch3 230nm

Peak#	Ret. Time	Area	Height	Conc.	Unit	Mark	Name
1	19,004	5717736	590837	0,000		M	
Total		5717736	590837				

Figure S45: RP-HPLC-UV chromatogram of diethyl 3,6-dimethylpyrazine-2,5-dicarboxylate (pyrazine ***3a****)*

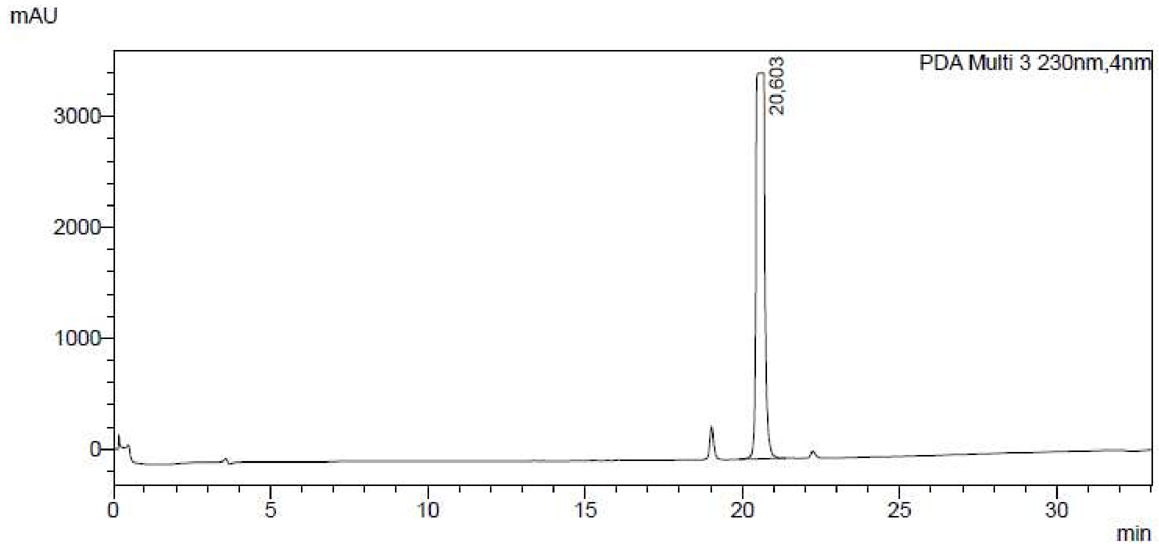

PDA Ch3 230nm

Peak#	Ret. Time	Area	Height	Conc.	Unit	Mark	Name
1	20,603	66529551	3482053	0,000		M	
Total		66529551	3482053				

*Figure S46: RP-HPLC-UV chromatogram of ethyl (Z)-2-(methoxyimino)-3-oxopentanoate **Z-7b***

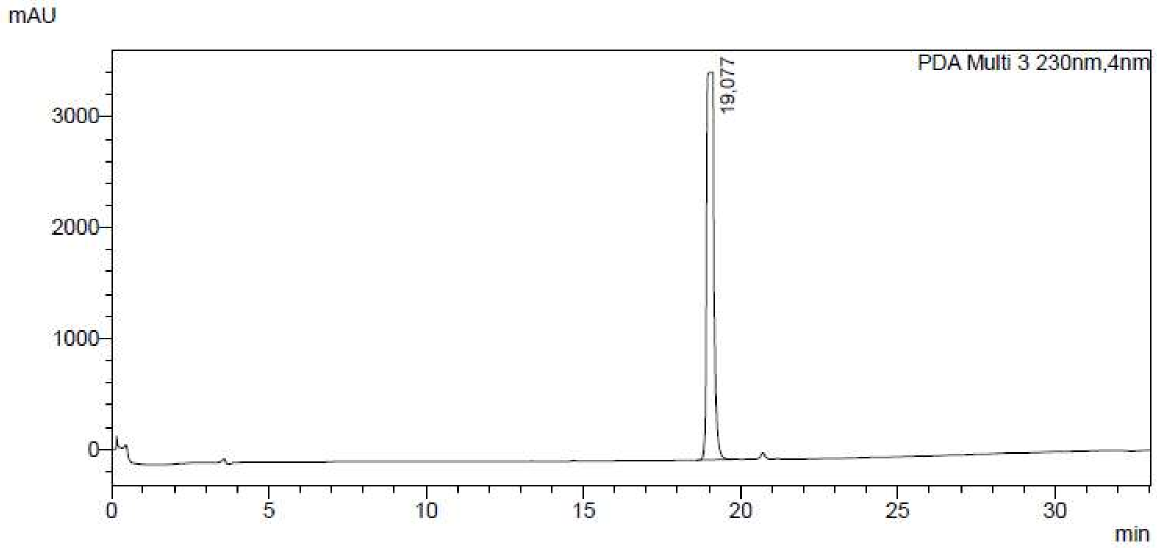

PDA Ch3 230nm

Peak#	Ret. Time	Area	Height	Conc.	Unit	Mark	Name
1	19,077	57899060	3488309	0,000		M	
Total		57899060	3488309				

*Figure S47: RP-HPLC-UV chromatogram of ethyl (E)-2-(methoxyimino)-3-oxopentanoate **E-7b***

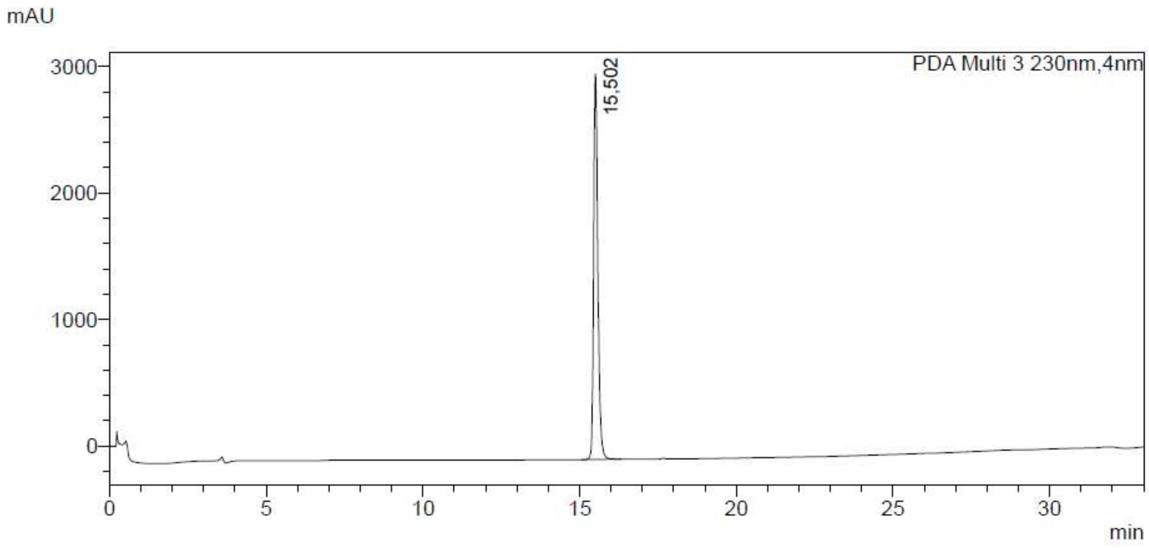

PDA Ch3 230nm

Peak#	Ret. Time	Area	Height	Conc.	Unit	Mark	Name
1	15,502	28597300	3047085	0,000		M	
Total		28597300	3047085				

*Figure S48: RP-HPLC-UV chromatogram of ethyl 2-(methoxyamino)-3-oxopentanoate **8b***

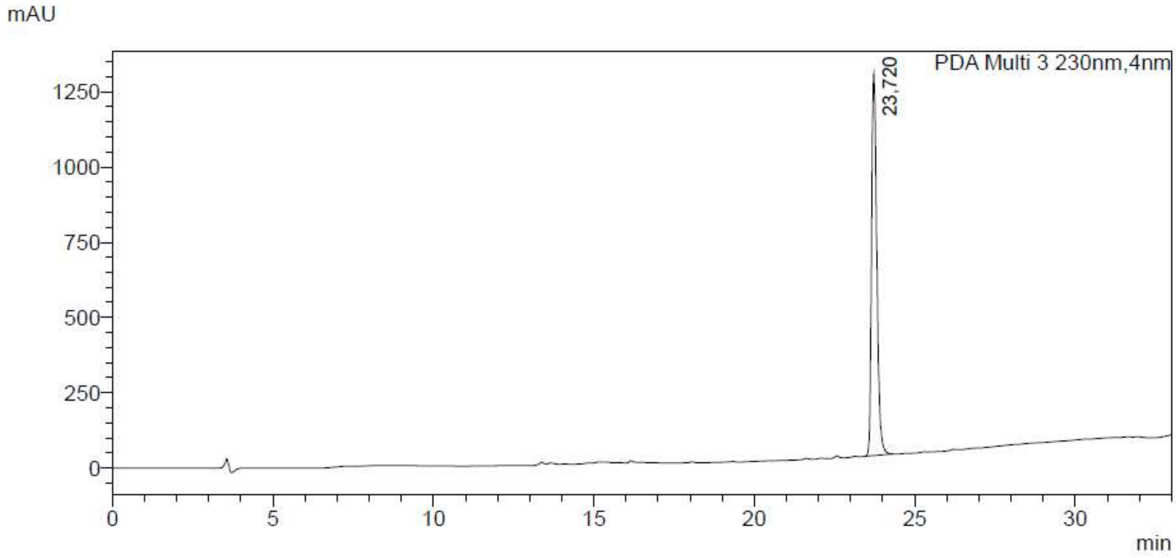

PDA Ch3 230nm

Peak#	Ret. Time	Area	Height	Conc.	Unit	Mark	Name
1	23,720	14547698	1270622	0,000		M	
Total		14547698	1270622				

*Figure S49: RP-HPLC-UV chromatogram of diethyl 3,6-diethylpyrazine-2,5-dicarboxylate (pyrazine **3b**)*

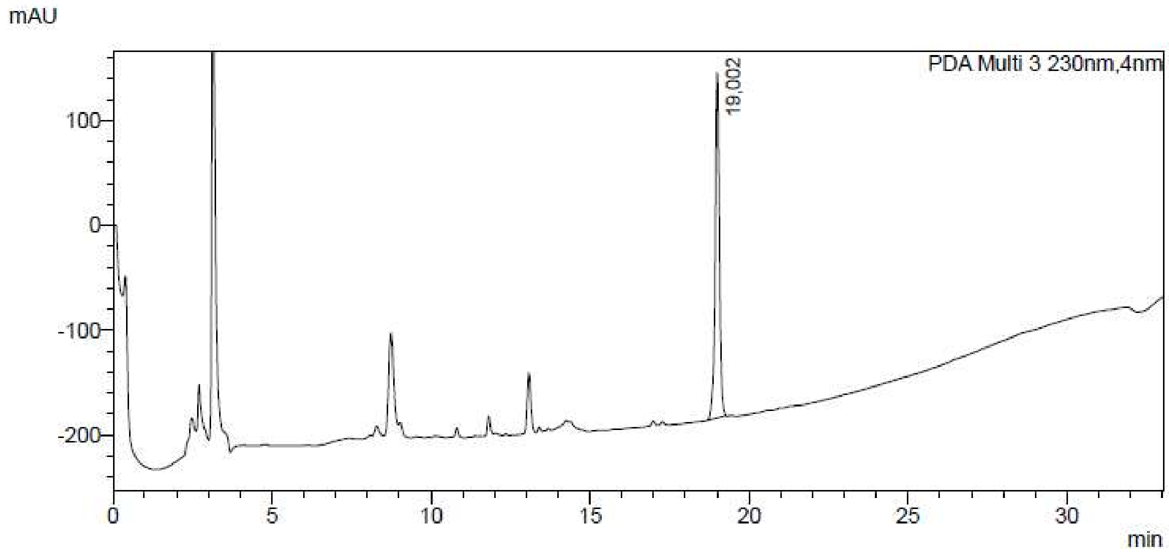

PDA Ch3 230nm

Peak#	Ret. Time	Area	Height	Conc.	Unit	Mark	Name
1	19,002	2968695	328329	0,000		M	
Total		2968695	328329				

*Figure S50: RP-HPLC-UV chromatogram of biocatalytic reduction of ethyl (Z)-2-(methoxyimino)-3-oxobutanoate (**Z-7a**) to pyrazine **3a** catalyzed by OYE3, showing the pyrazine at 19.00 min.*

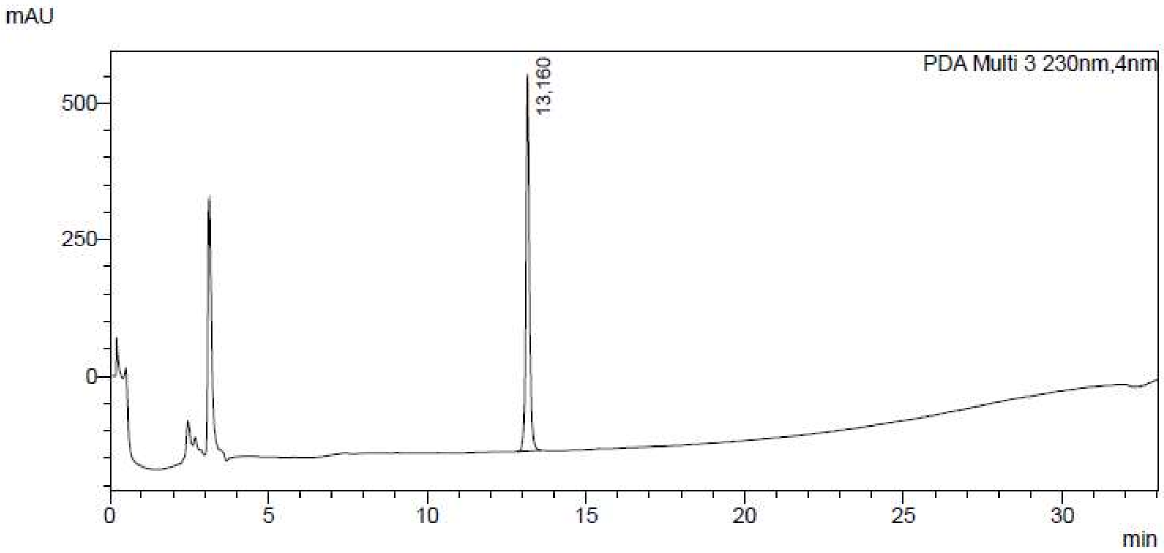

PDA Ch3 230nm

Peak#	Ret. Time	Area	Height	Conc.	Unit	Mark	Name
1	13,160	5493016	690485	0,000		M	
Total		5493016	690485				

*Figure S51: RP-HPLC-UV chromatogram of attempted biocatalytic reduction of ethyl 2-(methoxyamino)-3-oxobutanoate (**8a**) to pyrazine **3a** catalyzed by OYE3, showing only the hydroxylamine starting material (13.16 min).*

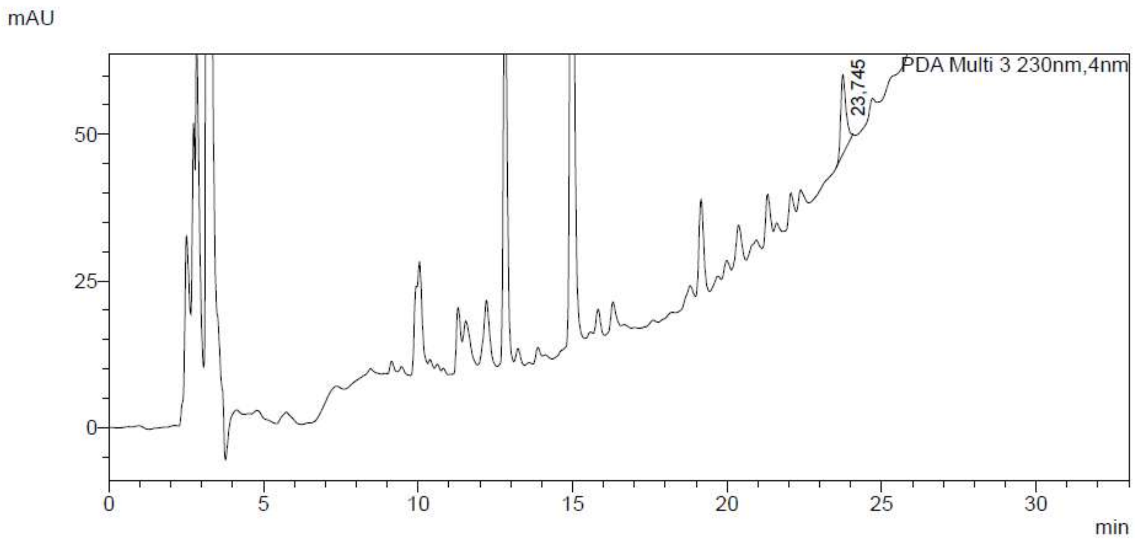

PDA Ch3 230nm

Peak#	Ret. Time	Area	Height	Conc.	Unit	Mark	Name
1	23,745	161006	13488	0,000		M	
Total		161006	13488				

*Figure S52: RP-HPLC-UV chromatogram of biocatalytic reduction of ethyl (Z)-2-(methoxyimino)-3-oxopentanoate) (**Z-7b**) to pyrazine **3b** (23.745 min) using XenA_WT.*

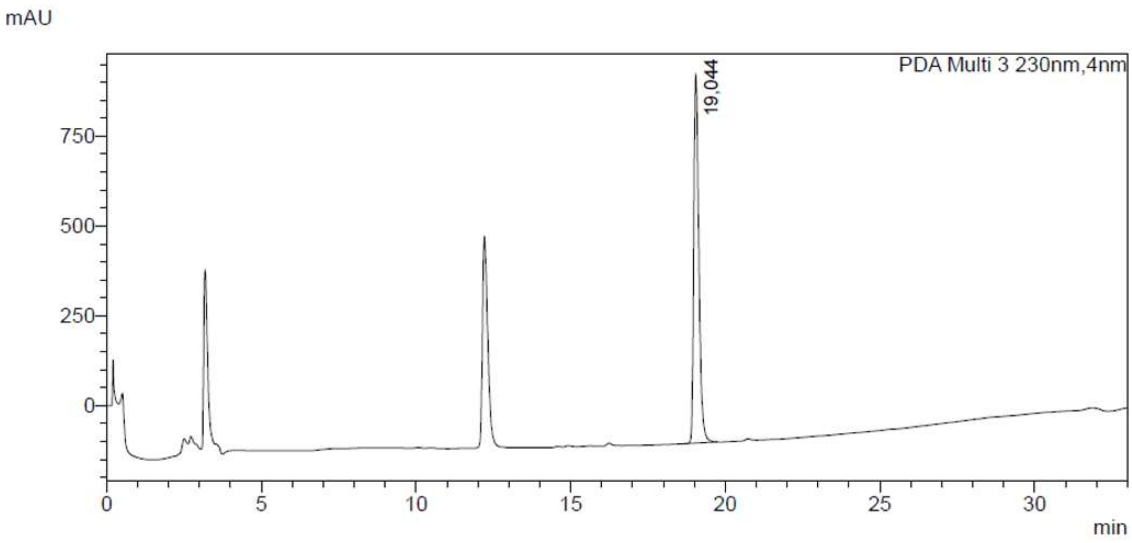

PDA Ch3 230nm

Peak#	Ret. Time	Area	Height	Conc.	Unit	Mark	Name
1	19,044	11723098	1025098	100,000		M	
Total		11723098	1025098				

*Figure S53: RP-HPLC-UV chromatogram of unsuccessful biocatalytic reduction of ethyl (E)-2-(methoxyimino)-3-oxopentanoate) (**E-7b**) to pyrazine **3b** using XenA_WT, showing only the starting material (19.04 min).*

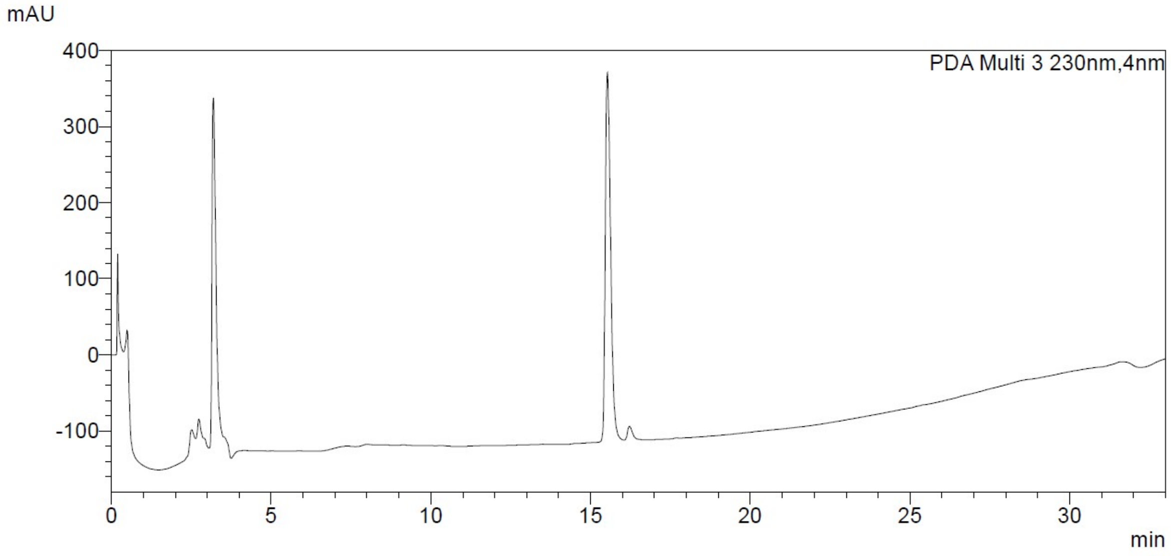

*Figure S54: RP-HPLC-UV chromatogram of unsuccessful biocatalytic reduction of ethyl 2-(methoxyamino)-3-oxopentanoate (**8b**) to pyrazine **3b** using XenA_WT, showing only the starting material (15.49 min).*

α-Oximo-ß-hydroxy esters

For reactions forming α-oximo-ß-hydroxy esters, analysis was done by RP-HPLC-UV using a Shimadzu HPLC system (Communication Bus Module CBM-20 A, Column Oven CTO-20 AC, Degasser DGU-20 A5, Liquid Chromatograph LC-20 AD, Auto sampler SIL-20 AC HT, Diode Array Detector SPD-M20 A) using a Phenomenex Luna® 5 µm C18(2) 100 Å column (250 x 4.6 mm) eluting with the following gradient of acetonitrile in water (both containing 0.1% TFA): 10% MeCN for two minutes, linear gradient to 60% MeCN over 26 minutes, linear gradient to 100% MeCN over five minutes, then straight drop to 10% MeCN, hold at 10% MeCN for three minutes. Flow rate: 1 mL/min, column oven temperature 25 °C, wavelength used for analysis: 230 nm. The retention times can be found in Table S29. A representative chromatogram can be found in Figure S57.

Table S29: Retention times on RP-HPLC-UV

Name	Structure	Retention time (minutes)	Chromatogram
2-methoxyethyl 2-(hydroxyimino)-3-oxobutanoate (**1c**)	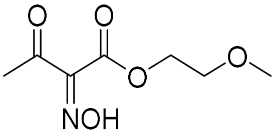	13.198	Figure S55
2-methoxyethyl 3-hydroxy-2-(hydroxyimino)butanoate, (**S1**)	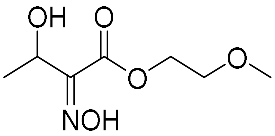	7.83 and 8.96	Figure S56
ethyl 2-(hydroxyimino)-3-oxopentanoate (**1b**)	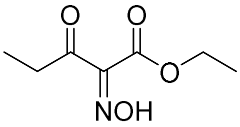	13.22 and 15.06	Figure S58
ethyl 3-hydroxy-2-(hydroxyimino)pentanoate	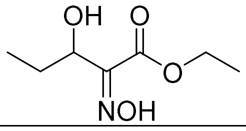	10.30 and 11.96	Figure S59

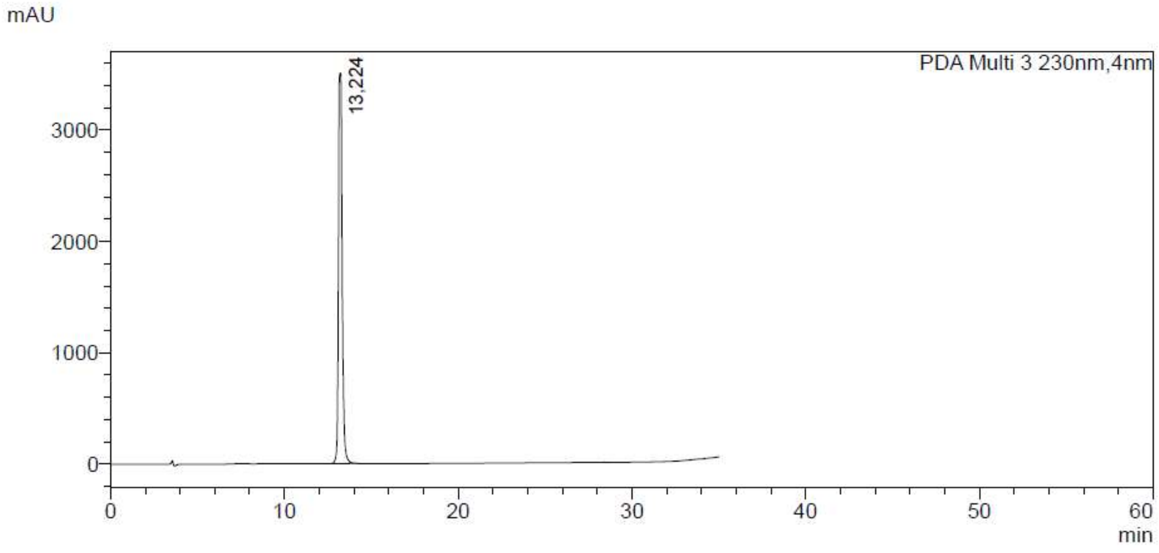

PDA Ch3 230nm

Peak#	Ret. Time	Area	Height	Conc.	Unit	Mark	Name
1	13,224	53027381	3505919	0,000		M	
Total		53027381	3505919				

*Figure S55: RP-HPLC-UV chromatogram of 2-methoxyethyl 2-(hydroxyimino)-3-oxobutanoate (oxime **1c**)*

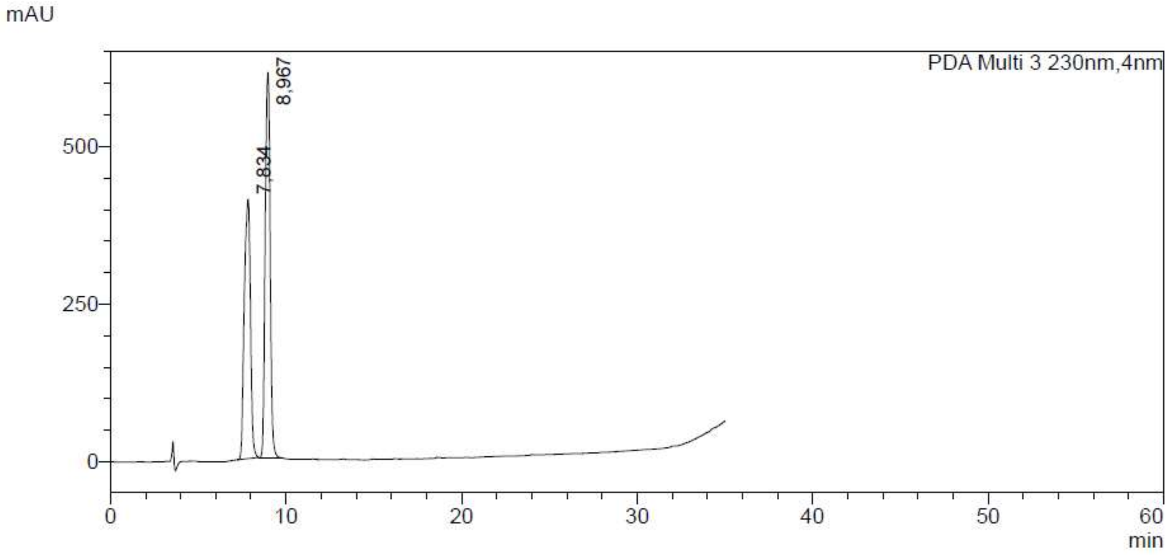

PDA Ch3 230nm

Peak#	Ret. Time	Area	Height	Conc.	Unit	Mark	Name
1	7,834	9979749	411147	0,000		M	
2	8,967	11988000	609651	0,000		M	
Total		21967749	1020798				

*Figure S56: RP-HPLC-UV chromatogram of E and Z 2-methoxyethyl 3-hydroxy-2-(hydroxyimino)butanoate **S1** (peaks not assigned to individual isomers).*

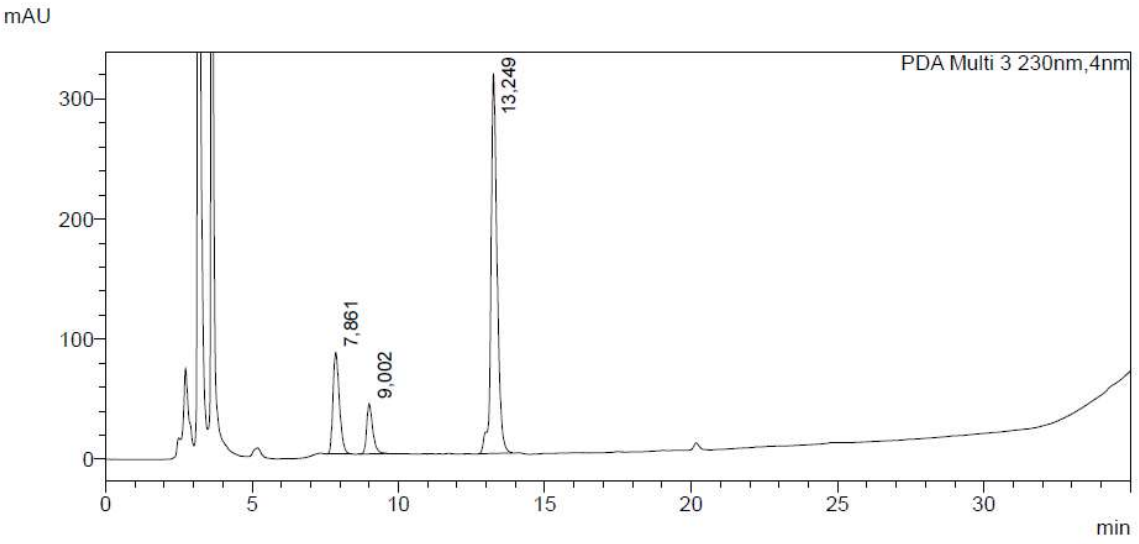

PDA Ch3 230nm

Peak#	Ret. Time	Area	Height	Conc.	Unit	Mark	Name
1	7,861	1291622	84537	0,000		M	
2	9,002	595731	41662	0,000		M	
3	13,249	4591942	316299	0,000		M	

*Figure S57: RP-HPLC-UV chromatogram of biocatalytic synthesis of E and Z 2-methoxyethyl 3-hydroxy-2-(hydroxyimino)butanoate **S1** (7.86 and 9.00 min) from 2-methoxyethyl 2-(hydroxyimino)-3-oxobutanoate (oxime **1c**, 13.25 min)) catalyzed by ADH-A.*

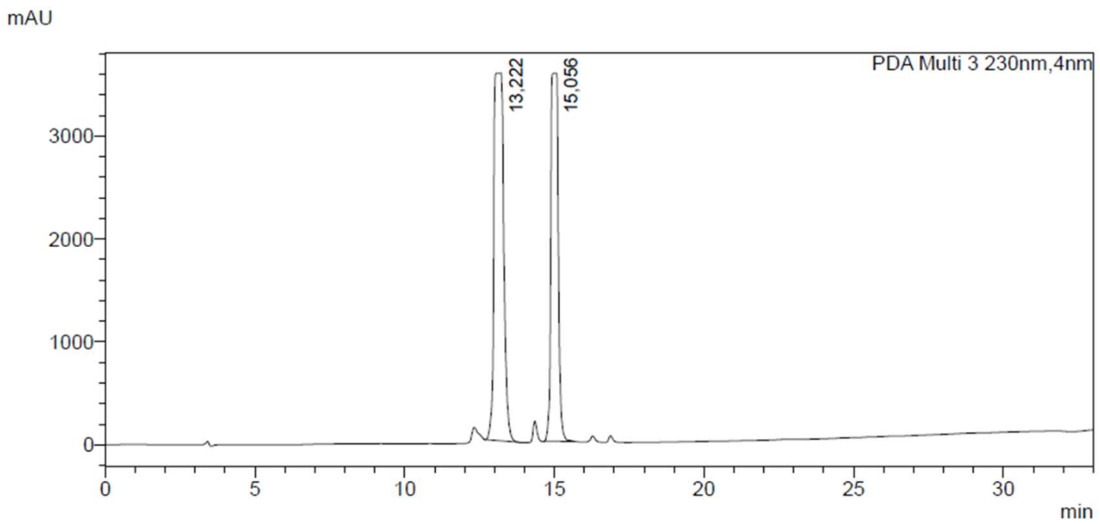

PDA Ch3 230nm

Peak#	Ret. Time	Area	Height	Conc.	Unit	Mark	Name
1	13,222	80806392	3574101	0,000		M	
2	15,056	61315130	3577644	0,000		M	
Total		142121522	7151745				

*Figure S58: HPLC-UV chromatogram of oxime **1b***

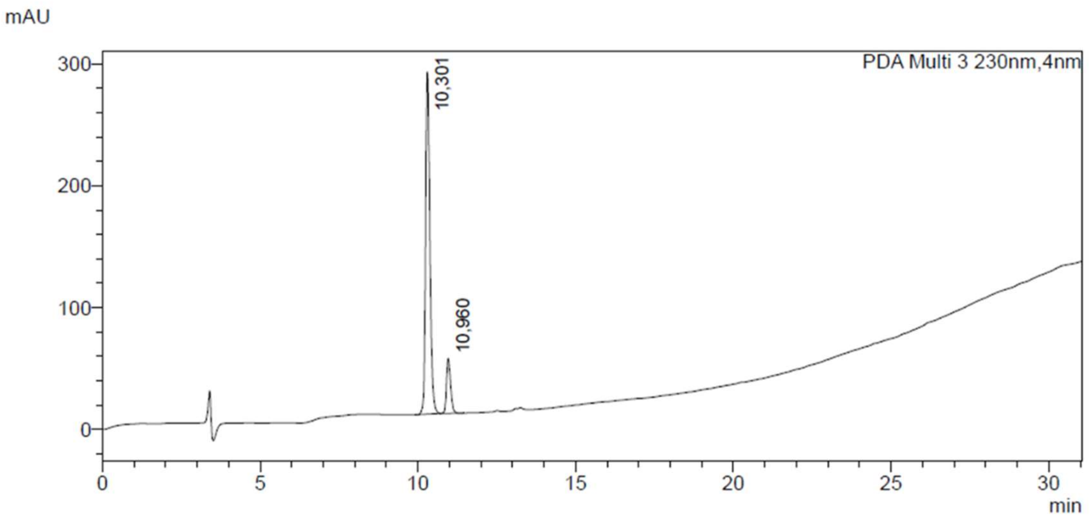

PDA Ch3 230nm

Peak#	Ret. Time	Area	Height	Conc.	Unit	Mark	Name
1	10,301	2654199	281184	0,000		M	
2	10,960	403666	45107	0,000		M	
Total		3057865	326291				

Figure S59: HPLC-UV chromatogram of oxime ethyl 3-hydroxy-2-(hydroxyimino)pentanoate

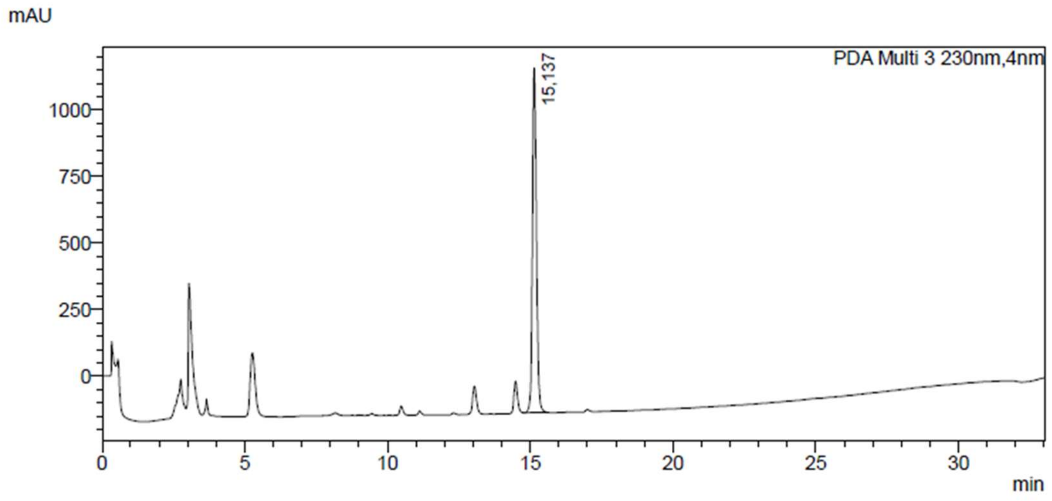

PDA Ch3 230nm

Peak#	Ret. Time	Area	Height	Conc.	Unit	Mark	Name
1	15,137	12447707	1296881	0,000		M	
Total		12447707	1296881				

*Figure S60: HPLC-UV chromatogram of attempted biocatalytic reduction of **93h** with Lica-ADH, showing only the starting material*

HPLC-MS

The (reversed-phase) HPLC-UV analytics of biotransformations gave ambiguous results in some cases. These experiments were repeated and analysis was done using HPLC-MS. Furthermore, new compounds were analyzed by HPLC-MS.

Low resolution mass spectra were recorded on an Agilent Technologies 6120 Quadrupole LC/MS detector in combination with an Agilent Technologies 1260 Infinity HPLC system, equipped with a Phenomenex Luna 5 µM C18(2) 100Å column (50 x 4.6 mm). Water/acetonitrile (+0.1 vol-% of formic acid) was used as eluent. The following gradient of water to acetonitrile was used: 0% MeCN for 1.5 minutes, linear gradient to 70% MeCN over 13.5 minutes, linear gradient to 80% MeCN over five minutes, hold at 80% MeCN for two minutes, then straight drop to 0% MeCN, hold at 0% MeCN for three minutes. Total time: 25 minutes. Relevant information on the reference compounds used can be found in Table S30, the HPLC-MS chromatograms and mass spectra of relevant peaks can be found in Figure S61 through Figure S72. HPLC-MS chromatograms and spectra of reactions that gave ambiguous results on HPLC-UV can be found in Figure S73 through Figure S76.

Table S30: Names, structures retention times and mass to charge ratios of the reference compounds analyzed using LC-MS

Compound	Structure	Retention time (minutes)	m/z (found)	m/z (calculated)	Spectrum and chromatogram
**2a**	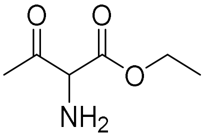	4.2	146.1	146.08 (M+H)	Figure S61
**2b**	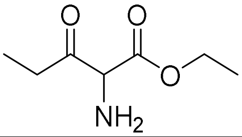	7.65	160.2	160.10 (M+H)	Figure S62
***N*-Bz-4b** (racemic)	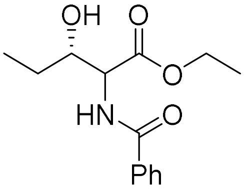	14.48	266.2 (M+H)	266.14 (M+H)	Figure S63
***N*-Bz-4b** (enantio-enriched)	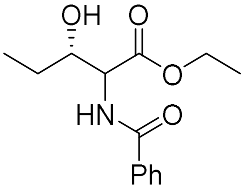	14.75	266.2 (M+H)	266.14 (M+H)	Figure S64
***N*-Bz-4b** (enzymatic synthesis)	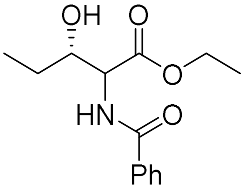	14.705 & 14.995	266.2 (M+H)	266.14 (M+H)	Figure S65
***N*-Bz-D-4c**	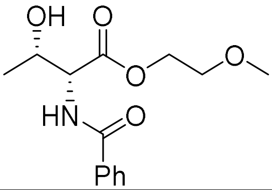	13.053	282.2 (M+H)	282.13 (M+H)	Figure S66

***N*-Bz-D-allo-4c**	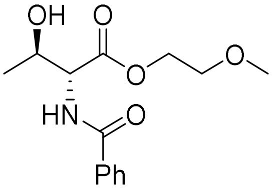	12.65	282.2 (M+H)	282.13 (M+H)	Figure S67
***N*-Bz-L-4c**	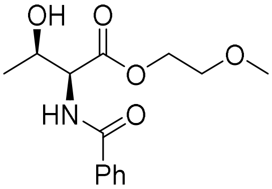	12.89	282.2 (M+H)	282.13 (M+H)	Figure S68
***N*-Bz-L-allo-4c**	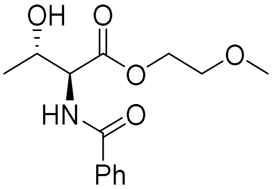	12.65	282.2 (M+H)	282.13 (M+H)	Figure S69
**8a**	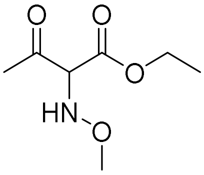	13.19	176.1 (M+H)	176.09 (M+H)	Figure S70
**8b**	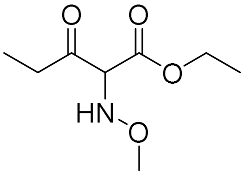	14.705	190.2 (m+H)	190.11 (M+H)	Figure S71
**S1**	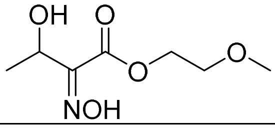	9.876 & 10.262	192.1 (M+H)	192.01 (M+H)	Figure S72

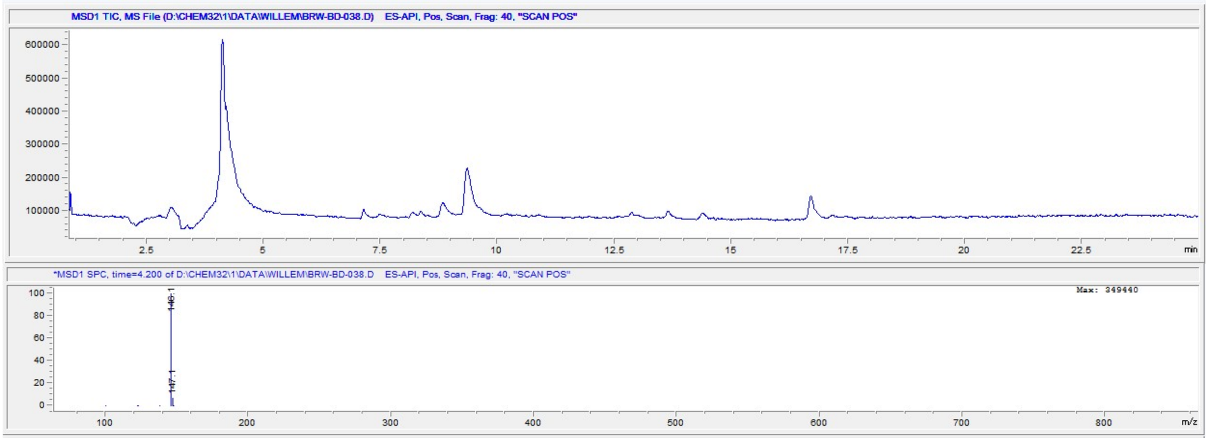

*Figure S61: HPLC-MS chromatogram and mass spectrum of **2a***

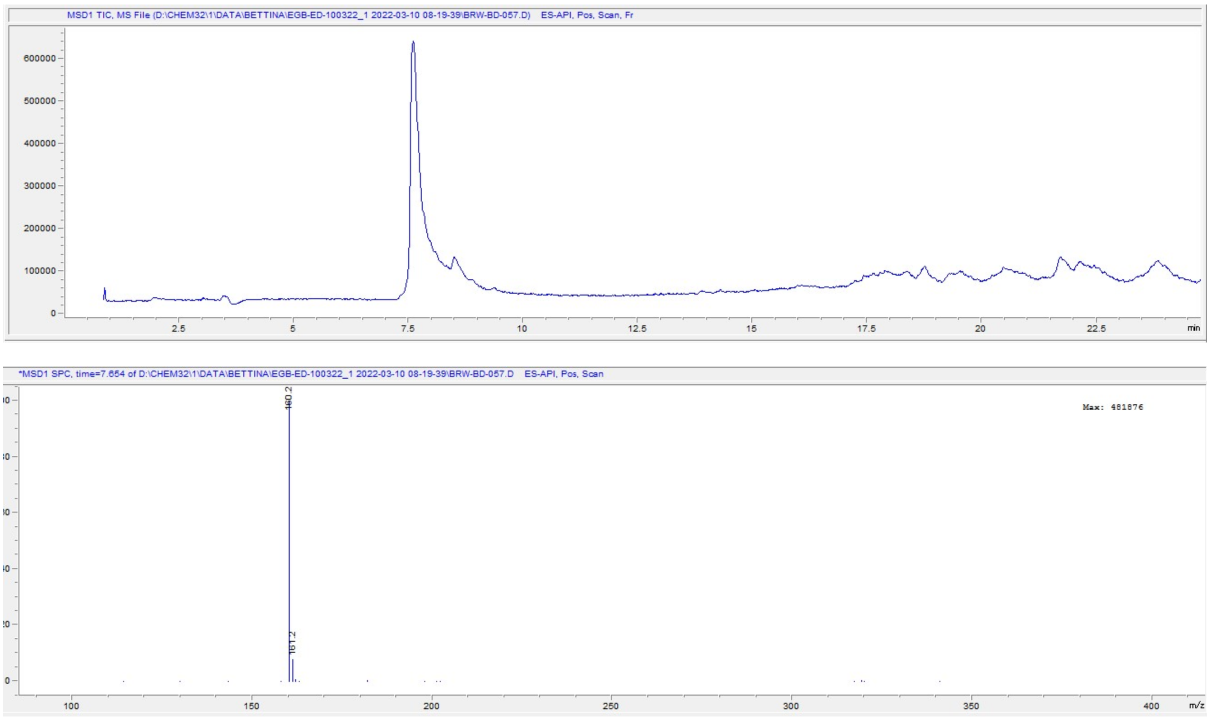

*Figure S62 HPLC-MS chromatogram and mass spectrum of **2b***

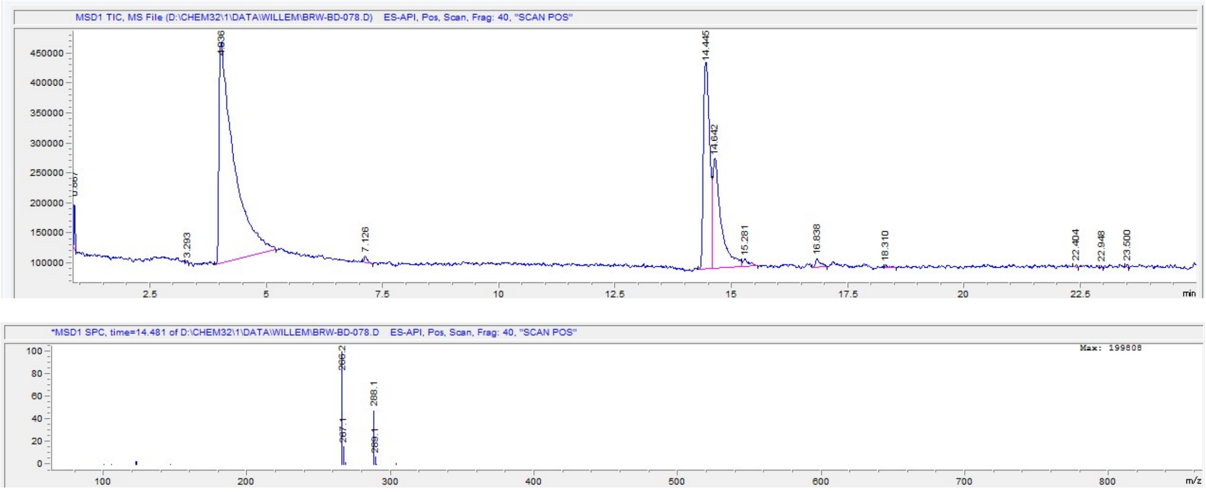

Figure S63: HPLC-MS chromatogram and mass spectrum of racemic **N-*Bz-4b***

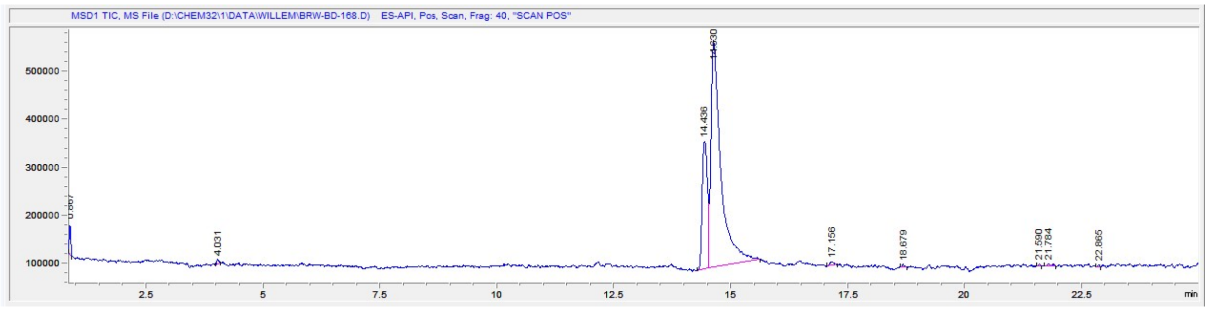

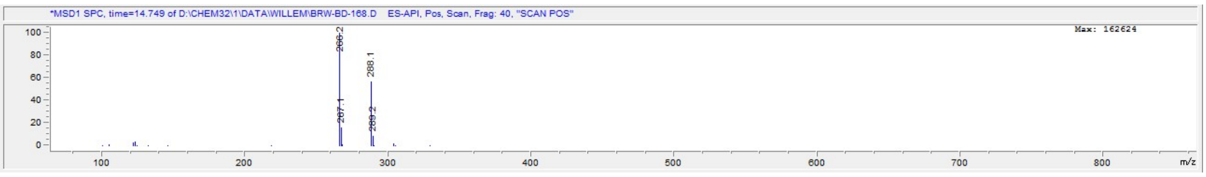

Figure S64: HPLC-MS chromatogram and mass spectrum of enantioenriched **N-*Bz-4b***

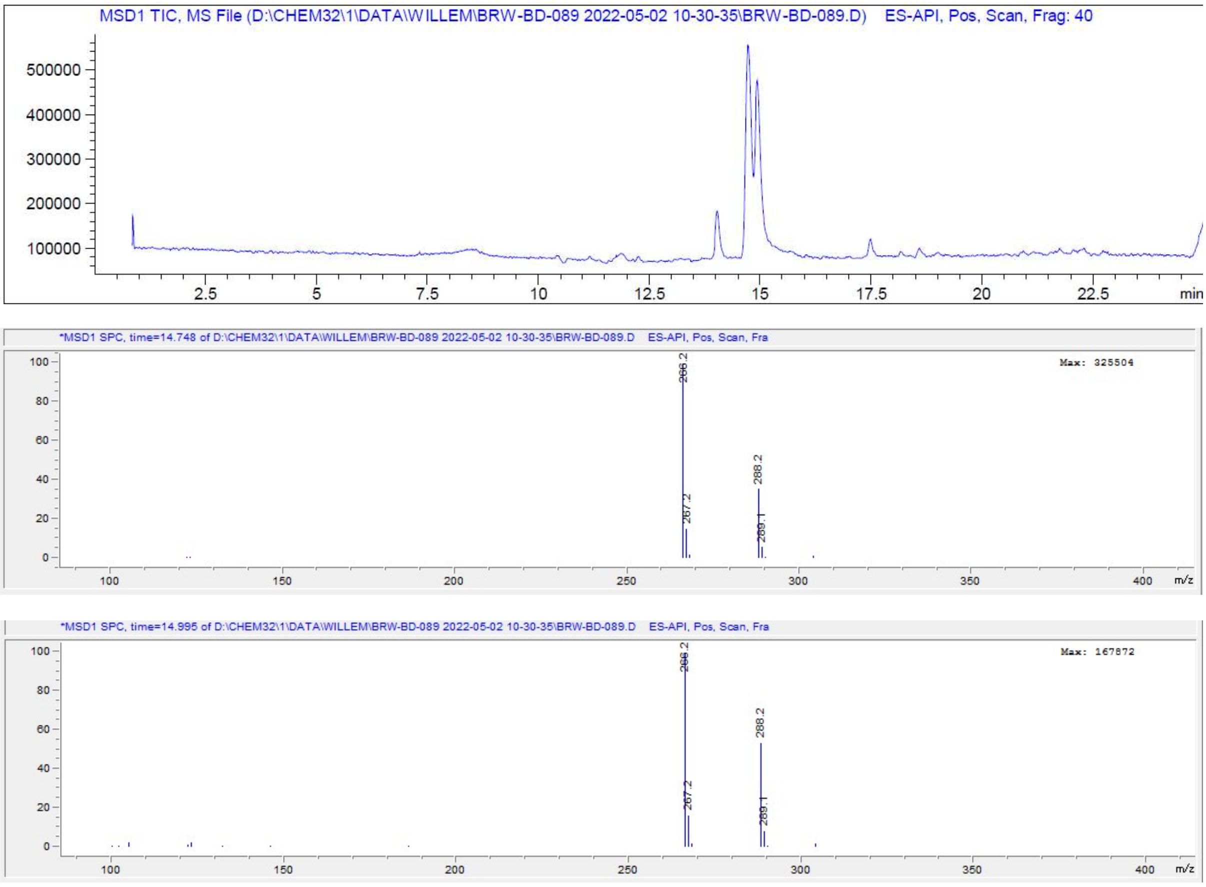

Figure S65: HPLC-MS chromatogram of ethyl 2-benzamido-3-hydroxypentanoate N-Bz-4b (14.75 and 15.00 min) synthesized chemoenzymatically from oxime 1b, catalyzed by XenA-WT and Lk*ADH-Lica.*

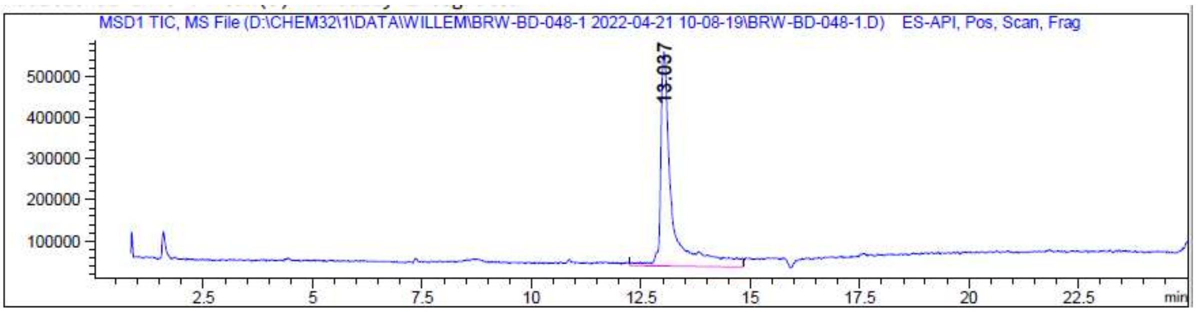

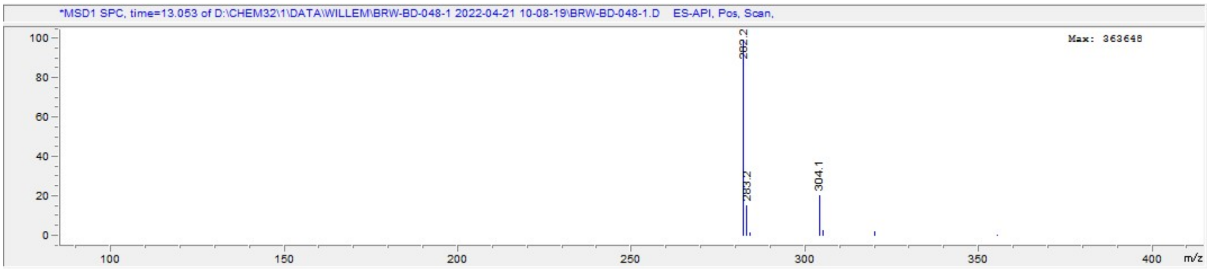

Figure S66: HPLC-MS chromatogram and mass spectrum of synthetic 2-methoxyethyl benzoyl-D-threoninate (**N-Bz-4c**)

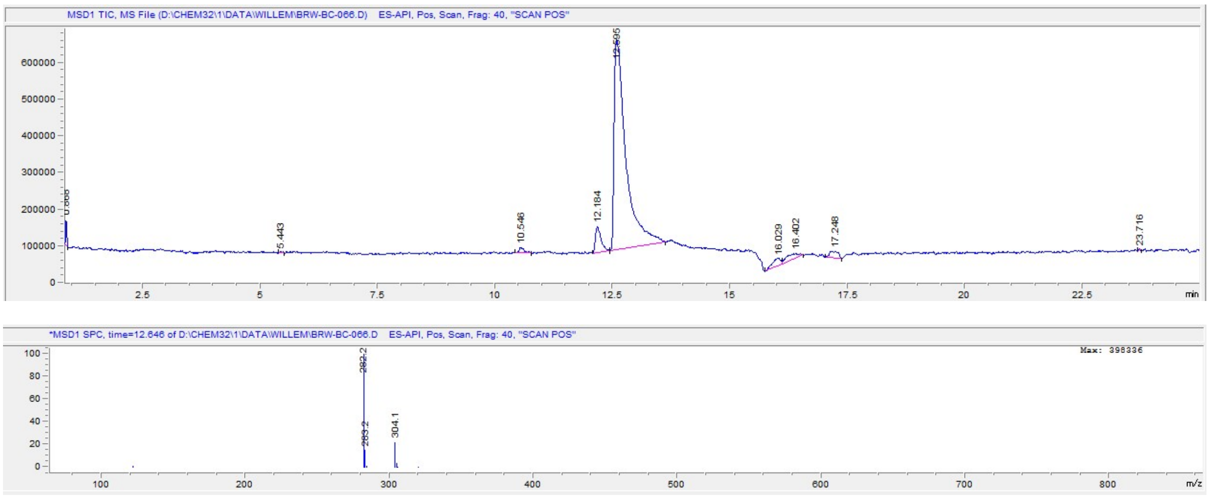

Figure S67: HPLC-MS chromatogram and mass spectrum of synthetic 2-methoxyethyl benzoyl-D-allo-threoninate (**N-Bz-4c**)

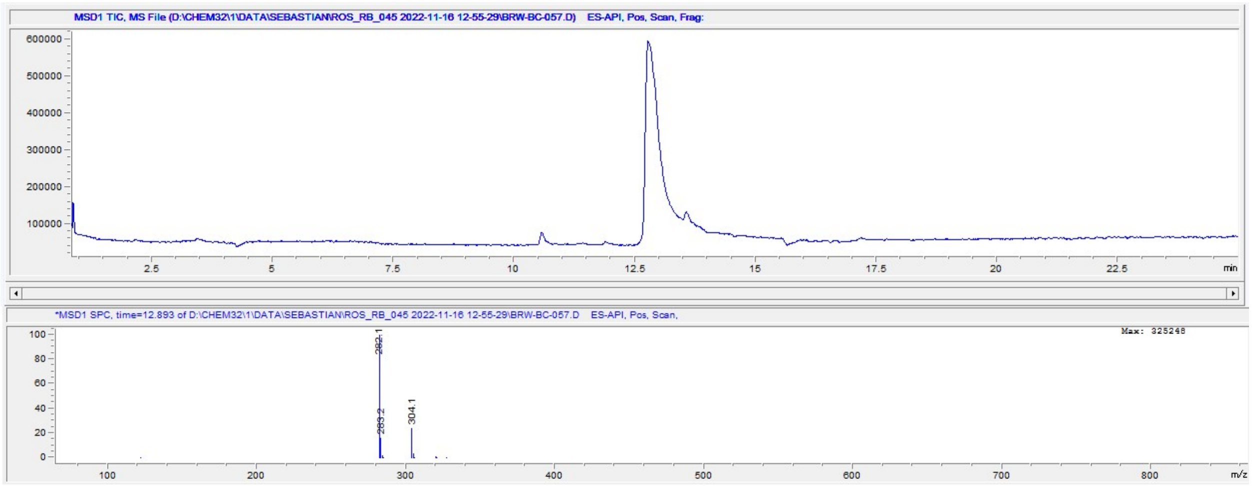

Figure S68: HPLC-MS chromatogram and mass spectrum of synthetic 2-methoxyethyl benzoyl-L-allo-threoninate (**N-Bz-4c**)

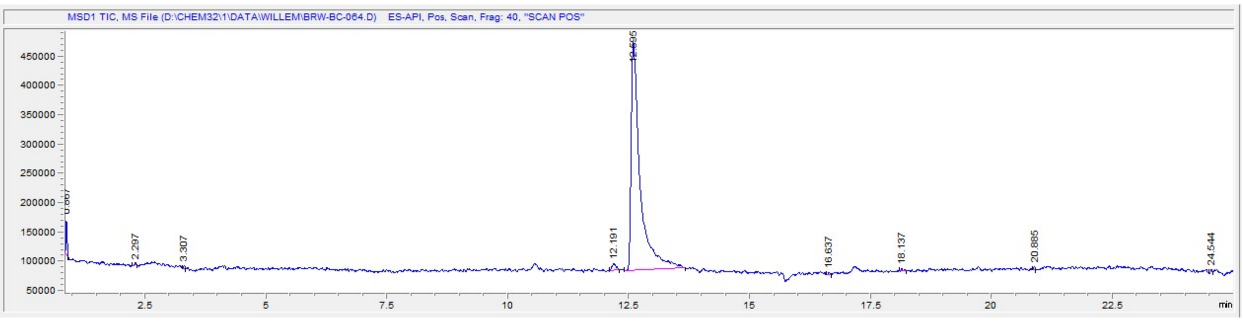

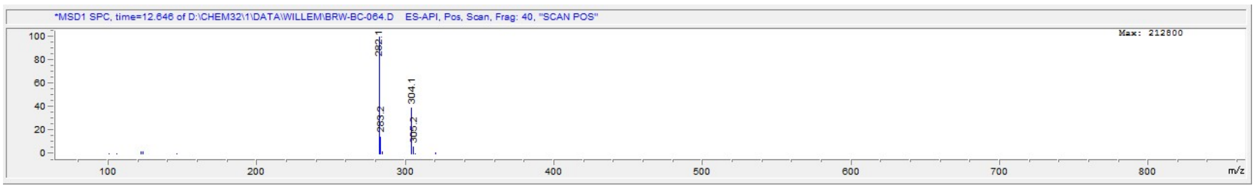

*Figure S69: HPLC-MS chromatogram and mass spectrum of synthetic 2-methoxyethyl benzoyl-L-allo-threoninate (**N-Bz-4c**)*

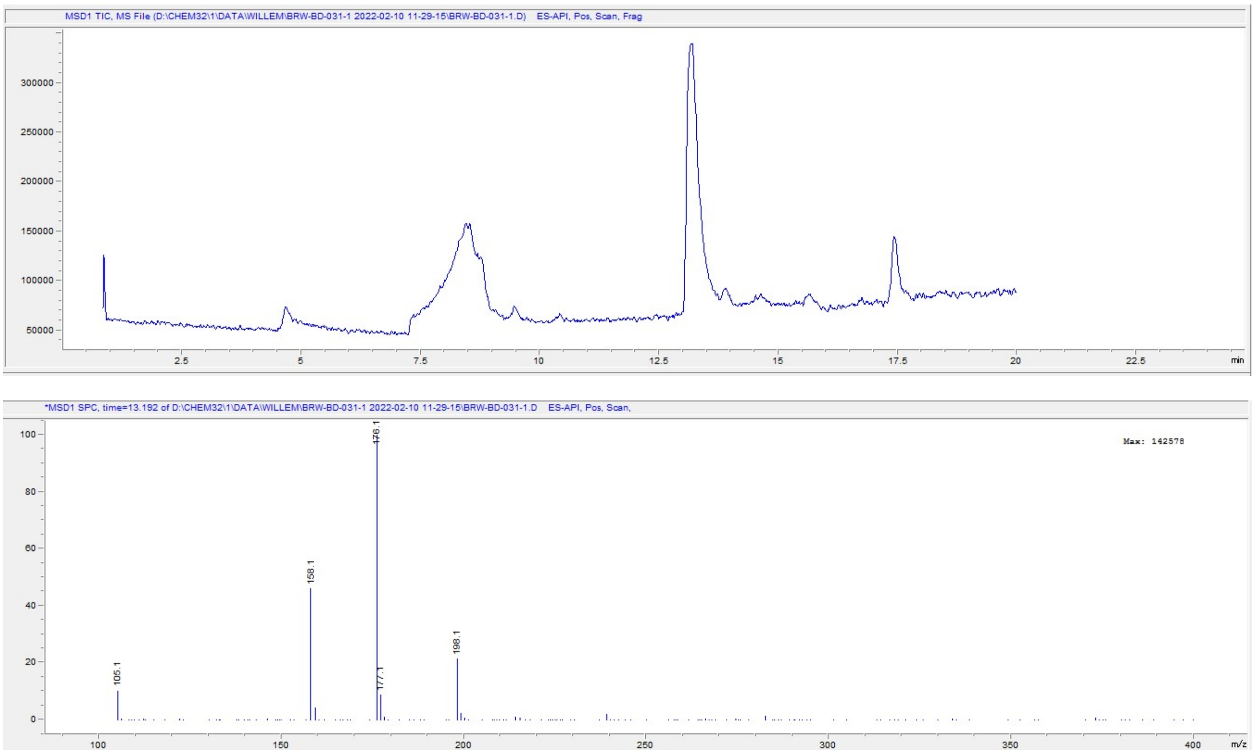

*Figure S70: HPLC-MS chromatogram and mass spectrum of **8a***

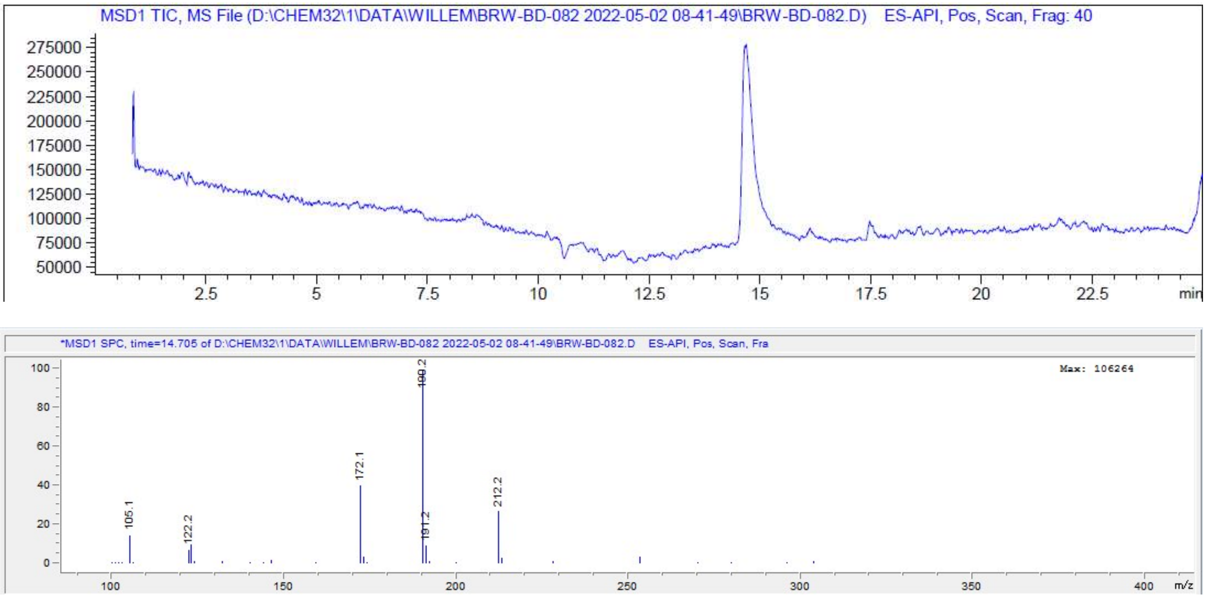

*Figure S71: HPLC-MS chromatogram and mass spectrum of synthetic ethyl 2-(methoxyamino)-3-oxopentanoate **8b***

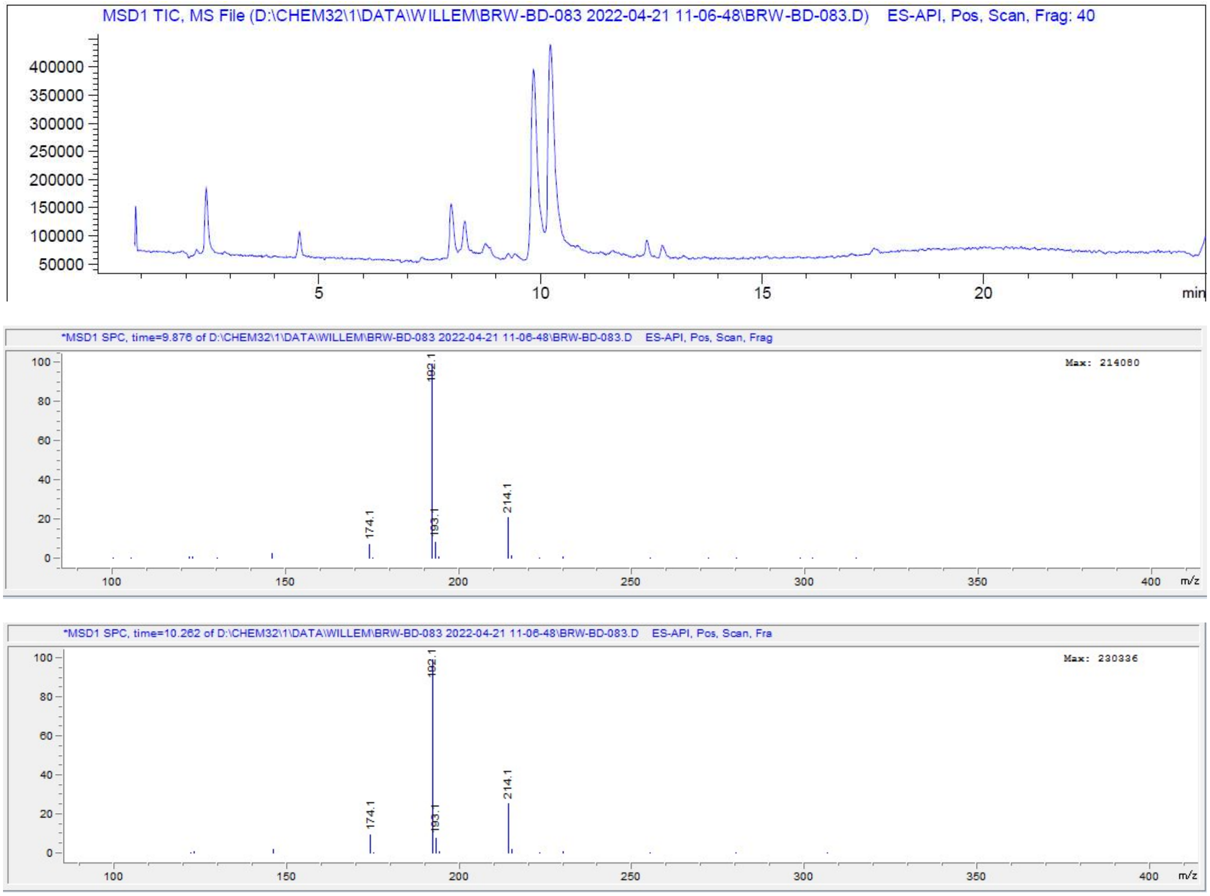

*Figure S72: HPLC-MS chromatogram and mass spectra (E/Z isomers) of 2-methoxyethyl 3-hydroxy-2-(hydroxyimino) butanoate **S1***

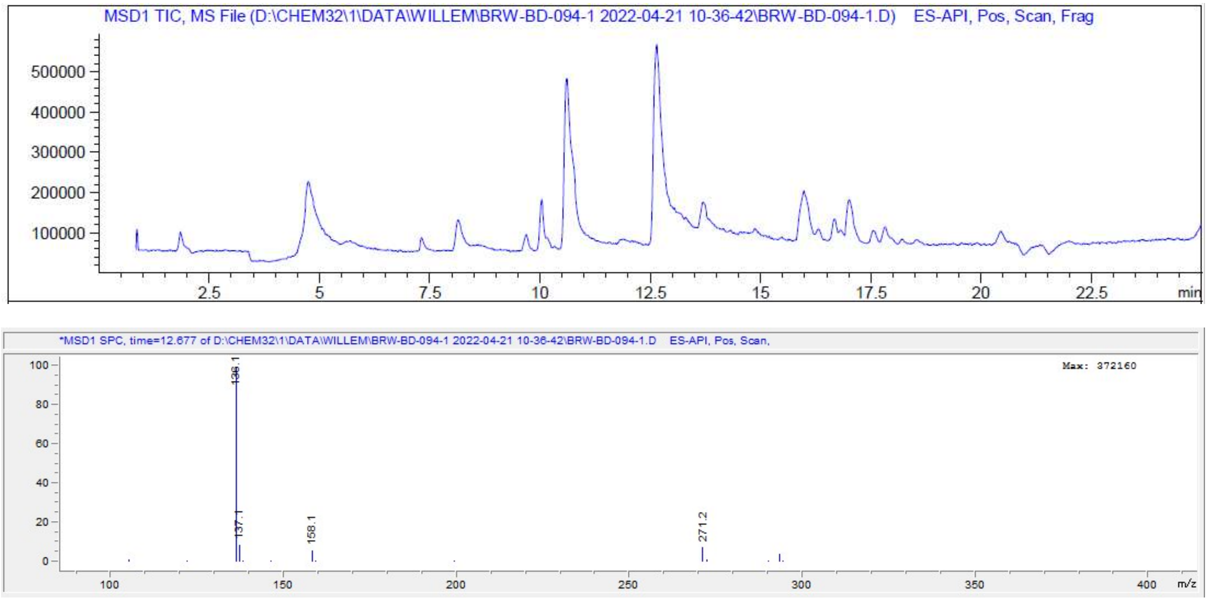

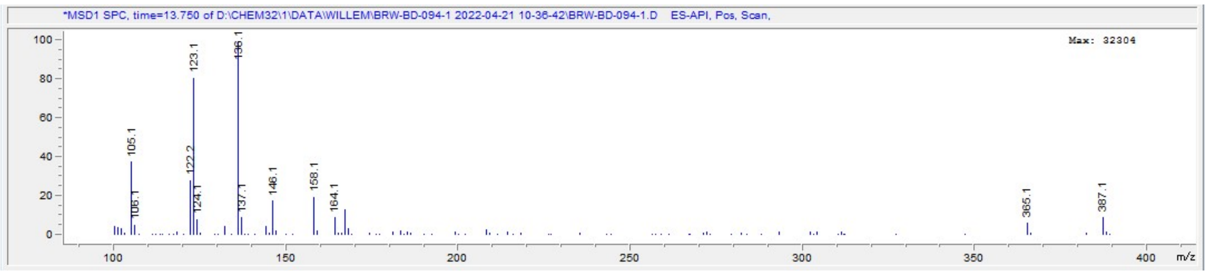

Figure S73: *HPLC-MS chromatogram (top) of attempted chemoenzymatic synthesis of 2-methoxyethyl benzoyl-D-threoninate from 2 -methoxyethyl 3-hydroxy-2-(hydroxyimino)butanoate* ***S1*** *using OYE3 and mass spectra of the peaks fond at 12.677 (middle) and 13.750 (bottom) minutes, showing that 2 -methoxyethyl 3-hydroxy-2-(hydroxyimino)butanoate* ***S1*** *is not a substrate for OYE3, as 2-methoxyethyl benzoyl-D-threoninate* ***N-Bz-4c*** *is not found.*

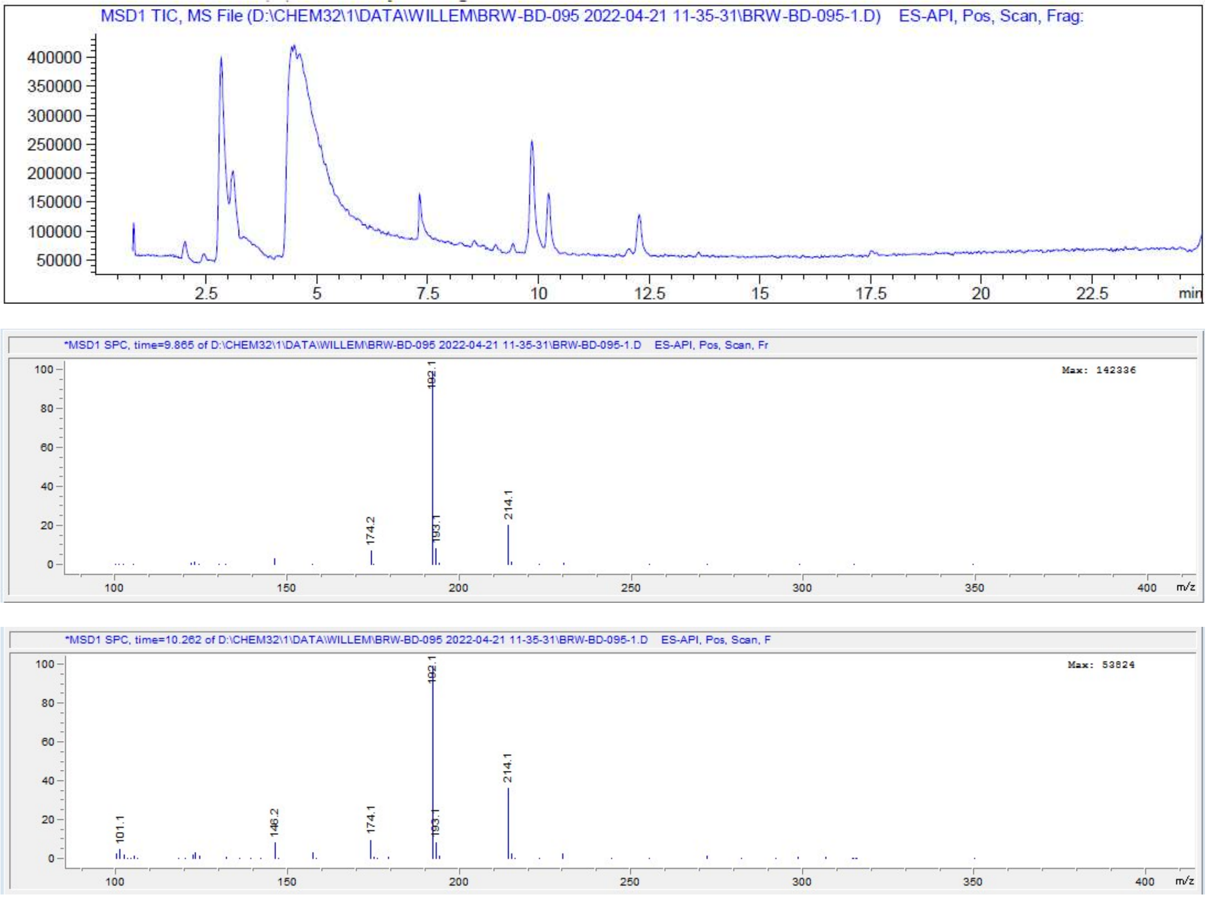

Figure S74: *HPLC-MS chromatogram and mass spectra at 9.865 and 10.262 min of 2-methoxyethyl 3-hydroxy-2-(hydroximino)butanoate* ***S1*** *obtained from reduction of oxime* ***1c*** *with ADH-A, proving that oxime* ***1c*** *is a substrate for ADH-A, which forms both E/Z isomers of the product.*

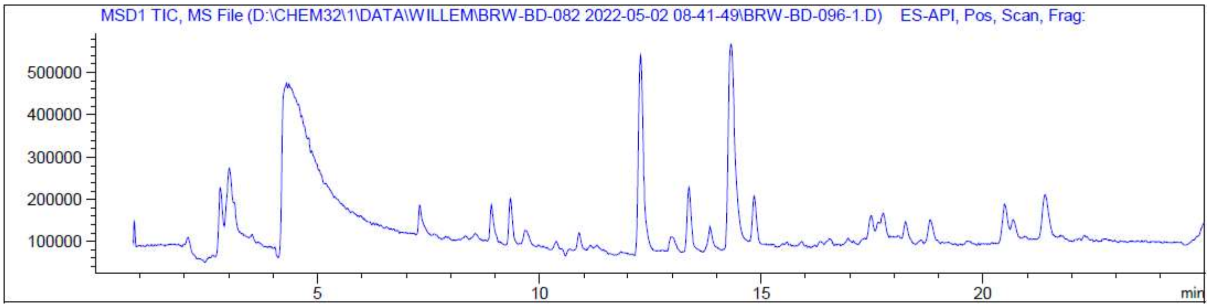

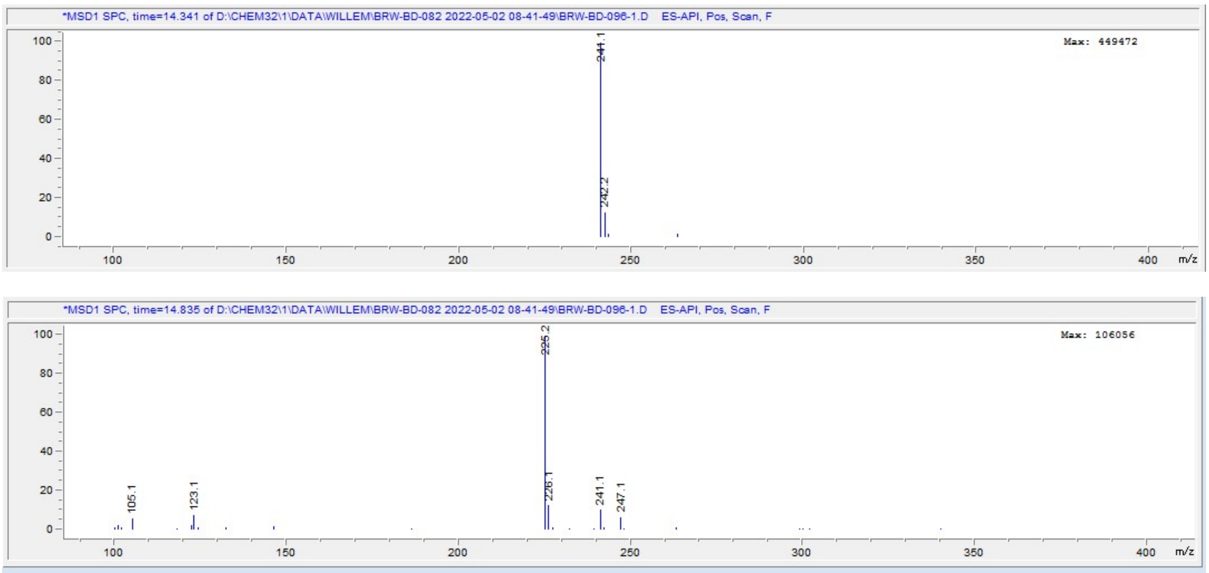

Figure S75: *HPLC-MS chromatogram of the attempted biocatalytic reduction of ethyl (Z) 2-(methoxyimino)-3-oxopentanoate* ***Z-7b*** *with XenA_WT as catalyst, and mass spectra at 14.341 and 14.835 minutes, proving that ethyl 2-(methoxyamino)-3-oxopentanoate* ***8b*** *is not formed.*

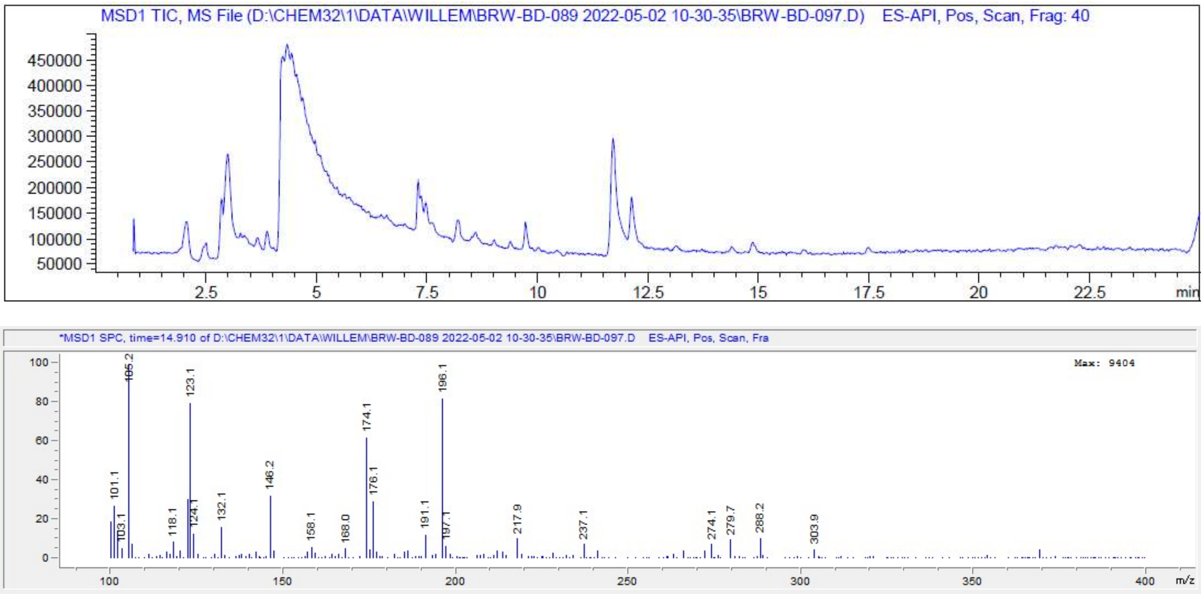

Figure S76: HPLC-MS chromatogram of the reduction of oxime 1b with LkADH-Lica *without ERED and mass spectrum of the peak at 14.910 minutes, proving that ethyl 2-benzamido 3-hydroxypentanoate is not formed with only* LkADH-Lica *as catalyst.*

References

(1) Velikogne, S.; Breukelaar, W. B.; Hamm, F.; Glabonjat, R. A.; Kroutil, W. C=C-Ene-Reductases Reduce the C=N Bond of Oximes. *ACS Catal.* **2020**, *10*, 13377–13382.

(2) Turrini, N. G.; Cioc, R. C.; Niet, D. J. H. van der; Ruijter, E.; Orru, R. V. A.; Hall, M.; Faber, K. Biocatalytic Access to Nonracemic γ-Oxo Esters via Stereoselective Reduction Using Ene-Reductases. *Green Chem.* **2017**, *19*, 511–518.

(3) Scholtissek, A.; Ullrich, S. R.; Mühling, M.; Schlömann, M.; Paul, C. E.; Tischler, D. A Thermophilic-like Ene-Reductase Originating from an Acidophilic Iron Oxidizer. *Appl. Microbiol. Biotechnol.* **2017**, *101*, 609–619.

(4) Gruber, C. C.; Oberdorfer, G.; Voss, C. V.; Kremsner, J. M.; Kappe, C. O.; Kroutil, W. An Algorithm for the Deconvolution of Mass Spectrosopic Patterns in Isotope Labeling Studies. Evaluation for the Hydrogen−Deuterium Exchange Reaction in Ketones. *J. Org. Chem.* **2007**, *72*, 5778–5783.

(5) Velikogne, S.; Resch, V.; Dertnig, C.; Schrittwieser, J. H.; Kroutil, W. Sequence-Based In-Silico Discovery, Characterisation, and Biocatalytic Application of a Set of Imine Reductases. *ChemCatChem* **2018**, *10*, 3236–3246.

(6) Weckbecker, A.; Hummel, W. Cloning, Expression, and Characterization of an (*R*)-Specific Alcohol Dehydrogenase from Lactobacillus Kefir. *Biocatal. Biotransformation* **2006**, *24*, 380–389.

(7) Borowiecki, P.; Telatycka, N.; Tataruch, M.; Żądło-Dobrowolska, A.; Reiter, T.; Schühle, K.; Heider, J.; Szaleniec, M.; Kroutil, W. Biocatalytic Asymmetric Reduction of γ-Keto Esters to Access Optically Active γ-Aryl-γ-Butyrolactones. *Adv. Synth. Catal.* **2020**, *362*, 2012–2029.

(8) Gohel, A.; Smith, D.; Wong, B.; Sukumaran, J.; Yeo, W. L.; Collier, S. J. Biocatalytic Process for Preparing Eslicarbazepine and Analogs Thereof. WO2012142302A2, 2012.

(9) Lavandera, I.; Kern, A.; Resch, V.; Ferreira-Silva, B.; Glieder, A.; Fabian, W. M. F.; De Wildeman, S.; Kroutil, W. One-Way Biohydrogen Transfer for Oxidation of Sec-Alcohols. *Org. Lett.* **2008**, *10*, 2155–2158.

(10) Lavandera, I.; Kern, A.; Ferreira-Silva, B.; Glieder, A.; De Wildeman, S.; Kroutil, W. Stereoselective Bioreduction of Bulky-Bulky Ketones by a Novel ADH from Ralstonia Sp. *J. Org. Chem.* **2008**, *73*, 6003–6005.

(11) Vonrhein, C.; Flensburg, C.; Keller, P.; Sharff, A.; Smart, O.; Paciorek, W.; Womack, T.; Bricogne, G. Data Processing and Analysis with the AutoPROC Toolbox. *Acta Crystallogr. Sect. D Biol. Crystallogr.* **2011**, *67*, 293–302.

(12) Glusker, J. P. Biological Crystallography. *Acta Crystallogr. Sect. D Biol. Crystallogr.* **1993**, *49*, 1–1.

(13) Evans, P. Scaling and Assessment of Data Quality. *Acta Crystallogr. Sect. D Biol. Crystallogr.* **2006**, *62*, 72–82.

(14) Evans, P. R.; Murshudov, G. N. How Good Are My Data and What Is the Resolution? *Acta Crystallogr. Sect. D Biol. Crystallogr.* **2013**, *69*, 1204–1214.

(15) Winn, M. D.; Ballard, C. C.; Cowtan, K. D.; Dodson, E. J.; Emsley, P.; Evans, P. R.; Keegan, R. M.; Krissinel, E. B.; Leslie, A. G. W.; McCoy, A.; et al. Overview of the CCP4 Suite and Current Developments. *Acta Crystallogr. Sect. D Biol. Crystallogr.* **2011**, *67*, 235–242.

(16) Schrödinger. Schrödinger Release 2022-2: Maestro, Schrödinger, LLC, New York, NY, 2021. 2022.

(17) Liebschner, D.; Afonine, P. V.; Baker, M. L.; Bunkoczi, G.; Chen, V. B.; Croll, T. I.; Hintze, B.; Hung, L. W.; Jain, S.; McCoy, A. J.; et al. Macromolecular Structure Determination Using X-Rays, Neutrons and Electrons: Recent Developments in Phenix. *Acta Crystallogr. Sect. D Struct. Biol.* **2019**, *75*, 861–877.

(18) Bowers, K. J.; Chow, E.; Xu, H.; Dror, R. O.; Eastwood, M. P.; Gregersen, B. A.; Klepeis, J. L.; Kolossvary, I.; Moraes, M. A.; Sacerdoti, F. D.; et al. Scalable Algorithms for Molecular Dynamics Simulations on Commodity Clusters. *Proc. 2006 ACM/IEEE Conf. Supercomput.*

SC'06 **2006**, No. November.

(19) Lu, C.; Wu, C.; Ghoreishi, D.; Chen, W.; Wang, L.; Damm, W.; Ross, G. A.; Dahlgren, M. K.; Russell, E.; Von Bargen, C. D.; et al. OPLS4: Improving Force Field Accuracy on Challenging Regimes of Chemical Space. *J. Chem. Theory Comput.* **2021**, *17*, 4291–4300.

(20) Lonsdale, R.; Reetz, M. T. Reduction of α,β-Unsaturated Ketones by Old Yellow Enzymes: Mechanistic Insights from Quantum Mechanics/Molecular Mechanics Calculations. *J. Am. Chem. Soc.* **2015**, *137*, 14733–14742.

(21) Fraaije, M. W.; Mattevi, A. Flavoenzymes: Diverse Catalysts with Recurrent Features. *Trends Biochem. Sci.* **2000**, *25*, 126–132.

(22) Benkovic, S. J.; Hammes-Schiffer, S. A Perspective on Enzyme Catalysis. *Science* **2003**, *301*, 1196–1202.

(23) Clark, A. J.; Tiwary, P.; Borrelli, K.; Feng, S.; Miller, E. B.; Abel, R.; Friesner, R. A.; Berne, B. J. Prediction of Protein–Ligand Binding Poses via a Combination of Induced Fit Docking and Metadynamics Simulations. *J. Chem. Theory Comput.* **2016**, *12*, 2990–2998.

(24) Corrêa, I. R.; Moran, P. J. S. Diastereoselective Reduction of E and Z α-Alkoxyimino-β-Ketoesters by Sodium Borohydride. *Tetrahedron* **1999**, *55*, 14221–14232.

(25) Tharra, P. R.; Mikhaylov, A. A.; Švejkar, J.; Gysin, M.; Hobbie, S. N.; Švenda, J. Short Synthesis of (+)-Actinobolin: Simple Entry to Complex Small-Molecule Inhibitors of Protein Synthesis. *Angew. Chemie Int. Ed.* **2022**, *61*, e202116520.

(26) Fell, S. C. M.; Pearson, M. J.; Burton, G.; Elder, J. S. Synthesis and Biological Activity of New C-6 and C-7 Substituted Vinyloxyimino-Penicillins and -Cephalosporins. *J. Chem. Soc. Perkin Trans. 1* **1995**, *12*, 1483–1493.

(27) Yokoyama, M.; Menjo, Y.; Watanabe, M.; Togo, H. Synthesis of Oxazoles and Thiazoles Using Thioimidates. *Synthesis* **1994**, *1994*, 1467–1470.

(28) Thalluri, K.; Nadimpally, K. C.; Chakravarty, M. P.; Paul, A.; Mandal, B. Ethyl 2-(Tert-Butoxycarbonyloxyimino)-2-Cyanoacetate (Boc-Oxyma) as Coupling Reagent for Racemization-Free Esterification, Thioesterification, Amidation and Peptide Synthesis. *Adv. Synth. Catal.* **2013**, *355*, 448–462.

(29) Zheng, L. S.; Férard, C.; Phansavath, P.; Ratovelomanana-Vidal, V. Rhodium-Mediated Asymmetric Transfer Hydrogenation: A Diastereo- and Enantioselective Synthesis of: Syn -α-Amido β-Hydroxy Esters. *Chem. Commun.* **2018**, *54*, 283–286.

(30) Shibata, Y.; Kagechika, K.; Yamaguchi, M.; Yoshikawa, K.; Chiba, K.; Takano, H.; Akiyama, C.; Ono, M.; Nishi, M.; Kubo, H.; et al. Synthesis and Structure-Activity Relationships of Novel Zwitterionic Compounds as Peroxisome Proliferator Activated Receptor α/γ Dual Agonists with Improved Physicochemical Properties. *Chem. Pharm. Bull* **2013**, *61*, 1248–1263.

(31) Vitale, R.; Ottonello, G.; Petracca, R.; Bertozzi, S. M.; Ponzano, S.; Armirotti, A.; Berteotti, A.; Dionisi, M.; Cavalli, A.; Piomelli, D.; et al. Synthesis, Structure-Activity, and Structure-Stability Relationships of 2-Substituted- *N* -(4-Oxo-3-Oxetanyl) *N* -Acylethanolamine Acid Amidase (NAAA) Inhibitors. *ChemMedChem* **2014**, *9*, 323–336.

(32) Blaskovich, M. A.; Lajoie, G. A. Synthesis of a Chiral Serine Aldehyde Equivalent and Its Conversion to Chiral .Alpha.-Amino Acid Derivatives. *J. Am. Chem. Soc.* **1993**, *115*, 5021–5030.

(33) Blaskovich, M. A.; Evindar, G.; Rose, N. G. W.; Wilkinson, S.; Luo, Y.; Lajoie, G. A. Stereoselective Synthesis of Threo and Erythro-Hydroxy and-Disubstituted-Hydroxy r-Amino Acids. *J. Org. Chem* **1998**, *63*, 3631–3646.

Appendix A: NMR spectra

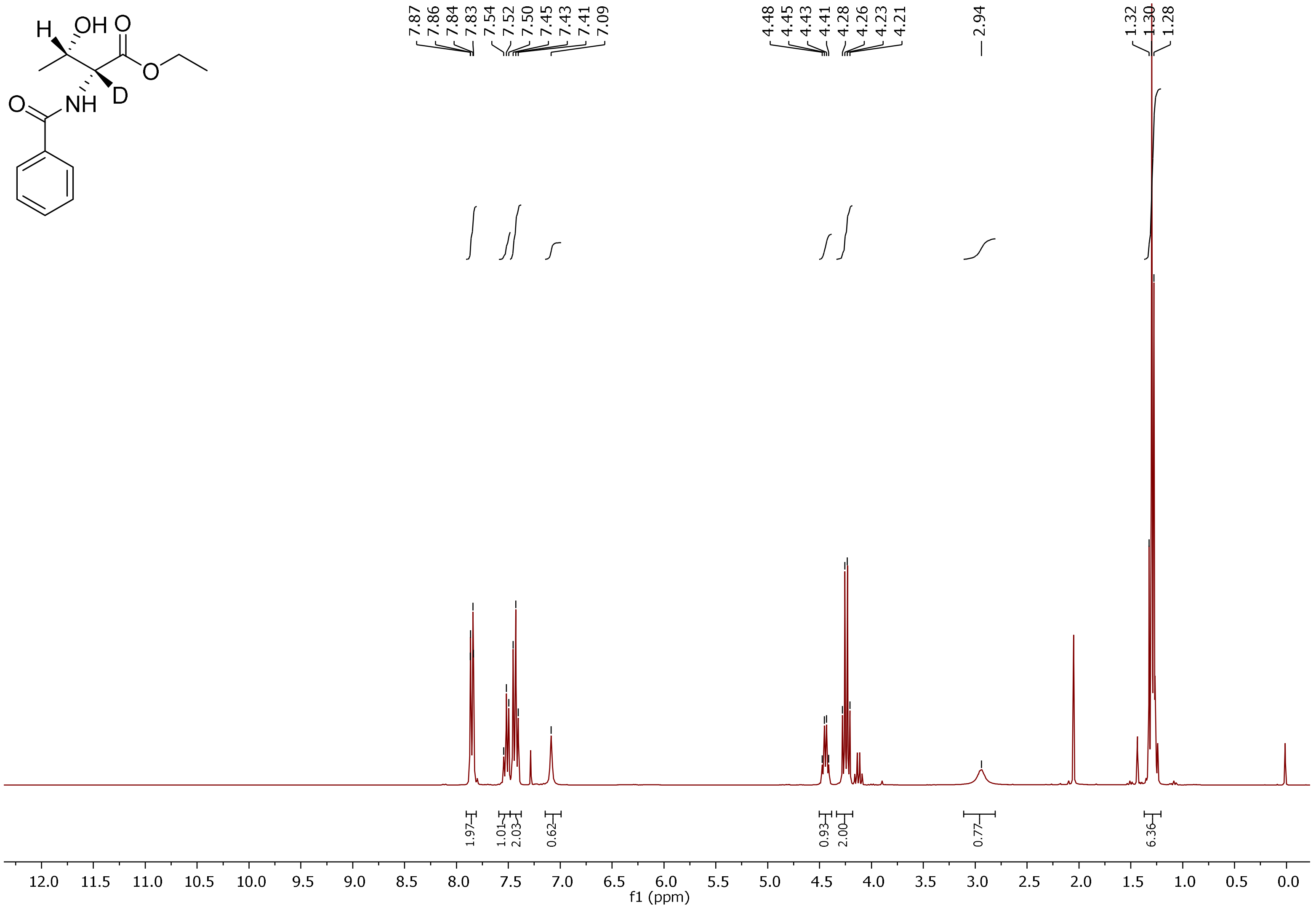

Figure S77: ¹H and ¹³C NMR spectra of ethyl benzoyl-D-threoninate-2-d, obtained from ADH-A catalyzed reduction of ethyl 2-amino-3-oxobutanoate hydrochloride

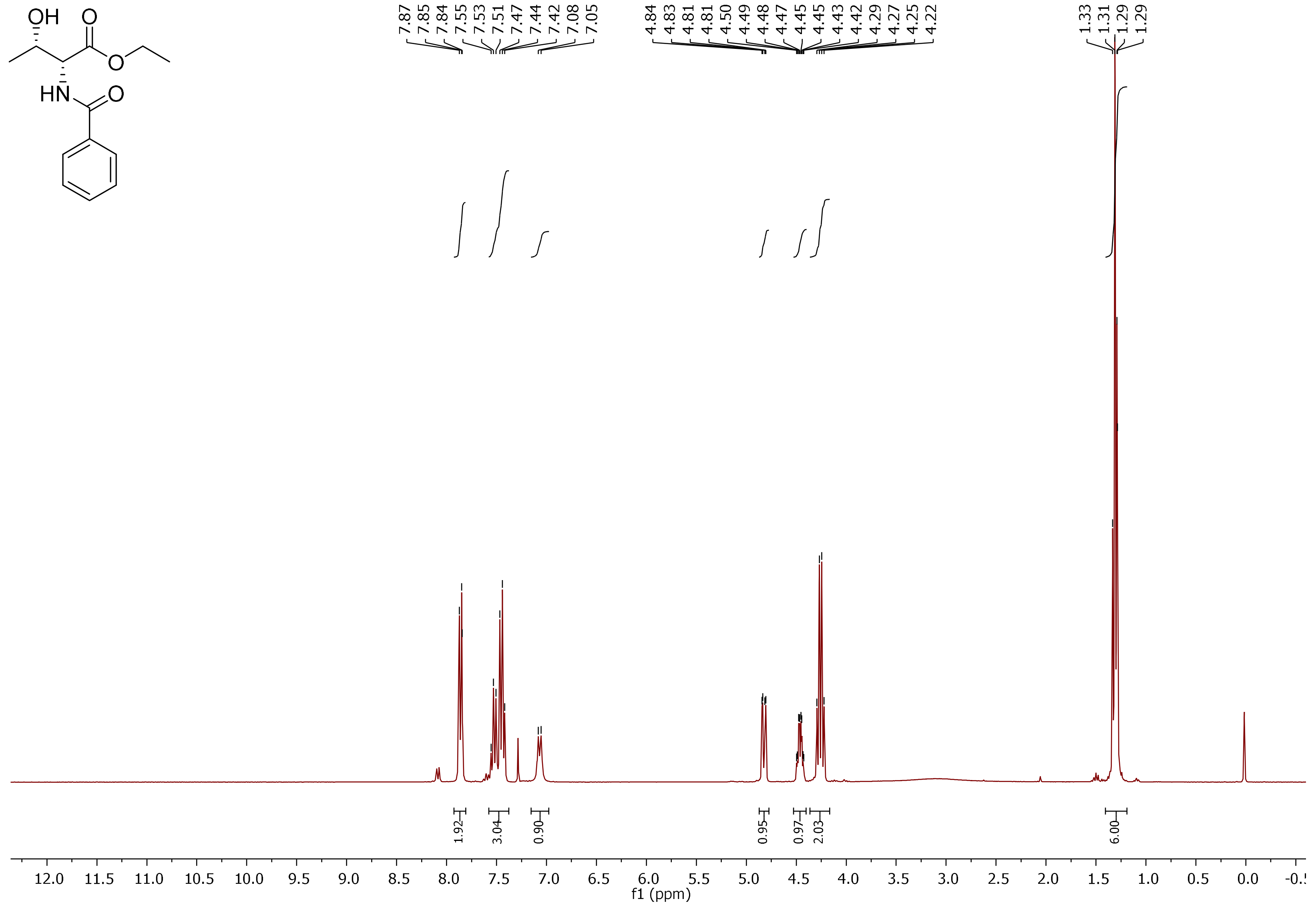

Figure S78: ^{1}H NMR spectrum of ethyl N-*benzoyl* D-*threoninate obtained from cascade biotransformation*

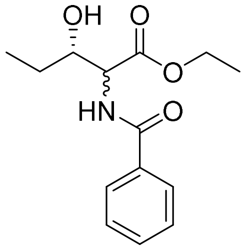

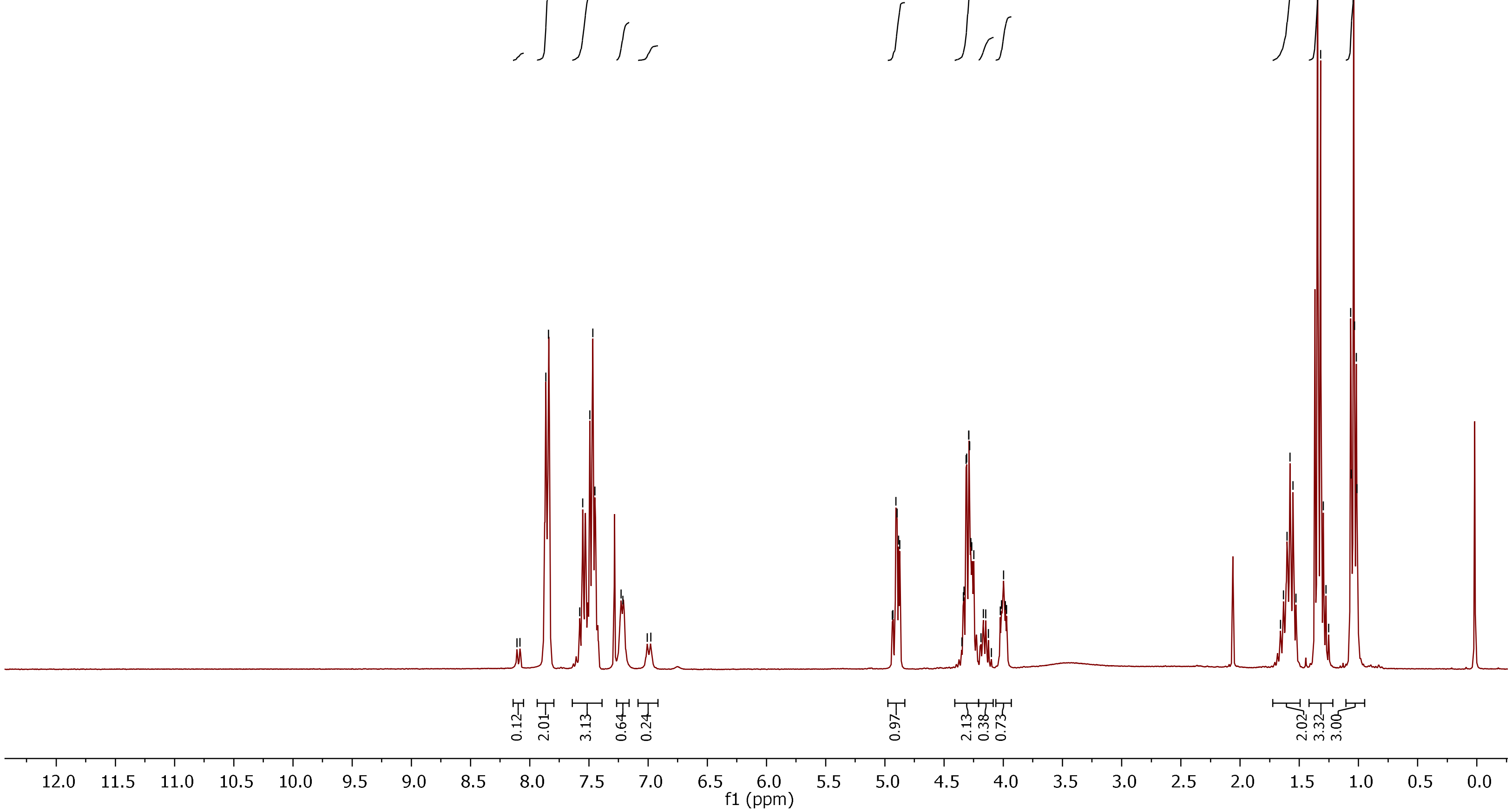

Figure S79: ¹H NMR spectrum of ethyl 2-benzamido-3-hydroxypentanoate obtained from cascade biotransformation

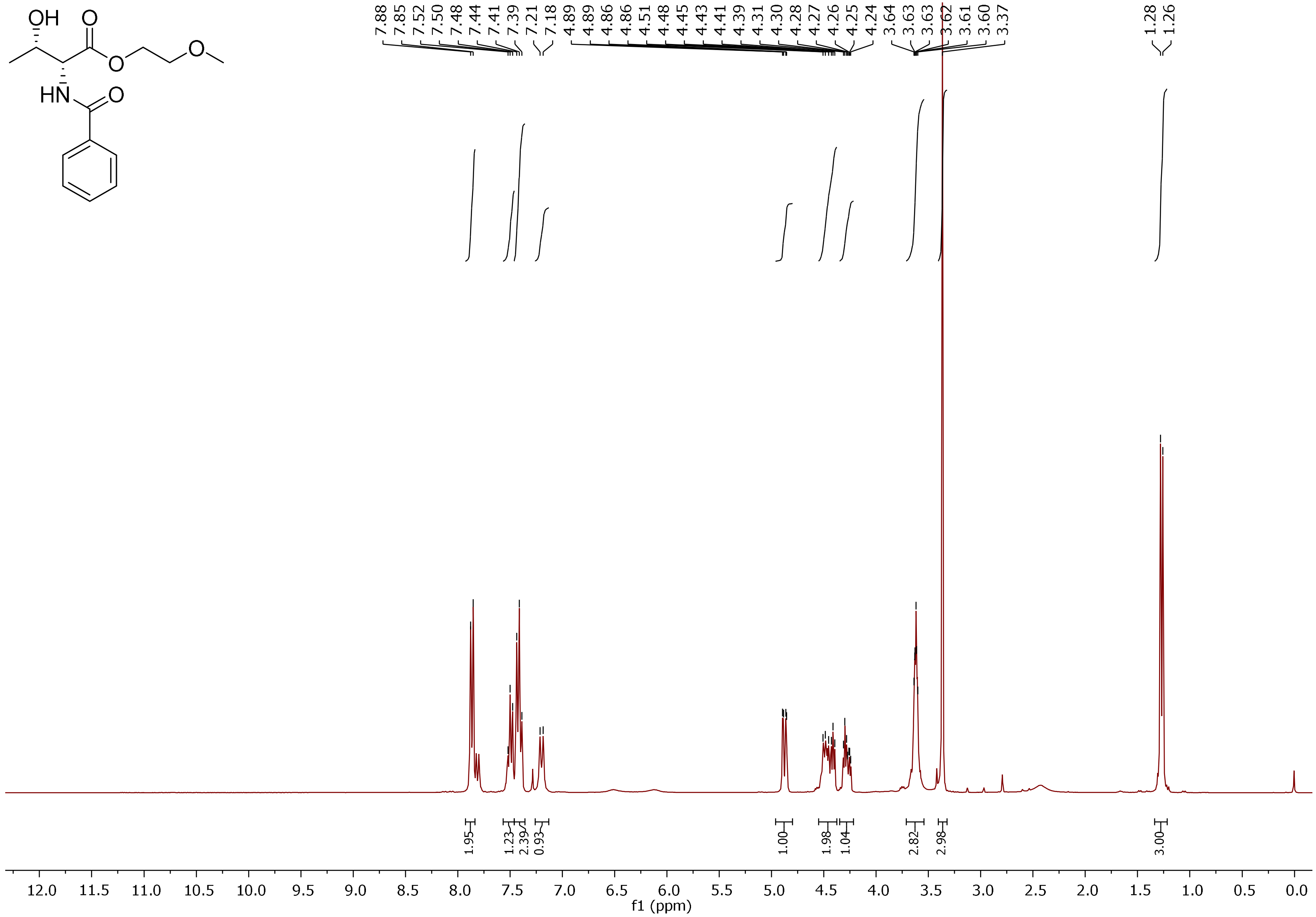

Figure S80: ^{1}H NMR spectrum of 2-methoxyethyl 2-benzamido-3-hydroxybutanoate obtained from cascade biotransformation

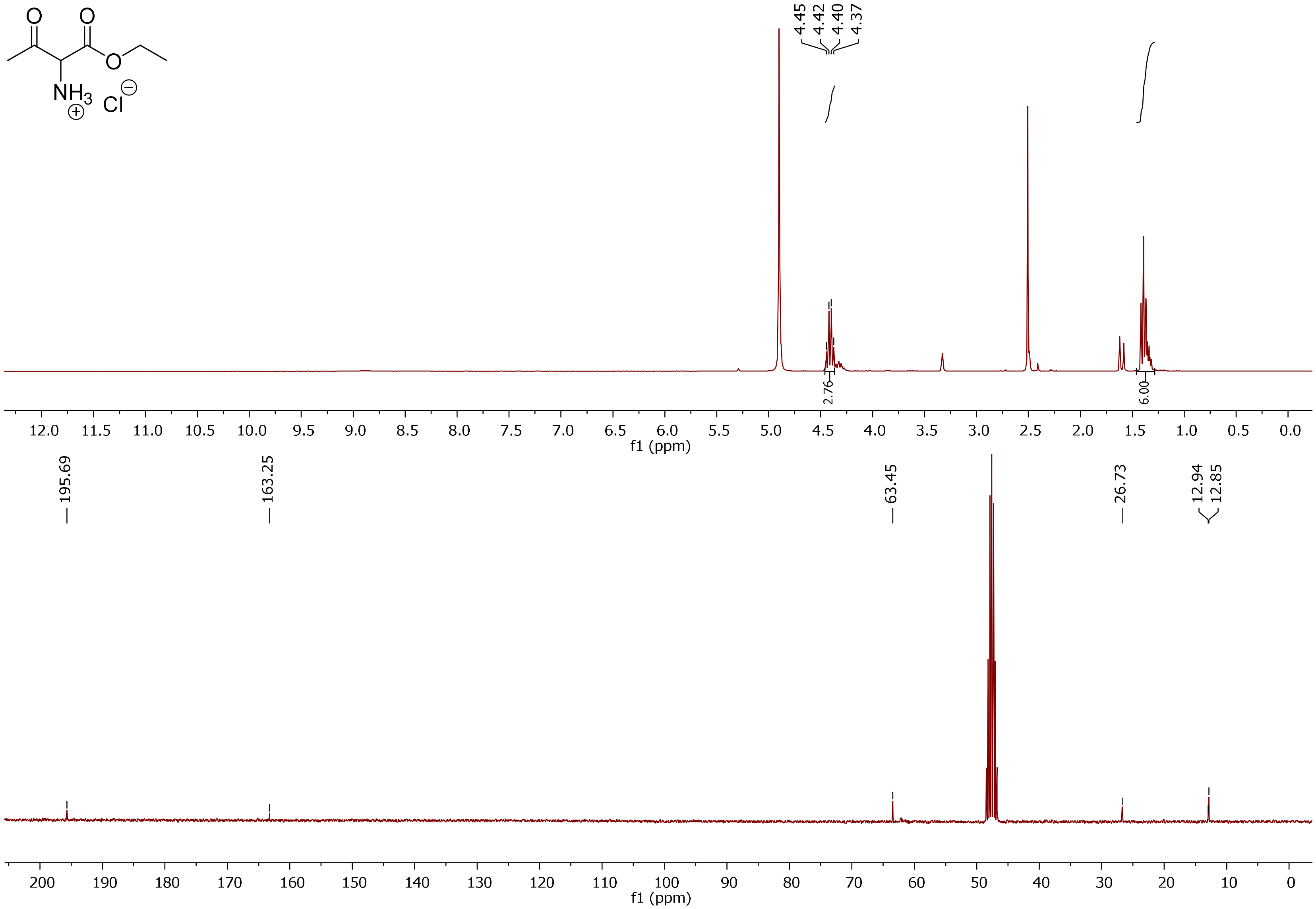

Figure S81: ^{1}H and ^{13}C NMR spectra of crude ethyl 2-amino-3-oxobutanoate hydrochloride ***2a·HCl***

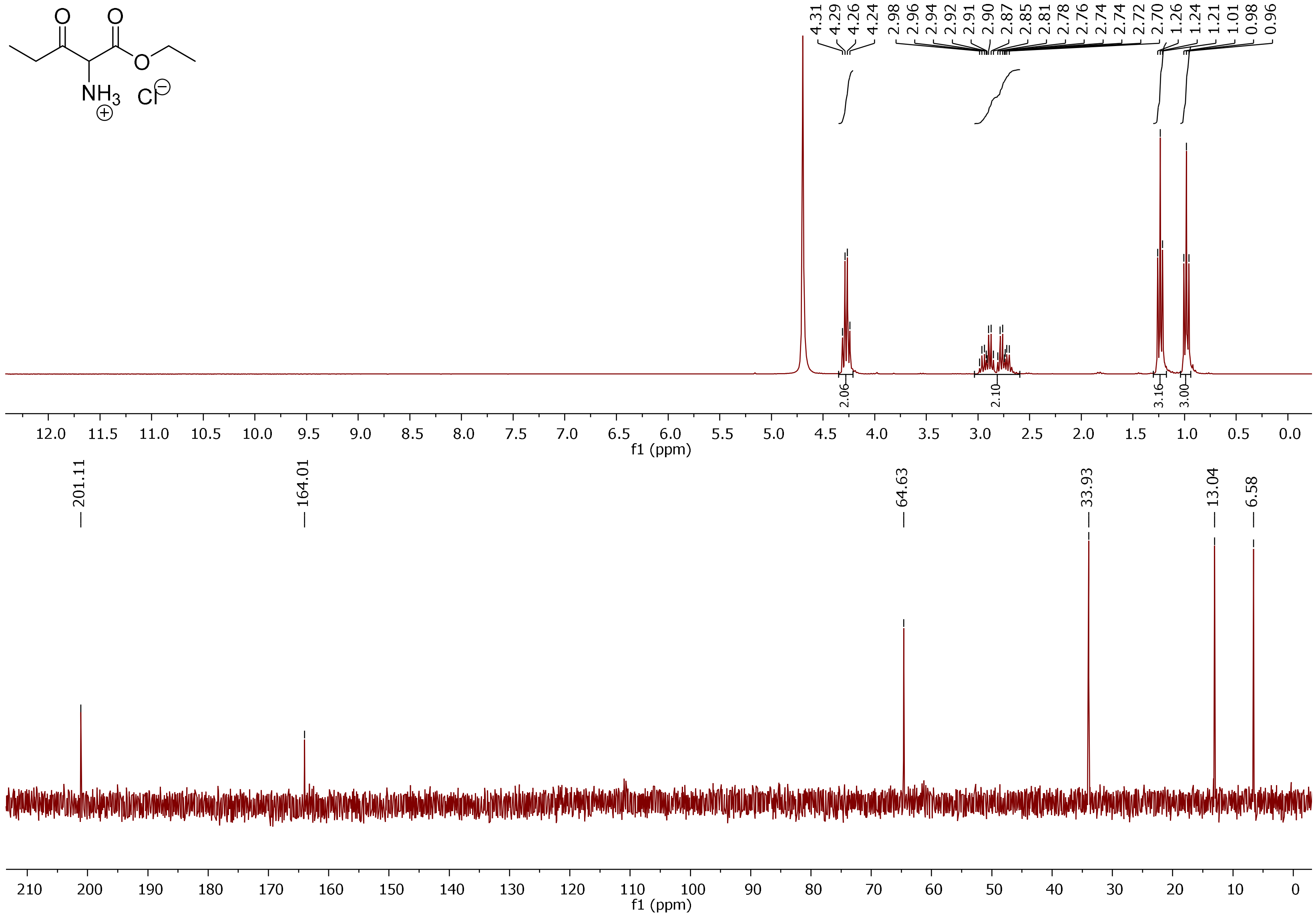

Figure S82: ¹H and ¹³C NMR spectra of ethyl 2-amino-3-oxopentanoate hydrochloride ***2b·HCl***

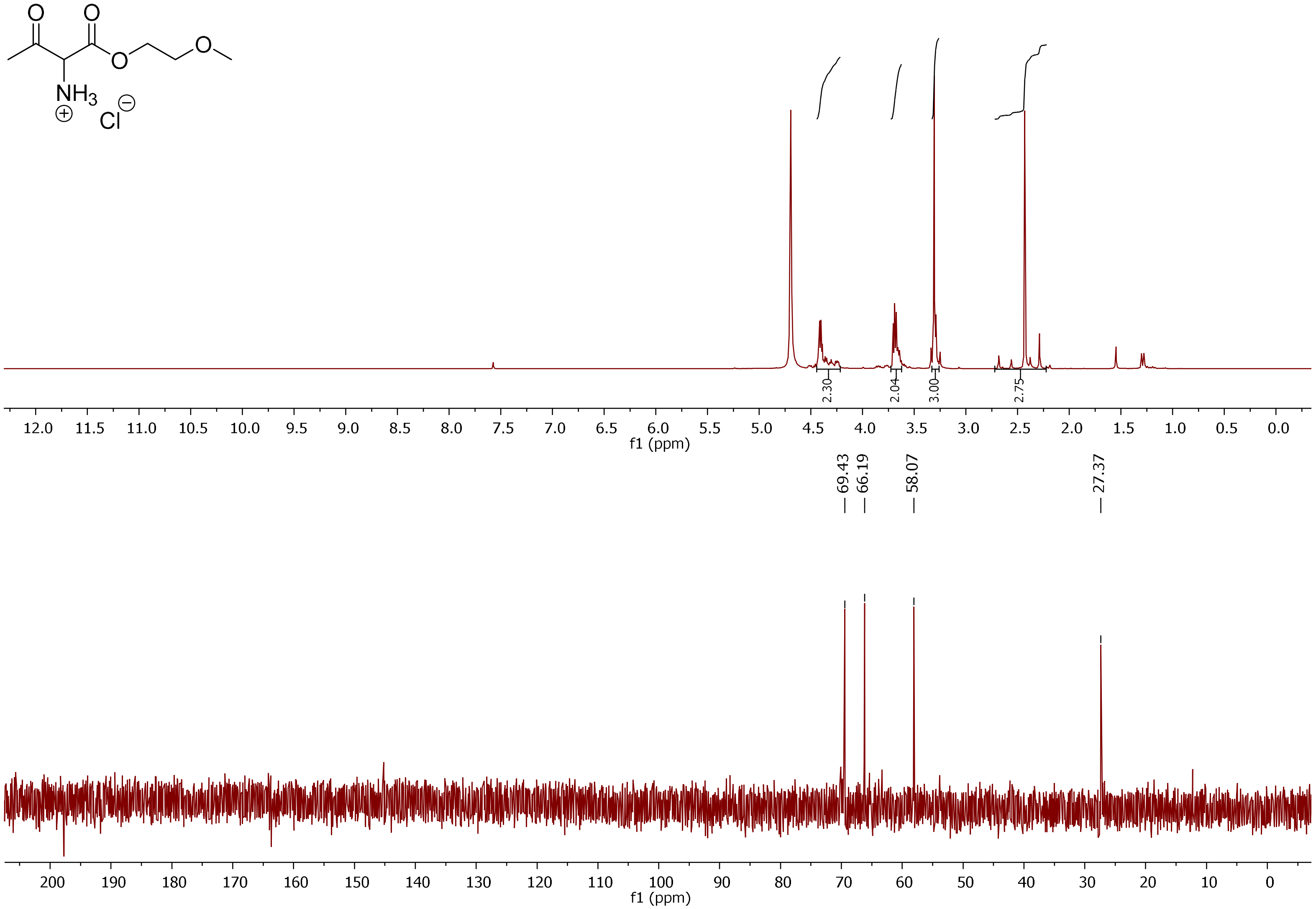

Figure S83: ^{1}H and ^{13}C NMR spectra of 2-methoxyethyl 2-amino-3-oxobutanoate ***1c·HCl***

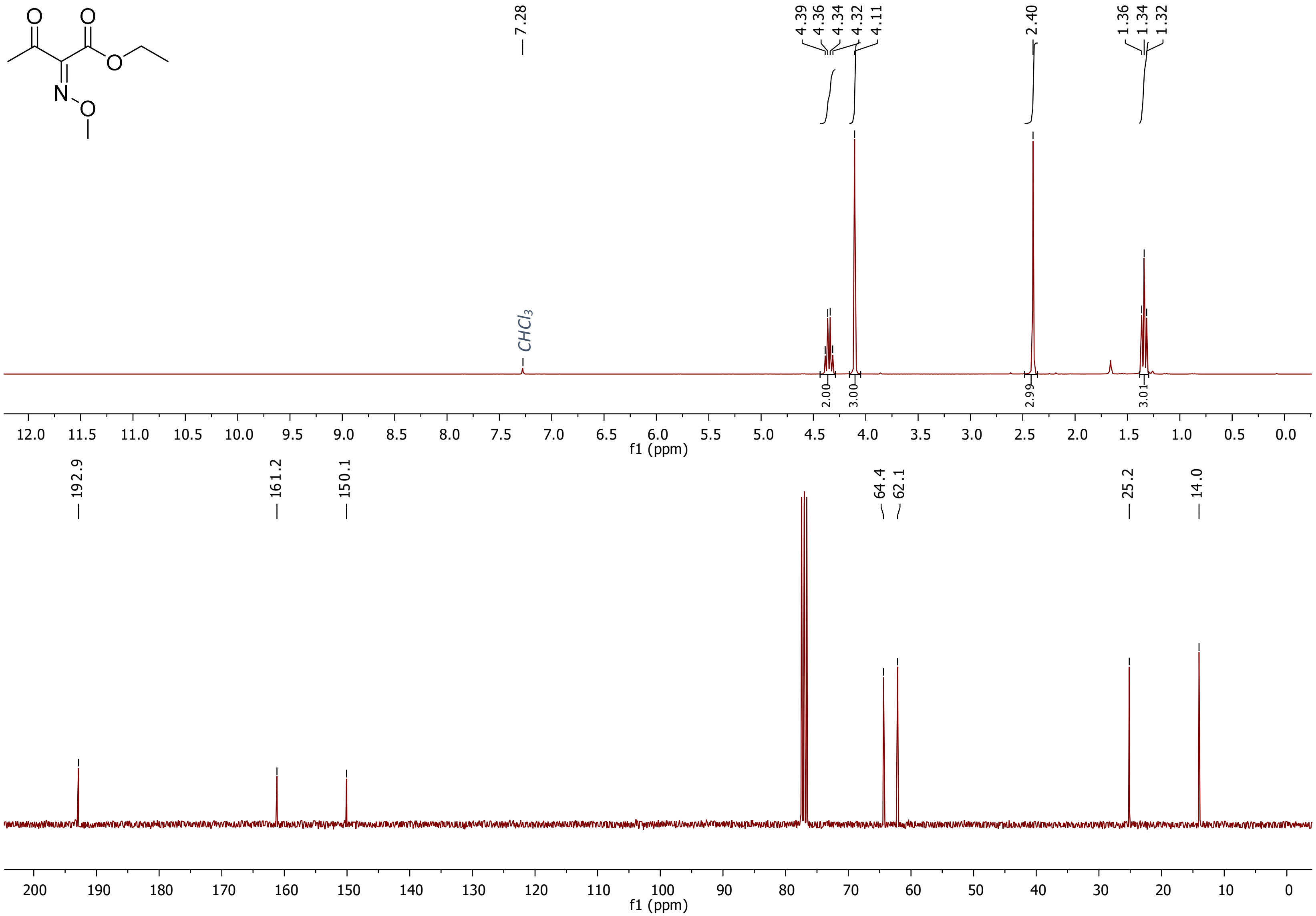

Figure S84. *^{1}H- and ^{13}C-NMR spectra of ethyl Z-2-(methoxyimino)-3-oxobutanoate Z-**7a***

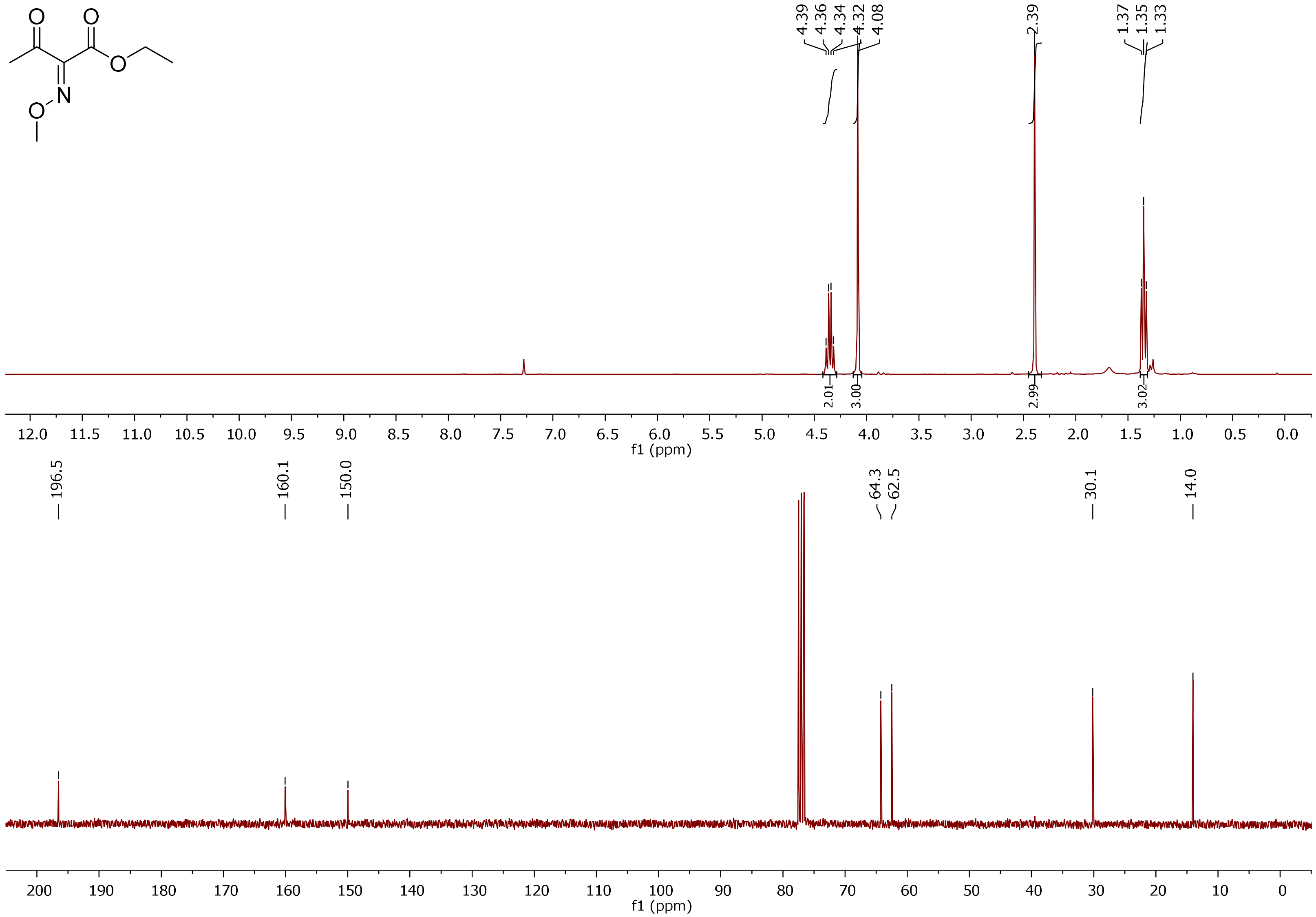

Figure S85. ^{1}H- and ^{13}C-NMR spectra of ethyl *E*-2-(methoxyimino)-3-oxobutanoate ***E*-7a**

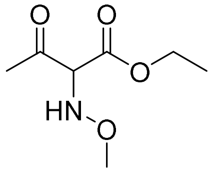

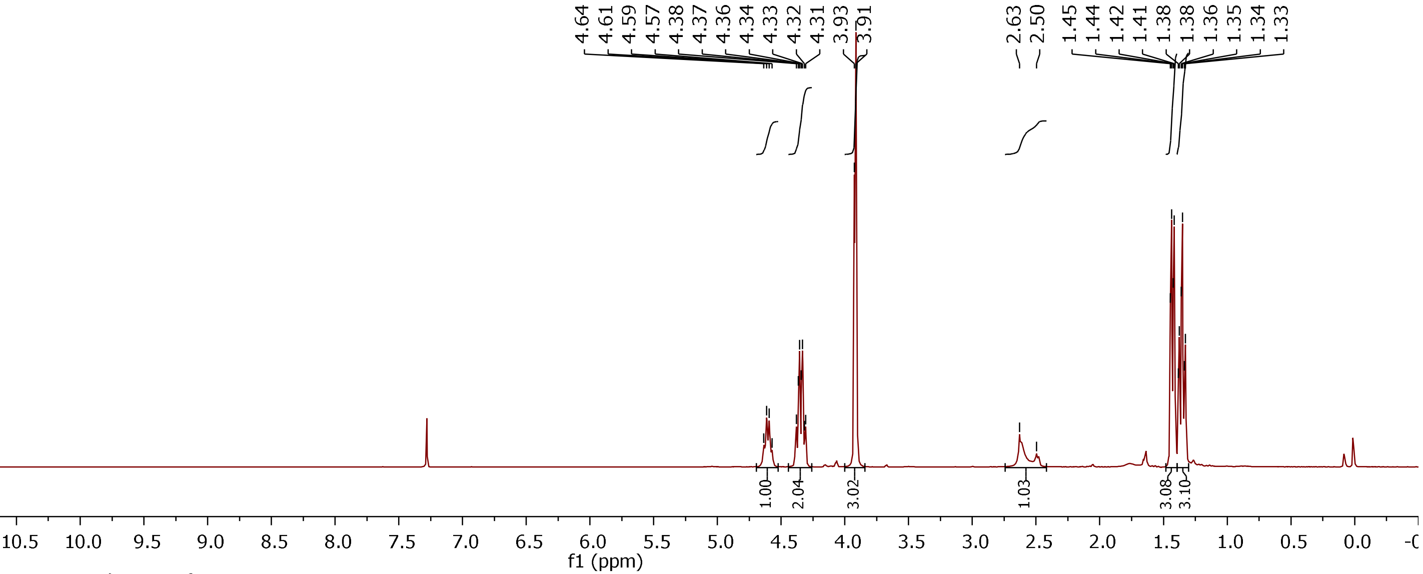

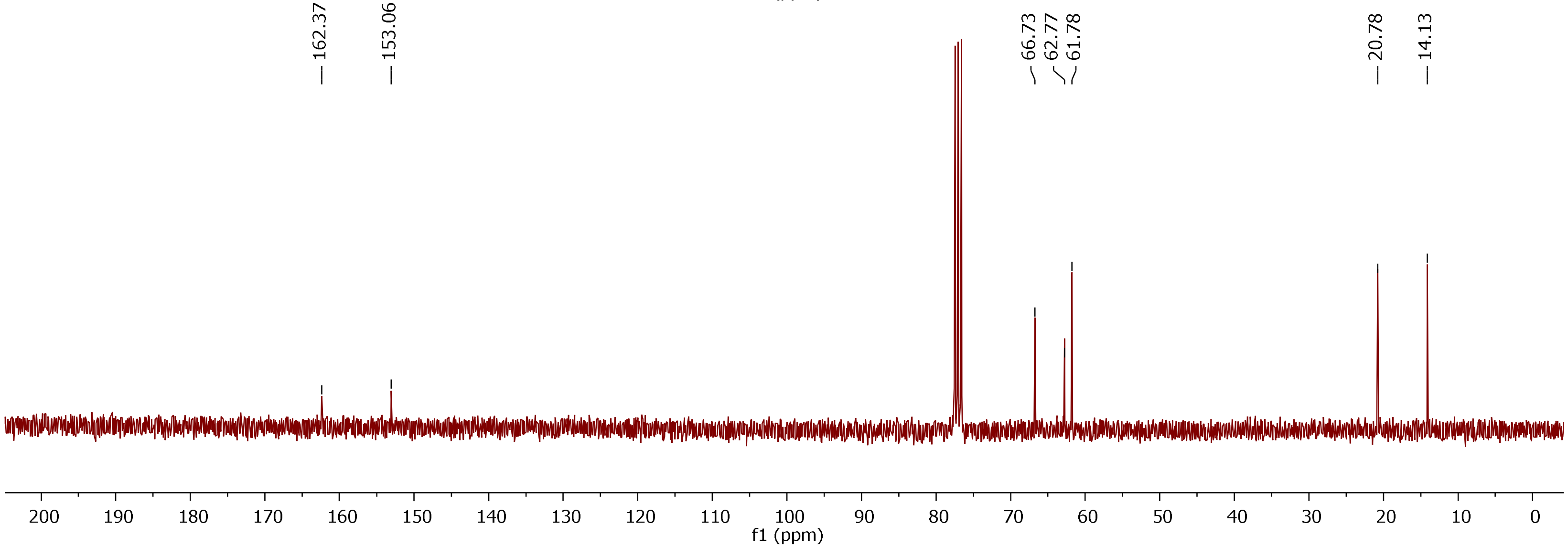

Figure S86: ^1H and ^{13}C NMR spectra of ethyl 2-(methoxyamino)-3-oxobutanoate ***8a***

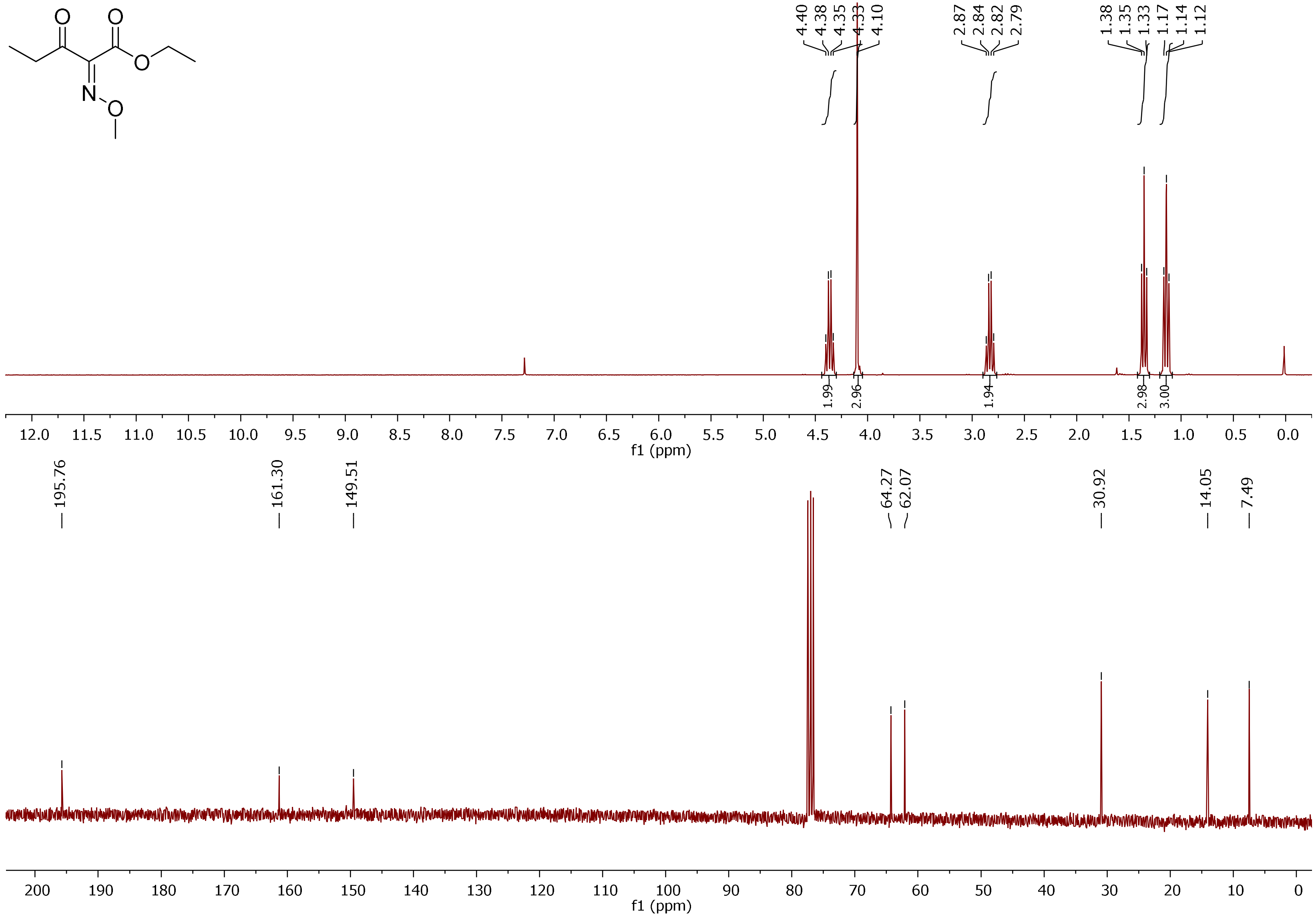

*Figure S87: ^{1}H and ^{13}C NMR spectra of ethyl Z-2-(methoxyimino)-3-oxopentanoate Z-***7b**

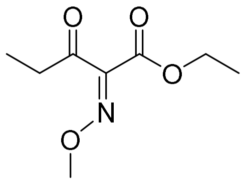

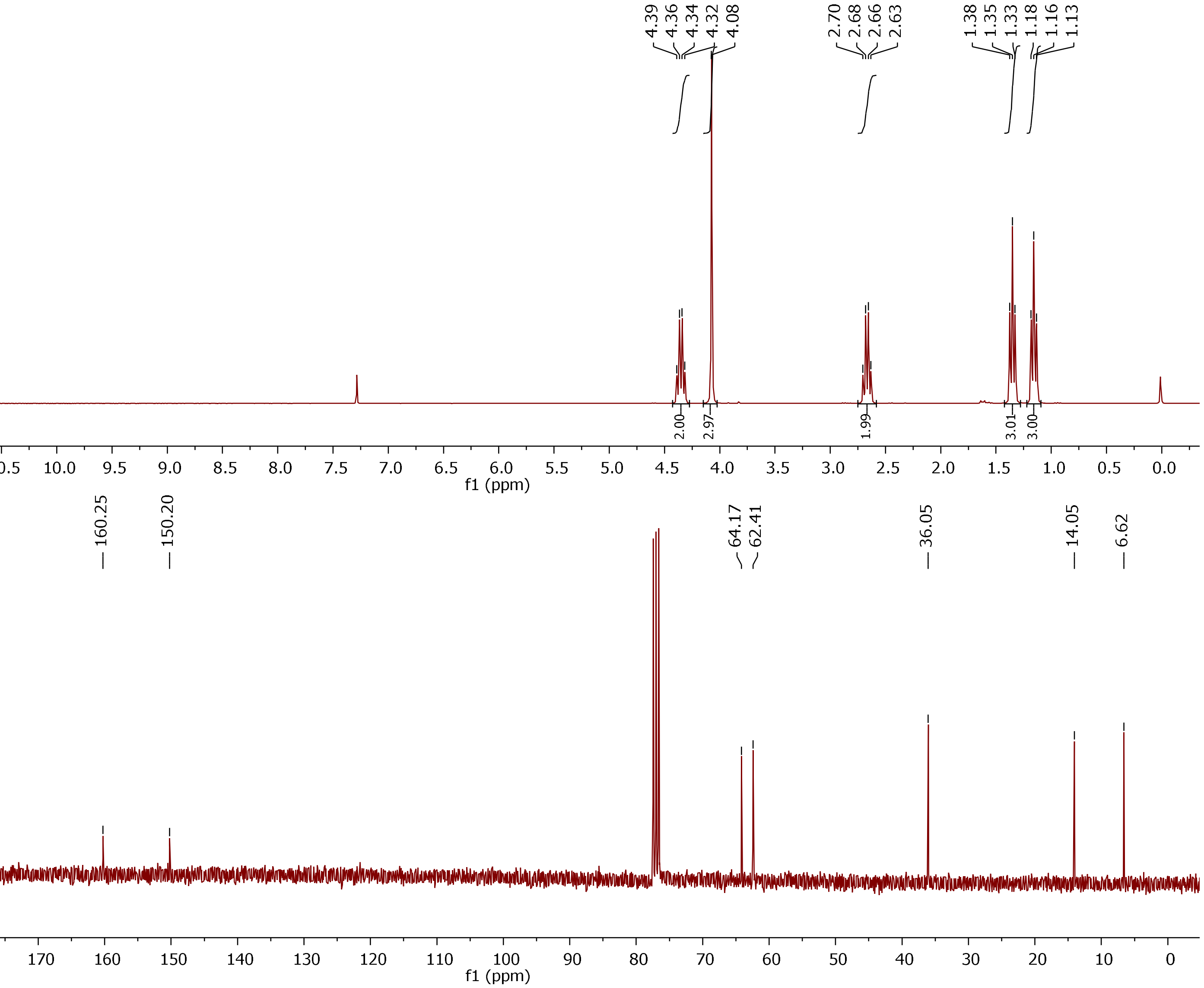

*Figure S88: ^{1}H and ^{13}C NMR spectra of ethyl E-2-(methoxyimino)-3-oxopentanoate E-**7b***

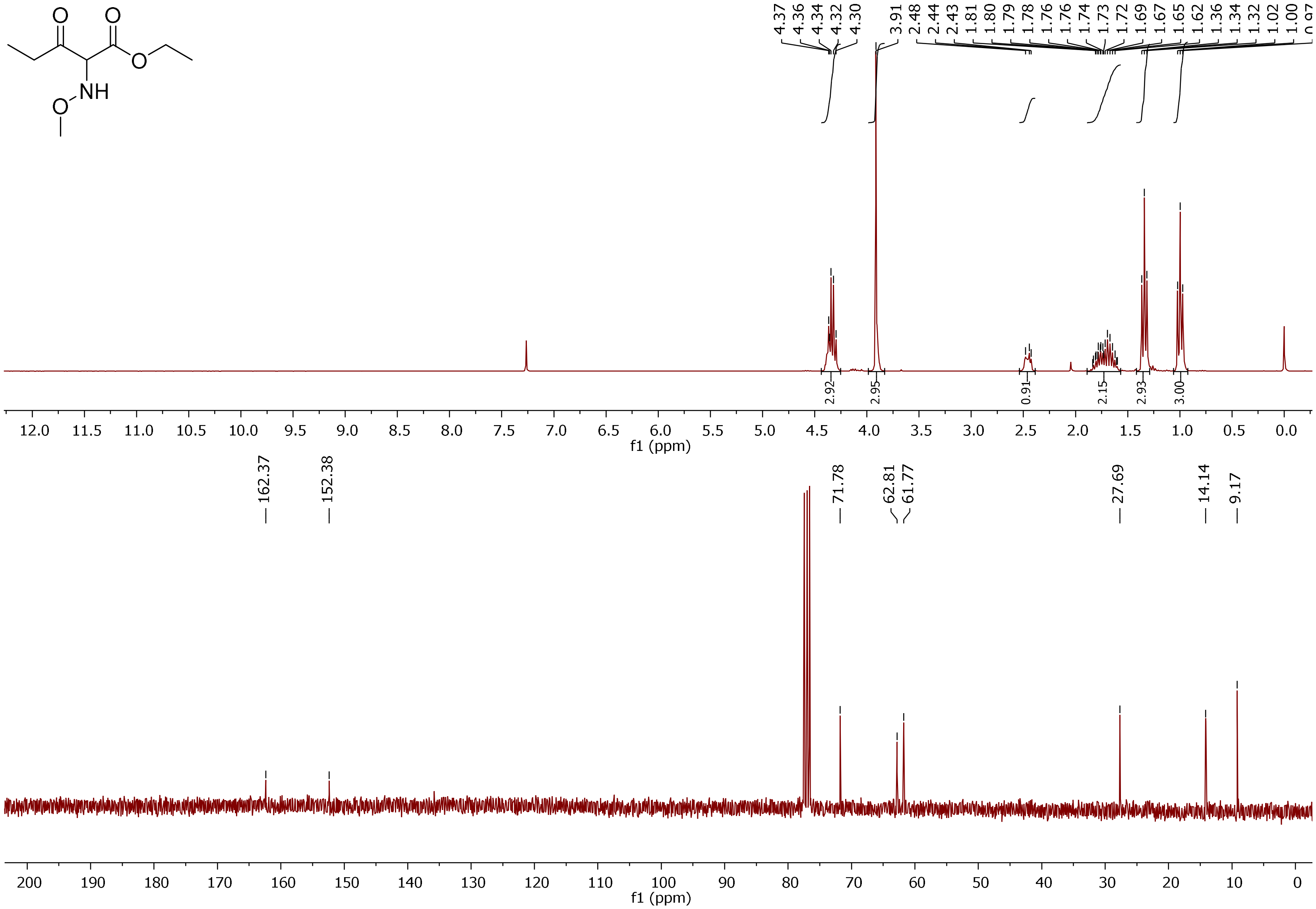

Figure S89: ^{1}H and ^{13}C NMR spectra of ethyl 2-(methoxyamino)-3-oxopentanoate ***8b***

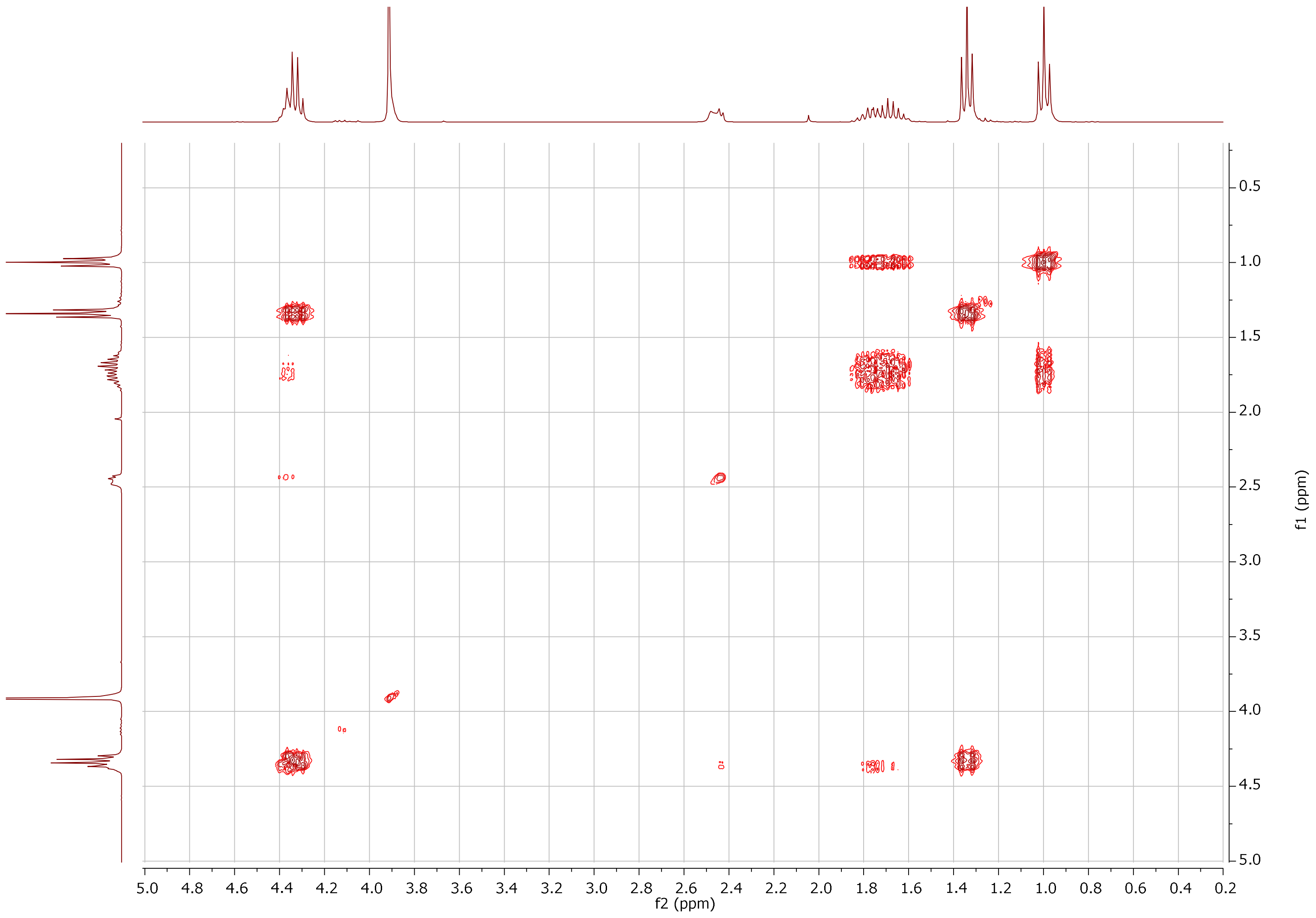

*Figure S90: ^{1}H-^{1}H COSY spectrum of ethyl 2-(methoxyamino)-3-oxopentanoate **8b***

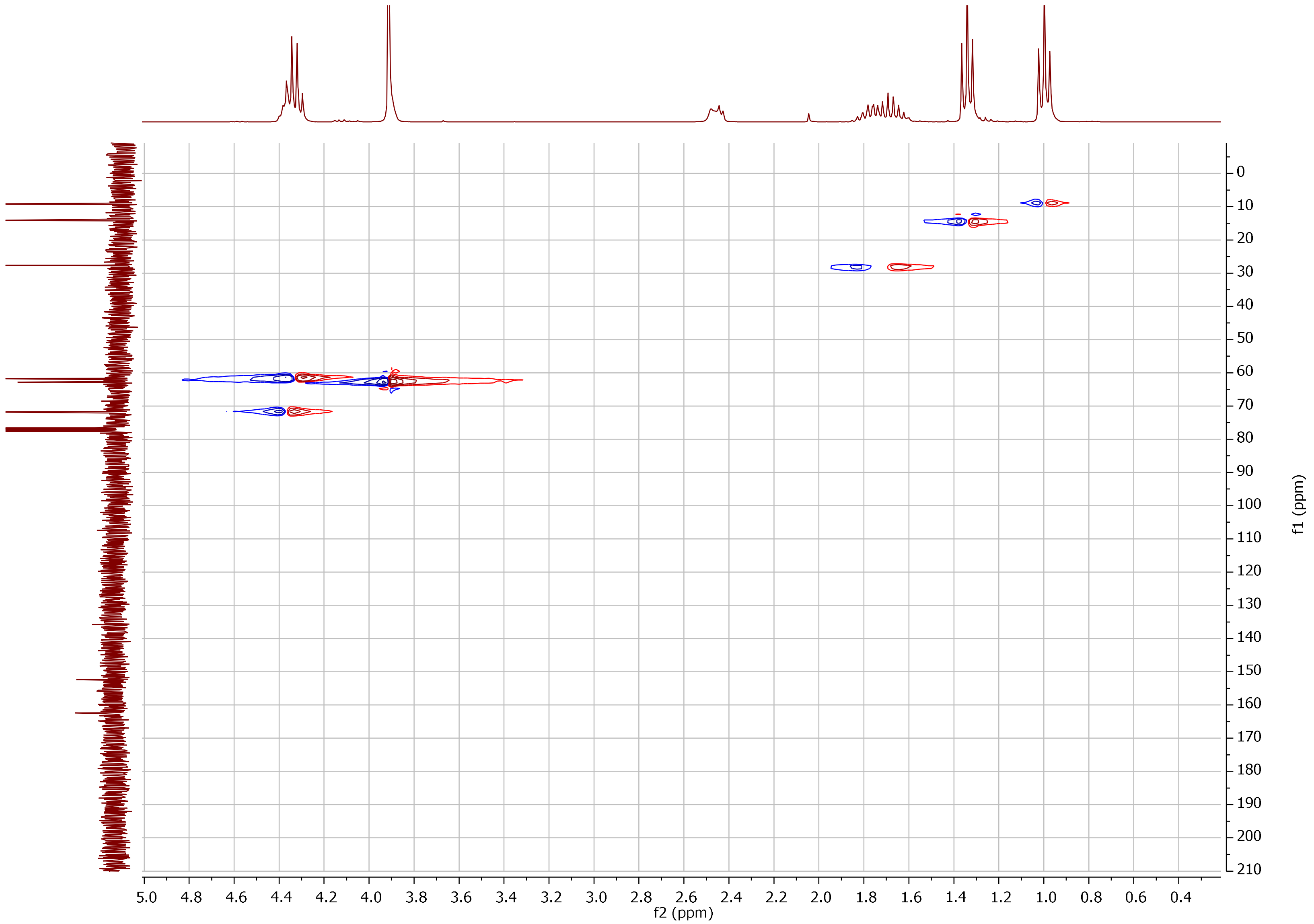

Figure S91: ^{1}H-^{13}C HSQC spectrum of ethyl 2-(methoxyamino)-3-oxopentanoate ***8b***

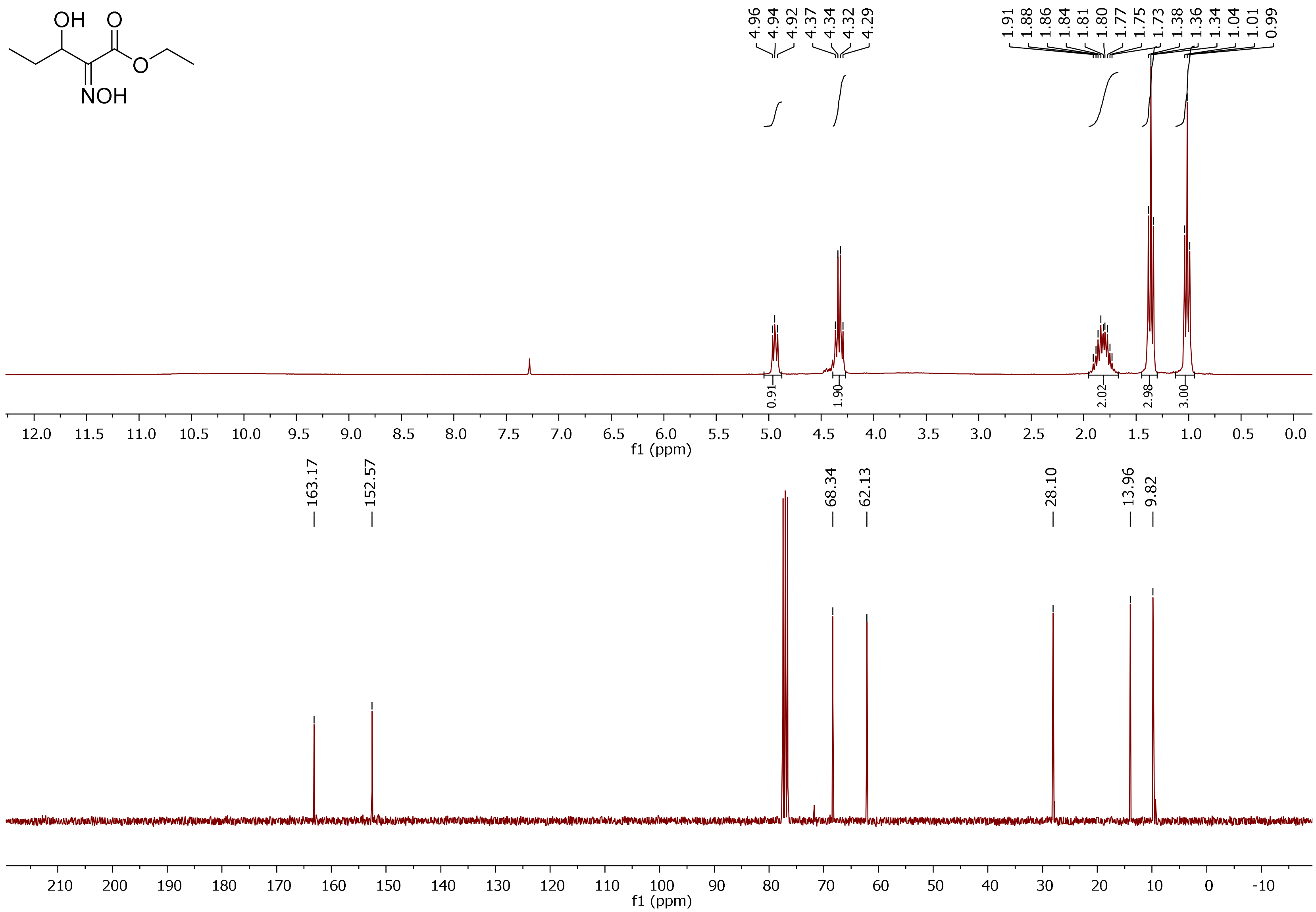

Figure S92: ^{1}H and ^{13}C NMR spectra of ethyl 3-hydroxy-2-(hydroxyimino)pentanoate ***S2***

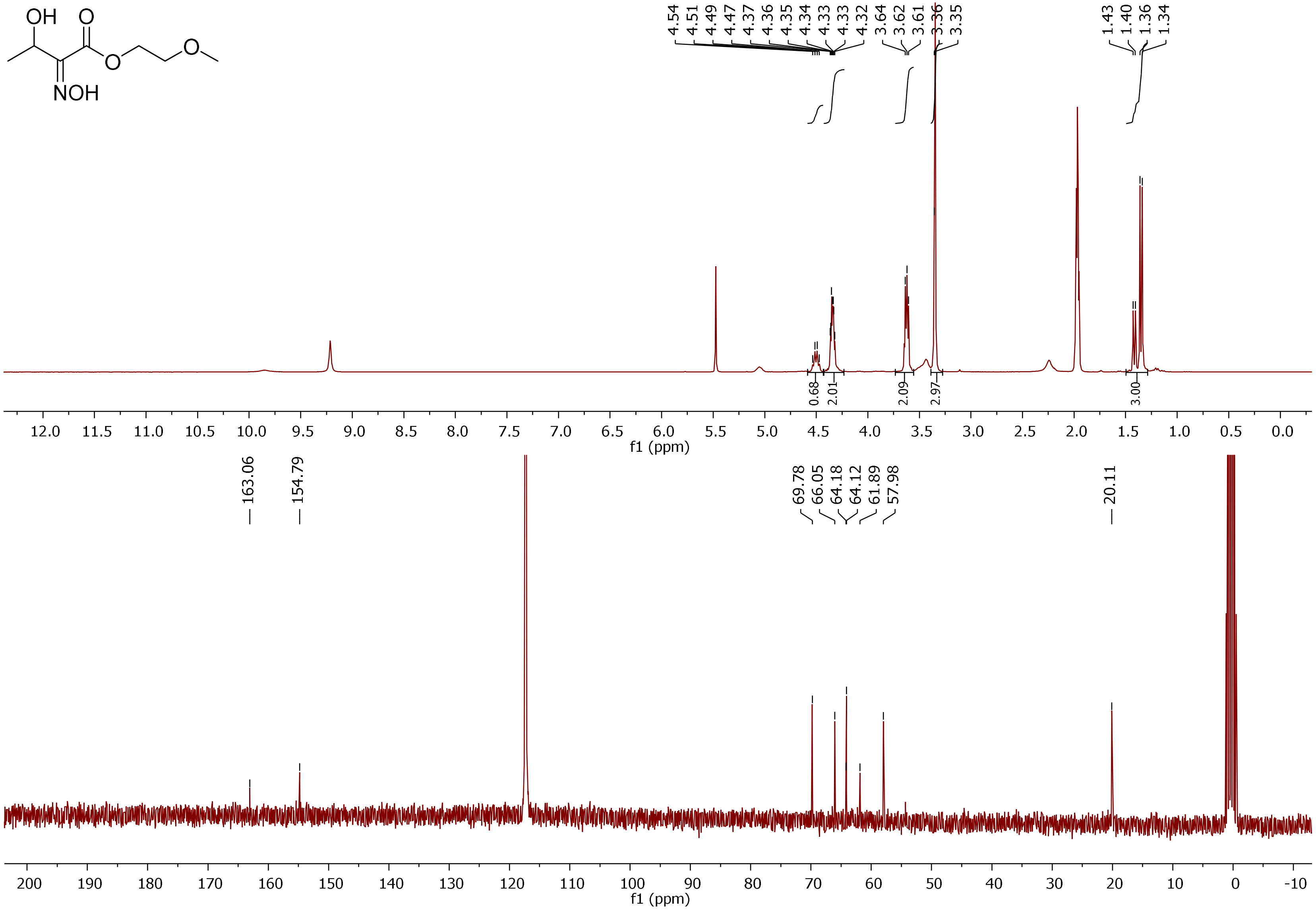

*Figure S93: ^{1}H and ^{13}C NMR spectra of 2-methoxyethyl (Z)-3-hydroxy-2-(hydroxyimino)butanoate **S1***

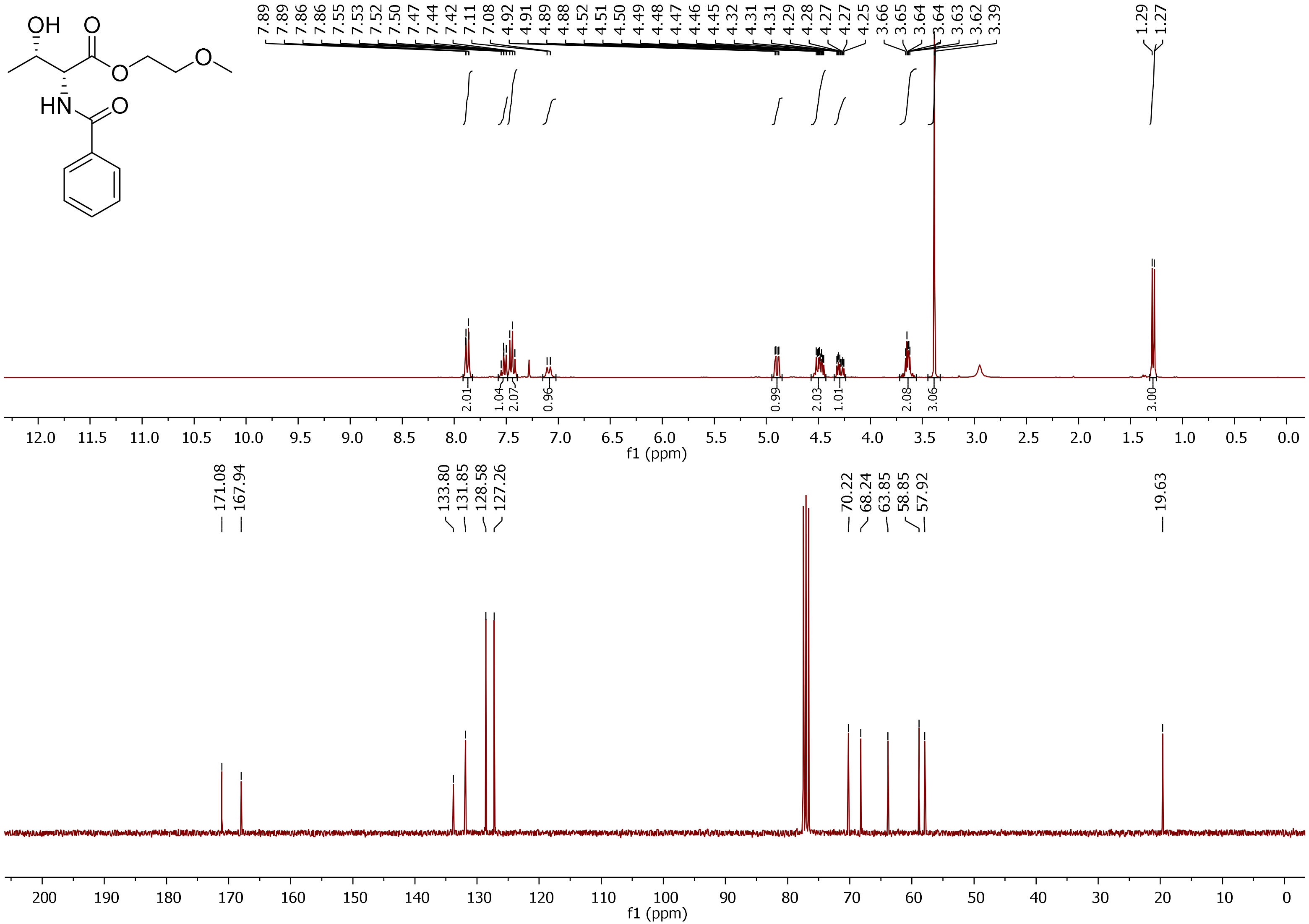

*Figure S94: ^{1}H and ^{13}C NMR spectra of 2-methoxyethyl benzoyl-D-threoninate (**N-Bz-4c**)*

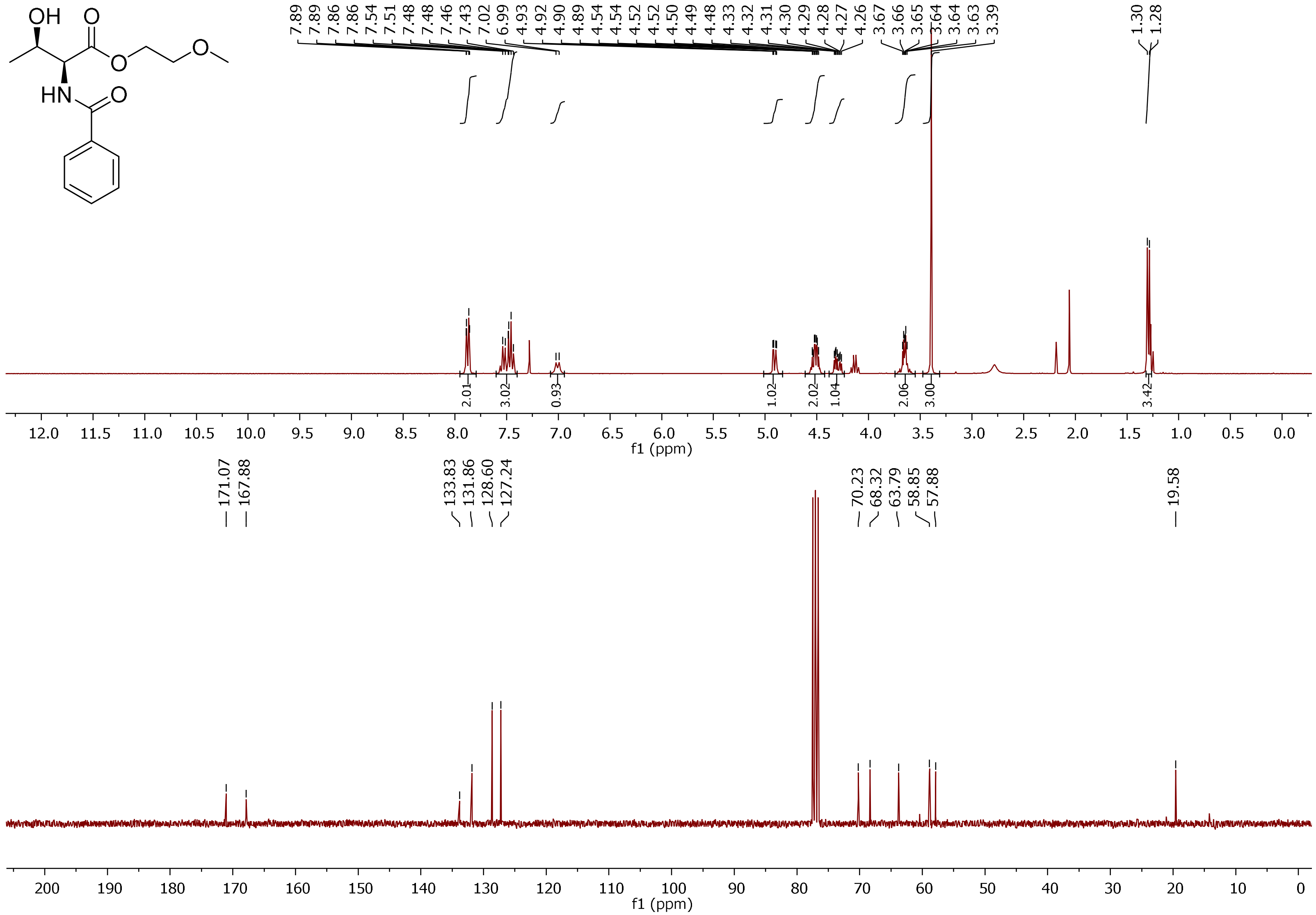

*Figure S95: ¹H and ¹³C NMR spectra of 2-methoxyethyl benzoyl-L-threoninate (**N-Bz-4c**)*

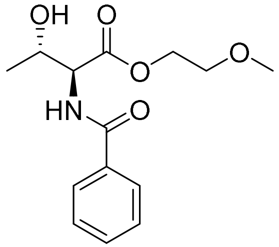

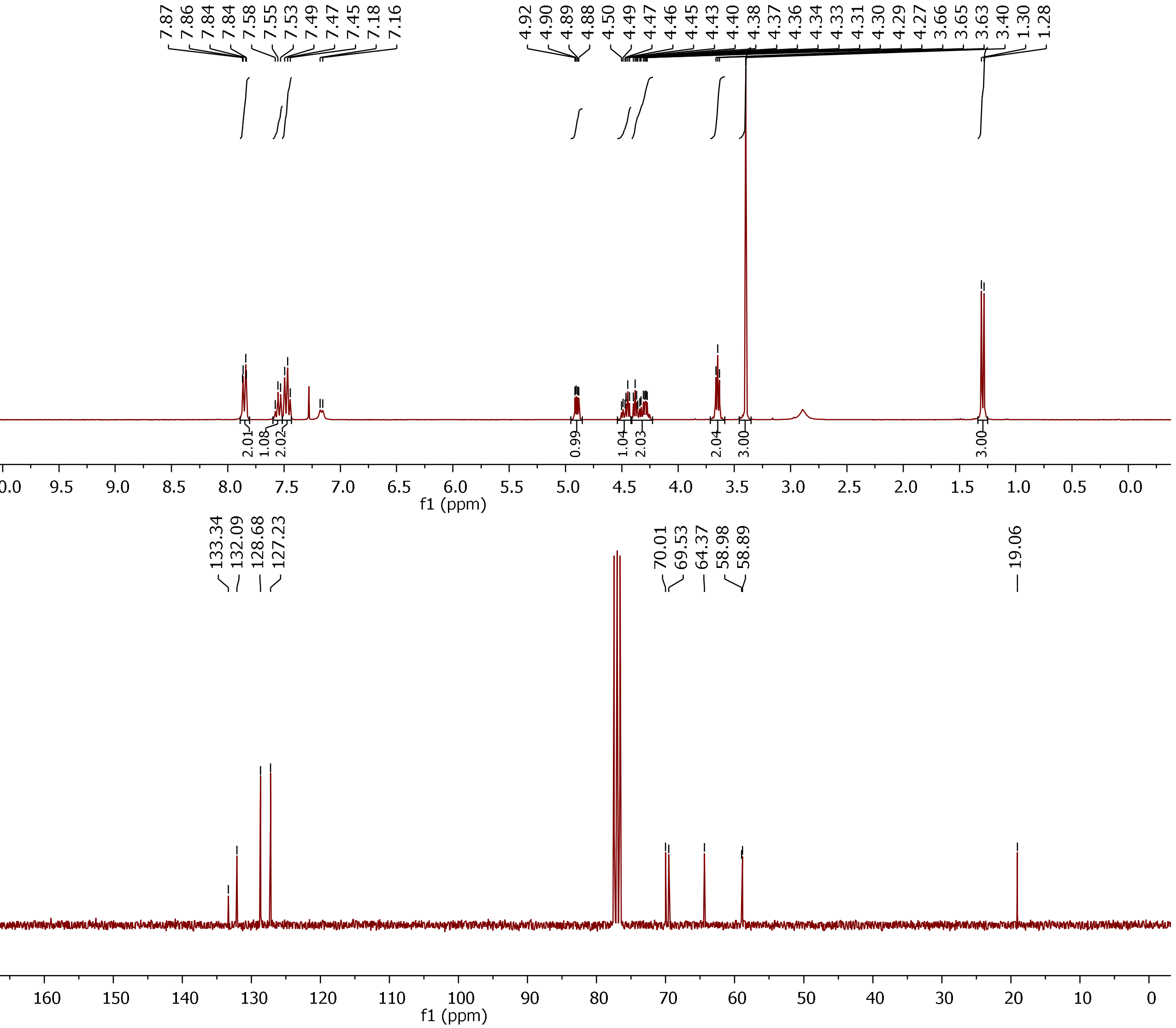

*Figure S96: 1H and 13C NMR spectra of 2-methoxyethyl benzoyl-L-allo-threoninate (**N-Bz-4c**)*

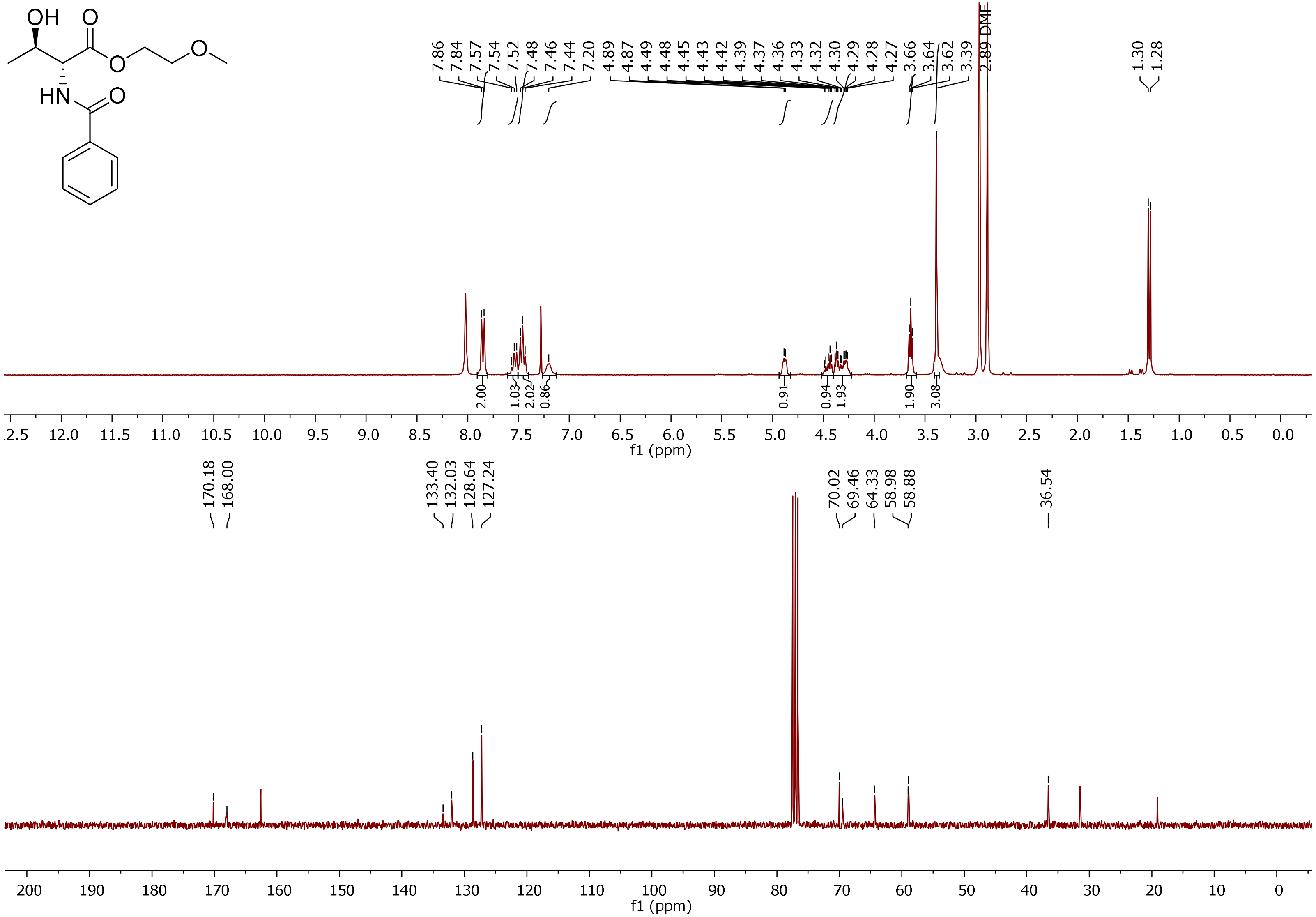

*Figure S97: 1H and 13C NMR spectra of 2-methoxyethyl benzoyl-D-allo-threoninate (containing residual DMF) (**N-Bz-4c**)*

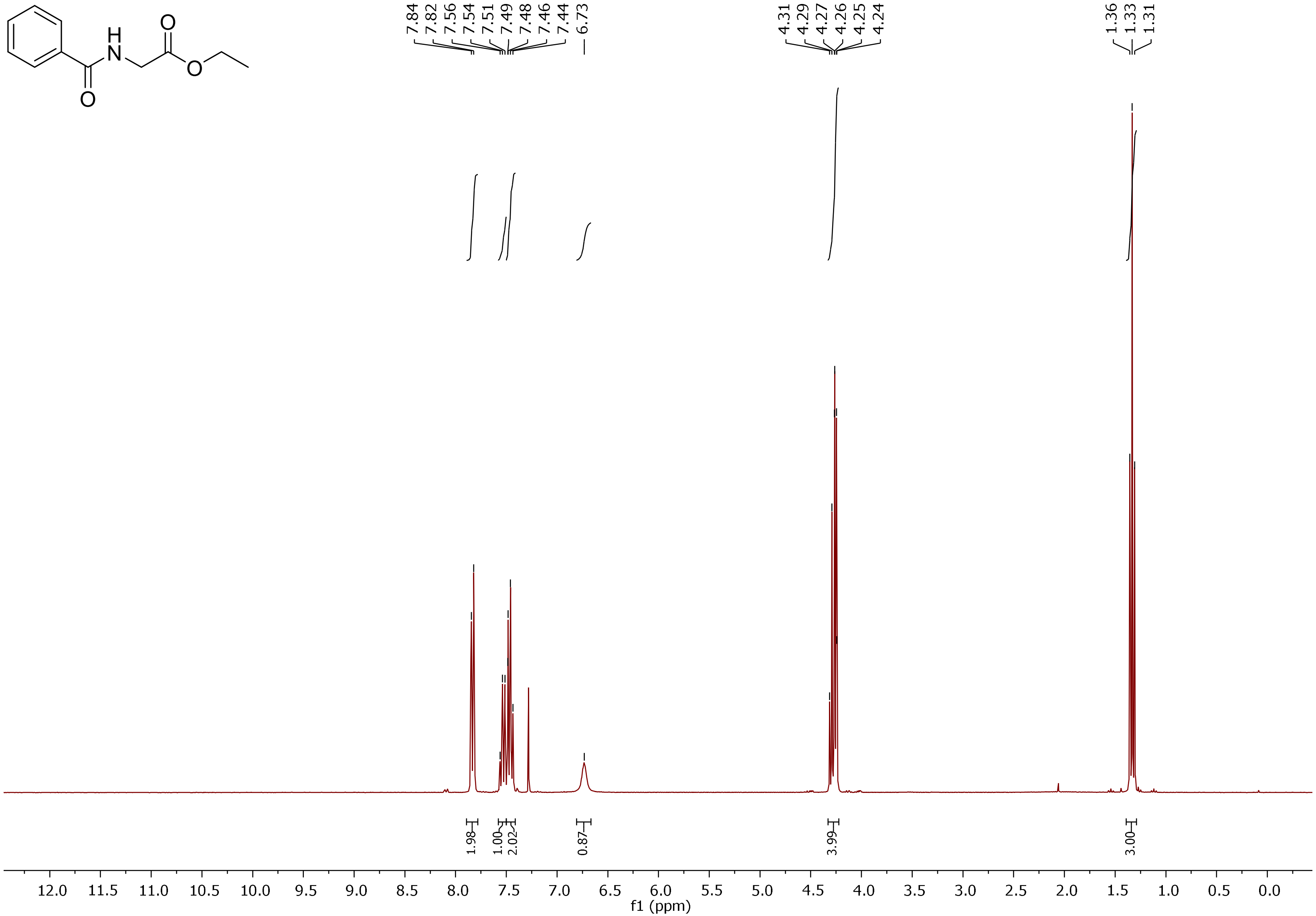

Figure S98: ¹H NMR spectrum of N-*benzoyl glycine ethyl ester* ***S3***

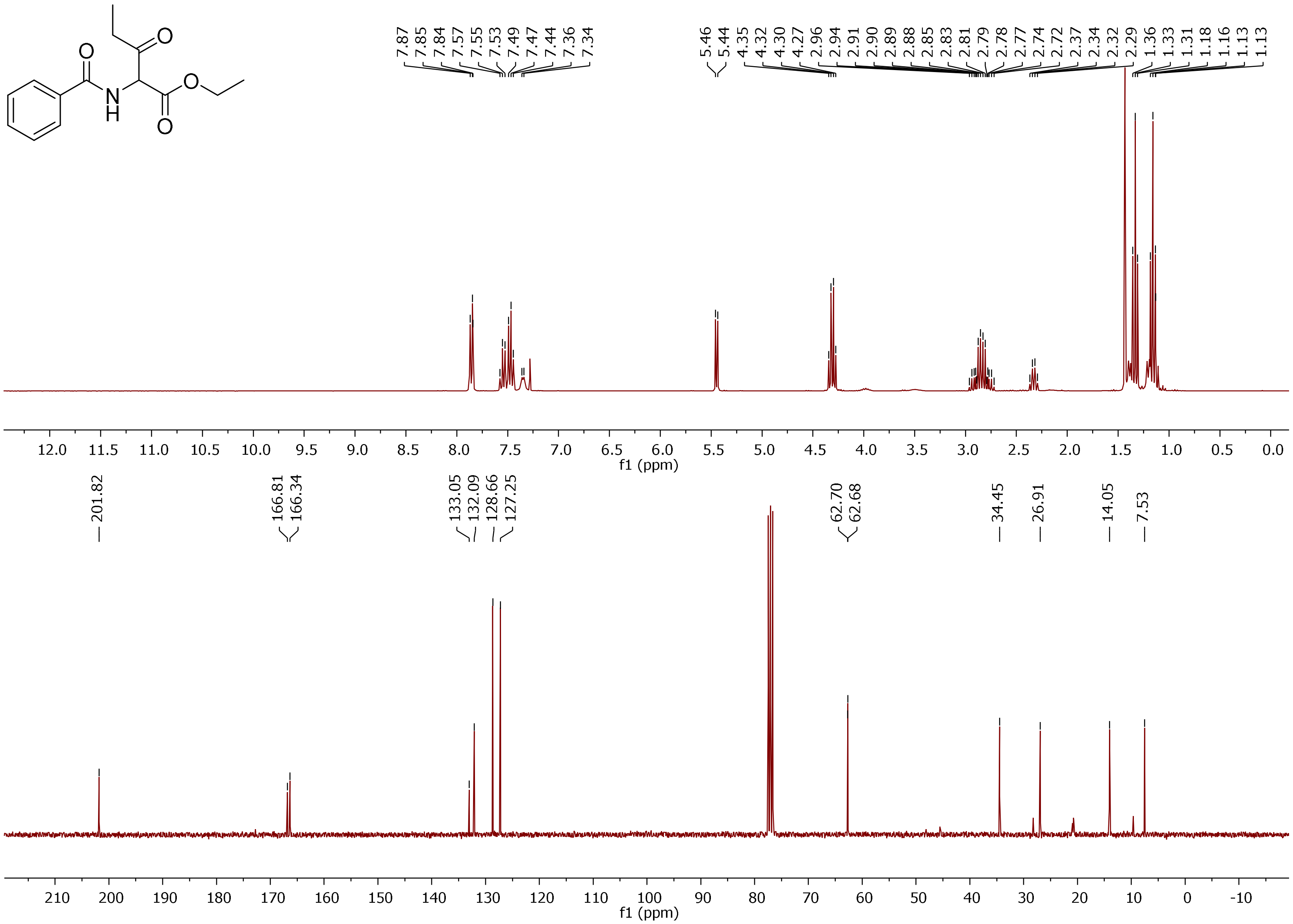

*Figure S99: ^{1}H and ^{13}C NMR spectra of ethyl 2-benzamido-3-oxopentanoate **S4***

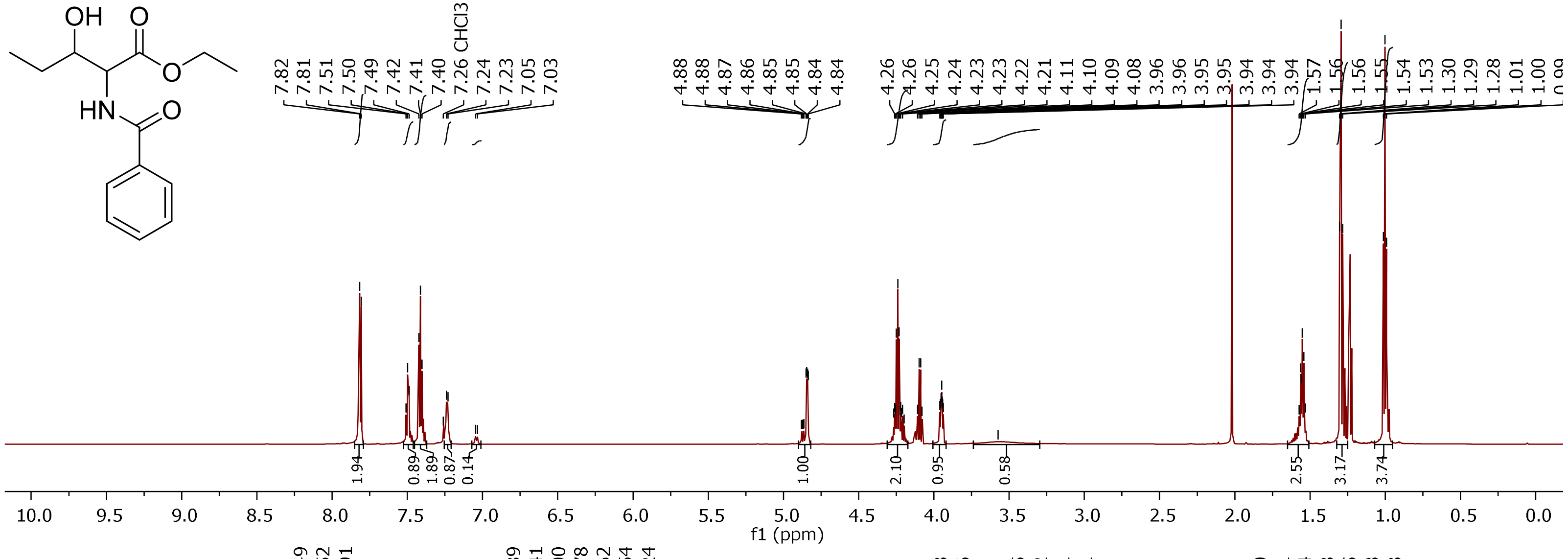

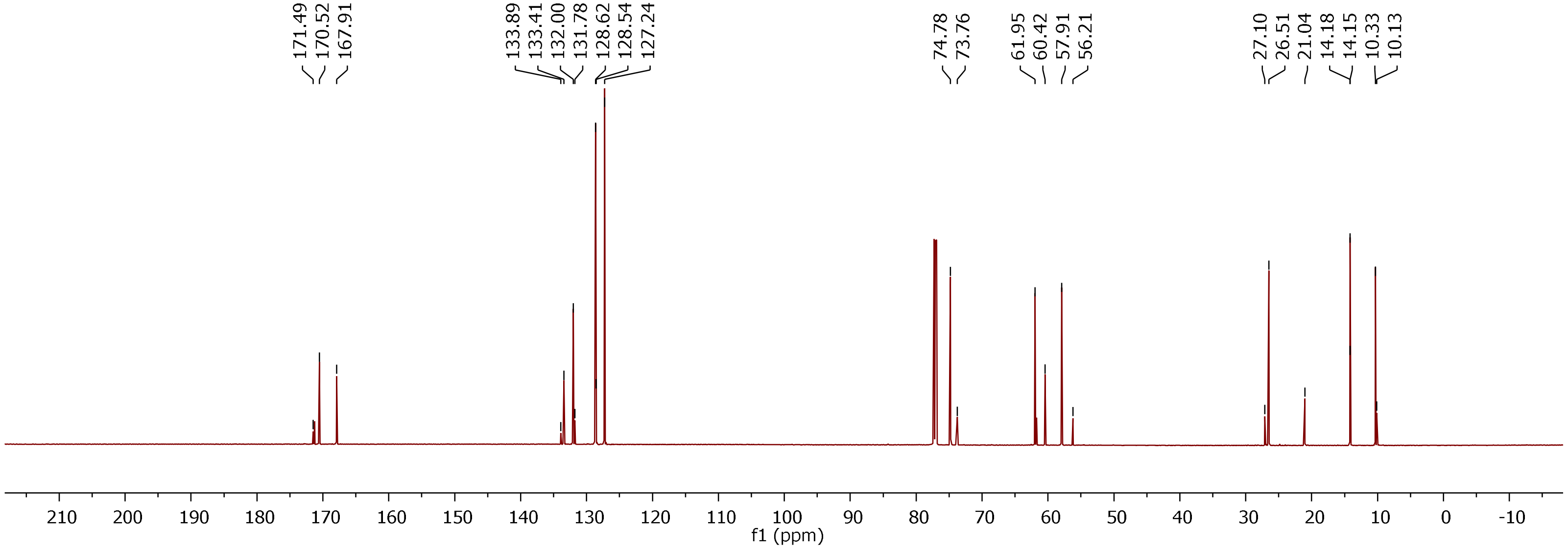

*Figure S100: ^{1}H and ^{13}C NMR spectra of racemic ethyl 2-benzamido-3-hydroxypentanoate (with residual ethyl acetate) (N-Bz-**4b**)*

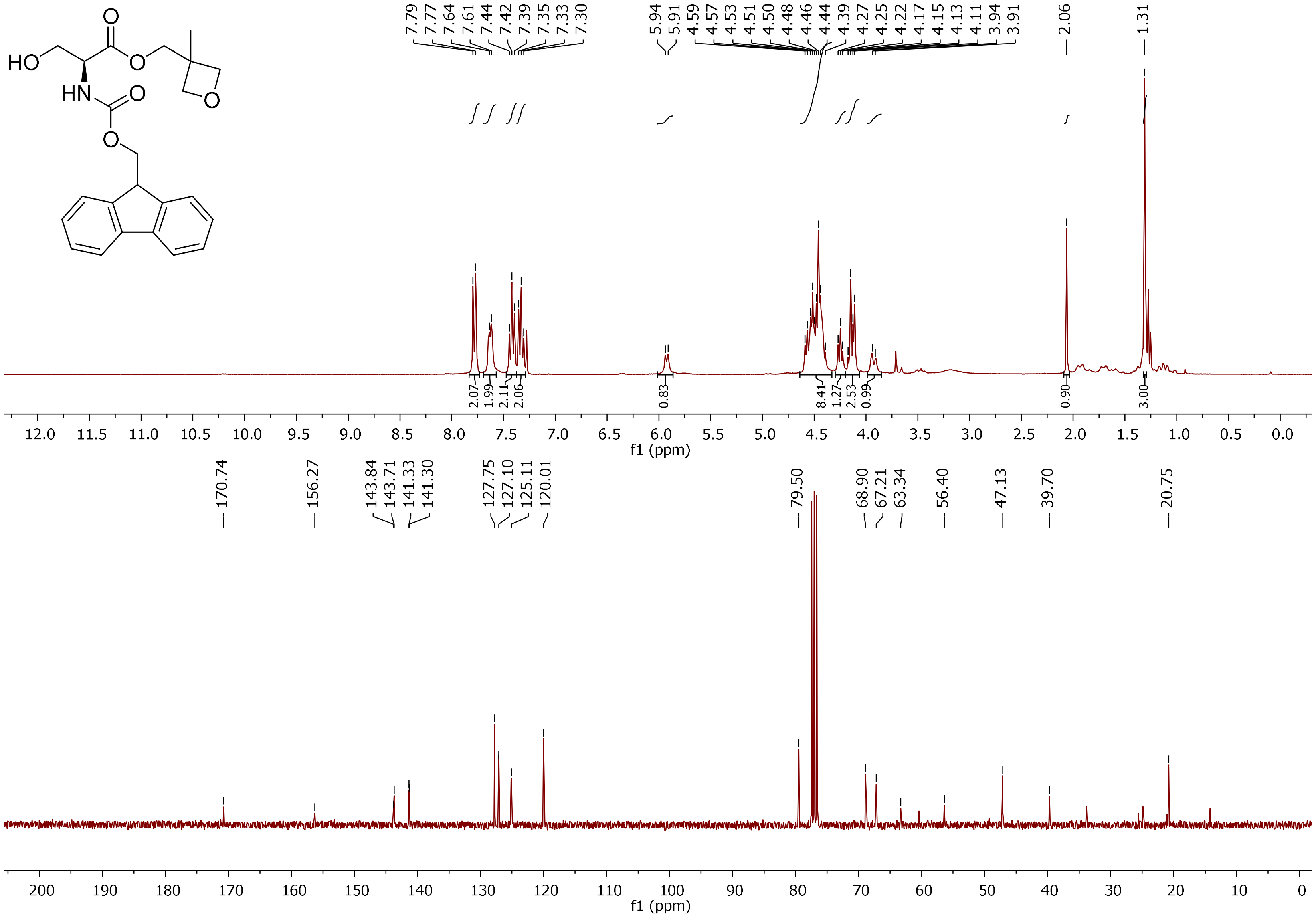

*Figure S101: ^{1}H and ^{13}C NMR spectra of Fmoc-L-Ser-oxetane ester **S5***

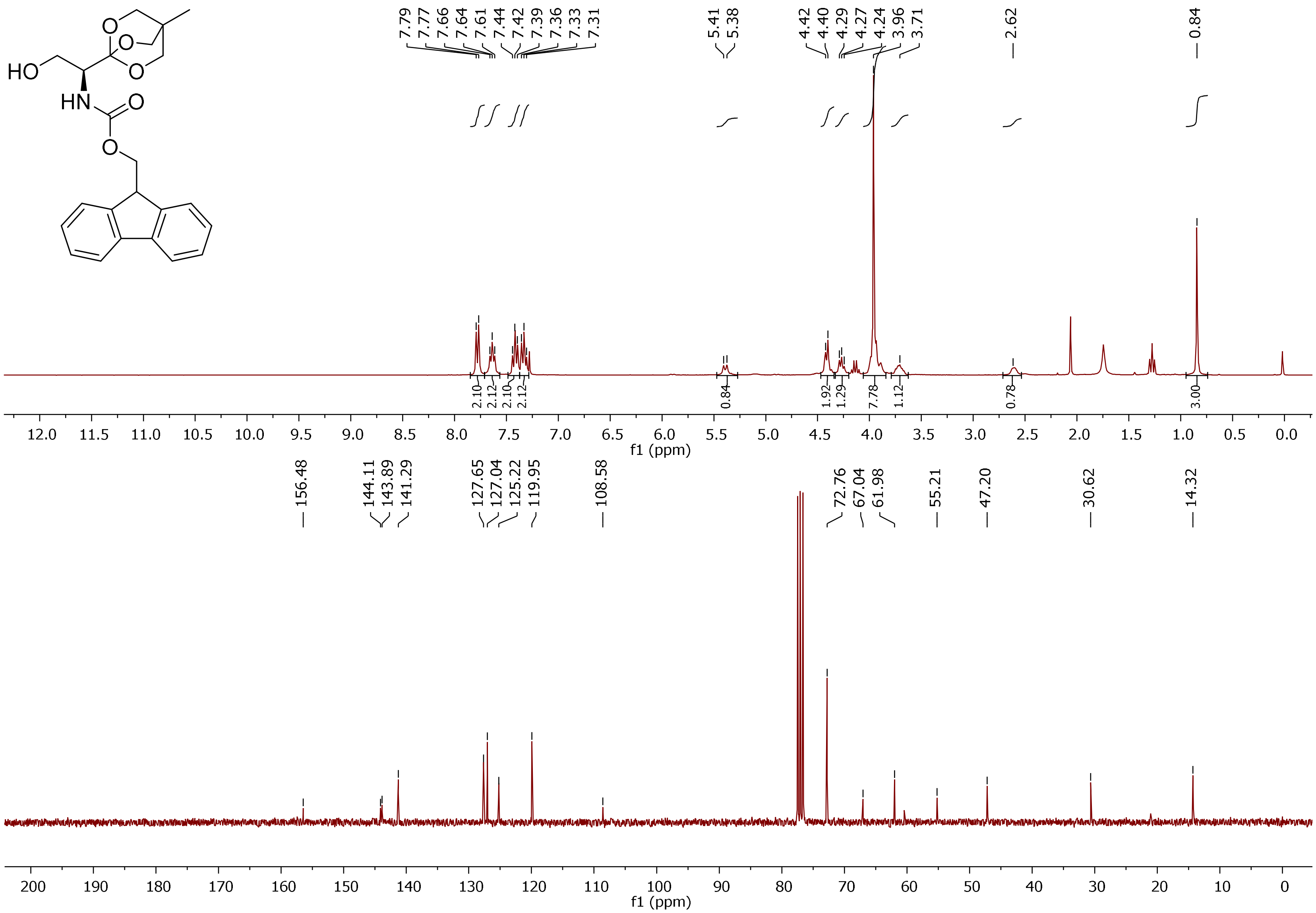

*Figure S102: ^{1}H and ^{13}C NMR spectra of Fmoc-L-Ser-OBO ester **S6***

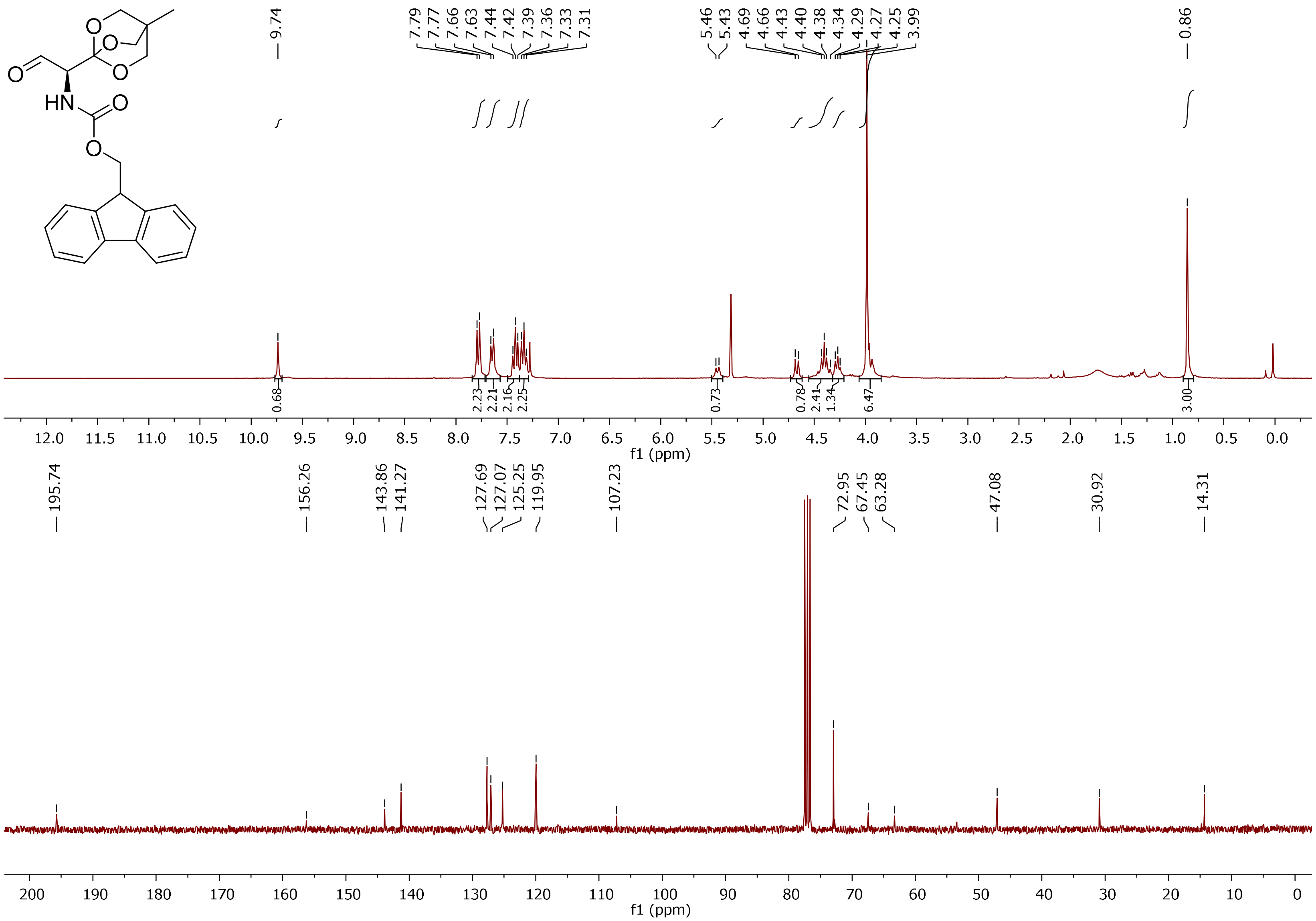

*Figure S103: ¹H and ¹³C NMR spectra of Fmoc-L-Ser(ald)-OBO ester **S7***

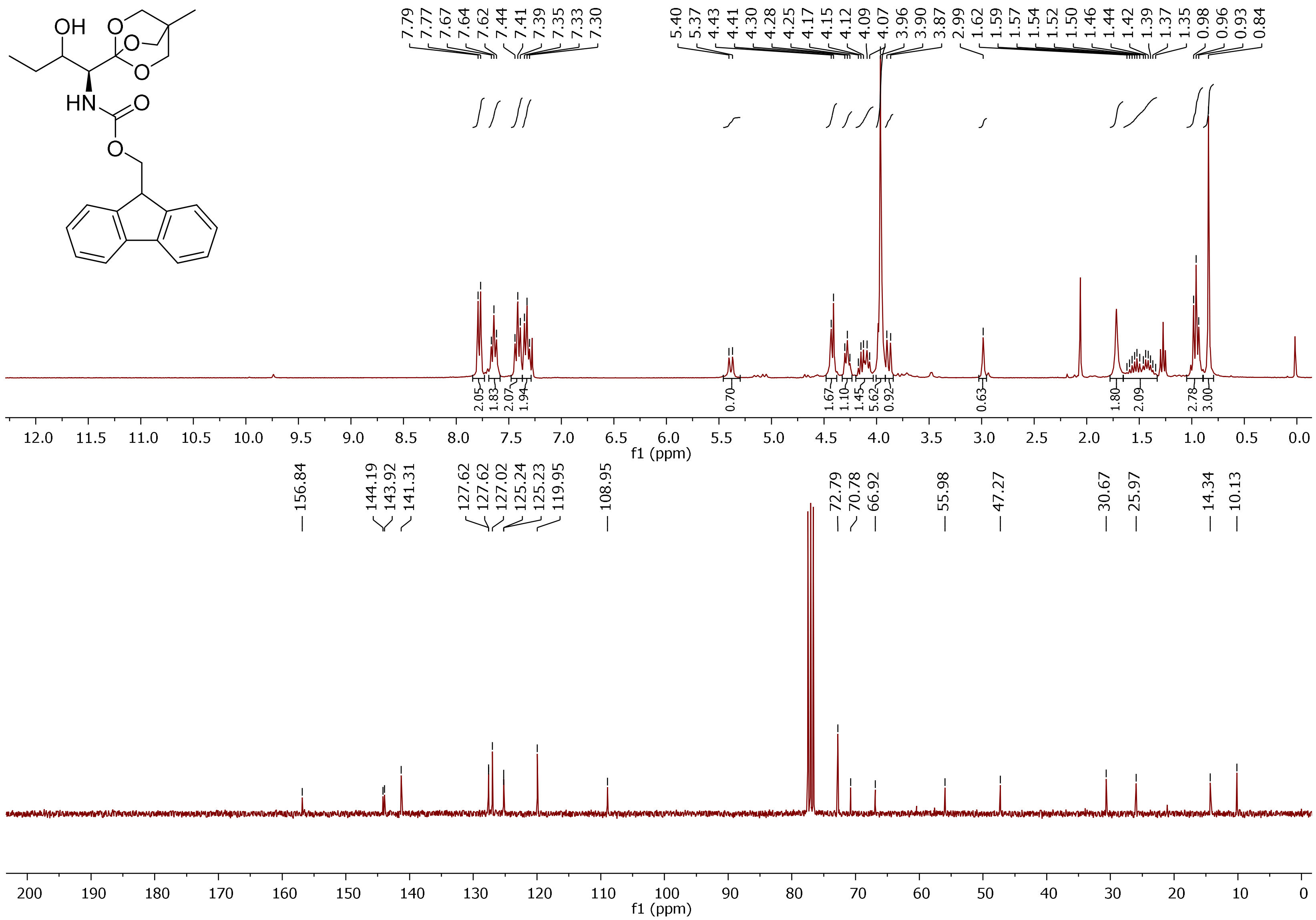

Figure S104: ^{1}H and ^{13}C NMR spectra of (9H-fluoren-9-yl)methyl ((1S)-2-hydroxy-1-(4-methyl-2,6,7-trioxabicyclo[2.2.2]octan-1-yl)butyl)carbamate ***S9***

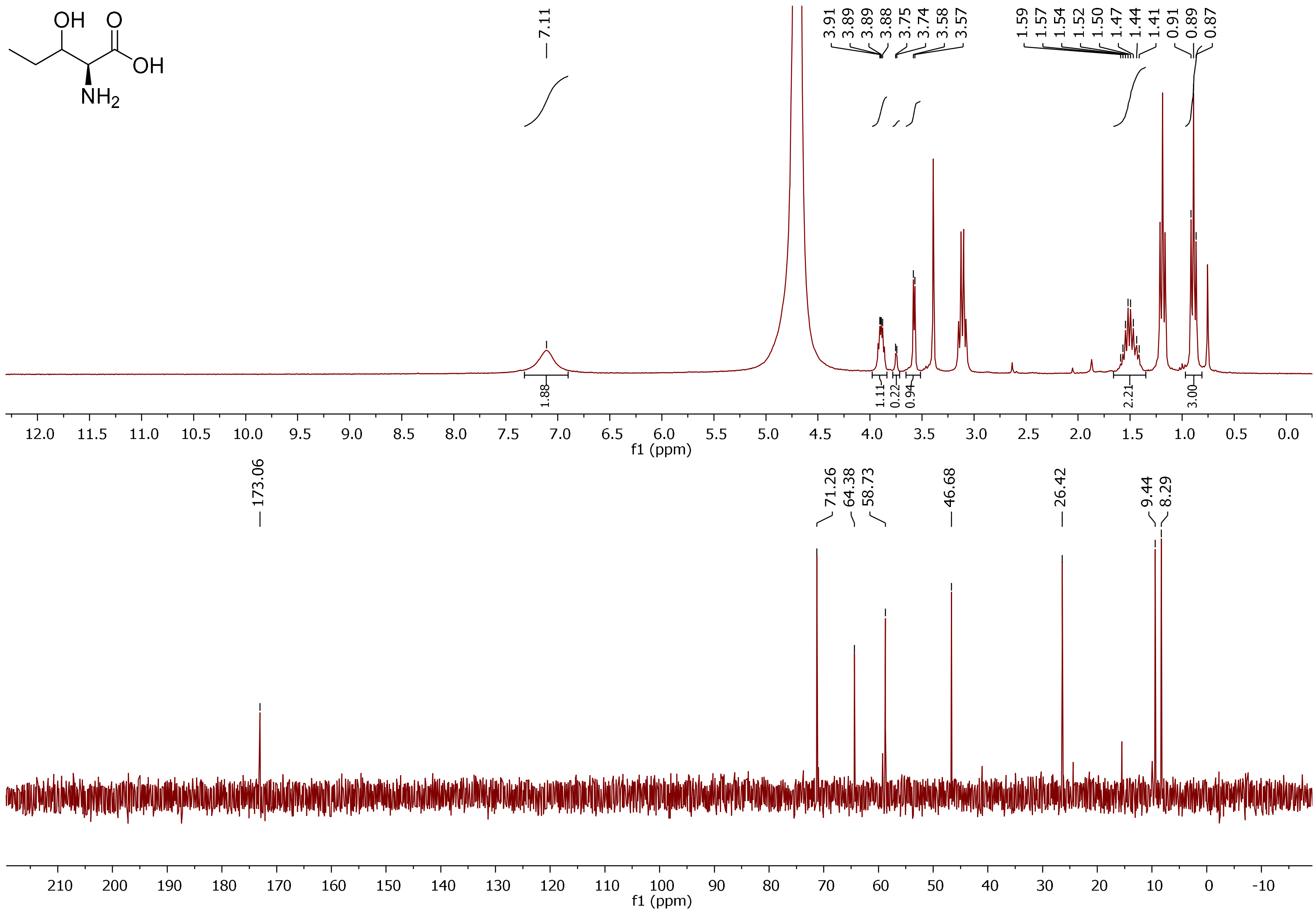

Figure S105: ¹H and ¹³C NMR spectra of ß-hydroxynorvaline ***S10***

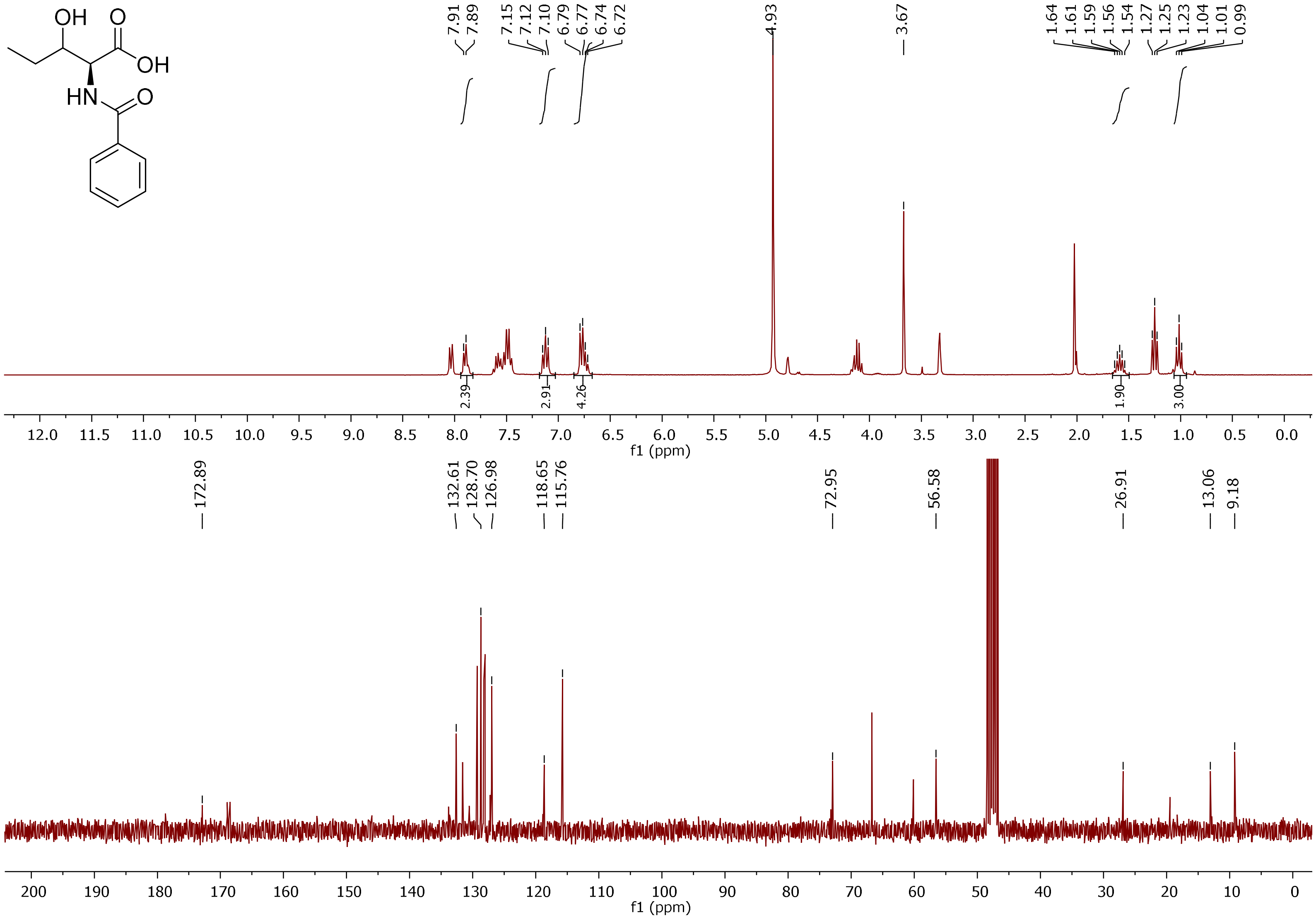

Figure S106. ^{1}H and ^{13}C NMR spectra of N*-benzoyl-ß-hydroxynorvaline **S11***

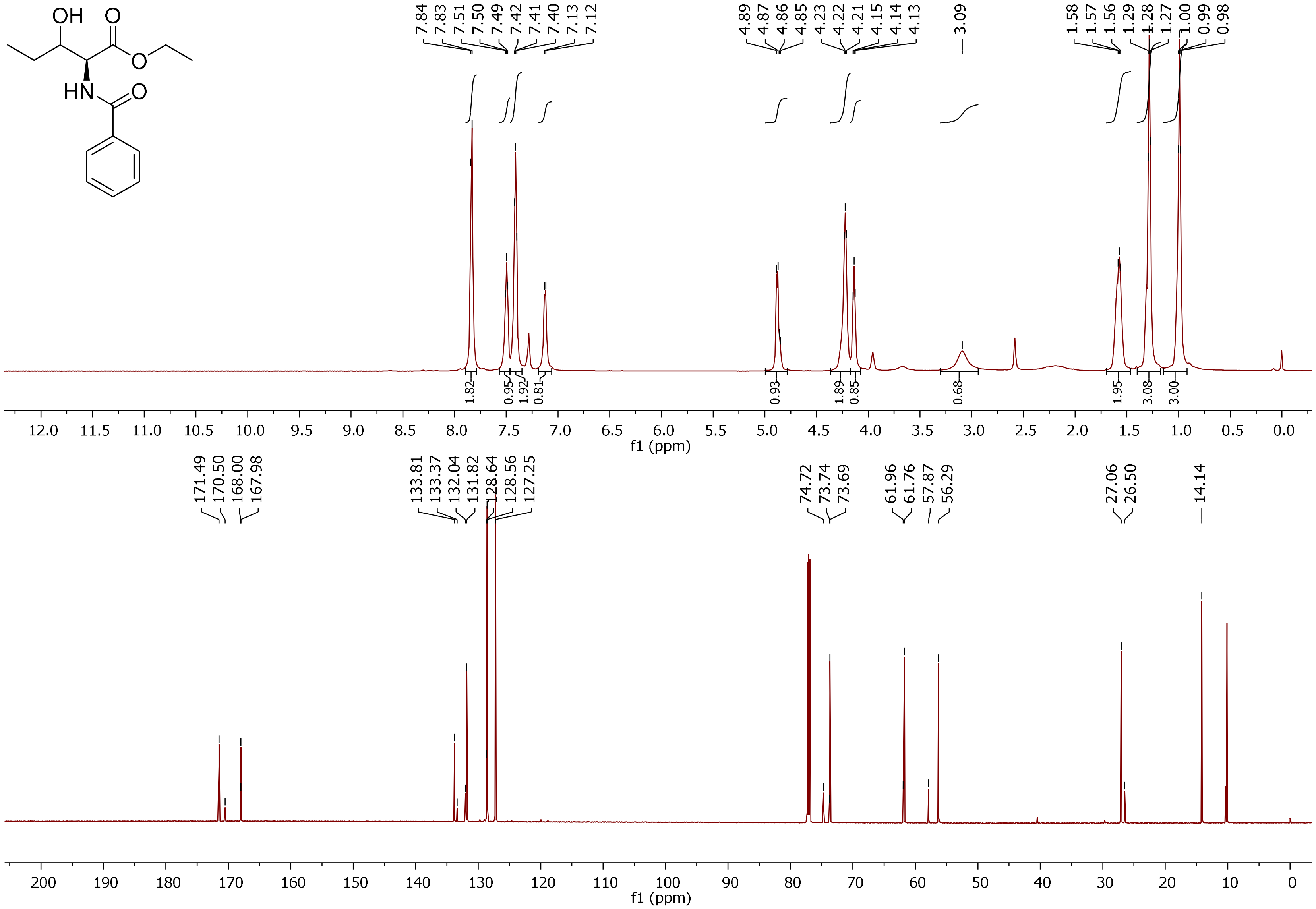

*Figure S107: ¹H and ¹³C NMR spectra of ethyl (2S)-2-benzamido-3-hydroxypentanoate (**N-Bz-4b**)*
